# Traditional knowledge 10 min far from Barcelona: ethnobotanical study in the Llobregat river delta (Catalonia, NE Iberian Peninsula), a heavily anthropized agricultural area

**DOI:** 10.1186/s13002-023-00615-2

**Published:** 2023-09-26

**Authors:** Jon Marín, Teresa Garnatje, Joan Vallès

**Affiliations:** 1 Laboratori de Botànica – Unitat associada al CSIC, Facultat de Farmàcia i Ciències de l’Alimentació, Universitat de Barcelona, Avinguda Joan XXIII 27-31, 08028 Barcelona, Catalonia Spain; 2 Institut Botànic de Barcelona (IBB), CSIC-Ajuntament de Barcelona, Passeig del Migdia S/N, 08038 Barcelona, Spain; 3 Secció de Ciències Biològiques, Institut d’Estudis Catalans, Carrer del Carme 47, 08001 Barcelona, Catalonia Spain

**Keywords:** Agricultural periurban areas, Ethnobotanical indexes, Folk agrosilvopastoral uses, Folk functional food, Spain

## Abstract

**Background:**

The right floodplain at the Llobregat river delta (Catalonia, NE Iberian Peninsula) constitutes an agricultural periurban area adjacent to Barcelona, which has remained ethnobotanically unexplored until now. This area comprises a very heavily anthropized mosaic of soil uses—urban, industrial, natural, agricultural—including the Agricultural Park of Baix Llobregat. The main aim of this work has been to collect and analyze the ethnoflora of this area in order to fill a gap in the ethnobotanical knowledge in industrialized areas.

**Methods:**

The followed methodology has been based on semi-structured interviews. The obtained data have been qualitatively and quantitatively analyzed and compared with other studies.

**Results:**

Data have been gathered from 83 informants. The interviewed informants referred 1965 use reports from 292 taxa, including both non-cultivated and cultivated species, from 85 botanical families. Among those, 451 were referred to medicinal uses, 1247 to food uses and 267 to other uses. In the present study, 779 vernacular names have been reported for 287 taxa. In addition to medicinal and food uses, this study significantly enhances our understanding of some agrosilvopastoral uses of plants, artistic use of plants and insights into folk functional foods. In this regard, we propose a novel quantitative ethnobotany index (the folk functional food index) to assess the relative significance of taxa employed as folk functional foods.

**Conclusions:**

The findings of this study highlight the enduring presence of ethnobotanical knowledge in this periurban agricultural region and underscore the significance of its preservation.

**Supplementary Information:**

The online version contains supplementary material available at 10.1186/s13002-023-00615-2.

## Introduction

Plants are used by humans from immemorial times. From 60,000 years ago, evidences have been suggested of this use, which most probably started much earlier [1]. Since its first definition [2], ethnobotanical research, dealing with peoples’ plant naming, using and managing, was conducted all over the World. The Catalan linguistic area (CLA) is one of the most studied territories in Europe at the level of traditional knowledge on plant biodiversity [3].

However, both in this territory [4] and worldwide [5], these studies have historically focused on rural regions. However, there is an urgent need to study areas in industrialized countries, especially in southern Europe, where the alteration of the physical and biological environment, rural depopulation and the new means of communication are causing an accelerated loss of traditional knowledge [6, 7]. Among particularly interesting areas, urban agglomerations, including not only cities but also their metropolitan areas, hold significant relevance [8]. Likewise, Pardo de Santayana et al. [9] encourage researchers to conduct ethnobotanical studies in metropolitan areas throughout Europe.

Urban ethnobotany is a relatively novel and specialized subdiscipline [8], with limited international literature currently accessible on this subject. Consequently, certain studies concentrate on urban markets [10–13], while others explore urban home gardens [14–17], cross-cultural adaptations [18–21] or semirural regions within industrialized environments [22–24].

The current study centers on a predominantly agricultural periurban region adjacent to Barcelona (Catalonia, Iberian Peninsula). According to Pochettino et al. [25], agrourban areas offer a favorable environment for the advancement of ethnobotanical studies. This is mainly attributed to the substantial coexistence of the productive, industrial and residential sectors, along with the botanical diversity of specific and infraspecific taxa.

The aim of this work is to identify the plant lore within the right floodplain of the Llobregat river delta (NE Iberian Peninsula). This pursuit serves a dual purpose: (1) to enhance ethnobotanical knowledge in the Catalan linguistic area (CLA) and the Mediterranean basin, and (2) to further the ethnobotanical understanding of agricultural and periurban landscapes.

## Material and methods

### Study area

**Fig. 1:**
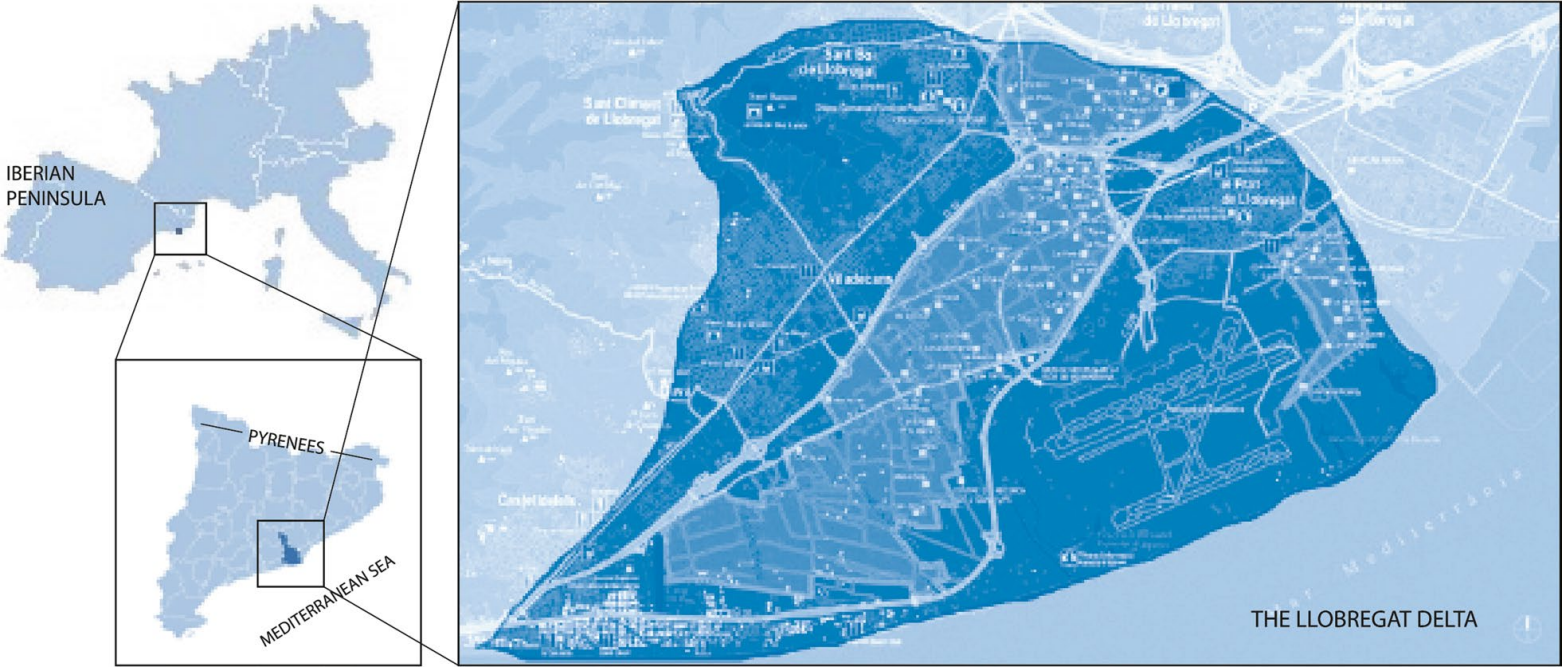
Studied area in Europe and Catalonia. On the right, dark-blue area corresponds to municipalities studied. Font: own elaboration

The studied area is located in the central coast of Catalonia (Fig. 1), in its turn situated in the NE Iberian Peninsula. It comprises the right floodplain at the Llobregat river delta. It covers an area of 127.71 km^**2**^ and is inhabited by 335,759 residents, leading to a population density of 2629.07 individuals per km^**2**^. Details about the study area are provided in the supplementary material (Additional file 1).

The study area features a Mediterranean climate. Temperature is significantly regulated by its proximity to the sea, resulting in mild winters and moderately warm summers, characterized by relatively moderate temperature fluctuations. The average annual temperature is 16.5 °C [26]. The average annual rainfall amounts to 612.38 mm, often concentrated in brief yet intense periods [27].

The vegetation features of the studied area were described by Blanco et al*.* [28]. Four major plant community groups can be differentiated: halophilic communities, associated with soil salinity; psammophilic communities, linked to coastal sands; hygrophilous communities, influenced by water presence; and anthropic communities, connected to agricultural and urban settings. However, human influence is highly significant, and some of the plant communities are found in disturbed areas to a greater or lesser extent. [29].

Historically, the area’s roots extend to the twelfth century, driven by agriculture. During the eighteenth century, landscape changes, including marshland drainage. Water innovations further enabled irrigated farms by the nineteenth century [30]. These transformations accelerated in the twentieth century due to industrialization and immigration [31], resulting a significant urban growth and environmental impact [32, 33]. Currently, the study area is part of the periurban area of Barcelona and the interdependence with Barcelona and its extensive area of influence is highly significant [34]. Regarding the economy, 44% of the population is engaged in the tertiary sector, while only 1% is involved in the primary sector [35], experiencing clear regression. Despite its limited economic impact, agriculture holds significant social importance within the study area, primarily due to the creation of the Baix Llobregat Agricultural Park (BLAP) in the 1990s, aimed at conserving and enhancing the region's territorial values [36]. BLAP has gained recognition and has been extensively studied as a Spanish model for preserving agricultural activities in a highly urbanized setting [33, 37–39].

### Field work

Field work was conducted from May 2015 to August 2018 throughout the study area. We carried out 80 interviews to 83 informants: 77 interviews were individual and three were concerning two people each, no one implying a bigger group. For interviews, we selected local experts working, either currently or in the past, as farmers who, because of their greater age, occupation, family tradition or personal interests, were most likely to have retained ethnobotanical knowledge. We applied a snowball sampling approach [40, 41] asking the informants to indicate further people experienced in traditional plant use. We conducted semi-structured interviews to foster communication and facilitate memory flow [3, 42].

In all, 42 men and 41 women born between 1918 and 1994 were interviewed. Regarding their occupations, the most notable professions include farmers (35%), fruit and vegetable merchants (19%), and individuals involved in household activities (10%). About 65% of the interviewed informants were born in the same region (Baix Llobregat), while the rest have spent a significant portion of their lives there. The notable presence of informants not originally from the studied area, coming from other parts of Spain, can be attributed to the historical waves of migration that the Baix Llobregat region has experienced [31].

Ethical principles of the International Society of Ethnobiology [43] were taken into account and oral informed consent was provided by informants. Ethnobotanical survey was conducted by adopting the guidelines on ethnopharmacological studies [44, 45]. All information was registered, transcribed and introduced into our research group database (http://gestio.etnobotanica.cat).

The plant taxa cited by the informants were identified mainly using *Flora Manual dels Països Catalans* [46], which was followed for taxonomy and nomenclature. *Flora agricola* [47] was used as a supplement to identify some cultivated plants. These floras represent the study area and are frequently used in many other works related to this region. In Additional file 2, the equivalence with Plants of the World Online [48] is given. APG IV [49] was adopted for botanical families. The herbarium vouchers have been deposited in the herbarium BCN (Centre de Documentació de Biodiversitat Vegetal, Universitat de Barcelona). In the ethnobotanical catalog (see Additional files 2 and 3, in original language), codes of each taxon from the herbarium are indicated.

### Data analyses

With the aim of assessing the state of ethnobotanical knowledge in the studied area, the following indexes were calculated. The ethnobotanicity index (EI) [50], expressed as a percentage, is calculated as the ratio between the number of plants used and the total number of plants constituting the territory’s flora, previously studied by González et al. [29]; the informant consensus factor (F_IC_) [51], which is the quotient between the number of medicinal use reports minus the number of used medicinal plants and the number of medicinal use reports minus one. This indicates the degree of reliability of the uses claimed (higher when closer to 1); the relative frequency of citation (RFC), which is obtained by dividing the number of informants who mention the use of the species by the number of informants. An RFC close to 1 indicates that a plant is widely known and used in the community, while an RFC close to 0 suggests that the plant is less known or rarely used [52]; the index of medicinal importance (MI), which is obtained by dividing the total of the use reports (UR) cited for a specific use-category by the number of taxa that have this use. MI is useful to evaluate the real importance of the use, as a specific use can be cited for a few or many species and this may change the relevance of the information [53].

To assess the relative importance of taxa utilized as folk functional food, we introduce a novel metric: the folk functional food index (FI). This index is computed using the following equation,

$${\text{FI}} = \frac{{{\text{UR}}_{{\text{F}}} \times {\text{UR}}_{{\text{M}}} }}{{{\text{UR}}_{{\text{T}}} }},$$

where UR_F_ is the number of food use reports, UR_M_ is the number of medicinal use reports, and UR_T_ represents the total number of use reports attributed to a specific taxon.

The outcome provides a relative gauge of the frequency of food use reports compared to medicinal use reports, considering the total number of use reports. A higher result signifies a greater recognition of a taxon as a functional food.

Regarding vernacular names, ethnophytonymy index [54] was calculated.

This index reflects the percentage of taxa with folk names. Additionally, we determined the allochthonous ethnophytonomy index [53] to gauge the proportion of taxa cited in languages other than Catalan. Furthermore, linguistic diversity index [55] was calculated by dividing the number of folk names by the number of taxa reported. This index illustrates the cultural richness of the folk plant knowledge.

## Results and discussion

### Plant species, use reports and botanical families

Data from 292 taxa belonging to 85 botanical families were collected in the present study, resulting in a total of 1,965 use reports (UR). All the data grouped by uses are included in the taxa catalog (see Additional file 3).

Regarding number of taxa, the best-represented families are Asteraceae (9%), followed by Poaceae (8%), Rosaceae (7%), Brassicaceae (5%), Lamiaceae (5%), Fabaceae (5%) and Apiaceae (5%). Regarding use reports, the same families are the most reported (in a different order) (Table 1).

**Table 1:** Botanical families, number of taxa and use reports (UR)

Family	Number of taxa	% taxa	UR	% UR
Asteraceae	26	8.90	168	8.55
Poaceae	23	7.88	101	5.14
Rosaceae	19	6.51	173	8.80
Brassicaceae	15	5.14	116	5.90
Lamiaceae	15	5.14	149	7.58
Fabaceae	15	5.14	122	6.21
Apiaceae	15	5.14	104	5.29

These results align with studies conducted in other agricultural regions of the Mediterranean basin [23, 56]. Furthermore, apart from Brassicaceae, the best-represented families are consistent with those observed in other surveyed regions of the CLA, owing to their considerable diversity and widespread distribution [22, 57, 58].

### Quantitative ethnobotany

Some quantitative ethnobotany indexes concerning 10 territories (including the one here studied) of the CLA are presented in Table 2. The ethnobotanicity index (EI) for the studied area is 25.50%, indicating that approximately one-quarter of the plants in the area have been reported as useful by the informants. This places it in an intermediate position within the range of values obtained for other CLA. The informant consensus factor (F_IC_) of medicinal information obtained (0.74) is among the lowest values observed in the quoted areas. However, these are common values in rural areas of the Mediterranean Basin, such as the Greek Aegean Islands (0.72) [59] or northeastern Algeria (0.72) [60]. While the interpretation of F_IC_ values should be approached cautiously, taking into account factors like culture, social context and methodology, the ability to compare these values on an equal footing leads us to believe that significant local ecological knowledge is still preserved in agricultural periurban regions like this one.

**Table 2:** Quantitative ethnobotany indexes in 10 territories (in bold, the one here studied) in the Catalan linguistic area

Studied areas	Extension (km^2^)	Population	Flora	MP	MP/km^2^	MP/inhab	MP/I	EI	F_IC_
Alt Empordà^a^	1358	118,718	1650	334	0.25	0.28 × 10^–2^	1.87	25.90	0.91
**Baix Llobregat (deltaic floodplain)**	**127.71**	**335,939**	**1144**	**117**	**0.92**	**0.035 × 10**^**–2**^	**1.41**	**25.50**	**0.74**
Castelló^b^	6679	385,283	2128	365	0.06	0.095 × 10^–2^	2.34	17.20	–
Cerdanya^c^	1140	26,250	1500	146	0.13	0.56 × 10^–2^	4.56	9.70	0.93
Garrigues^d^	1123	22,243	1500	196	0.17	0.88 × 10^–2^	1.94	23.47	0.89
Gironès^e^	187	10,659	1,500	137	0.73	1.29 × 10^–2^	2.40	22.56	0.86
Eastern Mallorca^f^	238	31,764	780	121	0.51	0.038 × 10^–2^	2.88	15.51	0.71
Montseny^g^	826	79,373	1500	351	0.42	0.44 × 10^–2^	1.95	23.20	0.91
Pallars^h^	2530	18,880	1500	437	0.17	2.32 × 10^–2^	1.66	29.10	0.87
Ripollès^i^	957	25,700	1600	282	0.30	1.10 × 10^–2^	1.73	28.60	0.96

Flora: approximate number of the species of vascular plants of the flora of the territory; MP: number of reported medicinal plants; MP/I: number of medicinal plants reported by informant; EI: ethnobotanicity index; F_IC_: informant consensus factor

Fonts: ^a^[57]; ^b^[61]; ^c^[62]; ^d^[63]; ^e^[22]; ^f^[53]; ^g^[64]; ^h^[65]; ^i^[58]

### Medicinal uses

Our informants mentioned 117 species with medicinal uses, representing 45 botanical families and 451 use reports. Among these, 99.12% are referring to human medicine, 0.44% to veterinary medicine and an additional 0.44% to both contexts. The average number of medicinal taxa cited by informant stands at 1.41, exhibiting striking similarity to other urban agricultural areas (1.28) [23]. Furthermore, these figures approach those derived from rural territories within the CLA surveyed [45, 46]. This underscores the assertion presented in Gras et al. [22] that ethnobotanical knowledge is still relevant in industrialized areas, even in comparison with non- or less-industrialized territories.

**Fig. 2:**
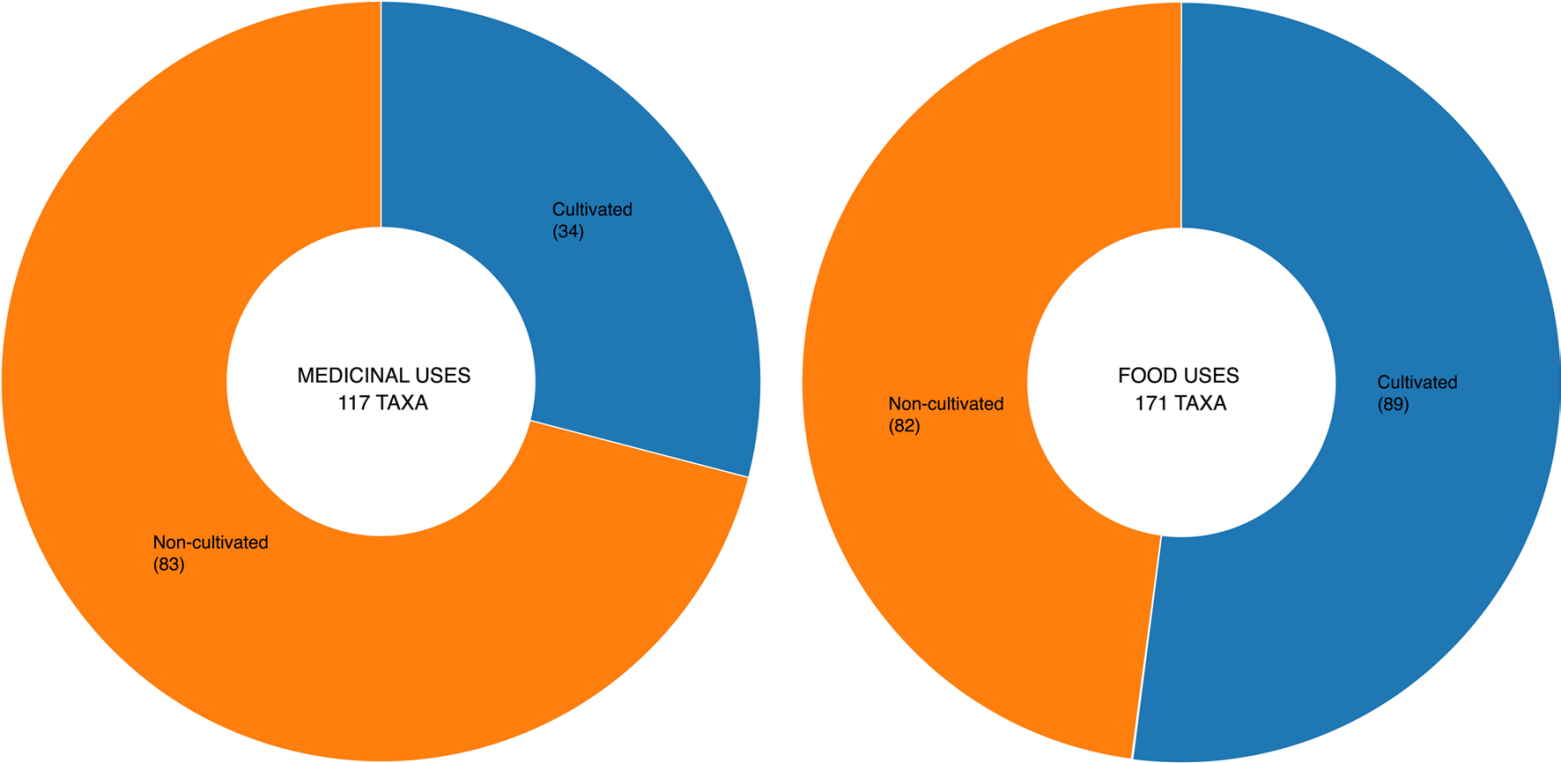
Sources of medicinal use (left) and food use (right) taxa reported

The 20 most cited species are included in Table 3. They represent the 52% of medicinal use citations. *Thymus vulgaris* is the most cited taxon, with a RFC of 0.205. Most of these taxa are among the most quoted in the rest of the CLA [22, 58, 63]. Furthermore, the presence of species abundant in ruderal environments stands out, such as *Malva sylvestris*, *Matricaria recutita* or *Foeniculum vulgare* subsp. *piperitum*. These findings align with those observed in other European periurban agricultural areas [23]. In addition to this, the medicinal use of *Cynara scolymus* is notable, as it is a cultivated species with a rich agricultural tradition in the area. In total, species cultivated for food that also have medicinal uses comprise 39%. (Fig. 2). These could be considered as folk functional foods, which are further discussed below.

**Table 3:** List of the 20 most cited species concerning medicinal uses

Taxon	Family	UR	UR (%)	RFC	C
*Thymus vulgaris*	Lamiaceae	33	7.30	0.205	No
*Malva sylvestris*	Malvaceae	17	3.77	0.169	No
*Cynara scolymus*	Asteraceae	17	3.77	0.133	Yes
*Matricaria recutita*	Asteraceae	15	3.33	0.145	No
*Foeniculum vulgare* subsp. *piperitum*	Apiaceae	13	2.88	0.133	No
*Eucalyptus globulus*	Myrtaceae	13	2.88	0.108	Yes
*Zea mays*	Poaceae	12	2.66	0.120	Yes
*Calendula officinalis*	Asteraceae	11	2.44	0.060	No
*Rosmarinus officinalis*	Lamiaceae	11	2.44	0.120	No
*Lippia triphylla*	Verbenaceae	11	2.44	0.072	Yes
*Sambucus nigra*	Adoxaceae	10	2.21	0.084	No
*Citrus limon*	Rutaceae	10	2.21	0.084	Yes
*Ruta graveolens*	Rutaceae	9	1.99	0.108	No
*Urtica dioica*	Urticaceae	9	1.99	0.060	No
*Borago officinalis*	Boraginaceae	8	1.77	0.024	No
*Origanum vulgare*	Lamiaceae	8	1.77	0.048	No
*Lavandula dentata*	Lamiaceae	8	1.77	0.036	No
*Mentha pulegium*	Lamiaceae	7	1.55	0.072	No
*Laurus nobilis*	Lauraceae	7	1.55	0.036	Yes
*Equisetum arvense*	Equisetaceae	6	1.33	0.060	No

*UR* use reports, *UR (%)* relative use reports, *RFC* relative frequency of citation, *C* cultivated

Concerning botanical families, Lamiaceae is the most cited (19%), followed by Asteraceae (16%), Apiaceae (7%) and Rosaceae (5%). These results agree with the general family distribution of the area. Lamiaceae and Asteraceae have a high number of representatives in the Mediterranean flora and Rosaceae include some fruit trees. The three of them agree with other ethnobotanical studies of Mediterranean areas [6]. In other regions of the Mediterranean basin, the Apiaceae family similarly emerges as one of the most prominently represented in terms of medicinal use reports [60, 66].

The results from the most reported parts align with those observed in other regions of the CLA, irrespective of geographic conditions [63–65]. The aerial parts (including the young aerial, sterile aerial, flowering aerial and fructified aerial parts) are the most frequently cited, accounting for 46% of UR. Fronds and leaves constitute 23% of UR, while fruits (including fruit juice like, for instance, from *Citrus limon*) comprise 12%. Generally, these are the plant parts that allow an easier identification to informants and that present less collection and conservation difficulties.

**Fig. 3:**
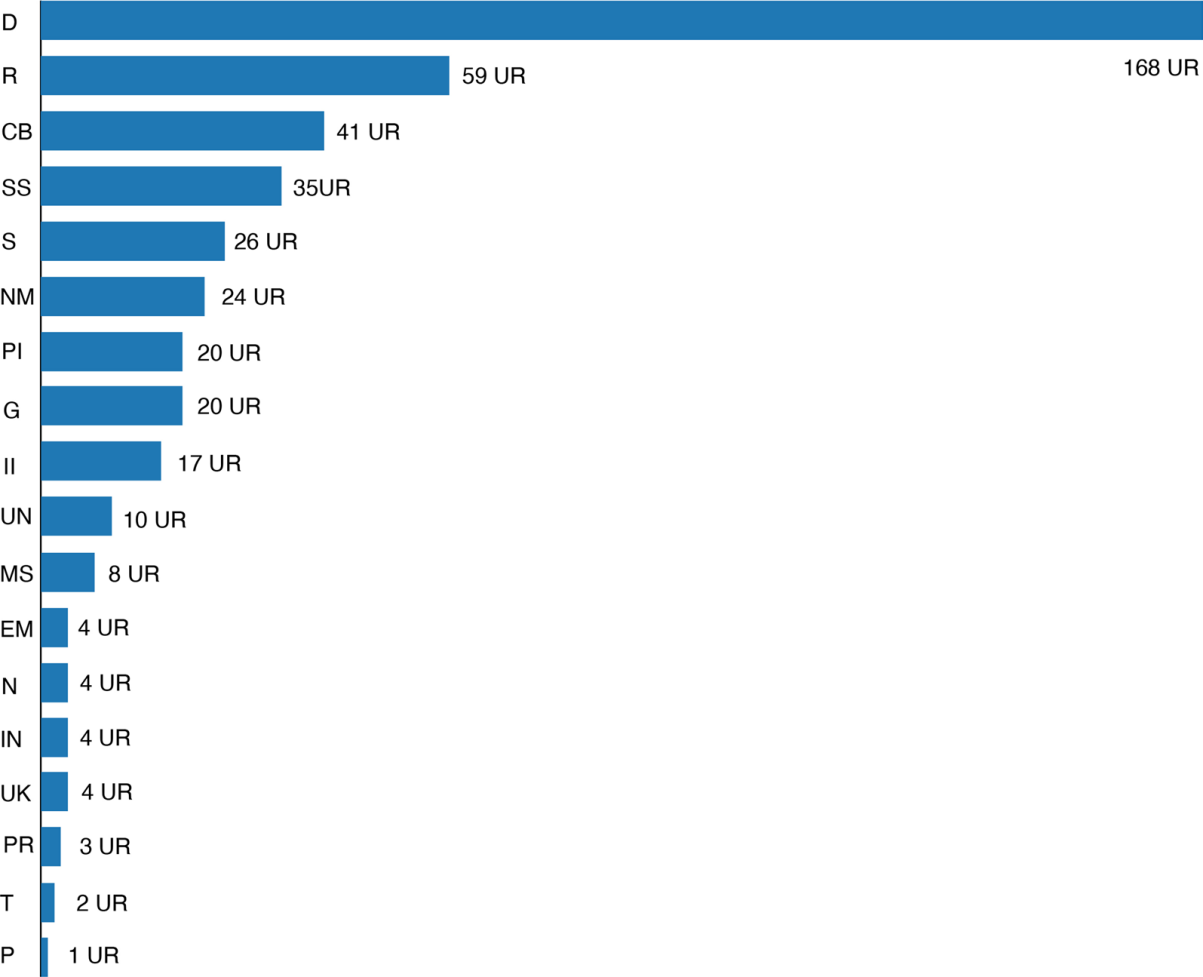
Systems and disorders addressed with medicinal plants in the area studied (UR). *D* digestive system disorders, *R* respiratory systems disorders, *CB* circulatory system and blood disorders, *SS* skin and subcutaneous system disorders, *S* sensory systems disorders, *NM* nervous system and mental disorders, *PI* pain and inflammations, *G* genitourinary system disorders, *II* infections and infestations, *UN* unspecific disorders, *MS* musculoskeletal system disorders, *EM* endocrine system and metabolic disorders, *N* nutritional disorders, *IN* immune system disorders and neoplasia, *UK* unknown by the informant, *PR* pregnancy, birth and puerperal disorders, *T* traumatisms, *P* poisoning

The most treated disorders are those referred to digestive system, accounting for 37% of the total. Respiratory system disorders represent 13% of the total (Fig. 3). The same findings have also been highlighted by several studies conducted in CLA [22, 57, 58] and all around Mediterranean basin [6, 23, 56, 60]. Most of the remedies concern the treatment of unimportant pathologies [63]. The most cited digestive ailments are related to diuretic and hepatoprotective uses. In terms of respiratory diseases, colds and coughs are predominant.

The most cited taxon for digestive system disorders is *Cynara scolymus*, primarily used as a hepatoprotective. This species is extensively cultivated within the surveyed area. This fact reflects the idea that plants used in folk medicine are obtained from places close to those where the users live [3, 4, 54, 58].

When the index of medicinal importance (MI) is calculated, sensory system disorders have the highest results. As an example of sensory system disorders, it is relevant to mention eye problems, which are treated with some species such as *Thymus vulgaris* (7 UR), *Matricaria recutita* (6 UR), *Ruta graveolens* (5 UR) or *Sambucus nigra* (5 UR).

Regarding the pharmaceutical form, tisane—including decoction and infusion—represents 41% of the total forms reported, in line with the findings of other studies in the Mediterranean basin, including Spain, Morocco, Algeria and Italy [3, 6, 23, 56, 60, 66]. Direct use represents 16%. Thus, the simplest pharmaceutical forms employed are the most common, as indicated by Parada et al. [67]. Likewise, it is noteworthy to highlight the 19% of use reports cited (the second most represented category) where the pharmaceutical form is either not specified or is unknown by the informant. Interestingly, these reports are primarily not associated with the most frequently mentioned medicinal uses (Fig. 2). This observation might suggest that informants are aware of the medicinal properties of certain plants but are not actively using them, potentially due to a decline in traditional knowledge. Nevertheless, further investigation into this aspect would be necessary in future surveys.

In terms of medicinal mixtures, 18 recipes have been inventoried (54 UR, involving 34 taxa). The species most commonly employed include *Rosmarinus officinalis*, *Thymus vulgaris* and *Olea europaea* subsp. *europaea* var. *europaea*. These mixtures are predominantly employed for pain relief purposes, encompassing analgesic, antalgic and anti-inflammatory properties, along with applications for treating colds and tetanus. Specifically, concerning the latter therapeutic application, informants refer to a practice known in Catalan as “fer una palla” (literally translating to “make a straw”). This method involves utilizing a straw made from *Triticum aestivum* combined with olive oil (*Olea europaea* subsp. *europaea* var. *europaea*). This remedy was used for instances where animals or humans sustained puncture wounds. In this approach, oil is inserted into the straw and positioned vertically over the wound. The lower end of the straw is ignited, causing the oil to heat and subsequently trickle onto the wound, facilitating its healing process.

### Food uses

Food uses are the most reported in this area: 1247 UR of 171 taxa belonging to 55 botanical families. These findings show a distinct pattern in comparison with other ethnobotanical studies on useful plants, where medicinal uses tend to surpass food uses [57, 58, 64]. However, this aligns with a study by Gras et al. [22] in a semirural area near industrial zones. While interview biases might exist, this reflects the agricultural importance within the local community.

Regarding their usage, 85.19% of these taxa are for human consumption, 14.58% for animal feed, and 0.23% are used for both human and animal consumption. On average, each informant cites 2.06 food taxa, with an F_IC_ of 0.86—both values are higher, compared to those calculated for medicinal uses. Based on informant responses and participatory observation, it has been determined that among the mentioned plants, 52% are cultivated and the rest are wild or restored to a natural state (Fig. 2).

The Rosaceae family leads in terms of use reports (11%), followed by Asteraceae (10%), Solanaceae (8%) and Brassicaceae (8%). With the exception of Solanaceae, these families align with the broader family distribution in the area. Notably, these families hold considerable importance for food applications in Mediterranean countries like Spain, France or Italy [68–70].

The 20 species with the highest use reports are listed in Table 4. Notably, the most frequently cited taxa are cultivated species. This observation aligns with the logical assumption that cultivated species are primarily consumed as food and have been reported accordingly. Among the non-cultivated species, *Thymus vulgaris*, *Portulaca oleracea*, *Foeniculum vulgare* subsp. *piperitum* and *Borago officinalis* hold the 9th, 15th, 17th and 19th positions, respectively. It is worth mentioning that these non-cultivated species emerge as prominently cited, echoing findings from an ethnobotanical review of wild edible plants in Spain [70] and other Mediterranean areas [71]. Additionally, *Thymus vulgaris* has consistently appeared as one of the most cited species for food use in studies across various Mediterranean regions [57, 63].

**Table 4:** The top twenty taxa regarding human food uses

Taxon	Family	UR	UR (%)	RFC	C
*Solanum lycopersicum*	Solanaceae	39	3.40	0.386	Yes
*Allium cepa*	Amaryllidaceae	36	3.14	0.398	Yes
*Cynara scolymus*	Asteraceae	33	2.87	0.289	Yes
*Lactuca sativa*	Asteraceae	32	2.79	0.386	Yes
*Brassica oleracea* subsp. *oleracea* var. *capitata*	Brassicaceae	29	2.53	0.337	Yes
*Cucurbita maxima*	Cucurbitaceae	30	2.26	0.301	Yes
*Solanum tuberosum*	Solanaceae	25	2.18	0.253	Yes
*Beta vulgaris s*ubsp.v*ulgaris* var. v*ulgaris*	Amaranthaceae	24	2.09	0.277	Yes
*Thymus vulgaris*	Lamiaceae	24	2.09	0.229	No
*Allium sativum*	Amaryllidaceae	23	2,00	0.241	Yes
*Pyrus malus* subsp. m*itis*	Rosaceae	23	2,00	0.253	Yes
*Prunus persica*	Rosaceae	22	1.92	0.241	Yes
*Brassica oleracea* subsp. *oleracea* var. *botrytis*	Brassicaceae	21	1.83	0.253	Yes
*Phaseolus vulgaris*	Fabaceae	20	1.74	0.241	Yes
*Portulaca oleracea*	Portulacaceae	20	1.74	0.241	No
*Daucus carota* subsp. *sativus*	Apiaceae	19	1.66	0.217	Yes
*Foeniculum vulgare* subsp. *piperitum*	Apiaceae	18	1.57	0.169	No
*Prunus avium*	Rosaceae	18	1.57	0.169	Yes
*Borago officinalis*	Boraginaceae	17	1.48	0.145	No
*Cichorium endivia* subsp. *endivia*	Asteraceae	17	1.48	0.181	Yes

*UR* use reports, *UR (%)* relative use reports, *RFC* relative frequency of citation, *C* cultivated

Interestingly, *Portulaca oleracea* is linked with crops and is considered a weed by farmers. Recently, it has gained attention as a functional food due to its chemical composition [72]. Another significant species is *Ceratonia siliqua* (13 use reports, 1% of total), once a fading crop in Catalonia used for human and animal consumption. However, its nutritional value is still recognized, and the demand for it as human food is rising due to the trend for natural products [73]. Both species have valuable economic and environmental potential, making them worthy of market exploration for future research.

Fruit and infructescence are the most used plant parts (36%), followed by leaves (21%), aerial part (15%), and flowers and inflorescences (7%). Regarding the mode of consumption, the most common way is fresh (28%). The plants boiled (11%) and used as a condiment (8%) constitute the following categories.

**Fig. 4:**
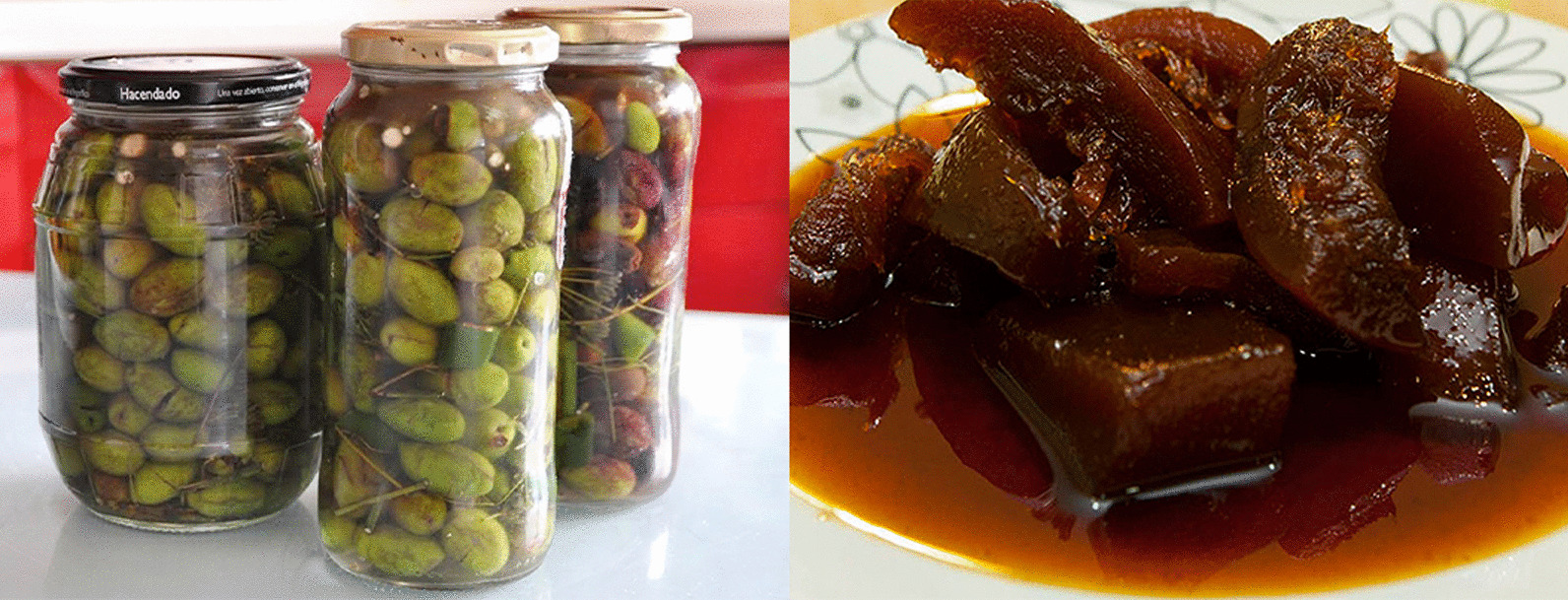
On the left, seasoned olives prepared with a mix of species. On the right, watermelon “arrop”

Regarding food mixtures, the predominant approach is focused on food preservation, accounting for 63% of the recipes. For instance, some recipes involve preserving olives (*Olea europaea* subsp. *europaea* var. *europaea*) in brine, along with other species like *Ceratonia siliqua*, *Satureja montana*, *Foeniculum vulgare* subsp. *piperitum*, *Allium sativum*, or *Citrus limon*. Likewise, an ancient Catalan practice called “arrop,” akin to confiture, is used to preserve fruits and vegetables. This involves boiling plant parts in wine [74]. Informants add sugar and fruits like *Prunus avium*, *Solanum melongena*, *Vitis vinifera* (grapes), *Cucumis melo* subsp*. melo*, *Citrus limon* and *Citrullus lanatus*. Typically, this approach involves repurposing fruits or their parts that are usually discarded due to their unappealing appearance (Fig. 4).

**Fig. 5:**
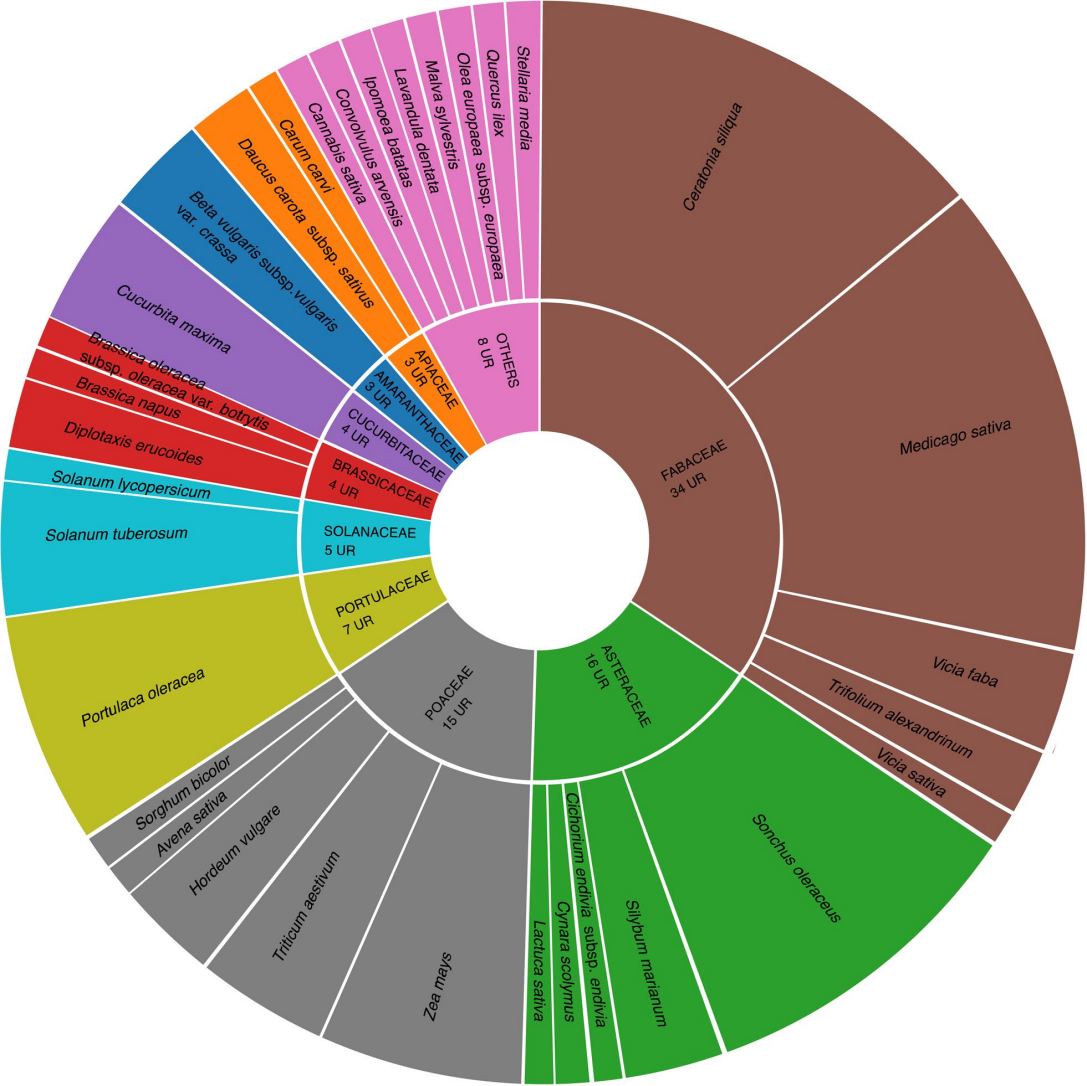
Botanical families and taxa for animal feed (UR)

Concerning animal fodder (Fig. 5), the Fabaceae family is most frequently mentioned (34%). The most cited taxa are *Ceratonia siliqua* (14%) and *Medicago sativa* (14%). *Ceratonia siliqua*, as mentioned earlier, was a widely cultivated crop in the Catalan region, and it was provided to horses and draft animals to supplement their energy intake. The commonly used plant parts include the aerial parts (39%), fruits (21%), raw or powdered seeds (17%) and underground parts (9%). Notably, 45% of the taxa used for animal feed are also cultivated for human consumption, suggesting that animals are often fed with discarded plants or parts. Of the taxa, 27% are intentionally grown for fodder, while the remaining 27% are wild plants.

### Folk functional food

In recent times, there has been a significant interest in food products that perform some healthful function in the human body [75–77]. In ethnobotany, the interface between the food and medicinal uses of plants is very permeable, so that many foods heal and many medicines nourish. It is what is called a popular, traditional or folk functional food [78–80]. In this study, 56% of reported medicinal plants also contribute to human nutrition, similar to findings in other Mediterranean studies [80].

The most relevant species identified as folk functional food are presented in Table 5. *Thymus vulgaris* occupies a preeminent position in this ranking, in line with findings by Vallès et al. [79] and Rivera et al. [71]. Except for *Allium cepa*, the remaining species are also prominent in terms of medicinal uses (as discussed earlier), suggesting a perceived continuum between medicinal and food plants [23, 77, 80, 81].

**Table 5:** The top ten taxa regarding folk functional food consideration index (FI)

Taxon	UR_F_	UR_M_	FFI
*Thymus vulgaris*	24	33	13.89
*Cynara scolymus*	34	17	11.33
*Foeniculum vulgare* subsp.* piperitum*	18	13	7.55
*Rosmarinus officinalis*	14	11	6.16
*Borago officinalis*	17	8	5.44
*Citrus limon*	11	10	5.24
*Allium cepa*	36	6	5.14
*Malva sylvestris*	7	17	4.96
*Laurus nobilis*	13	7	4.55
*Zea mays*	7	12	4.42

*UR*_*F*_ food use reports, *UR*_*M*_ medicinal use reports

When comparing the list of folk functional food in our study area with the encompassing CLA [79], it emerges that 77% of taxa have already been documented in prior studies, indicating their concurrent roles in both medicinal and food uses. Conversely, it is noteworthy that species such as *Ceratonia siliqua*, *Anethum graveolens* or *Cucurbita maxima* are being highlighted for the first time within the context of the CLA as folk functional food.

### Other uses

This category encompasses uses that are neither medicinal nor related to food. A total of 267 use reports are collected, concerning 111 taxa from 46 families. The average number of taxa mentioned per informant is 1.34. The most used taxa in this category are outlined in Table 6. The most frequently taxa cited are *Arundo donax*. This species is associated with agricultural management, particularly for supporting the growth of climbing cultivated plants. *Arundo donax* is also prominently cited in other regions with similar characteristics [22, 63]. Within this category, Poaceae emerges as the family with the highest UR (18%), followed by Fabaceae (12%). These families are also well represented in the broader distribution.

**Table 6:** List of the 10 most cited species regarding other uses

Taxon	Family	UR	UR%	RFC	C
*Arundo donax*	Poaceae	25	9.36	0.205	No
*Zea mays*	Poaceae	9	3.37	0.096	Yes
*Laurus nobilis*	Lauraceae	8	3.00	0.096	Yes
*Ulex parviflorus*	Fabaceae	8	3.00	0.060	No
*Lavandula dentata*	Lamiaceae	7	2.62	0.060	No
*Pinus halepensis*	Pinaceae	7	2.62	0.072	No
*Spartium junceum*	Fabaceae	7	2.62	0.072	No
*Phormium tenax*	Asparagaceae	6	2.25	0.060	Yes
*Symphytum officinale*	Boraginaceae	5	1.87	0.024	Yes
*Juncus acutus*	Juncaceae	4	1.50	0.048	No

*UR* use reports, *UR (%)* relative use reports, *RFC* relative frequency of citation, *C* cultivated

**Fig. 6:**
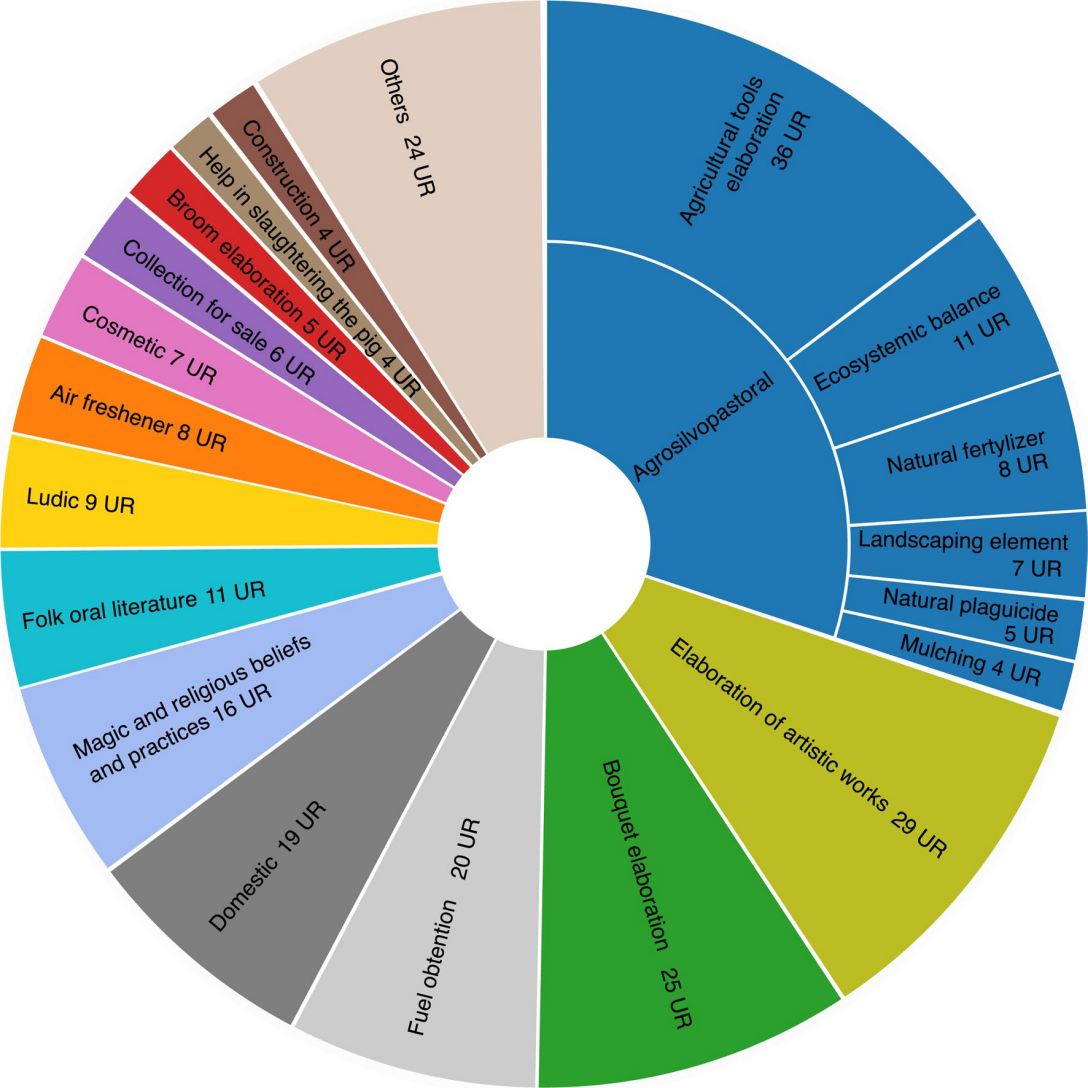
Other uses categories and subcategories (UR)

The most frequently mentioned uses are related to agrosilvopastoral applications (28%), followed by the creation of artistic works (11%) (Fig. 6). The significant use in agrosilvopastoral management is influenced by the area's agricultural nature. Some subcategories are incorporated within the agrosilvopastoral category, and the top cited use is the crafting of agricultural tools (13%). These tools primarily serve two purposes: (1) to support the growth of cultivated species (mainly tomatoes, beans and peas) using exclusively *Arundo donax* stems, and (2) for tying other cultivated species (mainly lettuce, chards and cabbages) with materials such as *Spartium junceum*, *Phormium tenax or Juncus acutus*. Other subcategories within the agrosilvopastoral category also hold significance due to their historical and current applications in non-intensive agricultural practices: ecosystemic balance (5%) involves growing certain species beneficial to crops; specific plants, like *Symphytum officinale* or *Equisetum arvense*, are used as natural fertilizers (4%); some contribute to the landscape's formation (3%) as natural enclosures or erosion-protecting soil barriers, such as *Opuntia maxima* (2 UR); some plants function as natural pesticides (2%) or are employed for mulching (1%), which entails applying crushed plant material to the ground to shield it from harsh weather conditions (excessive sun or frost).

**Fig. 7:**
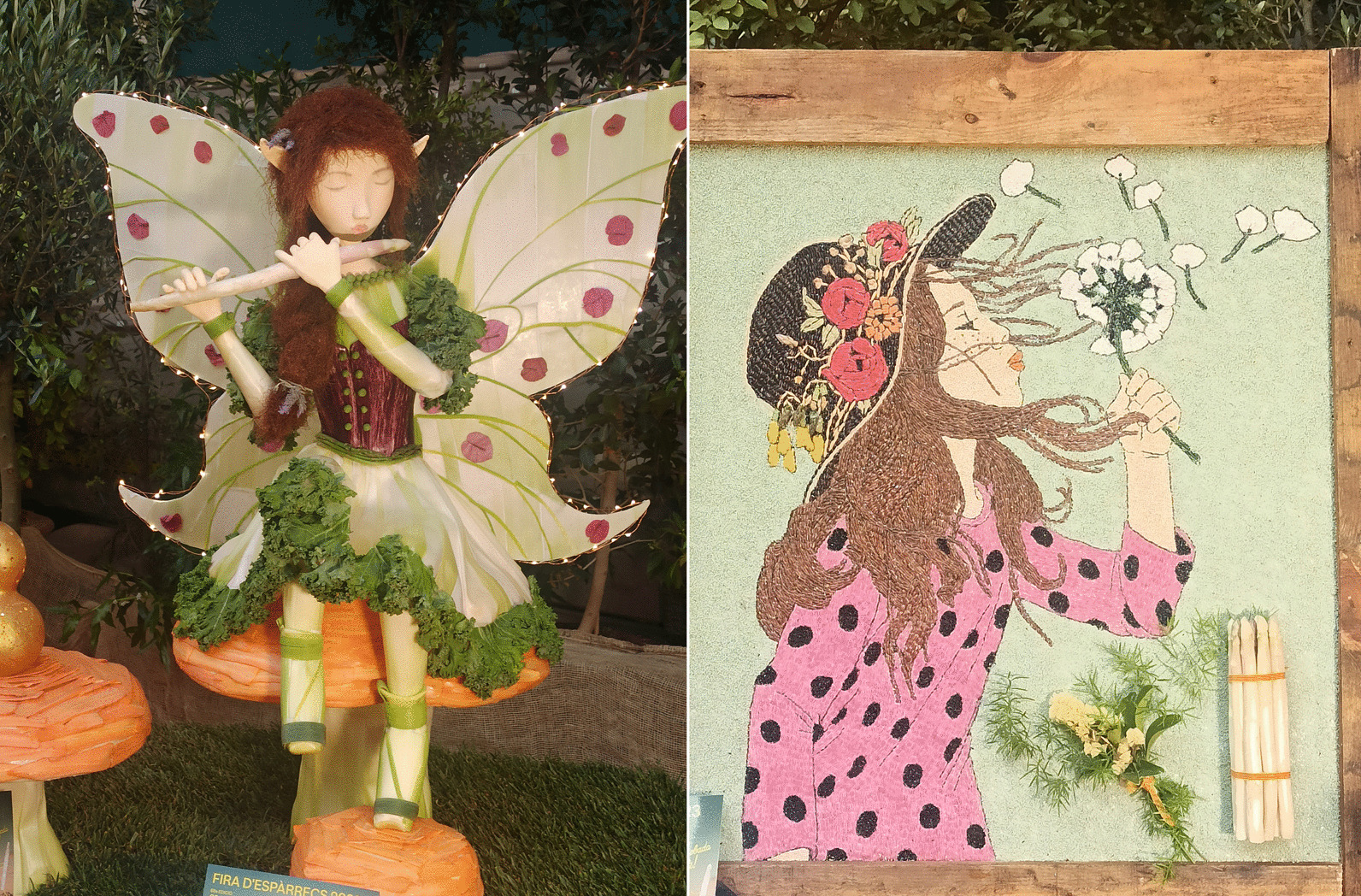
Some exhibited “paneres artístiques” at Gavà Asparagus Fair

The second most frequently mentioned category among other uses is the creation of artistic works. These mostly involve the crafting of “paneres artístiques” (“artistic baskets”) (Fig. 7), which are imaginative murals constructed from plant material. This practice is deeply rooted in local tradition and culminates in an exhibition and competition held during the Gavà Asparagus Fair, a spring event that has taken place annually since 1932. Around 20 distinct species are cited for this creative purpose.

The most used parts of plants for other uses are aerial parts (40%), followed by leaves (14%) and the living plant itself (9%).

Studying other uses is vital due to their strong cultural importance and potential economic contributions [82]. Traditional knowledge is crucial for enhancing agricultural sustainability, fostering self-sufficiency and promoting the responsible use of local resources [83]. Unlike some ethnobotanical studies, applying botany for non-food or non-medicinal purposes, particularly in agrosilvopastoral management, holds real value. Additionally, artistic uses play a key role in preserving traditions and strengthening social ties. Protecting and promoting these applications is essential for conserving natural resources and revitalizing associated knowledge [84].

### Vernacular names

In this study, 779 vernacular names have been recorded for 287 taxa, across 2295 reports. Notably, cultivated plants with distinct races, such as *Solanum lycopersicum* (95 reports, 31 folk names), *Phaseolus vulgaris* (65 reports, 27 folks names) and *Lactuca sativa* (69 reports, 15 folk names), stand out in terms of folk nomenclature. In total, 246 folk names are linked to specific races, making up 32% of the total, underscoring the importance of cultivated plants in our surveyed area. This wealth of naming diversity is common in regions with a strong agricultural presence [63]. Among non-cultivated plant species, those with fewer vernacular names are the most cited, like *Thymus vulgaris* (35 reports), *Rosmarinus officinalis* (29 reports) and *Portulaca oleracea* (28 reports).

The ethnophytonomy index for vernacular names (25%) closely resembles the traditional ethnobotanicity index, indicating that most plants have a vernacular name in Catalan. The allochthonous ethnophytonomy index is 17%, meaning around one-fifth of cited taxa have been named in a non-Catalan language (mainly Spanish, but also English), reflecting the diverse population in the area due to historical immigration.

The linguistic diversity index is 2.71. This is a high value compared to other places in the Catalan territory because many names have been found associated with the different cultivated races. If we do not count these races, the index is 1.84, a similar value to that of the periurban Gironès (1.90) [22], and the more rural—but including some small cities as well—Alt Empordà (1.94) [57] or Montseny (1.76) [64].

The linguistic diversity index is 2.71, higher than in many other parts of Catalonia. This is due to the numerous names associated with different cultivated races. Excluding these, the index becomes 1.84, similar to values in other CLA [22, 57, 64].

Lastly, it is worth noting certain unique folk names and deviations from standard names that are not covered in a comprehensive compilation of over 35,000 Catalan-language plant names for approximately 6000 taxa [85]. For instance, folk names such as “sargués” (*Rubus ulmifolius*; 5 reports), “vordolaga” (*Portulaca oleracea*; 4 reports) or “galerà” (*Ruscus aculeatus*; 3 reports) are observed. It is also notable to mention the frequent use of linguistic variations already documented in the compilation mentioned above, such as “llaurer” (*Laurus nobilis*; 17 reports), “ufals” (*Medicago sativa*; 12 reports), “àpit” (*Apium graveolens*; 4 reports) and “jonquillo” (*Narcissus tazetta;* 4 reports).

## Conclusions

This study reveals that this type of territory, located in a metropolitan area, is attractive for its scientific interest in biocultural diversity. Local ecological knowledge exists, persists and is generated in this periurban agricultural area. This is indicated by the number of taxa with names and use reports cited, as well as the values of the calculated quantitative ethnobotanical indexes.

Knowledge about medicinal uses of plants has been found among the informants. However, the decline in their use is significant, caused by acculturation or transculturation, partly due to the industrialization of medicinal and food systems [22]. At a food level, this periurban agricultural area plays a role as a reservoir of fundamental plant biodiversity, particularly at intraspecific level [25, 86]. Furthermore, in agricultural areas like this one, the relationship between medicine uses and food uses of plants becomes even closer, highlighting the possibility of a deeper exploration of folk functional foods in these type of territories.

Traditional botanical and agricultural knowledge is derived from the specific management of the local environment. As a result, several cultivated plants, plant races and land management practices have been documented, and this knowledge has been preserved and transmitted [25]. In the study area, there is a reservoir of knowledge concerning its agrosilvopastoral activities, which can be leveraged for the future management of the territory.

Hence, this study underscores the importance of ethnobotanical research in periurban agricultural regions, threatened by all kind of urban pressures. Such studies can lead to multidisciplinary initiatives for revitalization, including seed exchange programs, community gardens, cultural preservation projects and leveraging historical heritage as a tourist attraction [8, 33, 87].

### Supplementary Information

**Additional file 1**. Details about the study area.

**Additional file 2**. Table with uses reported.

**Additional file 3**. Ethnobotanical catalog, in original language.

## Data Availability

All data generated or analyzed during this study are included in this published article (and its supplementary information files).

## Abbreviations

CLA: Catalan linguistic area
EI: Ethnobotanical index
FFI: Folk functional food index
F_IC_: Informant consensus factor
MI: Index of medicinal importance
MP: Medicinal plants
RFC: Relative frequency of citation
UR: Use report
