# Supplementary material for: Traditional knowledge 10 min far from Barcelona: ethnobotanical study in the Llobregat river delta (Catalonia, NE Iberian Peninsula), a heavily anthropized agricultural area
Source: J Ethnobiol Ethnomed. 2023 Sep 26;19:41. doi: 10.1186/s13002-023-00615-2 (PMC10523798; doi:10.1186/s13002-023-00615-2)
Supplement: Supplementary file 1 — Additional file 1. Details about the study area. [file 13002_2023_615_MOESM1_ESM.docx]

**Supplementary material**

We consider the detailed investigation of the socio-economic characteristics of the examined area to be of interest, which is why we are presenting this supplementary material.

The study area, situated in the central coast of Catalonia within the NE Iberian Peninsula, encompasses the right floodplain of the Llobregat river delta. It spans 127.71 km2 and has a population of 335,759 residents, resulting in a population density of 2,629.07 individuals per km2 (Table 1). The territorial segmentation is illustrated in Figure 1.

Table 1. Municipalities, area, inhabitants, population density and proportion of inhabitants relative to the study area. L., Llobregat. Font: [1].

| **Municipality** | **Area (km^2^)** | **Inhabitants** | **Density (inhabitants/km^2^)** | **Inhabitants/area of study (%)** |
| --- | --- | --- | --- | --- |
| Castelldefels | 12.87 | 67,460 | 5,241.65 | 20.13 |
| El Prat de L. | 31.41 | 65,385 | 2,081.66 | 19.46 |
| Gavà | 30.75 | 47,057 | 1,530.31 | 14.01 |
| Sant Boi de L. | 21.47 | 84,500 | 3,935.72 | 25.16 |
| Sant Climent de L. | 10.81 | 4,160 | 384.83 | 1.24 |
| Viladecans | 20.40 | 67,197 | 3,293.97 | 20.00 |
| **TOTAL** | **127.71** | **335,759** | **2,629.07** | **100%** |

The territorial evolution requires considering high human influence. Human impact is widespread across the Llobregat delta, affecting even natural areas. The features of ponds, marshes, pine forests, and dune communities are shaped by historical human interventions [2].

**
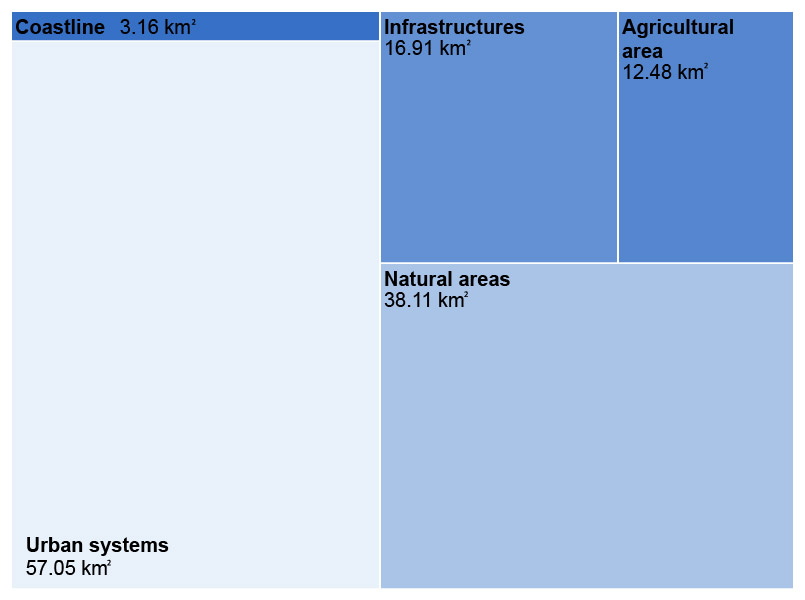
**

**Fig. 1.** Schematic representation of surface area for the different territorial elements. Font: own elaboration from [1, 3].

Historical evolution

Historically, the earliest settlements in the area date back to the 12th century, with agriculture and livestock as the primary economic activities. In the 18th century, a notable transformation of the landscape occurred, characterized by the drainage of marshlands and the subsequent alteration of the delta's original environment. The introduction of advanced water management techniques, including the construction of irrigation canals and the availability of artesian water sources, played a pivotal role in the development of highly productive irrigated farms in the latter part of the 19th century [2].

The most significant transformations in the Llobregat delta were driven by the emergence and progression of industrialization during the 20th century. A substantial wave of immigration occurred (Fig. 2), resulting in rapid population growth in municipalities adjacent to Barcelona, particularly during the 1960s-1970s. A majority of these migrants came from various Spanish regions, including Andalusia, Castile-La Mancha, Castile-León, Galicia, and Extremadura. Notably, migrants constituted 53% of the population in the 1970s and 37% in the 1990s [4]. This led to unplanned urban expansion and industrial development along the river's vicinity. Concurrently, there was significant environmental degradation of aquifers, the river, marine waters, and ponds [2, 5].

In the 1980s and 1990s, anthropogenic pressure increased exponentially due to the expansion of the Barcelona metropolitan area, which concentrated its development efforts on an area where only 16.8% of the territory was protected [6, 7]. This heightened vulnerability in the right floodplain of the Llobregat river delta, endangering both landscape resources and the associated knowledge base [8].

**
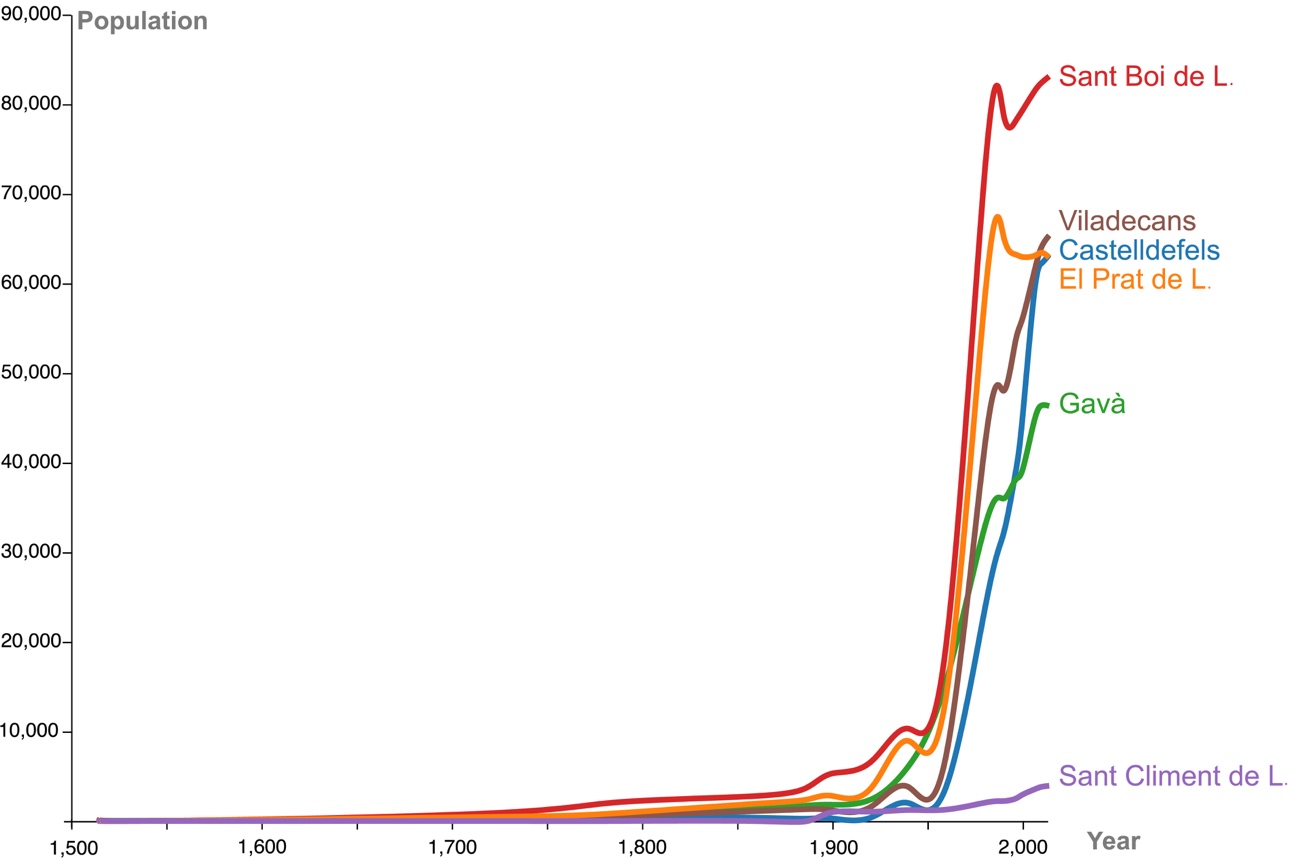
**

**Fig. 2**. Evolution of population in the municipalities studied from 1515 to 2014. L., Llobregat. Font: own elaboration from [1].

Contemporary human activity

The study area is situated within the Barcelona metropolitan region, establishing a direct connection to the capital of Catalonia, known for its vibrant economic activity (www.amb.cat). Notably, the tertiary sector dominates, employing 62% of the active population, with a primary focus on commercial services (17%) [9]. The primary sector represents a mere 1% of the population's activity, experiencing a noticeable decline (Fig. 3).


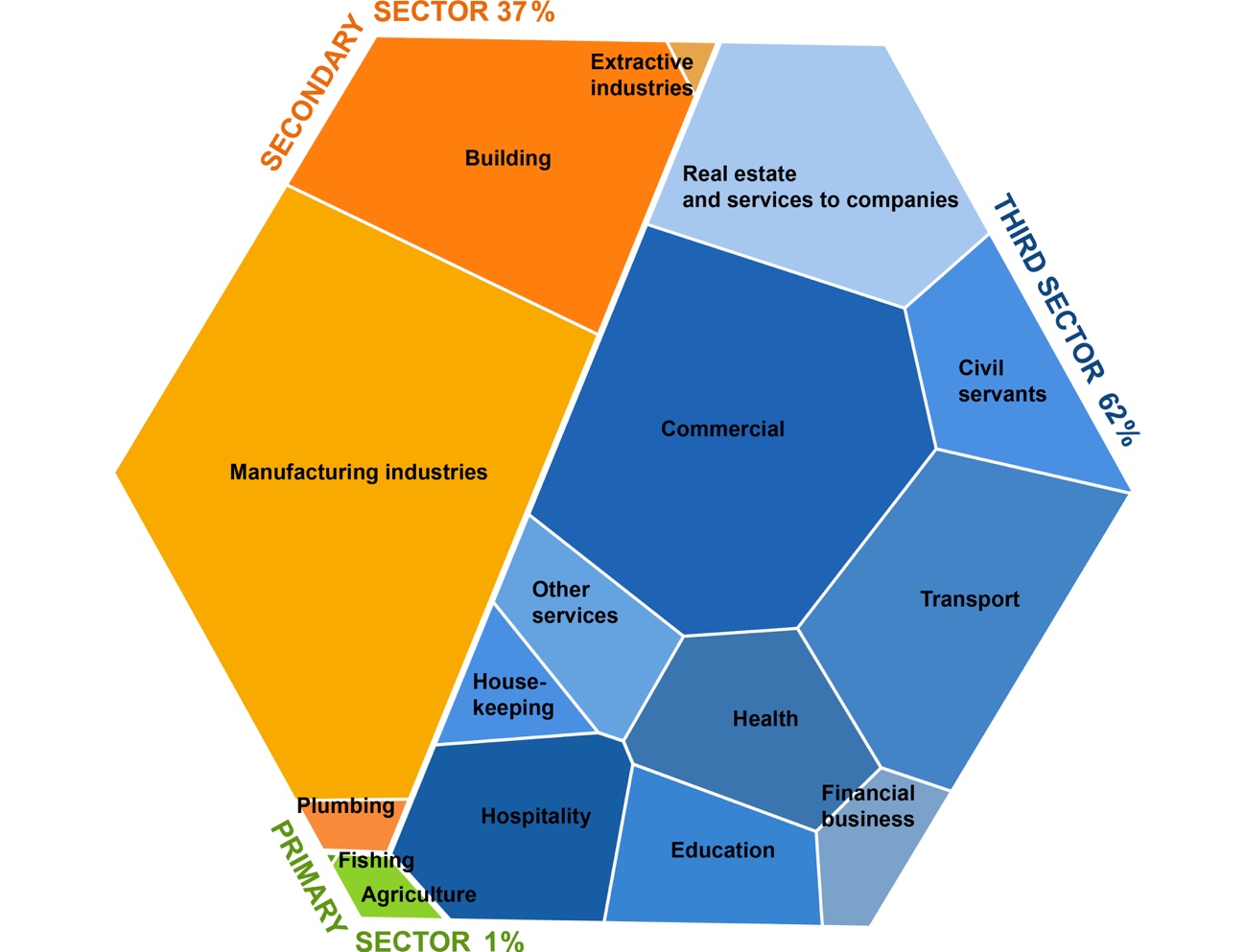


**Fig. 3.** Proportion of population (in %) regarding work activity. Font: own elaboration from [3].

Regarding employment and residents, there are more people working than there are available jobs. Notably, 44% of the local population works in the study area, while 16% both work and commute within the Baix Llobregat region. Additionally, 32% commute daily to Barcelona for work, and 8% work and commute in other areas. Conversely, workers who commute daily to the study area come from: I) other parts of the same region (20%), II) the city of Barcelona (35%), and III) other areas (1%) (Fig. 4). This underscores the significant interdependence within the broader Barcelona metropolitan area [10].


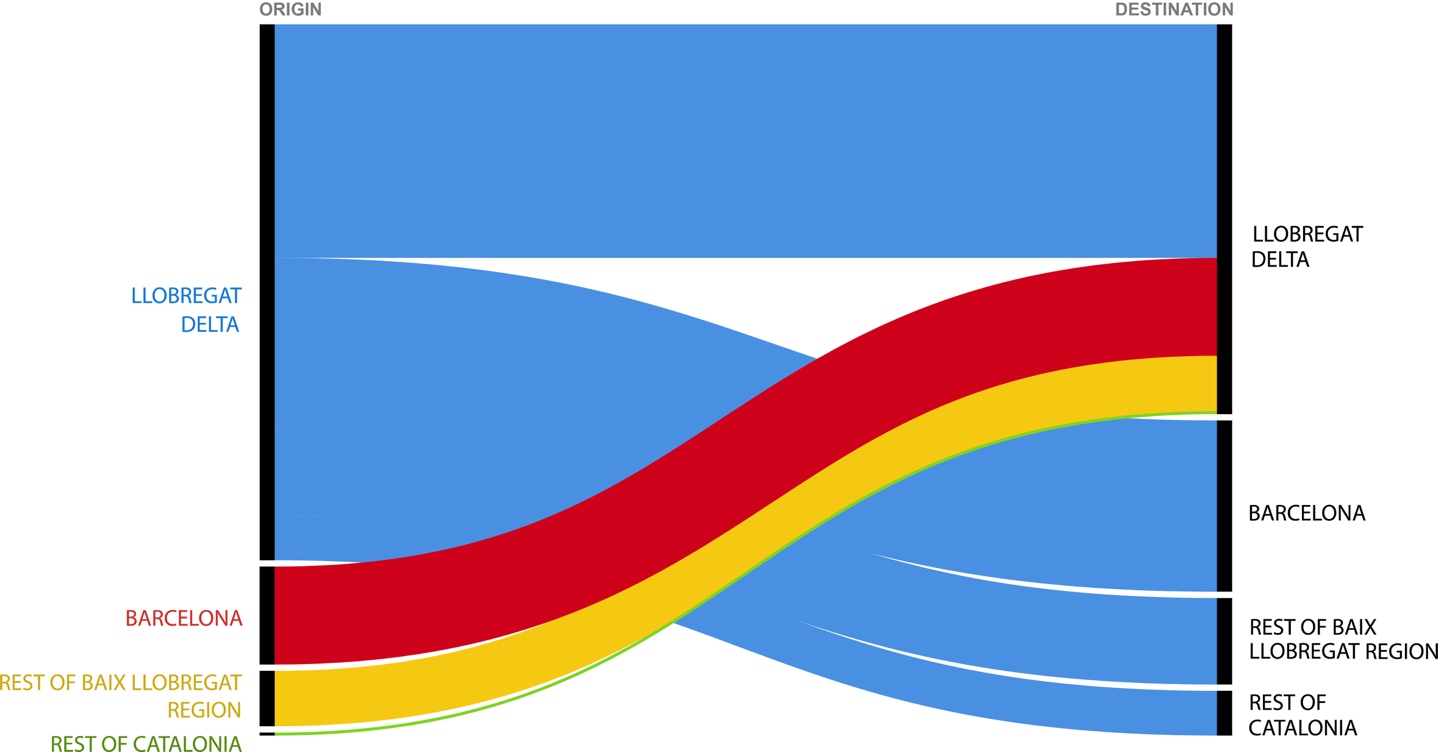


**Fig. 4.** Daily commuting for work. Relationship of the study area and the adjacent regions. Font: own elaboration from [3].

Baix Llobregat Agricultural Park

Despite its limited economic impact, agriculture plays a significant role in the study area, primarily due to the establishment of the Agricultural Park in the 1990s. The Baix Llobregat Agricultural Park, managed by a local public consortium, is dedicated to preserving and enhancing the region's natural and cultural assets (Fig. 5). Covering 3,473 hectares, the Baix Llobregat Agricultural Park primarily focuses on the cultivation of vegetables and fruit trees. It has gained recognition as a Spanish model for sustaining agricultural activities in a densely urbanized setting [8]. The study area accounts for 72% of the entire Baix Llobregat Agricultural Park (Fig. 6).

**
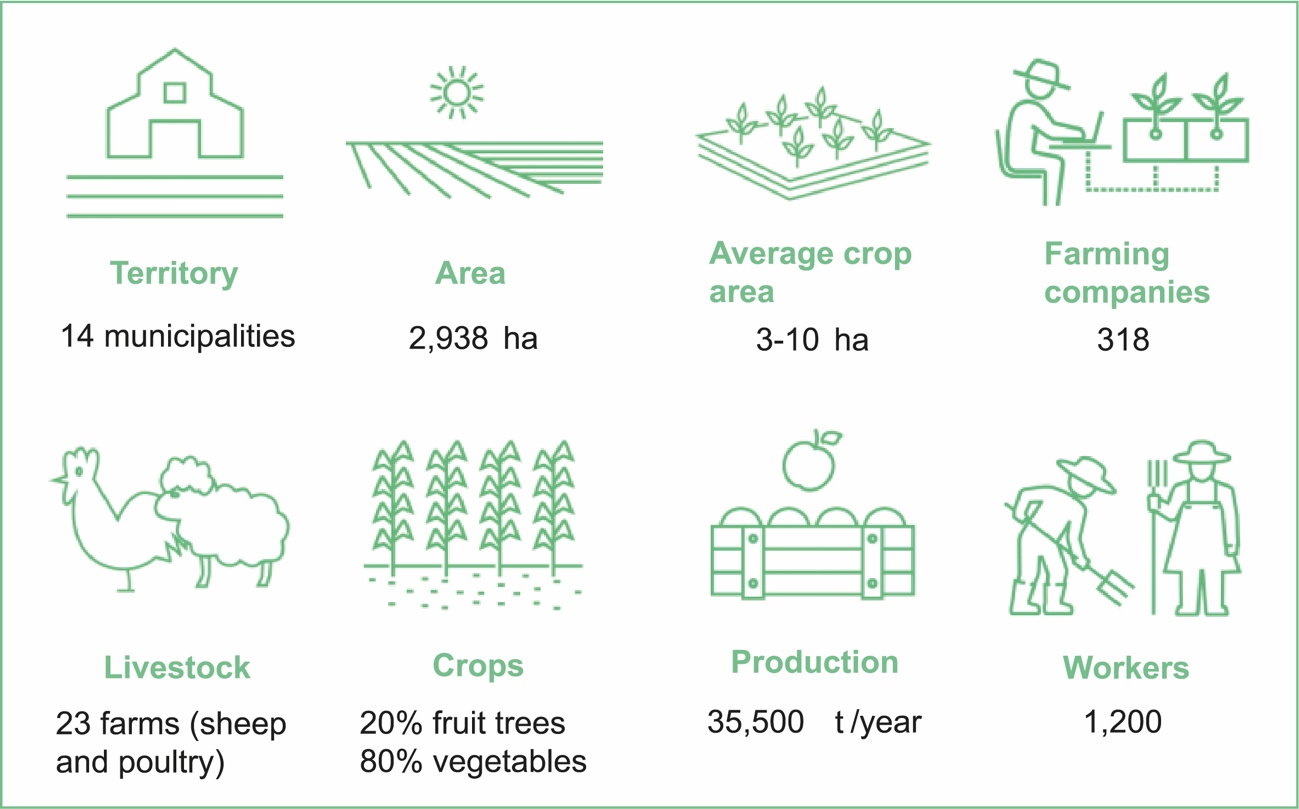
**

**Fig. 5.** Main characteristics of Baix Llobregat Agricultural Park (https://parcagrari.cat)


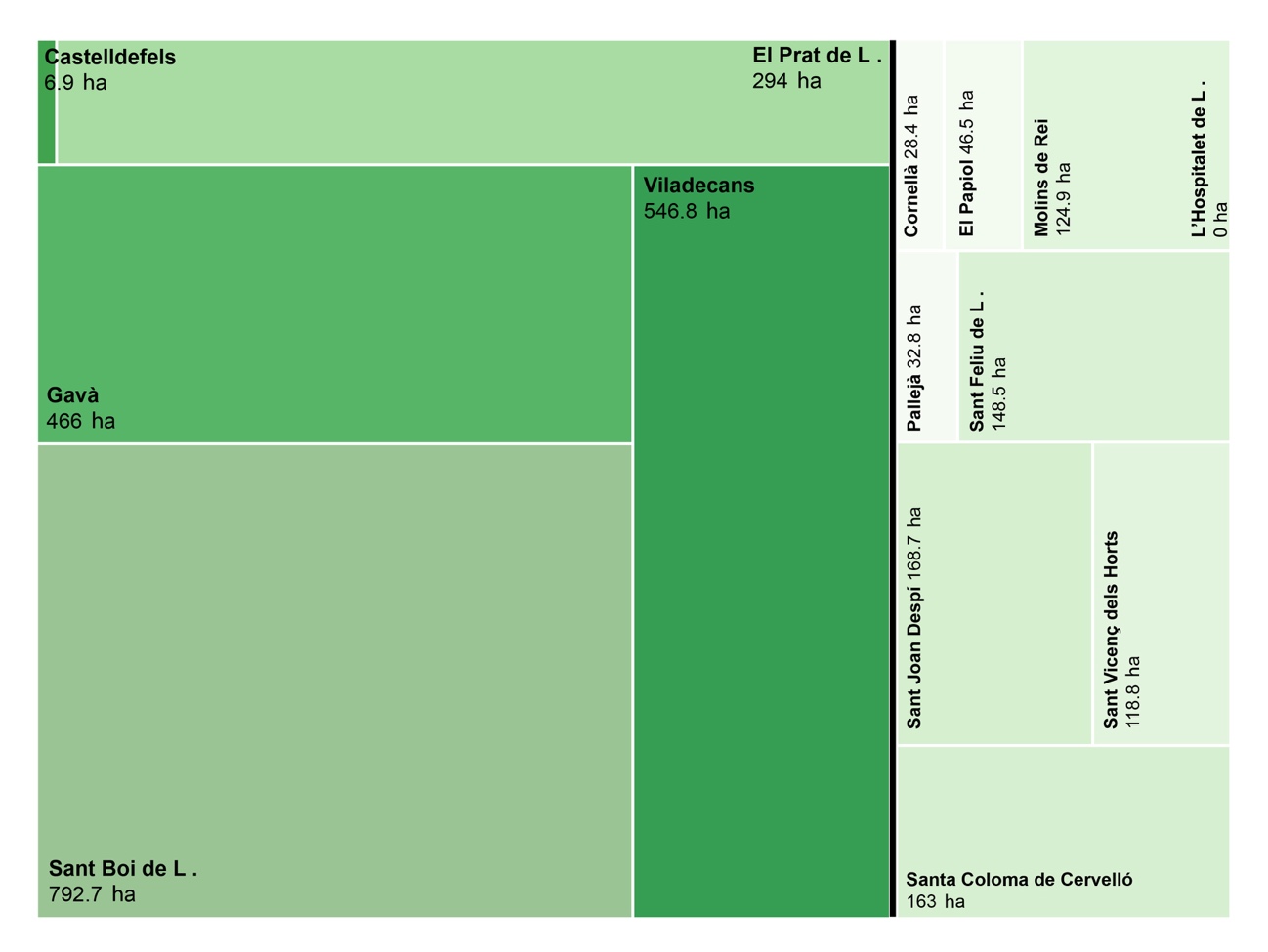
**Fig. 6.** Area of Baix Llobregat Agricultural Park. Dark-green left side corresponds to the study area municipalities. L., Llobregat. Font: own elaboration from [1].

**References**

1. Institut d’Estadística de Catalunya (IDESCAT). https://idescat.net. Accessed 21 March 2023.
2. Esteban P, Laredo S, Pino J, Valverde A. El context deltaic: situació, origen geologic i història del poblament humà. Els sistemes naturals del Delta del Llobregat. Institució Catalana d’Història Natural. Barcelona. 2018; 1:27-39.
3. Diputació de Barcelona. Hermes, informació estadística local*.* https://diba.cat/hermes. Accessed 1 February 2023.
4. Recaño, J. La immigració al Baix Llobregat. L’onada migratòria dels anys seixanta. Centre d’Estudis Demogràfics, Barcelona. 2000.
5. Bofill M. El Pas de la societat agrària a industrial al Baix Llobregat. Agricultura intensiva i industrialització. Ed. Abadia de Montserrat. Barcelona. 1995.
6. Bausa J. El Pla Delta, un riu de projectes. Revista Espais. 1994; p. 5-12.
7. Observatori del territori. Unitats de paisatge. Departament de Territori i Sostenibilitat. 2020; p. 40.
8. Zazo A, Hernández A. El parque agrario. Preservación de la actividad agraria en espacios periurbanos. El caso del Bajo Llobregat. CONAMA 10. Congreso Nacional de Medio Ambiente. Conference paper. 2010.
9. Culleré R, Canals JM. Estructura empresarial de la demarcació de Barcelona. Diputació de Barcelona. 2018.
10. Palos J. La dinámica de las periferias urbanas y su percepción. Agentes y factores de transformación del territorio. El caso del sector central del Baix Llobregat. [PhD thesis]. Universitat de Barcelona, Barcelona. 1999.
