# Supplementary material for: Traditional knowledge 10 min far from Barcelona: ethnobotanical study in the Llobregat river delta (Catalonia, NE Iberian Peninsula), a heavily anthropized agricultural area
Source: J Ethnobiol Ethnomed. 2023 Sep 26;19:41. doi: 10.1186/s13002-023-00615-2 (PMC10523798; doi:10.1186/s13002-023-00615-2)
Supplement: Supplementary file 3 — Additional file 3. Ethnobotanical catalog, in original language. [file 13002_2023_615_MOESM3_ESM.docx]

***Acacia dealbata***Link. (papilionàcies)
BCN 129006

**NOMS POPULARS**

Mimosa (2188, 2189, 2192)

**ALTRES USOS**

**Part aèria florida**

Elaboració de rams

FONT 2188, 2189, 2192. DESCRIPCIÓ DE L'ÚS FETA PER L'INFORMANT. Als anys 1950, per Rams se’n venien de 200 a 400 manats a la Rambla de Barcelona (2188). Es collien les branques florides per a vendre en manats (2189). Ornamental. Es collien les branques florides per a vendre (2192).

***Actinidia chinensis***Planch. (actinidiàcies)
BCN-E-233

**NOMS POPULARS**

Kiwi (fruit) (2194, 2199, 2200, 2206, 2223)

**USOS ALIMENTARIS**

**Fruit**

Ingestió de la part de la planta crua - Fresca (sense preparació)

FONTS 2194, 2199, 2200, 2206. DESTINACIÓ. Alimentació humana.

**ALTRES USOS**

**Epicarpi**

Elaboració d'obres artístiques

FONT 2223. DESCRIPCIÓ DE L'ÚS FETA PER L'INFORMANT. La pell del fruit serveix per a elaborar les paneres artístiques, sobretot per a fer vestimenta.

***Aesculus hippocastanum***L. (sapindàcies)
BCN 132987

**NOMS POPULARS**

Castanya borda (llavor) (2221)

**USOS MEDICINALS**

**Llavor**

Antihemorroidal

FONT 2221. FORMA FARMACÈUTICA I ÚS. Ungüent (ús extern). MODE D'UTILITZACIÓ/POSOLOGIA. Se’n feia un remei contra les morenes. Es ficaven en oli i s’untava la part dolorida amb l’ungüent. DESTINACIÓ. Medicina humana.

***Agaricus bisporus***(Lange) Imbach. (agaricàcies)
BCN-E-216

**NOMS POPULARS**

Xampinyó (2178, 2193)

**USOS ALIMENTARIS**

**Part aèria**

Ingestió de la part de la planta cuita - Cuita en oli

FONTS 2178, 2193. DESCRIPCIÓ DE L'ÚS FETA PELS INFORMANTS. Se'n menja. DESTINACIÓ. Alimentació humana.

***Aloe vera***(L.) Burm.f. (asfodelàcies)
BCN 27242

**NOMS POPULARS**

Àloe vera (2190, 2217)

**USOS MEDICINALS**

**Fulla**

Antipiròtic

FONT 2217. FORMA FARMACÈUTICA I ÚS. Sense forma farmacèutica (ús directe) (ús extern). MODE D'UTILITZACIÓ/POSOLOGIA. Tallada i aplicada superficialment. Per a les cremades. DESTINACIÓ. Medicina humana.

**Suc de la fulla**

Antipruriginós

FONT 2190. DESCRIPCIÓ DE L'ÚS FETA PER L'INFORMANT. Al gos, li posava el suc de les fulles a les orelles per a curar-li els talls i aconseguir que no es rasqués. FORMA FARMACÈUTICA I ÚS. Sense forma farmacèutica (ús directe) (ús extern). DESTINACIÓ. Medicina veterinària.

***Alyssum maritimum***(L.) Lam. (crucíferes)
BCN 129699

**USOS MEDICINALS**

**Summitat florífera**

Diürètic

FONT 2214. FORMA FARMACÈUTICA I ÚS. Infusió (ús intern). DESTINACIÓ. Medicina humana.

Protector renal

FONT 2214. FORMA FARMACÈUTICA I ÚS. Infusió (ús intern). DESTINACIÓ. Medicina humana.

***Allium cepa***L. (amaril·lidàcies)
BCN-E-214

**NOMS POPULARS**

Calçot (2160, 2178, 2188, 2193, 2194, 2199, 2200, 2202, 2206, 2212)

Ceba (2172, 2174, 2178, 2181, 2193, 2195, 2196, 2197, 2198, 2199, 2204, 2210, 3700, 2221, 2212, 2215, 2220, 2223, 2228)

Ceba bavosa (raça) (2226)

Ceba blanca (raça) (2194, 2199)

Ceba campeia (raça) (2212)

Ceba de Figueres (raça) (2199, 2200, 2203, 2205, 2226)

Ceba platillo (raça) (2212)

Ceba dolça (2201, 2203, 2205)

Ceba dolça Fuentes de Ebro (raça) (2204)

Ceba morada (raça) (2193, 2200, 2205)

Ceba sang de bou (raça) (2226)

Ceba seca (2200, 2201, 2202, 2205)

Ceba tendra (2164, 2178, 2182, 2193, 2198, 2200, 2206, 3700)

Ceballot (2221)

*Cebolleta* (castellà) (2168, 2169, 2170, 2171)

**USOS MEDICINALS**

**Bulb**

Antitussigen

FONT 2172. DESCRIPCIÓ DE L'ÚS FETA PER L'INFORMANT. Se'n fa un xarop que va bé per a la tos. FORMA FARMACÈUTICA I ÚS. Xarop (ús intern). DESTINACIÓ. Medicina humana.

Diürètic

FONT 2228. FORMA FARMACÈUTICA I ÚS. Desconegut per l'informant / No consta. DESTINACIÓ. Medicina humana.

Mucolític

FONT 2174. FORMA FARMACÈUTICA I ÚS. Desconegut per l'informant / No consta. MODE D'UTILITZACIÓ/POSOLOGIA. Es cou la ceba i es deixa en l’aigua del bull. DESTINACIÓ. Medicina humana.

Resolutiu

FONT 2204. FORMA FARMACÈUTICA I ÚS. Sinapisme (ús extern). MODE D'UTILITZACIÓ/POSOLOGIA. Contra els furóncols anava bé un tall de ceba. Abans, era freqüent veure joves amb furóncols al coll. Doncs bé, aquests es curaven simplement amb un tros de ceba. Es parteix una ceba pel mig i n’agafem un tall que faci cassoleta i, a l’interior, hi posem un trosset de sabó de rentar roba, de la mida aproximada d’una avellana. Aleshores, el posem al foc i quan el sabó s’ha desfet i la ceba estigui mig cuita, s’aplica –el més calent possible- al furóncol, com si fos una boina i s’embena fortament. A les poques hores, ha deixat anar el [dit "la"] pus i es cura aviat. DESTINACIÓ. Medicina humana.

Salutífer

FONT 2228. DESCRIPCIÓ DE L'ÚS FETA PER L'INFORMANT. Activa el drenatge de les toxines del cos, per a eliminar-les. FORMA FARMACÈUTICA I ÚS. Sense forma farmacèutica (ús directe) (ús intern). DESTINACIÓ. Medicina humana.

**USOS ALIMENTARIS**

**Bulb**

Ingestió de la part de la planta crua - Fresca (sense preparació)

FONTS 2168, 2169, 2170, 2171, 2182. CONSUMICIÓ. Amanida (2170, 2171). DESTINACIÓ. Alimentació humana (2168, 2169, 2170, 2171, 2182).

Ingestió de la part de la planta cuita - Cuita sense vehicle

FONTS 2160, 2215. DESCRIPCIÓ DE L'ÚS FETA PELS INFORMANTS. Al vapor (2215). CONSUMICIÓ. Plat principal (2160). DESTINACIÓ. Alimentació humana (2160, 2215).

Ingestió de la part de la planta cuita - Cuita en aigua

FONT 2181. DESCRIPCIÓ DE L'ÚS FETA PER L'INFORMANT. Se'n fan sopes. DESTINACIÓ. Alimentació humana.

No consta el tipus d'ingestió - No consta el mode de preparació

FONTS 2175, 2178, 2193, 2194, 2195, 2196, 2197, 2198, 2199, 2200, 2201, 2202, 2203, 2204, 2205, 2210, 2212, 2220, 2221, 2226, 3700. DESCRIPCIÓ DE L'ÚS FETA PELS INFORMANTS. Se'n menja (2221). S’enforcaven amb trenes de sègol i es penjaven del sostre (2212). Comestible (2226). DESTINACIÓ. Alimentació humana (2175, 2178, 2193, 2194, 2195, 2196, 2197, 2198, 2199, 2200, 2201, 2202, 2203, 2204, 2205, 2210, 2212, 2220, 2221, 2226, 3700).

**Planta sencera**

Ingestió de la part de la planta cuita - Cuita en aigua

FONT 2228. DESCRIPCIÓ DE L'ÚS FETA PER L'INFORMANT. Per a fer caldo. DESTINACIÓ. Alimentació humana.

**Tija amb fulles/branques**

Ingestió de la part de la planta crua - Fresca (sense preparació)

FONTS 2164, 3700. CONSUMICIÓ. Amanida. DESTINACIÓ. Alimentació humana.

Ingestió de la part de la planta cuita - Cuita sense vehicle

FONT 2188. DESCRIPCIÓ DE L'ÚS FETA PER L'INFORMANT. Els calçots es mengen. DESTINACIÓ. Alimentació humana.

No consta el tipus d'ingestió - No consta el mode de preparació

FONTS 2200, 2203, 2206. DESTINACIÓ. Alimentació humana.

**ALTRES USOS**

**Bulb**

Elaboració d'obres artístiques

FONT 2223. DESCRIPCIÓ DE L'ÚS FETA PER L'INFORMANT. Les pells de la ceba [catafil·les] serveixen per a elaborar les paneres artístiques.

**BARREGES AMB AQUEST TÀXON (vegeu catàleg de barreges)**

**USOS ALIMENTARIS**

**Planta sencera**

FONT 2228. Caldo depuratiu.

***Allium porrum***L. (amaril·lidàcies)
BCN 129003

**NOMS POPULARS**

Porro (2164, 2178, 2193, 2196, 2198, 2199, 2200, 2202, 2205, 2212, 2225, 2223, 2218, 2227)

*Puerro* (castellà) (3933)

**USOS ALIMENTARIS**

**Part aèria**

No consta el tipus d'ingestió - No consta el mode de preparació

FONT 2227. DESCRIPCIÓ DE L'ÚS FETA PER L'INFORMANT. Se'n menja. DESTINACIÓ. Alimentació humana.

Ingestió de la part de la planta cuita - Cuita en greix

FONT 2164. DESTINACIÓ. Alimentació humana.

No consta el tipus d'ingestió - No consta el mode de preparació

FONTS 2178, 2193, 2196, 2198, 2199, 2200, 2202, 2205, 2218, 3933. DESCRIPCIÓ DE L'ÚS FETA PELS INFORMANTS. Comestible (2218). DESTINACIÓ. Alimentació humana (2178, 2193, 2196, 2198, 2199, 2200, 2202, 2205, 2218, 3933).

**ALTRES USOS**

**Bulb**

Elaboració d'obres artístiques

FONTS 2223, 2225. DESCRIPCIÓ DE L'ÚS FETA PELS INFORMANTS. Les catafil·les [dit "fulles"] serveixen per a elaborar les paneres artístiques (2225). Les primeres fulles [catafil·les] són les que es fan servir per a fer les paneres artístiques. Es netegen, s’estenen per tal que s’assequin una mica i llavors és quan es poden manipular. Es tallen i se’ls dona forma (2223).

***Allium sativum***L. (amaril·lidàcies)
BCN-E-295

**NOMS POPULARS**

All (2182, 2190, 2199, 2200, 2204, 3700, 2221, 2212, 3935, 2225, 2223, 2228)

All de Lleida (raça) (2212)

All morisc (raça) (2212)

All sec (2184, 2201, 2202)

All tendre (2165, 2167, 2178, 2193, 2194, 2196, 2197, 2198, 2200, 2201, 2202, 2203)

**USOS MEDICINALS**

**Bulb**

Antisèptic intern

FONT 3935. FORMA FARMACÈUTICA I ÚS. Sense forma farmacèutica (ús directe) (ús intern). DESTINACIÓ. Medicina humana.

Diürètic

FONT 3935. FORMA FARMACÈUTICA I ÚS. Sense forma farmacèutica (ús directe) (ús intern). DESTINACIÓ. Medicina humana.

Per als penellons

FONT 2204. FORMA FARMACÈUTICA I ÚS. Sense forma farmacèutica (ús directe) (ús extern). MODE D'UTILITZACIÓ/POSOLOGIA. Per a curar els penellons als dits o a les orelles es fregava un gra d’all tallat directament al penelló. DESTINACIÓ. Medicina humana.

Vasotònic

FONT 2228. DESCRIPCIÓ DE L'ÚS FETA PER L'INFORMANT. Activa la circulació. FORMA FARMACÈUTICA I ÚS. Desconegut per l'informant / No consta. DESTINACIÓ. Medicina humana.

**USOS ALIMENTARIS**

**Bulb**

Condiment

FONT 2184. DESCRIPCIÓ DE L'ÚS FETA PER L'INFORMANT. Adobat d'olives. DESTINACIÓ. Alimentació humana.

No consta el tipus d'ingestió - No consta el mode de preparació

FONTS 2182, 2190, 2194, 2199, 2200, 2201, 2202, 2212, 2221, 3700, 3935. DESCRIPCIÓ DE L'ÚS FETA PELS INFORMANTS. Comestible (2221). S’enforcaven amb trenes de sègol i es penjaven del sostre (2212). Potencia el gust de la carn, el peix i les verdures (3935). DESTINACIÓ. Alimentació humana (2182, 2190, 2194, 2199, 2200, 2201, 2202, 2212, 2221, 3700, 3935).

**Fulla**

Ingestió de la part de la planta crua - Fresca (sense preparació)

FONT 2165. DESCRIPCIÓ DE L'ÚS FETA PER L'INFORMANT. Es van tallant les fulles joves per a posar-les sobre amanides. Deixa un gust picant. CONSUMICIÓ. Amanida. DESTINACIÓ. Alimentació humana.

**Tija**

Ingestió de la part de la planta cuita - Cuita en oli

FONTS 2167, 2202. CONSUMICIÓ. Truita (2167, 2202). DESTINACIÓ. Alimentació humana (2167, 2202).

No consta el tipus d'ingestió - No consta el mode de preparació

FONTS 2178, 2193, 2196, 2197, 2198, 2200, 2201, 2203. DESTINACIÓ. Alimentació humana.

**ALTRES USOS**

**Bulb**

Elaboració d'obres artístiques

FONTS 2223, 2225. DESCRIPCIÓ DE L'ÚS FETA PELS INFORMANTS. L'epidermis del bulb [dit "pell de la cabeça d’alls"] serveix per a elaborar les paneres artístiques (2225). Les pells de la cabeça d’all serveixen per a elaborar les paneres artístiques. Sobretot, per a fer coses petites, com la vestimenta. Es van tallant les pells i es van folrant les mànigues, la faldilla… (2223).

Creences i pràctiques magicoreligioses

FONT 3935. DESCRIPCIÓ DE L'ÚS FETA PER L'INFORMANT. Si es penja del marc de la porta, foragita els mals esperits.

**BARREGES AMB AQUEST TÀXON (vegeu catàleg de barreges)**

**USOS ALIMENTARIS**

**Bulb**

FONT 2192. Conserva de tomàquet.

FONT 2192. Olives arreglades.

***Allium schoenoprasum***L. (amaril·lidàcies)
BCN 129686

**NOMS POPULARS**

Cebollí (3935)

*Cebollino* (castellà) (2194)

**USOS MEDICINALS**

**Fulla**

Diürètic

FONT 3935. FORMA FARMACÈUTICA I ÚS. Sense forma farmacèutica (ús directe) (ús intern). DESTINACIÓ. Medicina humana.

Litotríptic renal

FONT 3935. FORMA FARMACÈUTICA I ÚS. Desconegut per l'informant / No consta. DESTINACIÓ. Medicina humana.

**USOS ALIMENTARIS**

**Fulla**

Ingestió de la part de la planta crua - Fresca (sense preparació)

FONTS 2194, 3935. DESCRIPCIÓ DE L'ÚS FETA PER L'INFORMANT. Per a amanir plats (2194). El cebollí tallat ben petit va bé per a les amanides, els formatges tous, els ous, les patates i les salses. És aconsellable posar-ho a darrera hora, ja que conservarà més el sabor (3935). DESTINACIÓ. Alimentació humana (2194, 3935).

**ALTRES USOS**

**Planta viva *ex situ***

Creences i pràctiques magicoreligioses

FONT 3935. DESCRIPCIÓ DE L'ÚS FETA PER L'INFORMANT. És un bon purificador i protector de la casa.

**Planta viva *in situ***

Agrosilvopastoral

FONT 3935. DESCRIPCIÓ DE L'ÚS FETA PER L'INFORMANT. Plantat a la vora de les pastanagues, repel·leix les mosques. OBSERVACIONS. Associació de cultius i equilibri sistèmic.

***Amaranthus retroflexus***L. (amarantàcies)
BCN 126572

**NOMS POPULARS**

Blet (2161, 2219, 3934)

**USOS ALIMENTARIS**

**Fulla**

Ingestió de la part de la planta cuita - Cuita en aigua

FONTS 2219, 3934. DESCRIPCIÓ DE L'ÚS FETA PER L'INFORMANT. Es poden menjar les fulles com una verdura (2219). Abans se'n menjava bullida (3934). DESTINACIÓ. Alimentació humana (3934, 2219).

***Ampelodesmos mauritanica***(Poiret) T.Durand et Schinz (gramínies)
BCN 130953

**NOMS POPULARS**

Càrritx (2213)

Escarç (2192)

Mauritana (2214)

**ALTRES USOS**

**Fulla**

Agrosilvopastoral

FONT 2192. DESCRIPCIÓ DE L'ÚS FETA PER L'INFORMANT. Per a fer lligalls. OBSERVACIONS. Elaboració d'estris d'ús hortícola/agrícola.

Elaboració de cordes i sogues

FONT 2214. DESCRIPCIÓ DE L'ÚS FETA PER L'INFORMANT. S’ha fet servir per a fer cordes i trenats per a cistells.

**Planta viva *in situ***

Agrosilvopastoral

FONT 2213. DESCRIPCIÓ DE L'ÚS FETA PER L'INFORMANT. És una planta que ajuda que no es degradin els talussos. OBSERVACIONS. Elements paisatgístics.

***Anagallis arvensis***L. (primulàcies)
BCN 130959

**NOMS POPULARS**

Gallinassa (2184)

***Ananas comosus***(Stickm.) Merr. (bromeliàcies)
BCN-E-262

**NOMS POPULARS**

Pinya (infructescència) (2200, 2216)

**USOS ALIMENTARIS**

**Infructescència**

Ingestió de la part de la planta crua - Fresca (sense preparació)

FONTS 2200, 2216. DESCRIPCIÓ DE L'ÚS FETA PELS INFORMANTS. Se'n menja. Abans era només per Nadal. Ara n’hi ha sempre (2216). DESTINACIÓ. Alimentació humana (2200, 2216).

***Anethum graveolens***L. (umbel·líferes)
BCN 126575

**NOMS POPULARS**

*Eneldo* (castellà) (2163, 2165)

Fonoll pudent (3935)

**USOS MEDICINALS**

**Fruit**

Cardiotònic

FONT 3935. FORMA FARMACÈUTICA I ÚS. Infusió (ús intern). DESTINACIÓ. Medicina humana.

Digestiu

FONT 3935. FORMA FARMACÈUTICA I ÚS. Infusió (ús intern). DESTINACIÓ. Medicina humana.

Per a trastorns de la pell o del teixit subcutani

FONT 3935. DESCRIPCIÓ DE L'ÚS FETA PER L'INFORMANT. Es prepara una infusió de llavors picolades per a enfortir les ungles. FORMA FARMACÈUTICA I ÚS. Infusió (ús extern). DESTINACIÓ. Medicina humana.

**USOS ALIMENTARIS**

**Fruit**

Condiment

FONTS 2165, 3935. DESCRIPCIÓ DE L'ÚS FETA PER L'INFORMANT. Sobre carns i peixos. Es fa servir també en pastissos (2165). En plats d’ous, carn i peix (3935). CONSUMICIÓ. Postres (2165). DESTINACIÓ. Alimentació humana (2165, 3935).

**Fulla jove**

Condiment

FONTS 2163, 3935. DESCRIPCIÓ DE L'ÚS FETA PER L'INFORMANT. Per al peix (2163). En plats d’ous, carn i peix (3935). DESTINACIÓ. Alimentació humana (2163, 3935).

**Inflorescència**

Ingestió de la part de la planta crua - Fresca (sense preparació)

FONT 3935. DESCRIPCIÓ DE L'ÚS FETA PER L'INFORMANT. En plats d’ous, carn i peix. DESTINACIÓ. Alimentació humana.

**ALTRES USOS**

**Tija amb fulles/branques**

Creences i pràctiques magicoreligioses

FONT 3935. DESCRIPCIÓ DE L'ÚS FETA PER L'INFORMANT. Posat a prop de les figures dels sants, ajuda a trobar objectes perduts.

***Annona cherimola***Mill. (annonàcies)
BCN-E 303

**NOMS POPULARS**

Xirimoia (fruit) (2200, 2201, 2206)

**USOS ALIMENTARIS**

**Fruit**

Ingestió de la part de la planta crua - Fresca (sense preparació)

FONTS 2200, 2201, 2206. DESTINACIÓ. Alimentació humana.

***Anthriscus cerefolium***(L.) Hoffm. (umbel·líferes)
BCN 127874

**NOMS POPULARS**

Cerfull (3935)

*Perifollo* (castellà) (2194)

**USOS MEDICINALS**

**Fulla**

Per a trastorns de la pell o del teixit subcutani

FONT 3935. FORMA FARMACÈUTICA I ÚS. Loció (ús extern). PREPARACIÓ. Una màscara de fulles de cerfull neteja la pell. DESTINACIÓ. Medicina humana.

**USOS ALIMENTARIS**

**Fulla**

Condiment

FONTS 2194, 3935. DESCRIPCIÓ DE L'ÚS FETA PER L'INFORMANT. Condiment per a tot tipus de menjar (3935). DESTINACIÓ. Alimentació humana (2194, 3935).

***Apium graveolens***L. var. ***dulce*** (Mill.) Pers. (umbel·líferes)
BCN 130965

**NOMS POPULARS**

Api (2170, 2171, 2173, 2174, 2178, 2194, 2196, 2197, 2198, 2199, 2200, 2201, 2205, 2218)

Àpit (2193, 2202, 2227, 2228)

**USOS MEDICINALS**

**Fulla**

Diürètic

FONTS 2173, 2174. FORMA FARMACÈUTICA I ÚS. Sense forma farmacèutica (ús directe) (ús intern). DESTINACIÓ. Medicina humana (2173, 2174).

Hematocatàrtic

FONT 2228. DESCRIPCIÓ DE L'ÚS FETA PER L'INFORMANT. Depuratiu. FORMA FARMACÈUTICA I ÚS. Sense forma farmacèutica (ús directe) (ús intern). DESTINACIÓ. Medicina humana.

**USOS ALIMENTARIS**

**Fulla**

Ingestió de la part de la planta cuita - Cuita en aigua

FONTS 2170, 2171, 2197, 2228. DESCRIPCIÓ DE L'ÚS FETA PELS INFORMANTS. Per al caldo (2170, 2171, 2197). Per a fer caldo (2228). DESTINACIÓ. Alimentació humana (2170, 2171, 2197, 2228).

No consta el tipus d'ingestió - No consta el mode de preparació

FONTS 2178, 2200, 2201, 2202. DESTINACIÓ. Alimentació humana (2178, 2200, 2201, 2202).

**Part aèria**

Ingestió de la part de la planta cuita - Cuita en aigua

FONTS 2194, 2199, 2205, 2218. DESCRIPCIÓ DE L'ÚS FETA PER L'INFORMANT. Per al caldo (2194, 2205). DESTINACIÓ. Alimentació humana (2194, 2199, 2205, 2218).

No consta el tipus d'ingestió - No consta el mode de preparació

FONTS 2193, 2196, 2198, 2227. DESCRIPCIÓ DE L'ÚS FETA PELS INFORMANTS. Comestible (2227). DESTINACIÓ. Alimentació humana (2193, 2196, 2198, 2227).

**BARREGES AMB AQUEST TÀXON (vegeu catàleg de barreges)**

**USOS ALIMENTARIS**

**Fulla**

FONT 2228. Caldo depuratiu.

***Apium graveolens***L. var. ***rapaceum***(Mill.) DC. (umbel·líferes)
BCN 46859

**NOMS POPULARS**

Api-nap (2196)

**USOS ALIMENTARIS**

**Arrel**

Ingestió de la part de la planta cuita - Cuita en aigua

FONT 2196. DESCRIPCIÓ DE L'ÚS FETA PER L'INFORMANT. Se'n menja. DESTINACIÓ. Alimentació humana.

***Arachis hypogaea***L. (papilionàcies)
BCN-E-449

**NOMS POPULARS**

Cacauet (2221)

**USOS ALIMENTARIS**

**Llavor**

Ingestió de la part de la planta crua - Conservada dessecada a l'aire

FONT 2221. DESCRIPCIÓ DE L'ÚS FETA PER L'INFORMANT. Se'n menja sec. DESTINACIÓ. Alimentació humana.

***Araujia sericifera***Brot. (apocinàcies)
BCN 129696

**NOMS POPULARS**

Miraguà (2213)

*Miraguano* (castellà) (2219, 2232)

**ALTRES USOS**

**Llavor**

Farciments tèxtils

FONTS 2213, 2219, 2232. DESCRIPCIÓ DE L'ÚS FETA PELS INFORMANTS. El plomall de les granes s’ha fet servir per a omplir coixins (2213). Per a omplir coixins (2232).

***Artemisia annua***L. (compostes)
BCN 130945

**NOMS POPULARS**

Artemisa (2219)

**USOS MEDICINALS**

**Part aèria**

Antipirètic (per a febre periòdica)

FONT 2219. DESCRIPCIÓ DE L'ÚS FETA PER L'INFORMANT. És utilitzada contra la febre. FORMA FARMACÈUTICA I ÚS. Desconegut per l'informant / No consta. DESTINACIÓ. Medicina humana.

Antiprotozoari

FONT 2219. DESCRIPCIÓ DE L'ÚS FETA PER L'INFORMANT. És utilitzada contra la malària. FORMA FARMACÈUTICA I ÚS. Desconegut per l'informant / No consta. DESTINACIÓ. Medicina humana.

***Artemisia arborescens***L. (compostes)
BCN 126569

**NOMS POPULARS**

*Té moruno* (castellà) (2163)

**USOS ALIMENTARIS**

**Fulla**

Preparació de begudes - Beguda preparada amb aigua

FONTS 2163, 2190. DESCRIPCIÓ DE L'ÚS FETA PER L'INFORMANT. Conreada per un veí marroquí. La fa servir per al te (2163). Els àrabs la bullen i la fan servir per al te (2190). DESTINACIÓ. Alimentació humana.

***Artemisia dracunculus***L. (compostes)
BCN 13328

**NOMS POPULARS**

Dragonet (3935)

Estragó (3935)

**USOS MEDICINALS**

**Fulla**

Digestiu

FONT 3935. FORMA FARMACÈUTICA I ÚS. Sense forma farmacèutica (ús directe) (ús intern). PREPARACIÓ. Com a condiment en el menjar. DESTINACIÓ. Medicina humana. FONT 3935. FORMA FARMACÈUTICA I ÚS. Infusió (ús intern). DESTINACIÓ. Medicina humana.

**USOS ALIMENTARIS**

**Fulla**

Condiment

FONT 3935. DESCRIPCIÓ DE L'ÚS FETA PER L'INFORMANT. Com a condiment en el menjar. DESTINACIÓ. Alimentació humana.

***Arundo donax***L. (gramínies)
BCN 129009

**NOMS POPULARS**

Canya (2160, 2162, 2164, 2167, 2179, 2186, 2188, 2190, 2191, 2204, 2210, 2212, 2214, 2218, 2226, 3700, 3934, 3936)

Canya americana (2219)

**USOS MEDICINALS**

**Tija defoliada**

Antiàlgic lumbar

FONT 2204. FORMA FARMACÈUTICA I ÚS. Sense forma farmacèutica (ús directe) (ús extern). MODE D'UTILITZACIÓ/POSOLOGIA. Hi havia qui curava la lumbàlgia amb aquest procediment: calia agafar dues canyes, d’un metre i mig o dos de llargada i aixafar-les amb els peus i, aleshores, partides pel mig i de llarg a llarg, l’esllomat les agafava per la punta i les estrenyia, una a cada costat de la cintura. S’havia de repetir el cerimonial uns quants dies fins que l’esllomat guaria. DESTINACIÓ. Medicina humana.

**ALTRES USOS**

**Fulla**

Jocs i joguines

FONT 2204. DESCRIPCIÓ DE L'ÚS FETA PER L'INFORMANT. Se'n feien unes flautetes que es deien trompetes.

**Planta viva *in situ***

Agrosilvopastoral

FONT 2179. DESCRIPCIÓ DE L'ÚS FETA PER L'INFORMANT. La van fer servir per a aguantar els talussos de les rieres, però és molt invasiva i s'ha estès per tot arreu. OBSERVACIONS. Elements paisatgístics.

**Tija**

Agrosilvopastoral

FONTS 2160, 2162, 2164, 2167, 2179, 2186, 2188, 2191, 2204, 2210, 2218, 2226, 3700, 3934, 3936. DESCRIPCIÓ DE L'ÚS FETA PELS INFORMANTS. Les assequen i les fan servir per a lligar tomaqueres i mongetes del ganxet (2160). S'assequen i les fan servir per a lligar tomaqueres i mongetes. Si es tallen en lluna vella duren més temps (2162). Per a encanyar tomaqueres (2164). Les fan servir per a enramar mongetes (2167). Per a emparrar tomàquets i mongeteres (2179). Per a fer enfilar tomaqueres i mongeteres (2186). S’obre la punta amb un ganivet i al mig es posa una pedra. S’utilitza per a agafar préssecs i figues (2188). Per a fer enfilar tomaqueres (2188). Per a fer enfilar les mongetes. Usa 14.000 canyes per cada hectàrea de mongetes. Les canyes es posen de quatre en quatre, fent una mena de piràmide, i es lliguen per dalt. D’això se’n diu “pollera”. Les canyes es llossen (se n’afila la punta) i es claven al terra, per tal que no caiguin si bufa el vent (2191). Per a fer enfilar mongeteres i tomaqueres (2204). Per a fer enfilar tomaqueres (2210). Per a fer enramar les tomaqueres (3934, 3936). Per a fer enfilar tomàquets, mongetes, pebrots i pèsols (2218). Marcaven amb una canya les coliflors i els bròquils grans que volien deixar granar per a l’any següent (2218). Per a fer enfilar les mongeteres (2226). Per a lligar tomaqueres i mongeteres (3700). OBSERVACIONS. Elaboració d'estris d'ús hortícola/agrícola (2160, 2162, 2164, 2167, 2179, 2186, 2188, 2191, 2198, 2204, 2210, 2218, 2226, 3700, 3936).

Elaboració d'estris de cuina

FONT 2212. DESCRIPCIÓ DE L'ÚS FETA PER L'INFORMANT. Es feia el tub o canut de la sal. Amb un tros de canya gruixuda d’uns 20 centímetres, al qual es deixava una anella per a fer de fons, i es tancava amb un tap de suro.

Obtenció de combustible: llenya

FONTS 2167, 2179, 2218. DESCRIPCIÓ DE L'ÚS FETA PELS INFORMANTS. Es tallen i es guarden per a encendre el foc i fer flama. Per a fer calçots van molt bé (2167). La canya que ja havia servit per a emparrar tomàquets i mongeteres es cremava per a escalfar la llar (2179). La canya que es trencava o no servia, la utilitzaven com a llenya per a encendre el foc (2218).

Obtenció de materials per a la construcció

FONTS 2179, 2214. DESCRIPCIÓ DE L'ÚS FETA PELS INFORMANTS. Tot l'Eixample de Barcelona estava construït amb cel ras, fet de guix i canya. La canya, la trencaven per tal que allargués més i la ficaven en guix (2179). Per a fer sostres i parets (2214).

**ALTRES OBSERVACIONS**

FONT 3700. DESCRIPCIÓ FETA PER L'INFORMANT. Calia pagar un cop l’any els propietaris del canal de la Dreta per a fer-les servir. Les canyes es tallaven després de Tots Sants.

***Asparagus acutifolius***L. (asparagàcies)
BCN 126582

**NOMS POPULARS**

Esparreguera (2190)

Espàrrec (turió) (2190, 2192, 2204, 2210, 2212)

Espàrrec de marge (turió) (2165)

**USOS MEDICINALS**

**Turió**

Protector renal

FONT 2210. FORMA FARMACÈUTICA I ÚS. Desconegut per l'informant / No consta. DESTINACIÓ. Medicina humana.

**USOS ALIMENTARIS**

**Turió**

Ingestió de la part de la planta crua - Fresca (sense preparació)

FONT 2165. DESCRIPCIÓ DE L'ÚS FETA PER L'INFORMANT. Brots crus. CONSUMICIÓ. Aperitiu. DESTINACIÓ. Alimentació humana.

Ingestió de la part de la planta cuita - Cuita en oli

FONTS 2165, 2190, 2204, 2212. CONSUMICIÓ. Truita. DESTINACIÓ. Alimentació humana.

***Asparagus officinalis***L. (asparagàcies)
BCN 126563

**NOMS POPULARS**

Espàrrec (turió) (2164, 2167, 2200, 2201, 2210, 2221, 2212, 3933, 3939, 3946, 2225, 2218)

Esparreguera (2210, 2225)

Falluc (part aèria) (2212)

Mota (rizoma) (2212)

Perico (turió gruixut) (2212)

**USOS MEDICINALS**

**Turió**

Protector renal

FONT 2210. FORMA FARMACÈUTICA I ÚS. Sense forma farmacèutica (ús directe) (ús intern). PREPARACIÓ. Menjats bullits. DESTINACIÓ. Medicina humana.

**USOS ALIMENTARIS**

**Turió**

Ingestió de la part de la planta cuita - Cuita en aigua

FONTS 2164, 2167. DESCRIPCIÓ DE L'ÚS FETA PELS INFORMANTS. Al febrer la planta s'asseca, s'arregla la terra i tornen a brotar. Si els vols blancs, s'han de tapar. Les plantes, però, han d'estar els tres o quatre primers anys sense tocar-les (2167). DESTINACIÓ. Alimentació humana (2164, 2167).

Ingestió de la part de la planta cuita - Cuita en oli

FONTS 3933, 3939. CONSUMICIÓ. Truita (3933, 3939). DESTINACIÓ. Alimentació humana (3933, 3939).

No consta el tipus d'ingestió - No consta el mode de preparació

FONTS 2200, 2201, 2212, 2218, 2221, 3946. DESCRIPCIÓ DE L'ÚS FETA PELS INFORMANTS. Comestible (2218). DESTINACIÓ. Alimentació humana (2200, 2201, 2212, 2218, 2221, 3946).

**ALTRES USOS**

**Fulla**

Elaboració d'obres artístiques

FONT 2225. DESCRIPCIÓ DE L'ÚS FETA PER L'INFORMANT. Per a elaborar les paneres artístiques.

**Turió**

Elaboració d'obres artístiques

FONT 2225. DESCRIPCIÓ DE L'ÚS FETA PER L'INFORMANT. Per a elaborar les paneres artístiques. Dels espàrrecs, en fan làmines molt fines amb el pelador.

**ALTRES OBSERVACIONS**

FONT 2210. DESCRIPCIÓ FETA PER L'INFORMANT. Quan es van començar a plantar aquí, es feia una mota rodona d’esparregueres calçada amb les sorres, però era dificultós. Més tard, començaren a fer rengles amb mates alternades.

***Asphodelus fistulosus***L. (asfodelàcies)
BCN 130949

**NOMS POPULARS**

*Gamonita* (castellà) (2213)

**ALTRES OBSERVACIONS**

FONT 2213. DESCRIPCIÓ FETA PER L'INFORMANT. És indicador que ve el bon temps.

***Atriplex halimus***L. (amarantàcies)
BCN 130944

**ALTRES USOS**

**Tija amb fulles/branques**

Elaboració d'escombres

FONT 2160.

***Atropa belladonna***L. (solanàcies)
BCN 19513

**NOMS POPULARS**

Belladona (2204)

**USOS MEDICINALS**

**No consta**

Antiparotidític

FONT 2204. FORMA FARMACÈUTICA I ÚS. Ungüent (ús extern). MODE D'UTILITZACIÓ/POSOLOGIA. Quan es tenien galteres, el mal alleugeria quan s’untava el coll i a sota de la barbeta amb un ungüent de belladona, aplicat amb un drap que es lligava al voltant del coll. DESTINACIÓ. Medicina humana.

Pediculicida

FONT 2204. FORMA FARMACÈUTICA I ÚS. Ungüent (ús extern). MODE D'UTILITZACIÓ/POSOLOGIA. Per a matar els polls del cap, els apotecaris feien un ungüent amb belladona. S’untava directament les parts infectades. Al cap d’algunes hores, es rentaven les parts tractades i es passava una pinta espessa per tal de treure els insectes morts o les llémenes aferrades als cabells. DESTINACIÓ. Medicina humana.

***Avena sativa***L. (gramínies)
BCN 130943

**NOMS POPULARS**

Civada (2204, 2212, 2217, 3947)

Civada Albacete (raça) (2204)

Civada Aurora 2 (raça) (2204)

Civada Blancaneus (raça) (2204)

Civada Kondor (raça) (2204)

Civada Nina (raça) (2204)

Civada Saia 6 (raça) (2204)

**USOS ALIMENTARIS**

**Trituració del gra**

No consta el tipus d'ingestió - No consta el mode de preparació

FONTS 2212, 2217, 3947. DESCRIPCIÓ DE L'ÚS FETA PELS INFORMANTS. Se’n cultivava pel gra. Conreu ja desaparegut en aquestes contrades (2212). En donaven als porcs (2217). Se'n menja (3947). DESTINACIÓ. Alimentació humana (2212, 3947). Alimentació animal (2217).

***Avena sterilis***L. (gramínies)
BCN 130963

**NOMS POPULARS**

Civada borda (2210)

**ALTRES USOS**

**Inflorescència**

Jocs i joguines

FONTS 2210, 2219. DESCRIPCIÓ DE L'ÚS FETA PER L'INFORMANT. L’espiga s’enganxa a la roba. El nombre d’espigues que s’enganxaven era el nombre de xicotes que tenies (2210). De petits, es tiraven les espigues i s’enganxaven a la roba. El nombre d’espigues que s’enganxaven era el nombre de xicotes que tenies (2219).

***Beta vulgaris***L. subsp. ***vulgaris*** var. ***vulgaris***(amarantàcies)
BCN 132999

**NOMS POPULARS**

*Acelga* (castellà) (2173, 2174)

Bleda (2161, 2164, 2178, 2180, 2186, 2190, 2193, 2194, 2195, 2196, 2197, 2198, 2199, 2200, 2201, 2202, 2204, 2205, 3700, 2221, 2219, 2227)

Bleda de colors (2164)

**USOS MEDICINALS**

**Fulla**

Laxant

FONT 3700. FORMA FARMACÈUTICA I ÚS. Sense forma farmacèutica (ús directe) (ús intern). MODE D'UTILITZACIÓ/POSOLOGIA. Menjada bullida, amb altres verdures. DESTINACIÓ. Medicina humana.

Per a trastorns del sistema digestiu

FONTS 2173, 2174, 3700. DESCRIPCIÓ DE L'ÚS FETA PELS INFORMANTS. Neteja l'intestí (2173, 2174). FORMA FARMACÈUTICA I ÚS. Sense forma farmacèutica (ús directe) (ús intern) (2173, 3700) PREPARACIÓ. Menjada bullida (2173, 3700). FORMA FARMACÈUTICA I ÚS. Decocció (ús intern) (2174). PREPARACIÓ. Es fa un caldo amb l’aigua de bullir (2174). MODE D'UTILITZACIÓ/POSOLOGIA. Té propietats digestives. Menjada bullida, amb altres verdures (3700). DESTINACIÓ. Medicina humana.

**USOS ALIMENTARIS**

**Fulla**

Ingestió de la part de la planta cuita - Cuita en aigua

FONTS 2161, 2164, 2173, 2174, 2180, 2193, 2195, 2197, 2221, 3700. DESCRIPCIÓ DE L'ÚS FETA PELS INFORMANTS. Es mengen bullides (2221). CONSUMICIÓ. Bullit (2161, 2164, 2180, 2221). DESTINACIÓ. Alimentació humana (2161, 2164, 2173, 2174, 2180, 2193, 2195, 2197, 2221, 3700).

No consta el tipus d'ingestió - No consta el mode de preparació

FONTS 2178, 2186, 2190, 2194, 2200, 2201, 2202, 2219, 2227. DESCRIPCIÓ DE L'ÚS FETA PELS INFORMANTS. Comestible (2227). DESTINACIÓ. Alimentació humana (2178, 2186, 2190, 2194, 2200, 2201, 2202, 2205, 2219, 2227).

**Fulla jove**

Ingestió de la part de la planta crua - Fresca (sense preparació)

FONT 2164. CONSUMICIÓ. Amanida. DESTINACIÓ. Alimentació humana.

**Part aèria**

No consta el tipus d'ingestió - No consta el mode de preparació

FONTS 2196, 2198, 2199. DESTINACIÓ. Alimentació humana.

**ALTRES OBSERVACIONS**

FONT 3700. DESCRIPCIÓ FETA PER L'INFORMANT. Quan es cull la bleda, es deixa un brot de la planta, perquè al cap de 25 dies torna a sortir.

***Beta vulgaris***L. subsp. ***maritima***(L.) Arcang. (amarantàcies)
BCN 130939

**NOMS POPULARS**

Bleda boscana (2182, 2204, 2216, 2218, 3934)

**USOS ALIMENTARIS**

**Fulla**

Ingestió de la part de la planta cuita - Cuita en aigua

FONTS 2182, 2204, 2218, 3934. DESCRIPCIÓ DE L'ÚS FETA PELS INFORMANTS. Se'n trobaven en els marges dels camins. Se'n mengen bullides. De sabor similar als espinacs. Ho cuinaven barrejat amb patates (2182). Se’n menja (2218). Abans se'n menjava bullida (3934). DESTINACIÓ. Alimentació humana (2182, 2204, 2218, 3934).

**Part aèria**

No consta el tipus d'ingestió - No consta el mode de preparació

FONT 2216. DESCRIPCIÓ DE L'ÚS FETA PER L'INFORMANT. Se'n menja, és molt bona, millor que els espinacs. DESTINACIÓ. Alimentació humana.

***Beta vulgaris***L. subsp. ***vulgaris*** var. ***conditiva*** Alef.  (amarantàcies)
BCN-E-198

**NOMS POPULARS**

Remolatxa (2164, 2178, 2196, 2198, 2204, 2208, 2215, 3936)

**USOS MEDICINALS**

**Arrel**

Antianèmic

FONT 2215. FORMA FARMACÈUTICA I ÚS. Suc aquós fluid ensucrat (ús intern). PREPARACIÓ. Se'n fa suc. DESTINACIÓ. Medicina humana.

**USOS ALIMENTARIS**

**Arrel**

Ingestió de la part de la planta crua - Fresca (sense preparació)

FONTS 2164, 2178, 2208. CONSUMICIÓ. Amanida (2164, 2208). DESTINACIÓ. Alimentació humana (2164, 2178, 2208).

No consta el tipus d'ingestió - No consta el mode de preparació

FONTS 2196, 2198, 3936. DESTINACIÓ. Alimentació humana.

**Fulla**

Ingestió de la part de la planta cuita - Cuita en aigua

FONT 2215. DESTINACIÓ. Alimentació humana.

Preparació de begudes - Beguda preparada amb aigua

FONT 2215. DESCRIPCIÓ DE L'ÚS FETA PER L'INFORMANT. En batuts. DESTINACIÓ. Alimentació humana.

**Part aèria**

No consta el tipus d'ingestió - No consta el mode de preparació

FONT 2196. DESTINACIÓ. Alimentació humana.

***Beta vulgaris***L. subsp. ***vulgaris*** var. ***crassa***(Alef.) Helm (amarantàcies)
BCN 50761

**NOMS POPULARS**

Remolatxa (3700, 2217)

Remolatxa sucrera (2184)

Sucre (producte elaborat) (2184, 2192, 3700, 2211)

**USOS ALIMENTARIS**

**Arrel**

Ingestió de la part de la planta crua - Conservada dessecada a l'aire

FONTS 2184, 2192, 3700. DESCRIPCIÓ DE L'ÚS FETA PELS INFORMANTS. La polpa era la resta de la remolatxa sucrera un cop se n'havia extret el sucre. Això es tenia sec en sacs. Quan calia, es posava en remull i es barrejava amb el pinso dels porcs (2184). Per a la producció de sucre i extracció d’alcohol (3700). L’arrel servia per a alimentar el bestiar (3700). DESTINACIÓ. Alimentació animal (2184, 3700). Alimentació humana (2192, 3700).

**Planta sencera**

Ingestió de la part de la planta crua - Fresca (sense preparació)

FONT 2217. DESCRIPCIÓ DE L'ÚS FETA PER L'INFORMANT. En donaven als porcs i als cavalls. DESTINACIÓ. Alimentació animal.

**BARREGES AMB AQUEST TÀXON (vegeu catàleg de barreges)**

**USOS ALIMENTARIS**

**Arrel**

FONT 2192, 3700. Arrop.

FONT 2211. Licor de llet.

***Bidens aurea***(Ait.) Sherff (compostes)

**NOMS POPULARS**

Te verd (2167, 2190)

**USOS MEDICINALS**

**Fulla**

Tranquil·litzant

FONT 2190. FORMA FARMACÈUTICA I ÚS. Infusió (ús intern). MODE D'UTILITZACIÓ/POSOLOGIA. Es bullen en aigua unes quantes fulles, seques o no. DESTINACIÓ. Medicina humana.

**USOS ALIMENTARIS**

**Part aèria florida**

Preparació de begudes - Beguda preparada amb aigua

FONT 2167. DESCRIPCIÓ DE L'ÚS FETA PER L'INFORMANT. Se'n cull en flor, que és quan està en el seu punt òptim. Se'n pren en infusió. DESTINACIÓ. Alimentació humana.

**BARREGES AMB AQUEST TÀXON (vegeu catàleg de barreges)**

**USOS MEDICINALS**

**Part aèria florida**

FONT 2190. Per al refredat.

***Borago officinalis***L. (boraginàcies)
BCN 129710

**NOMS POPULARS**

Borraina (2204, 2208, 2214, 2215, 3952)

Borratja (2187, 2190, 2192, 2202, 2210, 3935, 2228)

Penca (pecíol) (2215)

**USOS MEDICINALS**

**Fulla**

Antiinflamatori

FONT 3952. DESCRIPCIÓ DE L'ÚS FETA PER L'INFORMANT. Desinflama la pell i les mucoses. FORMA FARMACÈUTICA I ÚS. Cataplasma (ús extern). PREPARACIÓ. Escaldar les fulles i fer un cataplasma. DESTINACIÓ. Medicina humana.

Diürètic

FONTS 3935, 3952. FORMA FARMACÈUTICA I ÚS. Decocció (ús intern) (3935). Sense forma farmacèutica (ús directe) (ús intern) (3952). DESCRIPCIÓ DE L'ÚS FETA PER L'INFORMANT. Augmenta la producció d'orina i l’eliminació d'urea (3952). DESTINACIÓ. Medicina humana (3935, 3952).

Expectorant

FONT 3935. FORMA FARMACÈUTICA I ÚS. Decocció (ús intern). DESTINACIÓ. Medicina humana.

Hematocatàrtic

FONT 3935. DESCRIPCIÓ DE L'ÚS FETA PER L'INFORMANT. És depuratiu. FORMA FARMACÈUTICA I ÚS. Decocció (ús intern).

**No consta**

Antipirètic

FONT 3952. DESCRIPCIÓ DE L'ÚS FETA PER L'INFORMANT. Molt útil en malalties infeccioses i febrils com el xarampió i la varicel·la. FORMA FARMACÈUTICA I ÚS. Desconegut per l'informant / No consta. DESTINACIÓ. Medicina humana.

Expectorant

FONT 3952. DESCRIPCIÓ DE L'ÚS FETA PER L'INFORMANT. Té acció expectorant, indicada en bronquitis, grips i refredats. FORMA FARMACÈUTICA I ÚS. Desconegut per l'informant / No consta. DESTINACIÓ. Medicina humana.

**Summitat florífera**

Diaforètic

FONT 3952. DESCRIPCIÓ DE L'ÚS FETA PER L'INFORMANT. Sudorífica. Afavoreix la producció de la suor, eliminant impureses i residus que circulen per la sang. FORMA FARMACÈUTICA I ÚS. Infusió (ús intern). DESTINACIÓ. Medicina humana.

**USOS ALIMENTARIS**

**Flor**

Ingestió de la part de la planta crua - Fresca (sense preparació)

FONT 2228, 3935. DESCRIPCIÓ DE L'ÚS FETA PER L'INFORMANT. En amanida (2228). CONSUMICIÓ. Amanida (2228). DESCRIPCIÓ DE L'ÚS FETA PER L'INFORMANT. Es poden posar les flors als pastissos com a decoració. (3935). DESTINACIÓ. Alimentació humana (2228, 3935).

Preparació de begudes - Beguda preparada amb licor

FONT 3935. DESCRIPCIÓ DE L'ÚS FETA PER L'INFORMANT. Es poden posar les flors als còctels. DESTINACIÓ. Alimentació humana.

**Fulla**

Ingestió de la part de la planta cuita - Cuita en aigua

FONTS 2204, 2210, 2215, 2228, 3952. DESCRIPCIÓ DE L'ÚS FETA PELS INFORMANTS. Per a menjar. Es preparen com si fossin bledes, bullides. (2210). El nervi central gruixut [dit “penca”], bullit (2215). Es cultiva a l'Aragó com a verdura. Es bullen les fulles tendres (3952). Com a verdura (2228). CONSUMICIÓ. Bullit (2210). DESTINACIÓ. Alimentació humana (2204, 2210, 2215, 2228, 3952).

Ingestió de la part de la planta cuita - Cuita en oli

FONTS 2190, 2192, 2210, 2215, 3952. DESCRIPCIÓ DE L'ÚS FETA PELS INFORMANTS. Per a fer bunyols dolços (amb sucre, un cop tretes les punxes i bullides) o salats (només amb farina, un cop tretes les punxes i bullides) (2190). Posar en remull, enfarinar i fregir. (2192). Arrebossades (2210). La fulla, arrebossada amb farina i aigua i mel per sobre (2215). Se'n poden fer bunyols de les fulles arrebossades amb ou (3952). DESTINACIÓ. Alimentació humana (2190, 2192, 2210, 2215, 3952).

No consta el tipus d'ingestió - No consta el mode de preparació

FONT 2214. DESTINACIÓ. Alimentació humana.

**Part aèria**

Ingestió de la part de la planta cuita - Cuita en aigua

FONT 2208. DESTINACIÓ. Alimentació humana.

No consta el tipus d'ingestió - No consta el mode de preparació

FONT 2202. DESTINACIÓ. Alimentació humana.

**Tija**

No consta el tipus d'ingestió - No consta el mode de preparació

FONT 2187. DESTINACIÓ. Alimentació humana.

**ALTRES USOS**

**Flor**

Elaboració de bijuteria

FONT 3935. DESCRIPCIÓ DE L'ÚS FETA PER L'INFORMANT. Abans se’n feien collarets de flors.

***Brassica juncea***(L.) Czern. (crucíferes)
BCN 127887

**NOMS POPULARS**

Mostassa (2195)

**USOS ALIMENTARIS**

**Fulla**

Ingestió de la part de la planta crua - Fresca (sense preparació)

FONT 2195. DESTINACIÓ. Alimentació humana.

***Brassica napus***L. (crucíferes)
BCN 127892

**NOMS POPULARS**

Nap (2160, 2178, 2182, 2193, 2194, 2196, 2197, 2200, 2206, 3700, 2212, 2225, 2227)

Nap blanc (raça) (2221)

Nap negre (raça) (2221)

**USOS ALIMENTARIS**

**Arrel**

No consta el tipus d'ingestió - No consta el mode de preparació

FONTS 2178, 2182, 2193, 2194, 2196, 2197, 2200, 2206, 2212, 2221, 2227, 3700. DESCRIPCIÓ DE L'ÚS FETA PELS INFORMANTS. L’arrel servia per a alimentar el bestiar (3700). Comestible (2227). DESTINACIÓ. Alimentació humana (2178, 2182, 2193, 2194, 2196, 2197, 2200, 2206, 2212, 2221, 2227). Alimentació animal (3700).

**ALTRES USOS**

**Arrel**

Elaboració d'obres artístiques

FONT 2225. DESCRIPCIÓ DE L'ÚS FETA PER L'INFORMANT. L'epidermis de l'arrel serveix per a elaborar les paneres artístiques.

**Planta sencera**

Agrosilvopastoral

FONT 2160. DESCRIPCIÓ DE L'ÚS FETA PER L'INFORMANT. Triturada, es deixa sobre la terra i es rega. El gas que es desprèn desinfecta el sòl. OBSERVACIONS. Plaguicides naturals.

**BARREGES AMB AQUEST TÀXON (vegeu catàleg de barreges)**

**USOS ALIMENTARIS**

**Arrel**

FONT 2212. Peus de porc amb naps

***Brassica oleracea***L. subsp. ***oleracea*** var. ***botrytis***L. (crucíferes)
BCN-E-209

**NOMS POPULARS**

Bròcoli (2228)

Bròquil (2164, 2194, 2198, 2205, 2210, 3700, 2221, 2218, 2227)

Bròquil bord (raça) (2226)

Bròquil de Sant Isidre (raça) (3700)

Bròquil lila (raça) (2193)

Bròquil morat (raça) (2178, 2198, 2202)

Bròquil negre (raça) (2226)

Bròquil verd (raça) (2193, 2198, 2203)

Coliflor (2160, 2164, 2178, 2190, 2193, 2195, 2196, 2199, 2201, 2202, 2203, 2205, 2221, 2220, 2223, 2218, 2227)

Coliflor Cheddar (raça) (2195)

Coliflor lila (raça) (2196)

Coliflor verda (raça) (2178, 2196, 2199, 2201)

Romanesco (2228)

**USOS MEDICINALS**

**Inflorescència**

Carminatiu

FONT 2228. DESCRIPCIÓ DE L'ÚS FETA PER L'INFORMANT. Antiflatulent. FORMA FARMACÈUTICA I ÚS. Sense forma farmacèutica (ús directe) (ús intern). PREPARACIÓ. Bullida. DESTINACIÓ. Medicina humana.

**USOS ALIMENTARIS**

**Inflorescència**

Ingestió de la part de la planta cuita - Cuita en aigua

FONTS 2160, 2164, 2178, 2194, 2221, 3700. CONSUMICIÓ. Bullit (2160, 2164). DESTINACIÓ. Alimentació humana (2160, 2164, 2178, 2194, 2221, 3700).

No consta el tipus d'ingestió - No consta el mode de preparació

FONTS 2190, 2193, 2195, 2196, 2198, 2199, 2201, 2202, 2203, 2205, 2210, 2218, 2220, 2226, 2227. DESCRIPCIÓ DE L'ÚS FETA PELS INFORMANTS. Comestible (2218, 2227). El bròquil negre era molt dolç. Es tracta d'una raça desapareguda. El bròquil bord era una verdura que estava entre el bròquil i la coliflor (2226). DESTINACIÓ. Alimentació humana (2190, 2193, 2195, 2196, 2198, 2199, 2201, 2202, 2203, 2205, 2210, 2218, 2220, 2226, 2227).

**Part aèria**

Ingestió de la part de la planta cuita - Cuita en aigua

FONT 3700. DESCRIPCIÓ DE L'ÚS FETA PER L'INFORMANT. Es preparava un bullit amb restes d’aliments i es donava de menjar als porcs. DESTINACIÓ. Alimentació animal.

**ALTRES USOS**

**Inflorescència**

Elaboració d'obres artístiques

FONT 2223. DESCRIPCIÓ DE L'ÚS FETA PER L'INFORMANT. Els trossos de coliflor serveixen per a fer ovelles.

**Planta sencera**

Literatura oral popular: llegendes, gloses, contes, dites, refranys, poemes, cançons

FONT 2191. DESCRIPCIÓ DE L'ÚS FETA PER L'INFORMANT. "Com ha canviat el bròquil!" (dita popular que fa referència al pas del temps).

**ALTRES OBSERVACIONS**

FONT 3700. DESCRIPCIÓ FETA PER L'INFORMANT. Un camp que havia estat plantat amb coliflors quedava molt enriquit per a altres conreus, perquè les arrels desprenen molt nitrogen.

***Brassica oleracea***L. subsp. ***oleracea*** var. ***capitata***L. f. ***capitata*** (crucíferes)
BCN 130930

**NOMS POPULARS**

Col (2160, 2164, 2180, 2190, 2191, 2193, 2194, 2195, 2196, 2197, 2199, 2200, 2202, 2203, 2205, 2206, 2208, 2210, 3700, 2221, 2220, 2226, 2228)

Col arrissada (raça) (2201, 2202)

Col d'espigall (2167)

Col d'olla (raça) (2227)

Col de brotons (raça) (2193)

Col de cabdell (raça) (2178)

Col de Milà (raça) (2198, 2204)

Col de paperina (raça) (2178, 2198, 2202, 2204, 3700, 2227)

Col francesa (raça) (2204)

Col híbrida (raça) (3700)

Col holandesa (raça) (2204)

Col kale (raça) (2178, 2195, 2196)

Col llombarda (raça) (2205, 2227)

Col setsetmanera (raça) (2204)

Col valenciana (raça) (2204)

Col verda (raça) (2204, 2226)

Col verda d'espigall (2165)

Espigall (raça) (2206, 2227, 2226)

Llombarda (2215)

**USOS MEDICINALS**

**Fulla**

Carminatiu

FONT 2228. DESCRIPCIÓ DE L'ÚS FETA PER L'INFORMANT. Antiflatulent. FORMA FARMACÈUTICA I ÚS. Sense forma farmacèutica (ús directe) (ús intern). PREPARACIÓ. Bullida. DESTINACIÓ. Medicina humana.

Hipolipemiant

FONT 2208. FORMA FARMACÈUTICA I ÚS. Sense forma farmacèutica (ús directe) (ús intern). MODE D'UTILITZACIÓ/POSOLOGIA. Bullida, és bona per a mitigar el colesterol. DESTINACIÓ. Medicina humana.

Per a prevenir el càncer

FONT 2208. FORMA FARMACÈUTICA I ÚS. Sense forma farmacèutica (ús directe) (ús intern). MODE D'UTILITZACIÓ/POSOLOGIA. [La col] bullida. DESTINACIÓ. Medicina humana.

Protector renal

FONT 2226. DESCRIPCIÓ DE L'ÚS FETA PER L'INFORMANT. La fulla s’escalfava i es posava sobre els ronyons, per a guarir el seu mal. FORMA FARMACÈUTICA I ÚS. Apòsit medicamentós (ús extern). DESTINACIÓ. Medicina humana.

**Suc de la fulla**

Antianèmic

FONT 2215. FORMA FARMACÈUTICA I ÚS. Sense forma farmacèutica (ús directe) (ús intern). PREPARACIÓ. Se'n fa suc. DESTINACIÓ. Medicina humana.

**USOS ALIMENTARIS**

**Fulla**

Ingestió de la part de la planta cuita - Cuita sense vehicle

FONT 2215. DESCRIPCIÓ DE L'ÚS FETA PER L'INFORMANT. Al vapor. DESTINACIÓ. Alimentació humana.

Ingestió de la part de la planta cuita - Cuita en aigua

FONTS 2160, 2164, 2167, 2178, 2180, 2194, 2197, 2199, 2210, 2221, 2227, 3700. DESCRIPCIÓ DE L'ÚS FETA PELS INFORMANTS. Se'n menja bullida (2221). [La col d'espigall] és una col antiga que se'n menja bullida (2167). Se'n menja (2227). CONSUMICIÓ. Bullida (2160, 2164, 2178, 3700). DESTINACIÓ. Alimentació humana (2160, 2164, 2167, 2178, 2180, 2194, 2197, 2199, 2210, 2221, 2227, 3700).

Ingestió de la part de la planta cuita - Cuita en aigua i greix

FONTS 2165, 2178. DESCRIPCIÓ DE L'ÚS FETA PER L'INFORMANT. [La col verda d'espigall] és la millor que hi ha per a fer el trinxat (2165). [La col de paperina] es fa servir per al trinxat (2178). DESTINACIÓ. Alimentació humana (2165, 2178).

No consta el tipus d'ingestió - No consta el mode de preparació

FONTS 2190, 2191, 2193, 2195, 2196, 2198, 2200, 2201, 2202, 2203, 2205, 2220. DESTINACIÓ. Alimentació humana.

**Gemma de la fulla**

No consta el tipus d'ingestió - No consta el mode de preparació

FONT 2206. DESCRIPCIÓ DE L'ÚS FETA PER L'INFORMANT. Els espigalls són utilitzats pels clients gallecs com a verdura. DESTINACIÓ. Alimentació humana.

**Inflorescència**

Ingestió de la part de la planta cuita - Cuita en aigua

FONT 2208. DESTINACIÓ. Alimentació humana.

**ALTRES USOS**

**Fulla**

Recol·lecció per a la venda

FONT 2180. DESCRIPCIÓ DE L'ÚS FETA PER L'INFORMANT. Venen les fulles als pescaters per a posar-hi el peix a sobre.

**Part aèria**

Agrosilvopastoral

FONT 2160. DESCRIPCIÓ DE L'ÚS FETA PER L'INFORMANT. Triturada, es deixa sobre la terra i es rega. El gas que se'n desprèn desinfecta la terra. OBSERVACIONS. Element per a l'agricultura biològica.

***Brassica oleracea***L. subsp. ***oleracea*** var. ***gemmifera***DC. (crucíferes)
BCN-E-247

**NOMS POPULARS**

Col de Brussel·les (2178, 2193, 2199, 2200, 2205)

**USOS ALIMENTARIS**

**Gemma de la fulla**

Ingestió de la part de la planta cuita - Cuita en aigua

FONTS 2178, 2193. DESCRIPCIÓ DE L'ÚS FETA PELS INFORMANTS. Bullides (2193). DESTINACIÓ. Alimentació humana (2178, 2193).

No consta el tipus d'ingestió - No consta el mode de preparació

FONTS 2194, 2196, 2199, 2200, 2205. DESTINACIÓ. Alimentació humana.

***Brassica oleracea***L. subsp. ***oleracea*** var. ***italica***Plenck (crucíferes)
BCN-E-208

**NOMS POPULARS**

Bròcoli (2193, 2195, 2198, 2200, 2204, 2208)

Bròquil (2190, 2196)

**USOS ALIMENTARIS**

**Inflorescència**

Ingestió de la part de la planta cuita - Cuita en aigua

FONTS 2195, 2208. CONSUMICIÓ. Bullit (2208). DESTINACIÓ. Alimentació humana (2195, 2208).

No consta el tipus d'ingestió - No consta el mode de preparació

FONTS 2190, 2193, 2196, 2198, 2200. DESTINACIÓ. Alimentació humana.

***Bromus catharticus***Vahl (gramínies)
BCN 126551

**NOMS POPULARS**

Ordi bord (2160)

***Calendula arvensis***L. (compostes)
BCN 127886

**NOMS POPULARS**

Calèndula (2213, 2214)

**USOS MEDICINALS**

**Inflorescència**

Antisèptic extern

FONT 2214. DESCRIPCIÓ DE L'ÚS FETA PER L'INFORMANT. Es fan emplastres per a curar a ferides, ja que és desinfectant. FORMA FARMACÈUTICA I ÚS. Emplastre (ús extern). DESTINACIÓ. Medicina humana.

**USOS ALIMENTARIS**

**Flor**

No consta el tipus d'ingestió - No consta el mode de preparació

FONT 2213. DESCRIPCIÓ DE L'ÚS FETA PER L'INFORMANT. Les lígules del capítol [dit "els pètals"] es fan servir en amanides i en pastisseria. DESTINACIÓ. Alimentació humana.

***Calendula officinalis***L. (compostes)
BCN 127889

**NOMS POPULARS**

Boixac (3935)

Calèndula (2164, 2178, 3933, 3952, 2228)

**USOS MEDICINALS**

**Inflorescència**

Analgèsic

FONT 3935. DESCRIPCIÓ DE L'ÚS FETA PER L'INFORMANT. Disminueix el dolor en càncers no operables. FORMA FARMACÈUTICA I ÚS. Desconegut per l'informant / No consta. DESTINACIÓ. Medicina humana.

Antiberrugós

FONT 3952. DESCRIPCIÓ DE L'ÚS FETA PER L'INFORMANT. En aplicació local fa desaparèixer les berrugues víriques de la pell. S’aplica en compresa sucada en oli de calèndula. FORMA FARMACÈUTICA I ÚS. Embrocació (ús extern). DESTINACIÓ. Medicina humana.

Antidismenorreic

FONT 3935. DESCRIPCIÓ DE L'ÚS FETA PER L'INFORMANT. Regulador de la menstruació. FORMA FARMACÈUTICA I ÚS. Desconegut per l'informant / No consta. DESTINACIÓ. Medicina humana.

Antiinflamatori / Antiàlgic / Antiequimòtic

FONT 2228. DESCRIPCIÓ DE L'ÚS FETA PER L'INFORMANT. Antiinflamatori. Hi ha pomades que en contenen i es comercialitzen. FORMA FARMACÈUTICA I ÚS. Desconegut per l'informant / No consta. DESTINACIÓ. Medicina humana.

Antiulcerós (per a úlceres gàstriques)

FONT 3952. DESCRIPCIÓ DE L'ÚS FETA PER L'INFORMANT. Té la capacitat de cicatritzar les llagues d'estómac i duodè (antiulcerosa). FORMA FARMACÈUTICA I ÚS. Desconegut per l'informant / No consta. DESTINACIÓ. Medicina humana.

Per a trastorns de la pell o del teixit subcutani

FONTS 2164, 3933, 3935, 3952. DESCRIPCIÓ DE L'ÚS FETA PELS INFORMANTS. L'oli de calèndula suavitza i hidrata la pell. L'oli i la pomada donen molt bons resultats en el tractament de cremades i èczemes (3952). FORMA FARMACÈUTICA I ÚS. Crema (ús extern) (2164). Desconegut per l'informant / No consta (3933). Bany (ús extern) (3935). Liniment (ús extern) (3952). PREPARACIÓ. Es fiquen les lígules florals [dit "pètals"] a la banyera (3935). Es posen a macerar les seves inflorescències [dit "flors"] amb oli d'oliva i es deixen en un lloc fresc durant 12 hores. Després es filtra amb un colador i es guarda en un pot de vidre tapat i en un lloc fresc i sec (3952). DESTINACIÓ. Medicina humana.

Per a trastorns del sistema digestiu

FONT 3952. DESCRIPCIÓ DE L'ÚS FETA PER L'INFORMANT. És efectiva en problemes digestius perquè desinflama. S’ha de prendre en infusió. FORMA FARMACÈUTICA I ÚS. Infusió (ús intern). DESTINACIÓ. Medicina humana.

Vulnerari

FONT 3952. DESCRIPCIÓ DE L'ÚS FETA PER L'INFORMANT. Aplicada localment, accelera la curació de ferides, cremades, furóncols i èczemes. S’aplica en compreses i rentats, cataplasmes de pètals frescos o suc fresc de les inflorescències [dit "flors"]. FORMA FARMACÈUTICA I ÚS. Cataplasma (ús extern). DESTINACIÓ. Medicina humana.

**USOS ALIMENTARIS**

**Inflorescència**

Condiment

FONT 3935. DESCRIPCIÓ DE L'ÚS FETA PER L'INFORMANT. Se’n posen les flors ligulades [dit "pètals"] a l’arròs, sopes i peix; els dona un bon sabor. DESTINACIÓ. Alimentació humana.

Ingestió de la part de la planta crua - Fresca (sense preparació)

FONT 2228. DESCRIPCIÓ DE L'ÚS FETA PER L'INFORMANT. Les flors ligulades [dit “els pètals”] en amanida. CONSUMICIÓ. Amanida. DESTINACIÓ. Alimentació humana.

**ALTRES USOS**

**Planta viva *in situ***

Agrosilvopastoral

FONTS 2164, 2178. DESCRIPCIÓ DE L'ÚS FETA PELS INFORMANTS. Repel·lent de la tuta absoluta (2164). És font de fauna auxiliar (2178). OBSERVACIONS. Plaguicides naturals (2164). Associació de cultius i equilibri sistèmic (2178).

**BARREGES AMB AQUEST TÀXON (vegeu catàleg de barreges)**

**USOS MEDICINALS**

**Inflorescència**

FONT 2228. Oli de boixac i rosa.

***Cannabis sativa***L. (cannabàcies)
BCN 24735

**NOMS POPULARS**

Cànem (2231)

**USOS ALIMENTARIS**

**Llavor**

No consta el tipus d'ingestió - No consta el mode de preparació

FONT 2231. DESCRIPCIÓ DE L'ÚS FETA PER L'INFORMANT. La llavor és aliment per als canaris. DESTINACIÓ. Alimentació animal.

***Capsella bursa-pastoris***(L.) Medic. (crucíferes)
BCN 129698

**NOMS POPULARS**

Bossa de pastor (2214)

**USOS ALIMENTARIS**

**Part aèria**

No consta el tipus d'ingestió - No consta el mode de preparació

FONT 2214. DESCRIPCIÓ DE L'ÚS FETA PER L'INFORMANT. Se'n menja. DESTINACIÓ. Alimentació humana.

***Capsicum annuum***L. (solanàcies)
BCN-E-267

**NOMS POPULARS**

Pebrot (3700, 2221)

Pebrot (fruit) (2167, 2186, 2200, 2210)

Pebrot del Vendrell (raça) (3700)

Pebrot de Reus (raça) (3700)

Pebrot del Padrón (raça) (fruit) (2201, 2205)

Pebrot morro de llebre (raça) (3700)

Pebrot picant (fruit) (raça) (2201)

Pebrot roig (fruit) (2218)

Pebrot verd (fruit) (2202, 2206, 2218)

Pebrot vermell (fruit) (2178, 2199, 2206, 2208)

*Pimiento del Padrón* (castellà) (2180)

**USOS MEDICINALS**

**Fruit**

Salutífer

FONT 3700. DESCRIPCIÓ DE L'ÚS FETA PER L'INFORMANT. Ajuda a incrementar les defenses de l’organisme. FORMA FARMACÈUTICA I ÚS. Sense forma farmacèutica (ús directe) (ús intern). DESTINACIÓ. Medicina humana.

**USOS ALIMENTARIS**

**Fruit**

Ingestió de la part de la planta crua - Fresca (sense preparació)

FONT 2202. DESTINACIÓ. Alimentació humana.

Ingestió de la part de la planta cuita - Cuita sense vehicle

FONTS 2202, 2218. DESCRIPCIÓ DE L'ÚS FETA PELS INFORMANTS. Per a fer samfaina (2218). DESTINACIÓ. Alimentació humana (2202, 2218).

Ingestió de la part de la planta cuita - Cuita en oli

FONTS 2202, 2205, 2221. DESCRIPCIÓ DE L'ÚS FETA PELS INFORMANTS. Fregit (2221). DESTINACIÓ. Alimentació humana (2202, 2205, 2221).

No consta el tipus d'ingestió - No consta el mode de preparació

FONTS 2167, 2178, 2186, 2199, 2200, 2201, 2206, 2208, 2210, 3700. DESCRIPCIÓ DE L'ÚS FETA PELS INFORMANTS. Se'n menja (2167). DESTINACIÓ. Alimentació humana (2167, 2178, 2186, 2199, 2200, 2201, 2206, 2208, 2210, 3700).

**ALTRES USOS**

**Fruit**

Literatura oral popular: llegendes, gloses, contes, dites, refranys, poemes, cançons

FONT 2180. DESCRIPCIÓ DE L'ÚS FETA PER L'INFORMANT. *Unos pican y otros no* (castellà) [referit als pebrots del Padrón].

***Carica papaya***L. (caricàcies)
BCN-E-280

**NOMS POPULARS**

Papaia (fruit) (2200, 2205)

**USOS ALIMENTARIS**

**Fruit**

Ingestió de la part de la planta crua - Fresca (sense preparació)

FONTS 2200, 2205. DESTINACIÓ. Alimentació humana.

***Carum carvi***L. (umbel·líferes)
BCN 29642

**NOMS POPULARS**

Comí de prat (3935)

**USOS MEDICINALS**

**Fruit**

Antiespasmòdic

FONT 3935. FORMA FARMACÈUTICA I ÚS. Infusió (ús intern). DESTINACIÓ. Medicina humana.

Digestiu

FONT 3935. FORMA FARMACÈUTICA I ÚS. Infusió (ús intern). DESTINACIÓ. Medicina humana.

**USOS ALIMENTARIS**

**Fruit**

Condiment

FONT 3935. DESCRIPCIÓ DE L'ÚS FETA PER L'INFORMANT. Els fruits [dit "llavors"] donen bon sabor als formatges, pastissos, pa i les pomes al forn. DESTINACIÓ. Alimentació humana.

Ingestió de la part de la planta cuita - Cuita sense vehicle

FONT 3935. DESCRIPCIÓ DE L'ÚS FETA PER L'INFORMANT. Els afeccionats als coloms diuen que, si hi ha comí de prat torrat a la menjadora, els coloms sempre tornen. DESTINACIÓ. Alimentació animal.

***Castanea sativa***Mill. (fagàcies)
BCN 29844

**NOMS POPULARS**

Castanya (llavor) (2192)

**USOS ALIMENTARIS**

**Llavor**

Ingestió de la part de la planta cuita - Cuita sense vehicle

FONT 2192. DESCRIPCIÓ DE L'ÚS FETA PER L'INFORMANT. Torrades. DESTINACIÓ. Alimentació humana.

**BARREGES AMB AQUEST TÀXON (vegeu catàleg de barreges)**

**USOS ALIMENTARIS**

**Llavor**

FONT 2192. Platillo de Sant Climent.

***Celtis australis***L. (cannabàcies)
BCN 126567

**NOMS POPULARS**

Lledó (fruit) (2212, 2224)

Lledoner (2167)

**USOS ALIMENTARIS**

**Fruit**

Ingestió de la part de la planta crua - Fresca (sense preparació)

FONT 2224. DESTINACIÓ. Alimentació humana.

**ALTRES USOS**

**Fruit**

Jocs i joguines

FONTS 2167, 2212. DESCRIPCIÓ DE L'ÚS FETA PELS INFORMANTS. Agafaven les boletes de dins i amb un canut de canya jugaven a fer guerres (2167). S’agafaven per a jugar a guerres (2212).

***Centaurea aspera***L. (compostes)
BCN 142616

**NOMS POPULARS**

Escanyaboc (2191)

**USOS MEDICINALS**

**Arrel**

Antihemorroidal

FONT 2191. FORMA FARMACÈUTICA I ÚS. Sense forma farmacèutica (ús directe) (ús extern). MODE D'UTILITZACIÓ/POSOLOGIA. La gent amb problemes de morenes se'n posa l'arrel a la butxaca i es guareixen. La seva dona encara ho fa. DESTINACIÓ. Medicina humana.

***Centaurea cyanus***L. (compostes)
BCN 27257

**NOMS POPULARS**

Blauet (3937)

**USOS MEDICINALS**

**Inflorescència**

Per a la conjuntivitis

FONT 3937. DESCRIPCIÓ DE L'ÚS FETA PER L'INFORMANT. Bona per a la conjuntivitis i altres afeccions oculars. FORMA FARMACÈUTICA I ÚS. Bany (ús extern). PREPARACIÓ. Es fan banys amb l’aigua de bullir la planta. DESTINACIÓ. Medicina humana.

***Ceratonia siliqua***L. (papilionàcies)
BCN 127885

**NOMS POPULARS**

Garrofa (fruit) (2189, 2190, 2191, 2192, 2204, 2210, 2221, 2216, 2217, 2229, 2227, 3934)

Garrofa de sucre (raça) (fruit) (2190)

Garrofa negra (raça) (fruit) (2190)

Garrofa valenciana (raça) (fruit) (2190)

Garrofer (2162, 2189, 2212, 2222, 2226)

**USOS MEDICINALS**

**Fruit**

Antidiarreic

FONTS 2204, 2229. DESCRIPCIÓ DE L'ÚS FETA PELS INFORMANTS. Per a aturar la diarrea (2229). FORMA FARMACÈUTICA I ÚS. Desconegut per l'informant / No consta. DESTINACIÓ. Medicina humana.

Antitussigen

FONT 2229. DESCRIPCIÓ DE L'ÚS FETA PER L'INFORMANT. Se’n fa un remei per a la tos. FORMA FARMACÈUTICA I ÚS. Desconegut per l'informant / No consta. DESTINACIÓ. Medicina humana.

Laxant

FONT 2190. FORMA FARMACÈUTICA I ÚS. Desconegut per l'informant / No consta. DESTINACIÓ. Medicina humana.

**USOS ALIMENTARIS**

**Fruit**

Ingestió de la part de la planta crua - Fresca (sense preparació)

FONTS 2190, 2191, 2192, 2209, 2210, 2216, 2217, 2221, 2222, 2227, 2229, 3934. DESCRIPCIÓ DE L'ÚS FETA PELS INFORMANTS. Per als conills (2209). Per als animals de tir. Se’ls en posava al morralet, un sarró petit per a donar-los de menjar (2210). Quan anaven al Born a vendre, en posaven al morralet, per a donar energia als cavalls (2216). Per als animals (2217). En menjaven els cavalls (2221). La polpa és per al pinso dels animals (2191). Abans, mastegàvem la garrofa (2210). De petita la xuclava (2227). Per als cavalls (2227). Per al menjar dels animals. Els cavalls en menjaven la polpa i escopien les llavors [dit "pinyols"] (2229). Per als cavalls. Ara se la mengen els porcs senglars (3934). DESTINACIÓ. Alimentació animal (2191, 2192, 2209, 2210, 2216, 2217, 2221, 2227, 2229, 3934). Alimentació humana (2190, 2210, 2222, 2227).

Ingestió de la part de la planta cuita - Cuita en aigua

FONT 2192. DESCRIPCIÓ DE L'ÚS FETA PER L'INFORMANT. En temps de la guerra, trinxaves les garrofes amb un morter, es feien bullir i eren com xocolata. DESTINACIÓ. Alimentació humana.

No consta el tipus d'ingestió - No consta el mode de preparació

FONTS 2189, 2190, 2204, 2229. DESCRIPCIÓ DE L'ÚS FETA PELS INFORMANTS. És dolça i abans se'n menjava (2229). DESTINACIÓ. Alimentació humana (2189, 2229). Alimentació animal (2190, 2204).

Preparació de begudes - Beguda preparada amb aigua

FONT 2221. DESCRIPCIÓ DE L'ÚS FETA PER L'INFORMANT. Abans se'n feia cafè, amb la trituració del fruit. DESTINACIÓ. Alimentació humana.

**Llavor**

No consta el tipus d'ingestió - No consta el mode de preparació

FONT 2229. DESCRIPCIÓ DE L'ÚS FETA PER L'INFORMANT. Les llavors [dit "els pinyols"] servien per a adulterar el cafè. DESTINACIÓ. Alimentació humana.

**Pasta de la llavor**

No consta el tipus d'ingestió - No consta el mode de preparació

FONT 2162. DESCRIPCIÓ DE L'ÚS FETA PER L'INFORMANT. La llavor [dit "el pinyol"] es molia i se’n feia una pols per als gelats, que feia que aguantessin més. El que sobrava de la molta de la llavor [dit "pinyol"] es donava al bestiar. DESTINACIÓ. Alimentació humana. Alimentació animal.

**Trituració del gra**

No consta el tipus d'ingestió - No consta el mode de preparació

FONTS 2189, 2191, 2204, 2210. DESCRIPCIÓ DE L'ÚS FETA PELS INFORMANTS. Se’n fa farina, que s’utilitza en pastisseria. Els germans Armengol en compren per a la seva aplicació industrial (2191). Es feia una mena de xocolata desfeta amb farina de garrofa, llet i sucre (2204). La farina era un substitut de la xocolata (2210). DESTINACIÓ. Alimentació animal (2189). Alimentació humana (2191, 2204, 2210).

**ALTRES USOS**

**Part aèria**

Agrosilvopastoral

FONT 2229. DESCRIPCIÓ DE L'ÚS FETA PER L'INFORMANT. Se’n fa terra vegetal quan les branques moren i es descomponen. OBSERVACIONS. Adobs i fertilitzants naturals.

**BARREGES AMB AQUEST TÀXON (vegeu catàleg de barreges)**

**USOS ALIMENTARIS**

**Part aèria jove**

FONT 2190. Olives arreglades.

**USOS MEDICINALS**

**Fruit**

FONT 2229. Remei per a la tos.

***Chamaerops humilis***L. (arecàcies)
BCN 23832

**NOMS POPULARS**

Margalló (3947)

Palma (producte elaborat) (3700)

Palma del Garraf (3947)

Palmó (producte elaborat) (3700)

**ALTRES USOS**

**Fulla**

Ajuda a la llar

FONT 3947. DESCRIPCIÓ DE L'ÚS FETA PER L'INFORMANT. Elaboració d'escombres. OBSERVACIONS. Molts valencians visitaven durant l’estiu les nostres terres per a recollir margalló.

Creences i pràctiques magicoreligioses

FONT 3700. DESCRIPCIÓ DE L'ÚS FETA PER L'INFORMANT. Les seves fulles servien per a fer les palmes i palmons de Diumenge de Rams.

Elaboració de calçat

FONT 3947. DESCRIPCIÓ DE L'ÚS FETA PER L'INFORMANT. Se’n feien espardenyes.

***Chenopodium album***L. (amarantàcies)
BCN 130957

**NOMS POPULARS**

Blet (2160)

Blet pudent (2216)

***Chenopodium murale***L. (amarantàcies)
BCN 126548

**NOMS POPULARS**

Blet (2160, 2216, 2219)

**USOS ALIMENTARIS**

**Fulla**

No consta el tipus d'ingestió - No consta el mode de preparació

FONT 2219. DESCRIPCIÓ DE L'ÚS FETA PER L'INFORMANT. Es poden menjar les fulles com una verdura. DESTINACIÓ. Alimentació humana.

***Cicer arietinum***L. (papilionàcies)
BCN 29659

**NOMS POPULARS**

Cigró (llavor) (2191, 2212)

**USOS ALIMENTARIS**

**Llavor**

Ingestió de la part de la planta cuita - Cuita en aigua

FONT 2191. DESTINACIÓ. Alimentació humana.

**ALTRES OBSERVACIONS**

FONT 2212. DESCRIPCIÓ FETA PER L'INFORMANT. Els cereals i llegums, per a conservar-los, es posaven en sacs ben curullats, amb ferradures i altres peces de metall intercalades i, a sobre, es col·locaven tres o quatre dits de sorra, que s’anava a buscar a la platja abans no sortís el sol.

***Cichorium endivia***L. subsp. ***endivia***(compostes)
BCN 129004

**NOMS POPULARS**

Endívia (2178, 2200, 2208, 2212, 2224)

Escarola (2160, 2178, 2186, 2191, 2192, 2193, 2194, 2200, 2202, 2203, 2204, 2205, 2208, 3700, 2221, 2216, 2228)

Escarola de Santa Coloma (raça) (3700)

Escarola perroqueta (raça) (3700)

Escarolla (2185)

**USOS MEDICINALS**

**Fulla**

Hepatoprotector

FONT 2178. FORMA FARMACÈUTICA I ÚS. Sense forma farmacèutica (ús directe) (ús intern). PREPARACIÓ. En cru. DESTINACIÓ. Medicina humana.

**USOS ALIMENTARIS**

**Fulla**

Ingestió de la part de la planta crua - Fresca (sense preparació)

FONTS 2160, 2185, 2194, 2202, 2203, 2204, 2216, 2221, 3700. DESCRIPCIÓ DE L'ÚS FETA PELS INFORMANTS. Se'n menja crua. Se'n fa el xató (3700). Ho sembraven per encàrrec i ho enviaven a fora. En menjaven a França (2185). CONSUMICIÓ. Amanida (2160, 2185, 2204). DESTINACIÓ. Alimentació humana (2160, 2185, 2194, 2202, 2203, 2204, 2216, 2221, 3700).

Ingestió de la part de la planta cuita - Cuita en aigua

FONTS 2204, 2228. DESCRIPCIÓ DE L'ÚS FETA PELS INFORMANTS. Per a fer caldo (2228). CONSUMICIÓ. Bullit (2204). DESTINACIÓ. Alimentació humana (2204, 2228).

Ingestió de la part de la planta cuita - Cuita en oli

FONT 2204. DESCRIPCIÓ DE L'ÚS FETA PER L'INFORMANT. Saltada a la paella o en truita. DESTINACIÓ. Alimentació humana.

No consta el tipus d'ingestió - No consta el mode de preparació

FONTS 2191, 2193, 2200, 2205, 2208. DESTINACIÓ. Alimentació humana (2191, 2193, 2200, 2205, 2208).

**Fulla vella**

No consta el tipus d'ingestió - No consta el mode de preparació

FONT 2186. DESCRIPCIÓ DE L'ÚS FETA PER L'INFORMANT. Per a les vaques. DESTINACIÓ. Alimentació animal.

**ALTRES OBSERVACIONS**

FONT 2216. DESCRIPCIÓ FETA PER L'INFORMANT. Quan fa fred és tendra. Amb tres dies lligada en té prou perquè quedi blanca.

**BARREGES AMB AQUEST TÀXON (vegeu catàleg de barreges)**

**USOS ALIMENTARIS**

**Fulla**

FONT 2228. Caldo depuratiu.

***Cichorium intybus***L. (compostes)
BCN 296600

**NOMS POPULARS**

Masteguera (3935)

Xicoira (3952, 2226)

Xicòria (2211)

**USOS MEDICINALS**

**Arrel**

Per a trastorns del sistema digestiu

FONT 3952. DESCRIPCIÓ DE L'ÚS FETA PER L'INFORMANT. Infusió amb fulles i arrels fresques o seques. Se'n prenen dues o tres tasses diàries com a aperitiu abans de menjar o com a digestiu després. FORMA FARMACÈUTICA I ÚS. Infusió (ús intern). DESTINACIÓ. Medicina humana.

**Fulla**

Per a trastorns del sistema digestiu

FONT 3952. DESCRIPCIÓ DE L'ÚS FETA PER L'INFORMANT. Infusió amb fulles i arrels fresques o seques. Se'n prenen dues o tres tasses diàries com a aperitiu abans de menjar o com a digestiu per a després. FORMA FARMACÈUTICA I ÚS. Infusió (ús intern). DESTINACIÓ. Medicina humana.

**USOS ALIMENTARIS**

**Arrel**

Preparació de begudes - Beguda preparada amb aigua

FONTS 2211, 2226, 3952. DESCRIPCIÓ DE L'ÚS FETA PELS INFORMANTS. Com a substitut del cafè (2211). En temps difícils, servia com a cafè (2226). L'arrel de la xicoira s'arrenca a la tardor, assecada, torrada i triturada, serveix per a preparar el cafè de xicoira (3952). DESTINACIÓ. Alimentació humana (2211, 2226, 3952).

**Fulla**

Ingestió de la part de la planta crua - Fresca (sense preparació)

FONT 3952. DESCRIPCIÓ DE L'ÚS FETA PER L'INFORMANT. Fulles crues en amanida. S’han de collir abans de la floració per tal que no siguin tan amargants. CONSUMICIÓ. Amanida. DESTINACIÓ. Alimentació humana.

**Inflorescència**

Ingestió de la part de la planta crua - Fresca (sense preparació)

FONT 3935. DESCRIPCIÓ DE L'ÚS FETA PER L'INFORMANT. Per a les amanides. DESTINACIÓ. Alimentació humana.

***Cinnamomum verum***J.Presl (lauràcies)
BCN47283

**NOMS POPULARS**

Canyella (escorça) (2211)

**BARREGES AMB AQUEST TÀXON (vegeu catàleg de barreges)**

**USOS ALIMENTARIS**

**Tija**

FONT 2211. Licor de llet.

***Cirsium arvense***(L.) Scop. (compostes)
BCN 130947

**NOMS POPULARS**

Calcida (2210, 2219)

***Cistus albidus***L. (cistàcies)
BCN 129705

**NOMS POPULARS**

*Jara blanca* (castellà) (2214)

**ALTRES USOS**

**Fulla**

Ajuda a la llar

FONT 2214. DESCRIPCIÓ DE L'ÚS FETA PER L'INFORMANT. Les seves fulles s’han utilitzat per a netejar les sabates de pell.

***Cistus salviifolius***L. (cistàcies)
BCN 36767

**NOMS POPULARS**

Estepa borrera (2229)

***Citrullus lanatus***(Thunb.) Matsumara et Nakai (cucurbitàcies)
BCN-E-254

**NOMS POPULARS**

Meló de moro (fruit) (2212)

Síndria (2184, 2184, 3700, 2218)

Síndria (fruit) (2200, 2206, 2210, 2212, 3933, 3936)

Síndria de confiter (raça) (fruit) (2212)

Síndria ratllada (raça) (fruit) (2194)

**USOS ALIMENTARIS**

**Fruit**

Ingestió de la part de la planta crua - Fresca (sense preparació)

FONTS 2194, 2200, 2206, 2210, 2218, 3700, 3933, 3936. DESCRIPCIÓ DE L'ÚS FETA PELS INFORMANTS. Comestible (2218). DESTINACIÓ. Alimentació humana (2194, 2200, 2206, 2210, 2218, 3700, 3933, 3936).

**Epicarpi**

Ingestió de la part de la planta cuita - Cuita en sucre

FONT 2212. DESCRIPCIÓ DE L'ÚS FETA PER L'INFORMANT. L'epicarpi [dit "pela"] de la síndria del confiter era ideal per a fer-ne confitura. Algunes peces feien fins a 40 quilos. Cultiu ja desaparegut. DESTINACIÓ. Alimentació humana.

**Mesocarpi**

Ingestió de la part de la planta cuita - Cuita en sucre

FONT 2212. DESCRIPCIÓ DE L'ÚS FETA PER L'INFORMANT. El mesocarpi [dit "pela"] de la síndria del confiter era ideal per a fer-ne confitura. Algunes peces feien fins a 40 quilos. Cultiu ja desaparegut. DESTINACIÓ. Alimentació humana.

**ALTRES USOS**

**Fruit**

Jocs i joguines

FONT 2212. DESCRIPCIÓ DE L'ÚS FETA PER L'INFORMANT. Com a joc, la canalla les buidava, en feien quatre forats a la closca i a dins hi posaven una espelma.

**ALTRES OBSERVACIONS**

FONTS 2210, 2212. DESCRIPCIÓ FETA PELS INFORMANTS. Es fa un vall amb fems orgànics per a plantar-hi a sobre (2210). Per a obtenir llavors per a l’any següent, els pagesos convidaven els seus veïns, durant qualsevol calorosa nit d’agost, a menjar un parell de les síndries més grans que tinguessin, a condició de deixar les llavors sobre un gran llençol de sac que estenien al mig del carrer (2212).

**BARREGES AMB AQUEST TÀXON (vegeu catàleg de barreges)**

**USOS ALIMENTARIS**

**Fruit**

FONT 2184. Arrop.

***Citrus aurantiifolia***(Christm.) Swingle (rutàcies)
BCN-E 307

**NOMS POPULARS**

Llima (fruit) (2200, 2206)

**USOS ALIMENTARIS**

**Fruit**

No consta el tipus d'ingestió - No consta el mode de preparació

FONTS 2200, 2206. DESTINACIÓ. Alimentació humana.

***Citrus aurantium***L. (rutàcies)
BCN 46080

**NOMS POPULARS**

Taronger bord (2204, 3700)

Taronja agra (fruit) (2204)

**USOS MEDICINALS**

**Fulla**

Desconegut per l'informant

FONT 2204. FORMA FARMACÈUTICA I ÚS. Infusió (ús intern). MODE D'UTILITZACIÓ/POSOLOGIA. Se'n bevia dos o tres cops al dia. DESTINACIÓ. Medicina humana.

**ALTRES USOS**

**Fruit**

Ajuda en la matança del porc

FONTS 2204, 3700. DESCRIPCIÓ DE L'ÚS FETA PELS INFORMANTS. Servia perquè la mocadera netegés la moca dels animals (el conjunt d’estómac, budells i entranyes) durant la matança (3700). Servien per a la matança del porc, ja que, amb els àcids, s’havien de fregar i netejar els budells girats a l’inrevés (2204).

***Citrus deliciosa***Ten. (rutàcies)
BCN-E-204

**NOMS POPULARS**

Mandarina (fruit) (2194, 2195, 2199, 2200, 2201, 2202, 2225)

Mandariner (3936)

**USOS ALIMENTARIS**

**Fruit**

Ingestió de la part de la planta crua - Fresca (sense preparació)

FONTS 2194, 2195, 2199, 2200, 2201, 2202, 2225, 3936. DESCRIPCIÓ DE L'ÚS FETA PELS INFORMANTS. Comestible (2225). DESTINACIÓ. Alimentació humana (2194, 2195, 2199, 2200, 2201, 2202, 2225, 3936).

***Citrus japonica***Thunb. (rutàcies)
BCN-E-203

**NOMS POPULARS**

Cumquat (2193, 2194, 2200)

Mandariner xinès (2167)

**USOS ALIMENTARIS**

**Fruit**

Ingestió de la part de la planta crua - Fresca (sense preparació)

FONTS 2167, 2193, 2194, 2200. DESCRIPCIÓ DE L'ÚS FETA PELS INFORMANTS. Se'n menja amb pell i tot (2167). DESTINACIÓ. Alimentació humana (2167, 2193, 2194, 2200).

***Citrus limon***(L.) Burm. (rutàcies)
BCN 129012

**NOMS POPULARS**

*Limonero* (castellà) (2176, 2177)

Llimona (fruit) (2174, 2176, 2184, 2188, 2189, 2193, 2194, 2198, 2199, 2201, 2202, 2204, 2206, 3700, 3932, 2225)

Llimoner (2173, 2174, 2188, 3936, 2218, 2228)

**USOS MEDICINALS**

**Flor**

Tranquil·litzant

FONT 2228. DESCRIPCIÓ DE L'ÚS FETA PER L'INFORMANT. Calmant. FORMA FARMACÈUTICA I ÚS. Desconegut per l'informant / No consta. DESTINACIÓ. Medicina humana.

**Fruit**

Per a trastorns del sistema sanguini

FONT 2174. DESCRIPCIÓ DE L'ÚS FETA PER L'INFORMANT. Contra l'acidesa en sang. FORMA FARMACÈUTICA I ÚS. Infusió (ús intern). DESTINACIÓ. Medicina humana.

**Suc del fruit**

Antiàcid

FONTS 2176, 2177. DESCRIPCIÓ DE L'ÚS FETA PELS INFORMANTS. Va bé per a l'acidesa. FORMA FARMACÈUTICA I ÚS. Sense forma farmacèutica (ús directe) (ús intern). DESTINACIÓ. Medicina humana.

Antiferropènic

FONTS 2176, 2177. DESCRIPCIÓ DE L'ÚS FETA PELS INFORMANTS. Se'n consumeix el suc per a absorbir el ferro de la carn i el peix. FORMA FARMACÈUTICA I ÚS. Sense forma farmacèutica (ús directe) (ús intern). DESTINACIÓ. Medicina humana.

Antiinflamatori faringi

FONT 2204. FORMA FARMACÈUTICA I ÚS. Gargarisme (ús extern). MODE D'UTILITZACIÓ/POSOLOGIA. Per a guarir el mal de gola, s’havien de fer gàrgares amb una barreja d’aigua, suc de llimona i mel, tot ben calent. DESTINACIÓ. Medicina humana.

Per a trastorns del sistema sanguini

FONTS 2173, 2174. DESCRIPCIÓ DE L'ÚS FETA PELS INFORMANTS. Per a l'acidesa de sang (2173). Contra l'acidesa en sang. (2174). FORMA FARMACÈUTICA I ÚS. Sense forma farmacèutica (ús directe) (ús intern). DESTINACIÓ. Medicina humana.

Per al refredat

FONT 3932. DESCRIPCIÓ DE L'ÚS FETA PER L'INFORMANT. Per al refredat. Conté molta vitamina C. FORMA FARMACÈUTICA I ÚS. Sense forma farmacèutica (ús directe) (ús intern). MODE D'UTILITZACIÓ/POSOLOGIA. Es pot fer aigua amb mel i llimona. DESTINACIÓ. Medicina humana.

**USOS ALIMENTARIS**

**Fruit**

Ingestió de la part de la planta crua - Fresca (sense preparació)

FONT 2202. DESTINACIÓ. Alimentació humana.

No consta el tipus d'ingestió - No consta el mode de preparació.

FONTS 2188, 2193, 2194, 2198, 2199, 2201, 2206, 2218, 2225, 3936. DESCRIPCIÓ DE L'ÚS FETA PELS INFORMANTS. Comestible (2225). Se’n menja el fruit (2218). DESTINACIÓ. Alimentació humana (2188, 2193, 2194, 2198, 2199, 2201, 2206, 2218, 2225, 3936).

**ALTRES USOS**

**Epicarpi**

Elaboració d'obres artístiques

FONT 2225. DESCRIPCIÓ DE L'ÚS FETA PER L'INFORMANT. La pell del fruit serveix per a elaborar les paneres artístiques.

**Fruit**

Ajuda en la matança del porc

FONT 3700. DESCRIPCIÓ DE L'ÚS FETA PER L'INFORMANT. Servia perquè la mocadera netegés la moca dels animals (el conjunt d’estómac, budells i entranyes) durant la matança.

**Mesocarpi**

Elaboració d'obres artístiques

FONT 2225. DESCRIPCIÓ DE L'ÚS FETA PER L'INFORMANT. La pell del fruit serveix per a elaborar les paneres artístiques.

**BARREGES AMB AQUEST TÀXON (vegeu catàleg de barreges)**

**USOS ALIMENTARIS**

**Fruit**

FONT 2184. Arrop.

FONT 2192. Olives arreglades.

FONT 2228. Caldo depuratiu.

***Citrus paradisi***Macfadyen in Hooker (rutàcies)
BCN-E-263

**NOMS POPULARS**

*Pomelo* (fruit) (castellà) (2199, 2200)

Aranja (fruit) (2206)

**USOS ALIMENTARIS**

**Fruit**

Ingestió de la part de la planta crua - Fresca (sense preparació)

FONTS 2199, 2200, 2206. DESTINACIÓ. Alimentació humana.

***Citrus sinensis***(L.) Osbeck (rutàcies)
BCN-E-237

**NOMS POPULARS**

*Azahar* (flor) (castellà) (2228)

Taronger (2204, 2212, 2224)

Taronja (fruit) (2193, 2195, 2196, 2198, 2199, 2202, 2211, 2216, 2225)

Taronja sanguina (raça) (fruit) (2205)

**USOS MEDICINALS**

**Flor**

Tranquil·litzant

FONT 2228. DESCRIPCIÓ DE L'ÚS FETA PER L'INFORMANT. Calmant. FORMA FARMACÈUTICA I ÚS. Desconegut per l'informant / No consta. DESTINACIÓ. Medicina humana.

**USOS ALIMENTARIS**

**Fruit**

Ingestió de la part de la planta crua - Fresca (sense preparació)

FONTS 2205, 2216. DESTINACIÓ. Alimentació humana.

No consta el tipus d'ingestió - No consta el mode de preparació

FONTS 2193, 2195, 2196, 2198, 2199, 2202, 2225. DESCRIPCIÓ DE L'ÚS FETA PELS INFORMANTS. Comestible (2225). DESTINACIÓ. Alimentació humana (2193, 2195, 2196, 2198, 2199, 2202, 2225).

**ALTRES USOS**

**Epicarpi**

Elaboració d'obres artístiques

FONT 2225. DESCRIPCIÓ DE L'ÚS FETA PER L'INFORMANT. La pell del fruit serveix per a elaborar les paneres artístiques.

**BARREGES AMB AQUEST TÀXON**

**USOS ALIMENTARIS**

**Fruit**

FONT 2211. Licor de llet.

***Cocos nucifera***L. (arecàcies)
BCN-E 309

**NOMS POPULARS**

Coco (fruit) (2200)

**USOS ALIMENTARIS**

**Fruit**

No consta el tipus d'ingestió - No consta el mode de preparació

FONT 2200. DESTINACIÓ. Alimentació humana.

***Convolvulus arvensis***L. (convolvulàcies)
BCN 126547

**NOMS POPULARS**

Corretjola (2163, 2167, 2170, 2171, 2178, 2192, 2216)

**USOS ALIMENTARIS**

**Flor**

Ingestió de la part de la planta crua - Fresca (sense preparació)

FONTS 2170, 2171. DESCRIPCIÓ DE L'ÚS FETA PELS INFORMANTS. De petit, se'n menjaven les flors perquè eren dolces. CONSUMICIÓ. Llaminadura. DESTINACIÓ. Alimentació humana.

**Part aèria**

Ingestió de la part de la planta crua - Fresca (sense preparació)

FONT 2192. DESCRIPCIÓ DE L'ÚS FETA PER L'INFORMANT. Per als conills. DESTINACIÓ. Alimentació animal.

**ALTRES OBSERVACIONS**

FONT 2167. DESCRIPCIÓ FETA PER L'INFORMANT. S'enreda pertot arreu i no la mates mai.

***Conyza bonariensis***(L.) Cronq. (compostes)
BCN 126544

**NOMS POPULARS**

Miquelet (2178, 2219)

Trencadalles (2160)

**ALTRES OBSERVACIONS**

FONT 2160. DESCRIPCIÓ FETA PER L'INFORMANT. Els herbicides no li fan res.

***Conyza sumatrensis***(Retz.) E.Walker (compostes)
BCN 130937

**NOMS POPULARS**

Miquelet (2160, 2216, 2219)

**ALTRES USOS**

**Tija amb fulles/branques**

Elaboració d'escombres

FONT 2216.

***Coriandrum sativum***L. (umbel·líferes)
BCN 126574

**NOMS POPULARS**

Cilandre (2165)

*Cilantro* (castellà) (2163)

**USOS ALIMENTARIS**

**Fulla**

Ingestió de la part de la planta crua - Fresca (sense preparació)

FONT 2163. DESCRIPCIÓ DE L'ÚS FETA PER L'INFORMANT. Per a salses, guacamoles i *cebiches*. DESTINACIÓ. Alimentació humana.

***Coriaria myrtifolia***L. (coriariàcies)
BCN 132990

**NOMS POPULARS**

Rondor (2187, 2191)

**ALTRES USOS**

**Tija amb fulles/branques**

Agrosilvopastoral

FONTS 2187, 2191. DESCRIPCIÓ DE L'ÚS FETA PELS INFORMANTS. Es feia servir com a cobriment de les cireres quan es ficaven en les caixes (bitllots), per a protegir-les (2187). Se’n posava una capa a sobre de les cireres, per tal que la tapa de les caixes no les aixafés (2191).

***Coronopus didymus***(L.) Sm. (crucíferes)
BCN 126549

**NOMS POPULARS**

Cervell de gat (2160)

***Corylus avellana*** L. (betulàcies)
BCN 29831

**NOMS POPULARS**

Avellana (bessó) (2192)

**BARREGES AMB AQUEST TÀXON (vegeu catàleg de barreges)**

**USOS ALIMENTARIS**

**Bessó**

FONT 2192. Platillo de Sant Climent.

***Crataegus monogyna***Jacq. (rosàcies)
BCN 150360

**NOMS POPULARS**

Arç blanc (3952)

**USOS MEDICINALS**

**No consta**

Antihipertensor

FONT 3952. DESCRIPCIÓ DE L'ÚS FETA PER L'INFORMANT. Té un efecte regulador de la tensió arterial. FORMA FARMACÈUTICA I ÚS. Desconegut per l'informant / No consta. DESTINACIÓ. Medicina humana.

Sedant

FONT 3952. FORMA FARMACÈUTICA I ÚS. Desconegut per l'informant / No consta. DESTINACIÓ. Medicina humana.

***Craterellus cornucopioides***(L.) Pers. (cantarel·làcies)
BCN-E-604

**NOMS POPULARS**

Trompeta de la mort (2203)

**USOS ALIMENTARIS**

**Part aèria**

Ingestió de la part de la planta cuita - Cuita en oli

FONT 2203. DESCRIPCIÓ DE L'ÚS FETA PER L'INFORMANT. Se'n menja. DESTINACIÓ. Alimentació humana.

***Craterellus lutescens***(Fr.) Fr. (cantarel·làcies)
BCN-E-598

**NOMS POPULARS**

Camagroc (2194, 2210)

**USOS ALIMENTARIS**

**Part aèria**

Ingestió de la part de la planta cuita - Cuita en oli

FONTS 2194, 2210. DESCRIPCIÓ DE L'ÚS FETA PER L'INFORMANT. Es conserven assecant-los (2210). DESTINACIÓ. Alimentació humana (2194, 2210).

***Crocus sativus***L. (iridàcies)
BCN 32170

**NOMS POPULARS**

Safrà (2212)

**USOS ALIMENTARIS**

**Estil i/o estigma**

Condiment

FONT 2212. DESTINACIÓ. Alimentació humana.

***Cucumis melo***L. subsp. ***melo***(cucurbitàcies)
BCN-E-268

**NOMS POPULARS**

Meló (fruit) (2184, 2186, 2192, 2194, 2200, 2201, 2203, 2204, 2205, 2206, 2210, 3700, 2221, 2212, 2216, 2217, 3936, 2223, 2218, 2227)

Meló calent (raça) (fruit) (2204)

Meló d'atzavara (raça) (fruit) (2204)

Meló de la guatlla (raça) (fruit) (2204)

Meló de la torre (raça) (fruit) (3700)

Meló escrit (raça) (fruit) (2204)

Meló francès (raça) (fruit) (2204)

Meló groc (raça) (fruit) (2200)

Meló pinyonet (raça) (fruit) (3700)

Meló tendral clar (raça) (fruit) (3700)

Meló tendral negre (raça) (fruit) (2204, 3700)

Meló tendral verd (raça) (fruit) (2204)

**USOS ALIMENTARIS**

**Fruit**

Ingestió de la part de la planta crua - Fresca (sense preparació)

FONTS 2186, 2194, 2200, 2201, 2203, 2206, 2210, 2212, 2216, 2217, 2221, 2227, 3700, 3936. DESCRIPCIÓ DE L'ÚS FETA PELS INFORMANTS. Es conservaven fins a Nadal si es penjaven al lloc més sec de la casa (2212). Comestible. Se’n feien molts en aquesta zona. Ara no, perquè venen d’altres llocs (2216). Se'n menja (2227). DESTINACIÓ. Alimentació humana (2186, 2194, 2200, 2201, 2203, 2206, 2210, 2212, 2216, 2217, 2221, 2227, 3700, 3936).

No consta el tipus d'ingestió - No consta el mode de preparació

FONTS 2205, 2218. DESCRIPCIÓ DE L'ÚS FETA PELS INFORMANTS. Comestible (2218). DESTINACIÓ. Alimentació humana (2205, 2218).

**Epicarpi**

Ingestió de la part de la planta cuita - Cuita en sucre

FONT 2192. DESCRIPCIÓ DE L'ÚS FETA PER L'INFORMANT. La pell, per a fer confitures. DESTINACIÓ. Alimentació humana.

**ALTRES USOS**

**Llavor**

Elaboració d'obres artístiques

FONT 2223. DESCRIPCIÓ DE L'ÚS FETA PER L'INFORMANT. Les llavors serveixen per a elaborar les paneres artístiques.

**ALTRES OBSERVACIONS**

FONT 3700. DESCRIPCIÓ FETA PER L'INFORMANT. “Anar a fer melons”, es deia per a referir-se al conreu d’aquesta fruita.

**BARREGES AMB AQUEST TÀXON (vegeu catàleg de barreges)**

**USOS ALIMENTARIS**

**Fruit**

FONT 2184. Arrop.

***Cucumis melo***L. subsp. ***flexuosus***(L.) Pangalo (cucurbitàcies)
BCN-E-652

**NOMS POPULARS**

*Alficoz* (castellà) (2194)

**USOS ALIMENTARIS**

**Fruit**

Ingestió de la part de la planta crua - Fresca (sense preparació)

FONT 2194. DESCRIPCIÓ DE L'ÚS FETA PER L'INFORMANT. Se’n menja, sobretot la gent del sud d'Espanya. DESTINACIÓ. Alimentació humana.

***Cucumis sativus***L. (cucurbitàcies)
BCN-E-277

**NOMS POPULARS**

Cogombre (2164, 2199, 2201, 2205)

*Pepino* (castellà) (2218)

**USOS ALIMENTARIS**

**Fruit**

Ingestió de la part de la planta crua - Fresca (sense preparació)

FONTS 2164, 2199, 2201, 2205. CONSUMICIÓ. Amanida (2164). DESTINACIÓ. Alimentació humana (2164, 2199, 2201, 2205).

**ALTRES OBSERVACIONS**

FONT 2218. DESCRIPCIÓ FETA PER L'INFORMANT. Si trepitges la punta de la planta del *pepino* (castellà), aquest s’amarga.

***Cucurbita ficifolia***C.D.Bouché in Verh. (cucurbitàcies)
BCN 29980

**NOMS POPULARS**

Carbassa (2170, 2171, 2187)

Carbassa (fruit) (2217)

Carbassa de cabell d'àngel (fruit) (2178, 2211, 2212)

**USOS ALIMENTARIS**

**Fruit**

Ingestió de la part de la planta cuita - Cuita en sucre

FONTS 2170, 2171, 2178, 2187, 2212, 2217. DESCRIPCIÓ DE L'ÚS FETA PELS INFORMANTS. Se'n fa el cabell d'àngel (2170, 2171). Se'n fan dolços (2178). Confitura (2187). Per a fer confitura (2212). Per a fer melmelades (2217). CONSUMICIÓ. Postres (2178). DESTINACIÓ. Alimentació humana (2170, 2171, 2178, 2187, 2212, 2217).

***Cucurbita maxima***Duch. in Lam. (cucurbitàcies)
BCN-E-200

**NOMS POPULARS**

Carabassa (fruit) (2175, 2212)

Carabassa de porc (raça) (fruit) (2212)

Carbassa (fruit) (2167, 2168, 2169, 2173, 2174, 2177, 2178, 2182, 2189, 2193, 2194, 2196, 2197, 2198, 2200, 2201, 2202, 2204, 2205, 2208, 2209, 2210, 2211, 2216, 2217, 2218, 3700, 3936, 3946)

Carbassa de bon gust (raça) (fruit) (2210)

Carbassa de rabequet (raça) (fruit) (2209)

Carbassera (2178)

**USOS MEDICINALS**

**No consta**

Per a la melsa

FONTS 2173, 2174. FORMA FARMACÈUTICA I ÚS. Desconegut per l'informant / No consta. DESTINACIÓ. Medicina humana.

**USOS ALIMENTARIS**

**Flor**

No consta el tipus d'ingestió - No consta el mode de preparació

FONT 3936. DESTINACIÓ. Alimentació humana.

**Fruit**

Ingestió de la part de la planta cuita - Cuita en aigua

FONTS 2168, 2169, 2175, 2176, 2177, 2194, 2208, 2212, 2216. DESCRIPCIÓ DE L'ÚS FETA PELS INFORMANTS. Sopa (2175). Puré (2176, 2177). Per a fer cremes (2194, 2216). Per a fer sopa de carabassa. S’acostumava a fer els dies de pluja, quan els homes no podien anar al camp. Segons diuen, això és perquè les carbasses costaven molt de tallar i les dones no podien fer-ho soles (2212). Les carabasses de porc eren blanques i costelludes. Eren molt grans. Bullida, per a alimentar els porcs (2212). OBSERVACIONS. Es guardaven a l’aire lliure, col·locant-les als terrats o damunt les parets (2212). Sopes (2216). DESTINACIÓ. Alimentació animal (2212). Alimentació humana (2168, 2169, 2175, 2176, 2177, 2194, 2208, 2212, 2216).

No consta el tipus d'ingestió - No consta el mode de preparació

FONTS 2178, 2182, 2193, 2196, 2197, 2198, 2200, 2201, 2202, 2205, 2210, 2211, 2217, 2218, 3700, 3936, 3946. DESCRIPCIÓ DE L'ÚS FETA PELS INFORMANTS. Per al bestiar (2211). El fruit s’utilitza per a alimentar el bestiar (3700). En donaven als porcs (2217). Se’n menja (2218). DESTINACIÓ. Alimentació humana (2178, 2182, 2193, 2196, 2197, 2198, 2200, 2201, 2202, 2205, 2210, 2211, 2218, 3700, 3936, 3946). Alimentació animal (2211, 2217, 3700).

**ALTRES USOS**

**Fruit**

Literatura oral popular: llegendes, gloses, contes, dites, refranys, poemes, cançons

FONTS 2167, 2204, 2209, 2218. DESCRIPCIÓ DE L'ÚS FETA PELS INFORMANTS. Es diu "aquest xicot creix com una carbassa", en referència al fet que una única planta pot ocupar tot un camp (2167). “De carbassa, amb poca n’hi ha massa” (2204). “De carbassa, no me’n donis massa i de rabequet ben poquet” [rabequet és una raça de carbassa] (2209). “Creix més que les carbasses” (2218).

**ALTRES OBSERVACIONS**

FONT 3700. DESCRIPCIÓ FETA PER L'INFORMANT. Es poden recollir quan s’asseca el peduncle [dit "la tija que uneix el fruit amb la planta"].

***Cucurbita pepo***L. var. ***oblonga*** Link (cucurbitàcies)
BCN-E-258

**NOMS POPULARS**

Carbassó (2164, 2193, 2194)

Carbassó (fruit) (2199, 2200, 2201, 2221, 2224, 3936, 2225, 2228)

**USOS ALIMENTARIS**

**Flor**

No consta el tipus d'ingestió - No consta el mode de preparació

FONT 3936. DESTINACIÓ. Alimentació humana.

**Fruit**

Ingestió de la part de la planta cuita - Cuita en aigua

FONT 2225, 2228. DESCRIPCIÓ DE L'ÚS FETA PER L'INFORMANT. En puré (2225). Per a fer caldo (2228). DESTINACIÓ. Alimentació humana (2225, 2228).

Ingestió de la part de la planta cuita - Cuita en oli

FONTS 2164, 2224, 2225. DESCRIPCIÓ DE L'ÚS FETA PELS INFORMANTS. A la planxa (2225). CONSUMICIÓ. Truita (2224). DESTINACIÓ. Alimentació humana (2164, 2224, 2225).

No consta el tipus d'ingestió - No consta el mode de preparació

FONTS 2193, 2194, 2199, 2200, 2201, 2221, 3936. DESCRIPCIÓ DE L'ÚS FETA PELS INFORMANTS. Se'n menja (2221). DESTINACIÓ. Alimentació humana (2193, 2194, 2199, 2200, 2201, 2221, 3936).

**BARREGES AMB AQUEST TÀXON (vegeu catàleg de barreges)**

**USOS ALIMENTARIS**

**Fruit**

FONT 2228. Caldo depuratiu.

***Cuminum cyminum***L. (umbel·líferes)
BCN-E-275

**NOMS POPULARS**

Comí (2204, 2211)

**USOS MEDICINALS**

**Fruit**

Digestiu

FONT 2211. FORMA FARMACÈUTICA I ÚS. Desconegut per l'informant / No consta. DESTINACIÓ. Medicina humana.

**BARREGES AMB AQUEST TÀXON (vegeu catàleg de barreges)**

**USOS MEDICINALS**

**Fruit**

FONT 2204. Infusió de comí, romaní i sàlvia.

***Curcuma longa***L. (zingiberàcies)
BCN-E-602

**NOMS POPULARS**

Cúrcuma (2200)

**USOS ALIMENTARIS**

**Arrel**

Condiment

FONT 2200. DESTINACIÓ. Alimentació humana.

***Cydonia oblonga***Mill. (rosàcies)
BCN 126540

**NOMS POPULARS**

*Membrillo* (fruit) (castellà) (2168, 2169, 2173, 2174, 2199)

Codony (fruit) (2167, 2170, 2171, 2176, 2177, 2192, 2212, 2229, 2218)

Codonyer (3936)

**USOS MEDICINALS**

**Fruit**

Antidiarreic

FONTS 2173, 2174, 2176, 2177. FORMA FARMACÈUTICA I ÚS. Suspensió (ús intern) (2174). Desconegut per l'informant / No consta (2173, 2176, 2177). MODE D'UTILITZACIÓ/POSOLOGIA. En codonyat (2174). DESTINACIÓ. Medicina humana.

**USOS ALIMENTARIS**

**Fruit**

Ingestió de la part de la planta cuita - Cuita en sucre

FONTS 2167, 2168, 2212, 3936. DESCRIPCIÓ DE L'ÚS FETA PELS INFORMANTS. Se'n fa codonyat (2167). Per a fer codonyat [dit "*membrillo*"] (2168). Per a fer confitura (2212). Se'n fa codonyat (3936). CONSUMICIÓ. Postres (2167). DESTINACIÓ. Alimentació humana (2167, 2168, 2212, 3936).

No consta el tipus d'ingestió - No consta el mode de preparació

FONTS 2169, 2170, 2171, 2199, 2218. DESCRIPCIÓ DE L'ÚS FETA PELS INFORMANTS. Per a fer codonyat [dit "*membrillo*"] (2169). Dels seus fruits se'n fa el codonyat [dit "*carne de membrillo*"] (2170, 2171). Serveix per a fer la carn de codonyat (2218). DESTINACIÓ. Alimentació humana (2169, 2170, 2171, 2199, 2218).

Preparació de begudes - Beguda preparada amb vi

FONT 2229. DESCRIPCIÓ DE L'ÚS FETA PER L'INFORMANT. Per a fer arrop. DESTINACIÓ. Alimentació humana.

***Cymbopogon citratus***Stapf (gramínies)
BCN 72715

**NOMS POPULARS**

Citronel·la (2193)

**USOS ALIMENTARIS**

**Tija**

Condiment

FONT 2193. DESTINACIÓ. Alimentació humana.

***Cynara cardunculus***L. (compostes)
BCN 127872

**NOMS POPULARS**

Card (2193, 2194)

**USOS ALIMENTARIS**

**Tija**

Ingestió de la part de la planta crua - Fresca (sense preparació)

FONT 2194. CONSUMICIÓ. Amanida. DESTINACIÓ. Alimentació humana.

Ingestió de la part de la planta cuita - Cuita en aigua

FONT 2194. DESTINACIÓ. Alimentació humana.

No consta el tipus d'ingestió - No consta el mode de preparació

FONT 2193. DESTINACIÓ. Alimentació humana.

***Cynara scolymus***L. (compostes)
BCN 126536

**NOMS POPULARS**

*Alcachofa* (castellà) (2200)

Carxofa (2160, 2193, 2194, 2195, 2198, 2201, 2205, 2208)

Carxofa (inflorescència) (2196, 2197, 2198, 2199, 2202, 2204, 2215, 2220, 2217, 2225, 2228, 3932, 3934,)

Carxofera (2178, 2204)

Escarxofa (2184, 2185, 3700, 2216)

Escarxofa (inflorescència) (2167, 2182, 2186, 2191, 2221, 2227)

Escarxofa d'Aranjuez (raça) (3700)

Escarxofa de cardet (raça) (3700)

Escarxofa de Tudela (raça) (3700)

Escarxofera (2167, 2182, 2186, 2227)

**USOS MEDICINALS**

**Fulla**

Hepatoprotector

FONTS 2208, 2228. DESCRIPCIÓ DE L'ÚS FETA PELS INFORMANTS. Va bé per al fetge. En decocció (2228). FORMA FARMACÈUTICA I ÚS. Decocció (ús intern). DESTINACIÓ. Medicina humana.

Hipolipemiant

FONTS 2208, 2216. DESCRIPCIÓ DE L'ÚS FETA PELS INFORMANTS. Per al colesterol (2216). FORMA FARMACÈUTICA I ÚS. Decocció (ús intern) (2208). Infusió (ús intern) (2216). DESTINACIÓ. Medicina humana.

**Inflorescència**

Coadjuvant en el tractament antiobesitat

FONT 2178. FORMA FARMACÈUTICA I ÚS. Sense forma farmacèutica (ús directe) (ús intern). PREPARACIÓ. S'ha de menjar bullida. DESTINACIÓ. Medicina humana.

Diürètic

FONTS 2185, 2215. FORMA FARMACÈUTICA I ÚS. Decocció (ús intern). PREPARACIÓ. Se’n fa un caldo amb la inflorescència [dit “fulles”] (2215). DESTINACIÓ. Medicina humana.

Hematocatàrtic

FONT 2217. FORMA FARMACÈUTICA I ÚS. Desconegut per l'informant / No consta. DESTINACIÓ. Medicina humana.

Hepatoprotector

FONTS 2160, 2178, 2204, 2217, 3932. DESCRIPCIÓ DE L'ÚS FETA PELS INFORMANTS. Es bull i se’n pren el caldo (2204). FORMA FARMACÈUTICA I ÚS. Desconegut per l'informant / No consta (2160, 2217). Sense forma farmacèutica (ús directe) (ús intern) (2178). Decocció (ús intern) (2204, 3932). PREPARACIÓ. Consumida com a aliment (2160). S'ha de menjar bullida (2178). DESTINACIÓ. Medicina humana.

Hipolipemiant

FONT 2217. FORMA FARMACÈUTICA I ÚS. Desconegut per l'informant / No consta. DESTINACIÓ. Medicina humana.

Laxant

FONT 2217. FORMA FARMACÈUTICA I ÚS. Desconegut per l'informant / No consta. DESTINACIÓ. Medicina humana.

**Part aèria**

Hepatoprotector

FONT 2186. DESCRIPCIÓ DE L'ÚS FETA PER L'INFORMANT. Venien els renossos (les plantes ja velles) a una gent que feia uns complements medicinals bons per al fetge i el ronyó. FORMA FARMACÈUTICA I ÚS. Desconegut per l'informant / No consta. DESTINACIÓ. Medicina humana.

Protector renal

FONT 2186. DESCRIPCIÓ DE L'ÚS FETA PER L'INFORMANT. Venien els renossos (les plantes ja velles) a una gent que feia uns complements medicinals bons per al fetge i el ronyó. FORMA FARMACÈUTICA I ÚS. Desconegut per l'informant / No consta. DESTINACIÓ. Medicina humana.

**USOS ALIMENTARIS**

**Bràctea**

Ingestió de la part de la planta cuita - Cuita en aigua

FONT 2228. DESCRIPCIÓ DE L'ÚS FETA PER L'INFORMANT. Per a fer caldo. DESTINACIÓ. Alimentació humana.

**Flor**

Quall

FONT 2184. DESCRIPCIÓ DE L'ÚS FETA PER L'INFORMANT. Amb la flor, es quallava la llet per a fer mató. DESTINACIÓ. Alimentació humana.

**Inflorescència**

Ingestió de la part de la planta cuita - Cuita en aigua

FONTS 2204, 2208, 3700. DESCRIPCIÓ DE L'ÚS FETA PELS INFORMANTS. Amb ous durs (3700). CONSUMICIÓ. Bullit (2204, 2208, 3700). DESTINACIÓ. Alimentació humana (2204, 2208, 3700).

Ingestió de la part de la planta cuita - Cuita en oli

FONTS 2204, 2208, 2228, 3700. DESCRIPCIÓ DE L'ÚS FETA PELS INFORMANTS. Es més gustosa quan s’agafen ja fetes (madures) (2204). Arrebossades (2208). Truita (2204, 2208, 2228). DESTINACIÓ.

Alimentació humana (2204, 2208, 2228, 3700).

Ingestió de la part de la planta cuita - Cuita sense vehicle

FONT 3700. DESCRIPCIÓ DE L'ÚS FETA PER L'INFORMANT. A la brasa. DESTINACIÓ. Alimentació humana.

No consta el tipus d'ingestió - No consta el mode de preparació

FONTS 2182, 2191, 2193, 2194, 2195, 2196, 2197, 2199, 2200, 2201, 2202, 2205, 2216, 2220, 2221, 2227, 3934. DESCRIPCIÓ DE L'ÚS FETA PELS INFORMANTS. Les del Prat tenen molt bon gust (2216). Se'n menja (2227). DESTINACIÓ. Alimentació humana (2182, 2191, 2193, 2194, 2195, 2196, 2197, 2199, 2200, 2201, 2202, 2205, 2216, 2220, 2221, 2227, 3934).

Quall

FONTS 2198, 2217, 3700. DESCRIPCIÓ DE L'ÚS FETA PELS INFORMANTS. Es posaven embolicades amb roba dins de la llet i es feia bullir tot junt. Les flors ajudaven a quallar la llet (3700). Per a quallar la llet (2198). Part de la inflorescència [dit “flor”] ajuda a quallar la llet i fer mató (2217). DESTINACIÓ. Alimentació humana (2198, 2217, 3700).

**No consta**

Preparació de begudes - Beguda preparada amb aiguardent

FONT 3700. DESCRIPCIÓ DE L'ÚS FETA PER L'INFORMANT. Hi havia una licor aperitiu fet amb escarxofa. La marca comercial s’anomenava *Cynar®*. DESTINACIÓ. Alimentació humana.

**Part aèria**

No consta el tipus d'ingestió - No consta el mode de preparació

FONT 2186. DESCRIPCIÓ DE L'ÚS FETA PER L'INFORMANT. Les vaques es menjaven la planta, però llavors la llet no era tan bona. DESTINACIÓ. Alimentació animal.

**ALTRES USOS**

**Bràctea i inflorescència**

Elaboració d'obres artístiques

FONT 2225. DESCRIPCIÓ DE L'ÚS FETA PER L'INFORMANT. Per a elaborar les paneres artístiques.

**Inflorescència**

Elaboració de rams

FONT 2216. DESCRIPCIÓ DE L'ÚS FETA PER L'INFORMANT. Ornamental.

**Planta viva *in situ***

Agrosilvopastoral

FONT 2167. DESCRIPCIÓ DE L'ÚS FETA PER L'INFORMANT. Plantades en rengleres, entre els altres cultius, provoquen un equilibri en els camps que fa que hi hagi menys plagues. OBSERVACIONS. Associació de cultius i equilibri sistèmic.

**ALTRES OBSERVACIONS**

FONT 3700. DESCRIPCIÓ FETA PER L'INFORMANT. De la soca vella de l’escarxofera se’n treia un brot. Amb el càvec es feia un forat a terra on es plantava aquest brot, que s’havia de regar abundantment.

**BARREGES AMB AQUEST TÀXON (vegeu catàleg de barreges)**

**USOS ALIMENTARIS**

**Bràctea i inflorescència**

FONT 2228. Caldo depuratiu.

***Cynodon dactylon***(L.) Pers. (gramínies)
BCN 126543

**NOMS POPULARS**

Gram (2204, 2210, 3700, 2219)

Grama (2167)

**USOS MEDICINALS**

**Arrel**

Litotríptic renal

FONT 2204. FORMA FARMACÈUTICA I ÚS. Decocció (ús intern). DESTINACIÓ. Medicina humana.

**ALTRES USOS**

**Planta sencera**

Literatura oral popular: llegendes, gloses, contes, dites, refranys, poemes, cançons

FONT 2204. DESCRIPCIÓ DE L'ÚS FETA PER L'INFORMANT. “[Ser] més dolent que el gram”.

***Cyperus rotundus***L. (ciperàcies)
BCN 126545

**NOMS POPULARS**

Civatilla (3936)

Jonça (2161, 2163, 2178, 2221, 2216, 2219, 3936)

Xufla (arrel) (2219)

**ALTRES OBSERVACIONS**

FONTS 2161, 2178, 2219. DESCRIPCIÓ FETA PELS INFORMANTS. És una mala herba. Si et surt en el patatar, travessa fins les patates (2161). S'ha d'aprendre a conviure amb ella. És important saber combinar els cultius per a què causi la menor molèstia possible (2178). Els senglars en busquen l’arrel [dit “xufla”] (2219).

***Daucus carota***L. subsp. ***sativus***(Hoffm.) Arcang. (umbel·líferes)
BCN 129689

**NOMS POPULARS**

Pastanaga (2164, 2178, 2193, 2194, 2196, 2197, 2198, 2200, 2201, 2202, 2204, 2205, 2206, 2208, 2210, 3700, 2221, 2212, 2217, 3933, 2231, 2225, 2218, 2228)

Pastanaga morada (raça) (2178)

Pastanaga negra (raça) (2198)

**USOS MEDICINALS**

**Arrel**

Antidiarreic

FONT 2208. FORMA FARMACÈUTICA I ÚS. Decocció (ús intern). MODE D'UTILITZACIÓ/POSOLOGIA. L’aigua de pastanaga és bona per a aturar la diarrea. Es bullen quatre o cinc pastanagues i se’n beu l’aigua de bullir tèbia. DESTINACIÓ. Medicina humana.

Per a trastorns de la pell o del teixit subcutani

FONT 2204. DESCRIPCIÓ DE L'ÚS FETA PER L'INFORMANT. Se'n fan sucs. FORMA FARMACÈUTICA I ÚS. Sense forma farmacèutica (ús directe) (ús intern). DESTINACIÓ. Medicina humana.

**USOS ALIMENTARIS**

**Arrel**

Ingestió de la part de la planta crua - Fresca (sense preparació)

FONTS 2164, 2221. CONSUMICIÓ. Amanida (2164). DESTINACIÓ. Alimentació humana (2164, 2221).

Ingestió de la part de la planta cuita - Cuita en aigua

FONTS 2221. DESTINACIÓ. Alimentació humana (2221).

No consta el tipus d'ingestió - No consta el mode de preparació

FONTS 2178, 2193, 2194, 2196, 2197, 2198, 2200, 2201, 2202, 2205, 2206, 2210, 2212, 2218, 3700, 3933. DESCRIPCIÓ DE L'ÚS FETA PELS INFORMANTS. L’arrel servia per a alimentar el bestiar (3700). Comestible (2218). DESTINACIÓ. Alimentació animal (3700). Alimentació humana (2178, 2193, 2194, 2196, 2197, 2198, 2200, 2201, 2202, 2205, 2206, 2210, 2212, 2218, 3933).

**Planta sencera**

No consta el tipus d'ingestió - No consta el mode de preparació

FONT 2217. DESCRIPCIÓ DE L'ÚS FETA PER L'INFORMANT. En donaven als cavalls. DESTINACIÓ. Alimentació animal.

**ALTRES USOS**

**Arrel**

Elaboració d'obres artístiques

FONT 2225. DESCRIPCIÓ DE L'ÚS FETA PER L'INFORMANT. L'epidermis de l'arrel serveix per a elaborar les paneres artístiques.

**ALTRES OBSERVACIONS**

FONT 2212. DESCRIPCIÓ FETA PER L'INFORMANT. Al principi es feien dues anyades –setembre i febrer-, però després s’adonaren que es poden fer tot l’any.

**BARREGES AMB AQUEST TÀXON (vegeu catàleg de barreges)**

**USOS ALIMENTARIS**

**Arrel**

FONT 2228. Caldo depuratiu.

***Diospyros kaki***L.f. (ebenàcies)
BCN-E-201

**NOMS POPULARS**

*Palosanto* (castellà) (2160, 2192, 2198, 2224, 2226)

Caqui (fruit) (2201)

Caquier (2218)

Palosanter (2218)

**USOS ALIMENTARIS**

**Fruit**

Ingestió de la part de la planta crua - Fresca (sense preparació)

FONTS 2192, 2198, 2201, 2218, 2224. DESCRIPCIÓ DE L'ÚS FETA PELS INFORMANTS. Comestible (2218, 2224). DESTINACIÓ. Alimentació humana (2192, 2198, 2201, 2218, 2224).

**ALTRES OBSERVACIONS**

FONT 2218. DESCRIPCIÓ FETA PER L'INFORMANT. Al voltant de l’arbre, en un radi de tres metres, no hi creix res.

***Diplotaxis erucoides***(L.) DC. (crucíferes)
BCN 129685

**NOMS POPULARS**

*Jaramago* (castellà) (2174)

Ravenissa (2178, 2210, 2219, 2229)

**USOS ALIMENTARIS**

**Flor i fulla**

Condiment

FONT 2174. DESCRIPCIÓ DE L'ÚS FETA PER L'INFORMANT. Té gust de mostassa. CONSUMICIÓ. Amanida. DESTINACIÓ. Alimentació humana.

**No consta**

No consta el tipus d'ingestió - No consta el mode de preparació

FONT 2161. DESCRIPCIÓ DE L'ÚS FETA PER L'INFORMANT. Per als ocells. DESTINACIÓ. Alimentació animal.

**Part aèria**

No consta el tipus d'ingestió - No consta el mode de preparació

FONT 2229. DESCRIPCIÓ DE L'ÚS FETA PER L'INFORMANT. Bona per als conills. DESTINACIÓ. Alimentació animal.

**ALTRES USOS**

**Planta viva *in situ***

Agrosilvopastoral

FONT 2178. DESCRIPCIÓ DE L'ÚS FETA PER L'INFORMANT. És font de fauna auxiliar. OBSERVACIONS. Associació de cultius i equilibri sistèmic.

***Equisetum arvense***L. (equisetàcies)
BCN 126546

**NOMS POPULARS**

Cua de cavall (2162, 2164, 2178, 2204, 2217, 2229, 2226)

Cua de rata (2163)

**USOS MEDICINALS**

**Part aèria**

Antihipertensor

FONT 2229. DESCRIPCIÓ DE L'ÚS FETA PER L'INFORMANT. Rebaixa la pressió sanguínia. FORMA FARMACÈUTICA I ÚS. Desconegut per l'informant / No consta. DESTINACIÓ. Medicina humana.

Antiinflamatori faringi

FONT 2204. FORMA FARMACÈUTICA I ÚS. Gargarisme (ús extern). MODE D'UTILITZACIÓ/POSOLOGIA. Per a guarir el mal de gola, calia bullir una mica d’herba, i, un xic tèbia, es gargaritzava quatre o cinc vegades al dia. DESTINACIÓ. Medicina humana.

Diürètic

FONTS 2162, 2217, 2226. DESCRIPCIÓ DE L'ÚS FETA PELS INFORMANTS. Diürètic (2226). FORMA FARMACÈUTICA I ÚS. Infusió (ús intern) (2226). DESTINACIÓ. Medicina humana.

Hipouricemiant

FONT 2204. DESCRIPCIÓ DE L'ÚS FETA PER L'INFORMANT. Per a combatre l’àcid úric. FORMA FARMACÈUTICA I ÚS. Infusió (ús intern). DESTINACIÓ. Medicina humana.

**ALTRES USOS**

**Part aèria**

Agrosilvopastoral

FONT 2164. DESCRIPCIÓ DE L'ÚS FETA PER L'INFORMANT. L'extracte líquid resultant de la fermentació de la planta [dit "purins"] és un bon adob foliar. També prevé l'aparició de fongs en l'agricultura. OBSERVACIONS. Adobs i fertilitzants naturals. Plaguicides naturals.

Cosmètic

FONT 2229. DESCRIPCIÓ DE L'ÚS FETA PER L'INFORMANT. Reforça les ungles i el cabell.

***Equisetum ramosissimum***Desf. subsp. ***ramosissimum***(equisetàcies)
BCN 130960

**NOMS POPULARS**

Cua de cavall (2210)

**USOS MEDICINALS**

**Part aèria**

Diürètic

FONT 2210. FORMA FARMACÈUTICA I ÚS. Desconegut per l'informant / No consta. DESTINACIÓ. Medicina humana.

***Equisetum telmateia***Ehrh. (equisetàcies)
BCN 128109

**NOMS POPULARS**

Cua de cavall (2187, 2191, 2219)

**USOS MEDICINALS**

**Part aèria**

Antiartròsic

FONT 2191. FORMA FARMACÈUTICA I ÚS. Infusió (ús intern). DESTINACIÓ. Medicina humana.

Diürètic

FONT 2219. DESCRIPCIÓ DE L'ÚS FETA PER L'INFORMANT. Per a fer orinar. FORMA FARMACÈUTICA I ÚS. Infusió (ús intern).

Protector renal

FONT 2187. DESCRIPCIÓ DE L'ÚS FETA PER L'INFORMANT. Va bé per al ronyó. FORMA FARMACÈUTICA I ÚS. Infusió (ús intern). DESTINACIÓ. Medicina humana.

Hematocatàrtic

FONT 2187. DESCRIPCIÓ DE L'ÚS FETA PER L'INFORMANT. És depuratiu. FORMA FARMACÈUTICA I ÚS. Infusió (ús intern). DESTINACIÓ. Medicina humana.

***Erica arborea***L. (ericàcies)
BCN 113593

**NOMS POPULARS**

Bruc (2217)

**ALTRES USOS**

**Tija amb fulles/branques**

Elaboració d'escombres

FONT 2217.

***Erica multiflora***L. (ericàcies)
BCN 129704

**NOMS POPULARS**

Bruc (2214)

**ALTRES USOS**

**Planta viva *in situ***

Agrosilvopastoral

FONT 2214. DESCRIPCIÓ DE L'ÚS FETA PER L'INFORMANT. Per a fer cercats. OBSERVACIONS. Elements paisatgístics.

**Tija**

Elaboració de pipes de fumar

FONT 2214.

**Tija amb fulles/branques**

Elaboració d'escombres

FONT 2214.

Obtenció de combustible: carbó

FONT 2214. DESCRIPCIÓ DE L'ÚS FETA PER L'INFORMANT. Per a fer carbó.

***Eriobotrya japonica***(Thunb.) Lindl. (rosàcies)
BCN 127882

**NOMS POPULARS**

Nespra (fruit) (2170, 2171, 2186)

Nesprer (2160, 2170, 2171, 2172, 2186, 2189, 2218)

Nespro (fruit) (2179, 2192, 2225)

**USOS MEDICINALS**

**Fulla**

Protector renal

FONT 2172. FORMA FARMACÈUTICA I ÚS. Infusió (ús intern). PREPARACIÓ. Es fa bullir aigua (un litre), s’afegeixen 10 fulles, es fa bullir 10 minuts i es deixa reposar cinc minuts. DESTINACIÓ. Medicina humana.

**USOS ALIMENTARIS**

**Fruit**

Ingestió de la part de la planta crua - Fresca (sense preparació)

FONTS 2170, 2171, 2179, 2186, 2192, 2218, 2225. DESCRIPCIÓ DE L'ÚS FETA PELS INFORMANTS. Comestible (2225). Se’n menja el fruit (2218). DESTINACIÓ. Alimentació humana (2170, 2171, 2179, 2186, 2192, 2218, 2225).

***Eruca vesicaria***(L.) Cav. subsp. ***sativa***(Mill.) Thell. in Hegi (crucíferes)
BCN 127875

**NOMS POPULARS**

*Rúcula* (castellà) (2200)

Ruca (2195)

**USOS ALIMENTARIS**

**Fulla**

Ingestió de la part de la planta crua - Fresca (sense preparació)

FONTS 2195, 2200. CONSUMICIÓ. Amanida. DESTINACIÓ. Alimentació humana.

***Eryngium campestre***L. (umbel·líferes)
BCN 125407

**NOMS POPULARS**

Panical (2210)

**USOS MEDICINALS**

**Arrel**

Per a trastorns del sistema musculoesquelètic

FONT 2210. FORMA FARMACÈUTICA I ÚS. Bany (ús extern). MODE D'UTILITZACIÓ/POSOLOGIA. Per a fer banys, bons per a les inflamacions musculars. DESTINACIÓ. Medicina humana.

**ALTRES USOS**

**Arrel**

Literatura oral popular: llegendes, gloses, contes, dites, refranys, poemes, cançons

FONT 2210. DESCRIPCIÓ DE L'ÚS FETA PER L'INFORMANT. “Arrel del panical, per a les cames del meu pare, que li feien tant de mal”.

***Eryngium maritimum***L. (umbel·líferes)
BCN 99733

**NOMS POPULARS**

Panical (2184)

**BARREGES AMB AQUEST TÀXON (vegeu catàleg de barreges)**

**USOS MEDICINALS**

**Arrel**

FONT 2184. Per al refredat.

***Eucalyptus globulus***Labill. (mirtàcies)
BCN 132996

**NOMS POPULARS**

Caliptus (2182, 2210)

Calitus (2209)

Eucaliptu (2217)

Eucaliptus (2185, 2186, 2192, 2204, 2217, 2228, 2229, 3932, 3934)

**USOS MEDICINALS**

**Flor i fulla**

Per al refredat

FONTS 2204, 2217. FORMA FARMACÈUTICA I ÚS. Aerosol (ús intern). DESCRIPCIÓ DE L'ÚS FETA PELS INFORMANTS. Per a fer bafs quan estàs refredat (2217). MODE D'UTILITZACIÓ/POSOLOGIA. Es posava una olla amb aigua bullent i s’hi ficava unes fulles i flors d’eucaliptus. Al cap d’una estona, el malalt es tapava amb una flassada gran, cap inclòs, com una petita tenda de campanya, amb l’olla també a dins. Aleshores, havia de respirar el baf que desprenia l’eucaliptus una bona estona (2204). PREPARACIÓ. Es feia una paperina de paper amb un forat petit a dalt; llavors la posaves al nas i aspiraves el bafs (2217). DESTINACIÓ. Medicina humana.

**Fruit**

Per a trastorns del sistema respiratori

FONT 2228. DESCRIPCIÓ DE L'ÚS FETA PER L'INFORMANT. Bafs [dit “bafos”] per a malalties dels pulmons. FORMA FARMACÈUTICA I ÚS. Aerosol (ús intern). DESTINACIÓ. Medicina humana.

Per al refredat

FONTS 2210, 2217. FORMA FARMACÈUTICA I ÚS. Aerosol (ús intern). MODE D'UTILITZACIÓ/POSOLOGIA. Per a fer bafs quan estem constipats (2210). DESTINACIÓ. Medicina humana.

**Fulla**

Antitussigen

FONT 2229. DESCRIPCIÓ DE L'ÚS FETA PER L'INFORMANT. Se’n fa un remei per a la tos. FORMA FARMACÈUTICA I ÚS. Desconegut per l'informant / No consta. DESTINACIÓ. Medicina humana.

Per a trastorns del sistema respiratori

FONTS 2228, 3932. DESCRIPCIÓ DE L'ÚS FETA PELS INFORMANTS. Va bé per a les afeccions a les vies respiratòries (3932). Bafs [dit “bafos”] per a malalties dels pulmons (2228). FORMA FARMACÈUTICA I ÚS. Aerosol (ús intern). MODE D'UTILITZACIÓ/POSOLOGIA. Cal fer-ne bafs (3932). DESTINACIÓ. Medicina humana.

Per al refredat

FONTS 2182, 2192, 2210, 2229, 3934. DESCRIPCIÓ DE L'ÚS FETA PELS INFORMANTS. En fan bafs [dit "bafos"] de caliptu amb les fulles per als constipats (2182). Els bafs d’eucaliptus són bons per als constipats (2229). Per a fer bafs quan estàs constipat (3934). FORMA FARMACÈUTICA I ÚS. Aerosol (ús intern). MODE D'UTILITZACIÓ/POSOLOGIA. Per a fer bafs quan estem constipats (2210). DESTINACIÓ. Medicina humana.

**ALTRES USOS**

**Flor i fulla**

Elaboració de rams

FONT 2186. DESCRIPCIÓ DE L'ÚS FETA PER L'INFORMANT. Es fa servir per a decoració. OBSERVACIONS. Ornamental.

**Fruit sec**

Ambientador

FONT 2209. DESCRIPCIÓ DE L'ÚS FETA PER L'INFORMANT. En un plat de llauna, amb aigua calenta, es posaven alguns fruits per a escalfar i perfumar la llar.

**Fulla**

Ambientador

FONT 2209. DESCRIPCIÓ DE L'ÚS FETA PER L'INFORMANT. En un plat de llauna, amb aigua calenta, es posaven algunes fulles per a escalfar i perfumar la llar.

**BARREGES AMB AQUEST TÀXON (vegeu catàleg de barreges)**

**USOS MEDICINALS**

**Fulla**

FONT 2229. Remei per a la tos.

***Euphorbia characias***L. subsp. ***characias***(euforbiàcies)
BCN 129701

**NOMS POPULARS**

*Lechetrezna* (castellà) (2213, 2214)

**ALTRES USOS**

**Tija**

Elaboració de fibres

FONT 2213. DESCRIPCIÓ DE L'ÚS FETA PER L'INFORMANT. Se’n poden treure les fibres per a fer corda.

**ACCIONS NOCIVES O TÒXIQUES**

**Làtex**

Irritant

FONT 2213, 2214. VIA D'INTOXICACIÓ. Externa. DESCRIPCIÓ DE L'ÚS FETA PER L'INFORMANT. És tòxica (2213, 2214).

***Euphorbia lathyris***L. (euforbiàcies)
BCN 132994

**NOMS POPULARS**

*Cagamucho* (castellà) (2191)

Cagamuja (2191)

**USOS MEDICINALS**

**Llavor**

Laxant

FONT 2191. FORMA FARMACÈUTICA I ÚS. Macerat en oli (ús extern). MODE D'UTILITZACIÓ/POSOLOGIA. Es posa en oli 15-20 llavors seques i després s’unta l’anus amb l’ungüent. DESTINACIÓ. Medicina humana.

***Ficus carica***L. (moràcies)
BCN 127891

**NOMS POPULARS**

Figa (infructescència) (2166, 2179, 2180, 2182, 2187, 2190, 2191, 2192, 2204, 2211, 2212, 2218, 3934, 3936)

Figuera (2166, 2179, 2182, 2184, 2189, 2212, 2217, 2218, 3934, 3946)

Secallona (infructescència dessecada) (2212)

**USOS MEDICINALS**

**Infructescència**

Expectorant

FONT 2166. FORMA FARMACÈUTICA I ÚS. Decocció (ús intern). DESTINACIÓ. Medicina humana.

**Làtex**

Antiberrugós

FONTS 2204, 2217. DESCRIPCIÓ DE L'ÚS FETA PER L'INFORMANT. La llet era bona per a les berrugues. S’aplicava directament (2217) FORMA FARMACÈUTICA I ÚS. Sense forma farmacèutica (ús directe) (ús extern). MODE D'UTILITZACIÓ/POSOLOGIA. Per a guarir o treure berrugues ens fèiem aplicacions de figues verdes. S’havia d’aplicar, directament, damunt les berrugues la llet de les figues verdes, uns quants cops al dia (2204). DESTINACIÓ. Medicina humana (2204, 2217).

**USOS ALIMENTARIS**

**Infructescència**

Ingestió de la part de la planta crua - Conservada dessecada a l'aire

FONT 2212. DESCRIPCIÓ DE L'ÚS FETA PER L'INFORMANT. Es mengen seques. Se’ls diu “secallones”. DESTINACIÓ. Alimentació humana.

Ingestió de la part de la planta crua - Fresca (sense preparació)

FONTS 2179, 2180, 2182, 2190, 2191, 2192, 2218, 3934, 3936. DESCRIPCIÓ DE L'ÚS FETA PELS INFORMANTS. Se’n mengen els fruits (2218). DESTINACIÓ. Alimentació humana (2179, 2180, 2182, 2190, 2191, 2192, 2218, 3934, 3936).

Ingestió de la part de la planta cuita - Cuita en sucre

FONTS 2187, 2192, 2211, 3934. DESCRIPCIÓ DE L'ÚS FETA PELS INFORMANTS. Confitura (2187). Melmelada (2192). Per a fer confitura (3934). DESTINACIÓ. Alimentació humana (2187, 2192, 2211, 3934).

**ALTRES USOS**

**Tija**

Construcció de vehicles amb rodes

FONT 3946. DESCRIPCIÓ DE L'ÚS FETA PER L'INFORMANT. La seva fusta és bona per a fer els frens dels carros.

**ALTRES OBSERVACIONS**

FONT 2218. DESCRIPCIÓ FETA PER L'INFORMANT. No hi creix res al voltant.

***Foeniculum vulgare***Mill. subsp. ***piperitum***(Ucria) Cout. (umbel·líferes)
BCN 125404

**NOMS POPULARS**

Fonoll (2168, 2169, 2174, 2176, 2177, 2178, 2184, 2192, 2197, 2204, 2209, 2210, 2219, 2222, 2217, 2224, , 2225, 2228, 2232, 3934, 3946)

**USOS MEDICINALS**

**Fruit**

Carminatiu

FONTS 2174, 2178, 2228, 2232. DESCRIPCIÓ DE L'ÚS FETA PELS INFORMANTS. Es posen els fruits als potatges per tal que no donin gasos (2174). Va bé contra els gasos (2228). FORMA FARMACÈUTICA I ÚS. Sense forma farmacèutica (ús directe) (ús intern) (2174, 2228). Infusió (ús intern) (2178). Desconegut per l'informant / No consta (2232). DESTINACIÓ. Medicina humana.

Digestiu

FONTS 2178, 2210, 2217, 2228, 3934. FORMA FARMACÈUTICA I ÚS. Infusió (ús intern). DESTINACIÓ. Medicina humana.

Diürètic

FONTS 2184, 2209. FORMA FARMACÈUTICA I ÚS. Infusió (ús intern). DESTINACIÓ. Medicina humana.

**No consta**

Antiespasmòdic

FONTS 2176, 2177. FORMA FARMACÈUTICA I ÚS. Desconegut per l'informant / No consta. DESTINACIÓ. Medicina humana.

**USOS ALIMENTARIS**

**Bulb**

Ingestió de la part de la planta cuita - Cuita en aigua

FONTS 2168, 2169. DESTINACIÓ. Alimentació humana.

No consta el tipus d'ingestió - No consta el mode de preparació

FONT 2197. DESTINACIÓ. Alimentació humana.

**Fulla jove**

Ingestió de la part de la planta crua - Fresca (sense preparació)

FONTS 2204, 2228. DESCRIPCIÓ DE L'ÚS FETA PER L'INFORMANT. Amanida (2228). DESTINACIÓ. Alimentació humana.

**Summitat florífera**

Condiment

FONTS 2219, 2225. DESCRIPCIÓ DE L'ÚS FETA PELS INFORMANTS. Per a l’adobat de les olives (2225). OBSERVACIONS. Adobat d'olives (2219, 2225). DESTINACIÓ. Alimentació humana (2219, 2225).

**Tija amb fulles/branques**

Condiment

FONTS 2184, 2192, 2210, 2217, 2222, 2224, 2228, 3946. DESCRIPCIÓ DE L'ÚS FETA PELS INFORMANTS. Adobat d'olives (2184, 2192, 2210). Per a fer els cargols. Es bullien amb unes branques de fonoll (2192). Per als cargols (2184, 2222, 2224, 3946). Per a cuinar els cargols (2217). Per a l’adobat de les olives (2224). Per a l’arròs, els cigrons i altres llegums (se’n posa una branqueta a l’aigua de bull) (2228). DESTINACIÓ. Alimentació humana (2184, 2192, 2210, 2217, 2222, 2224, 2228, 3946).

**ALTRES USOS**

**Summitat florífera**

Ajuda a la llar

FONT 2232. DESCRIPCIÓ DE L'ÚS FETA PER L'INFORMANT. Per a netejar els cargols.

**BARREGES AMB AQUEST TÀXON (vegeu catàleg de barreges)**

**USOS ALIMENTARIS**

**Tija amb fulles/branques**

FONT 2192. Olives arreglades.

***Foeniculum vulgare***Mill. subsp. ***vulgare***(umbel·líferes)
BCN 129017

**NOMS POPULARS**

Fonoll (2178, 2193, 2194, 2196)

**USOS ALIMENTARIS**

**Bulb**

Ingestió de la part de la planta crua - Fresca (sense preparació)

FONT 2178. DESCRIPCIÓ DE L'ÚS FETA PER L'INFORMANT. El bulb ratllat en amanides.

Ingestió de la part de la planta cuita - Cuita en aigua

FONT 2178. DESTINACIÓ. Alimentació humana.

No consta el tipus d'ingestió - No consta el mode de preparació

FONTS 2193, 2194, 2196. DESTINACIÓ. Alimentació humana.

***Fragaria ×ananassa***Duchesne (rosàcies)
BCN 130936

**NOMS POPULARS**

*Fresó* (fruit) (2202, 2204, 2208)

Maduixa (fruit) (2167, 2199, 2205)

Maduixot (fruit) (2200, 2201, 2206)

**USOS ALIMENTARIS**

**Fruit**

Ingestió de la part de la planta crua - Fresca (sense preparació)

FONTS 2167, 2199, 2200, 2201, 2202, 2205, 2206. DESCRIPCIÓ DE L'ÚS FETA PELS INFORMANTS. Se les mengen senceres (2167). Se'n menja (2199, 2202, 2205, 2206). Comestible (2200). DESTINACIÓ. Alimentació humana (2167, 2199, 2200, 2201, 2202, 2205, 2206).

Ingestió de la part de la planta cuita - Cuita en sucre

FONT 2208. DESCRIPCIÓ DE L'ÚS FETA PER L'INFORMANT. Se'n fan melmelades. DESTINACIÓ. Alimentació humana.

***Fragaria vesca***L. (rosàcies)
BCN 29697

**NOMS POPULARS**

Maduixa de bosc (fruit) (2189)

**USOS ALIMENTARIS**

**Fruit**

Ingestió de la part de la planta crua - Fresca (sense preparació)

FONT 2189. DESCRIPCIÓ DE L'ÚS FETA PER L'INFORMANT. Per al consum propi. Tothom en tenia a les vinyes. Algunes famílies s’havien especialitzat en el seu conreu i en la venda a l’engròs. DESTINACIÓ. Alimentació humana.

***Galium aparine***L. (rubiàcies)
BCN 126550

**NOMS POPULARS**

Gàlium (2160)

***Genista scorpius***(L.) DC. in Lam. et DC. subsp. ***scorpius***(papilionàcies)
BCN 156592

**NOMS POPULARS**

Argelaga (2192)

**ALTRES USOS**

**Tija amb fulles/branques**

Obtenció de combustible: llenya

FONT 2192. DESCRIPCIÓ DE L'ÚS FETA PER L'INFORMANT. En feien feixines o gavelles, que servien per a cremar als forns.

***Globularia alypum***L. (plantaginàcies)
BCN 132993

**NOMS POPULARS**

Foixarda (2191)

**USOS MEDICINALS**

**No consta**

Antihipertensor

FONT 2191. FORMA FARMACÈUTICA I ÚS. Desconegut per l'informant / No consta. DESTINACIÓ. Medicina humana.

***Glycyrrhiza glabra***L. (papilionàcies)
BCN 130935

**NOMS POPULARS**

Regalèssia (2182, 2184, 2212, 2222, 2228)

**USOS MEDICINALS**

**Arrel**

Digestiu

FONTS 2182, 2222. FORMA FARMACÈUTICA I ÚS. Decocció (ús intern) (2182). Sense forma farmacèutica (ús directe) (ús intern) (2222). MODE D'UTILITZACIÓ/POSOLOGIA. Després de dinar o de sopar (2182). Es llepa (2222). DESTINACIÓ. Medicina humana (2182, 2222).

Salutífer

FONT 2228. DESCRIPCIÓ DE L'ÚS FETA PER L'INFORMANT. Molt energètic. FORMA FARMACÈUTICA I ÚS. Decocció (ús intern). PREPARACIÓ. Es bull cinc minuts. DESTINACIÓ. Medicina humana.

**USOS ALIMENTARIS**

**Arrel**

Ingestió de la part de la planta crua - Fresca (sense preparació)

FONT 2212. DESCRIPCIÓ DE L'ÚS FETA PER L'INFORMANT. Se'n xuclava el suc. DESTINACIÓ. Alimentació humana.

**ALTRES USOS**

**Arrel**

Altres informacions

FONT 2222. DESCRIPCIÓ DE L'ÚS FETA PER L'INFORMANT. Va bé per a deixar de fumar, perquè substitueix la cigarreta.

**BARREGES AMB AQUEST TÀXON (vegeu catàleg de barreges)**

**USOS MEDICINALS**

**Arrel**

FONT 2184. Per al refredat.

***Hedera helix***L. (araliàcies)
BCN 127880

**NOMS POPULARS**

Heura (2188)

***Helianthus tuberosus***L. (compostes)
BCN 126581

**NOMS POPULARS**

Nyàmera (2163)

Tupinamba (2163)

**USOS ALIMENTARIS**

**Tubercle**

Ingestió de la part de la planta cuita - Cuita en aigua

FONT 2163. DESCRIPCIÓ DE L'ÚS FETA PER L'INFORMANT. Se'n fan cremes. DESTINACIÓ. Alimentació humana.

***Helichrysum stoechas***(L.) Moench (compostes)
BCN 127877

**NOMS POPULARS**

Flor de Sant Joan (2187, 2189, 2190)

**USOS ALIMENTARIS**

**Fulla**

Condiment

FONT 2189. DESCRIPCIÓ DE L'ÚS FETA PER L'INFORMANT. Com a condiment, pel seu aroma similar a la cúrcuma o al curri. DESTINACIÓ. Alimentació humana.

Ingestió de la part de la planta cuita - Cuita en aigua

FONT 2189. DESCRIPCIÓ DE L'ÚS FETA PER L'INFORMANT. Com a beguda calenta. DESTINACIÓ. Alimentació humana.

**ALTRES USOS**

**Part aèria florida**

Creences i pràctiques magicoreligioses

FONT 2187. DESCRIPCIÓ DE L'ÚS FETA PER L'INFORMANT. Es collia i es posava dins de casa perquè donava sort.

Elaboració de rams

FONT 2190. DESCRIPCIÓ DE L'ÚS FETA PER L'INFORMANT. Es posava a les processons.

***Hordeum murinum***L. (gramínies)
BCN 130958

**NOMS POPULARS**

Blat del dimoni (2210)

***Hordeum vulgare***L. (gramínies)
BCN 156580

**NOMS POPULARS**

Malta (trituració del gra) (2210)

Ordi (2204, 2210, 2212, 2217)

Ordi Aurora (raça) (2204)

Ordi cabot (raça) (2204)

Ordi d'Igualada (raça) (2204)

Ordi de barbes llises (raça) (2204)

Ordi de sis ratlles (raça) (2204)

Ordi paner I (raça) (2204)

Ordi Samuntà (raça) (2204)

Ordi de Sant Isidre (raça) (2204)

**USOS ALIMENTARIS**

**Llavor**

No consta el tipus d'ingestió - No consta el mode de preparació

FONTS 2204, 2212, 2217. DESCRIPCIÓ DE L'ÚS FETA PER L'INFORMANT. Els grans d’ordi servien per a l’engreix d’un o dos porcs i per a aquells animals domèstics del corral (2204). Se’n cultivava pel gra. Conreu ja desaparegut (2212). En donaven als porcs (2217). DESTINACIÓ. Alimentació animal (2204, 2217). Alimentació humana (2212).

**Part aèria**

Ingestió de la part de la planta crua - Conservada dessecada a l'aire

FONT 2204. DESCRIPCIÓ DE L'ÚS FETA PER L'INFORMANT. La palla era aliment per als cavalls. DESTINACIÓ. Alimentació animal.

Preparació de begudes - Beguda preparada amb aigua

FONT 2210. DESCRIPCIÓ DE L'ÚS FETA PER L'INFORMANT. Torrat i triturat se’n feia malta, que era un substitut del cafè. DESTINACIÓ. Alimentació humana.

**ALTRES USOS**

**Tija**

Agrosilvopastoral

FONT 2204. DESCRIPCIÓ DE L'ÚS FETA PER L'INFORMANT. Era interessant sembrar gra per a obtenir-ne palla, que servia per a fer el jaç o llit a l’estable; després, mesclat amb els excrements i els pixums dels animals, s’obtenia l’adob biològic o fems per a l’hort de la casa. OBSERVACIONS. Adobs i fertilitzants naturals.

***Hypericum perforatum***L. (gutíferes)
BCN 96760

**NOMS POPULARS**

Flor de Sant Joan (2222)

Herba de Sant Joan (2204)

Hipèric (2222)

Pericó (2228)

**USOS MEDICINALS**

**Flor**

Antidepressiu

FONT 2228. FORMA FARMACÈUTICA I ÚS. Desconegut per l'informant / No consta. DESTINACIÓ. Medicina humana.

Antiinflamatori / Antiàlgic / Antiequimòtic

FONT 2228. DESCRIPCIÓ DE L'ÚS FETA PER L'INFORMANT. Útil contra els cops i el dolor. FORMA FARMACÈUTICA I ÚS. Embrocació (ús extern). DESTINACIÓ. Medicina humana.

Hipouricemiant

FONT 2204. DESCRIPCIÓ DE L'ÚS FETA PER L'INFORMANT. Com a prevenció del mal de gota. FORMA FARMACÈUTICA I ÚS. Infusió (ús intern). DESTINACIÓ. Medicina humana.

Per a les fissures cutànies

FONT 2204. DESCRIPCIÓ DE L'ÚS FETA PER L'INFORMANT. Calia collir-la durant la revetlla, a fi que fos plenament guaridora. Se’n feia servir per a molts tipus de ferides i nafres. FORMA FARMACÈUTICA I ÚS. Desconegut per l'informant / No consta. DESTINACIÓ. Medicina humana.

Per a trastorns de la pell o del teixit subcutani

FONT 2222. DESCRIPCIÓ DE L'ÚS FETA PER L'INFORMANT. Es collia la flor per Sant Joan. Es ficava en oli 40 dies a sol i serena. Un cop macerada es colava i s’utilitzava l’oli per a cremades, inflamacions i problemes de pell. FORMA FARMACÈUTICA I ÚS. Cataplasma (ús extern). DESTINACIÓ. Medicina humana.

Per a trastorns del sistema digestiu

FONT 2204. DESCRIPCIÓ DE L'ÚS FETA PER L'INFORMANT. Hi havia qui les prenia com a infusió per als trastorns intestinals. FORMA FARMACÈUTICA I ÚS. Infusió (ús intern). DESTINACIÓ. Medicina humana.

**BARREGES AMB AQUEST TÀXON (vegeu catàleg de barreges)**

**USOS MEDICIALS**

**Flor**

FONT 2228. Oli de romaní, ortiga i pericó.

***Ilex aquifolium***L. (aquifoliàcies)
BCN 29876

**NOMS POPULARS**

Boix grèvol (3952)

**USOS MEDICINALS**

**No consta**

Antipirètic (per a febre periòdica)

FONT 3952. FORMA FARMACÈUTICA I ÚS. Desconegut per l'informant / No consta. DESTINACIÓ. Medicina humana.

Diaforètic

FONT 3952. DESCRIPCIÓ DE L'ÚS FETA PER L'INFORMANT. Sudorífic. FORMA FARMACÈUTICA I ÚS. Desconegut per l'informant / No consta. DESTINACIÓ. Medicina humana.

***Ipomoea batatas***Poir. (convolvulàcies)
BCN-E-197

**NOMS POPULARS**

Moniato (2194, 2199, 2204, 3700, 2221, 2218)

**USOS ALIMENTARIS**

**Tubercle**

Ingestió de la part de la planta cuita - Cuita sense vehicle

FONTS 2204, 2218, 3700. DESCRIPCIÓ DE L'ÚS FETA PELS INFORMANTS. Escalivats a la cendra (2204). Es ficaven sota el foc durant una nit per a coure’ls (2218). CONSUMICIÓ. DESTINACIÓ. Alimentació humana (2204, 2218, 3700).

Ingestió de la part de la planta cuita - Cuita en oli

FONT 3700. DESCRIPCIÓ DE L'ÚS FETA PER L'INFORMANT. Fregit. DESTINACIÓ. Alimentació humana.

No consta el tipus d'ingestió - No consta el mode de preparació

FONTS 2194, 2199, 2218, 2221. DESCRIPCIÓ DE L'ÚS FETA PELS INFORMANTS. Es bullien i es donaven de menjar al bestiar (2218). DESTINACIÓ. Alimentació humana (2194, 2199, 2221). Alimentació animal (2218).

***Jasonia saxatilis***(Lam.) Guss. (compostes)
BCN 125409

**NOMS POPULARS**

Te de roca (2209, 2210, 2222)

**USOS MEDICINALS**

**Fulla**

Digestiu

FONT 2222. FORMA FARMACÈUTICA I ÚS. Infusió (ús intern). DESTINACIÓ. Medicina humana.

Estomacal

FONT 2210. FORMA FARMACÈUTICA I ÚS. Infusió (ús intern). DESTINACIÓ. Medicina humana.

**Summitat florífera**

Estomacal

FONT 2209. FORMA FARMACÈUTICA I ÚS. Infusió (ús intern). DESTINACIÓ. Medicina humana.

Per al refredat

FONT 2209. FORMA FARMACÈUTICA I ÚS. Infusió (ús intern). DESTINACIÓ. Medicina humana.

***Juglans regia***L. (juglandàcies)
BCN 126562

**NOMS POPULARS**

*Nogal* (castellà) (2229)

Noguera (2167, 2192, 3936, 2228)

**USOS MEDICINALS**

**Fulla**

Antiinflamatori / Antiàlgic / Antiequimòtic

FONT 2228. DESCRIPCIÓ DE L'ÚS FETA PER L'INFORMANT. Antiinflamatori. FORMA FARMACÈUTICA I ÚS. Desconegut per l'informant / No consta. DESTINACIÓ. Medicina humana.

**USOS ALIMENTARIS**

**Llavor**

No consta el tipus d'ingestió - No consta el mode de preparació

FONT 3936. DESTINACIÓ. Alimentació humana.

**ALTRES USOS**

**Fulla**

Ajuda a la llar

FONTS 2192. 2229. DESCRIPCIÓ DE L'ÚS FETA PER L'INFORMANT. S’utilitzaven les fulles de noguera per netejar les botes de vi (2192). Les fulles bullides servien per a treure l’agre de la bota de vi (2229).

***Juncus acutus***L. (juncàcies)
BCN 56530

**NOMS POPULARS**Jonc (2185, 2190, 2204)

**ALTRES USOS**

**Fulla jove**

Agrosilvopastoral

FONTS 2160, 2185, 2190, 2204. DESCRIPCIÓ DE L'ÚS FETA PELS INFORMANTS. Fins els anys 1990 anaven als Aiguamolls de l’Empordà a buscar joncs per a lligar escaroles i enciams. N'agafen dels més prims, que es vinclen sense trencar-se, no dels més gruixuts i buits per dins, que es trenquen. N'havien agafat anteriorment al Delta del Llobregat, però l’aviació (l’aeroport) va prohibir el pas a les jonqueres. Parlen de “passar jonc” per a referir-se a l’acte de lligar escaroles i enciams, activitat que acostumaven a fer les dones (2160). S’hi lligaven els enciams. Tallaven el jonc, el posaven estès al camp perquè es pansís i fos més flexible. Hi havia gent especialitzada que es dedicava a això (2185). Per a fer lligalls per a lligar enciams i escaroles. Anaven a les Filipines, perquè hi eren més prims i forts (2190). Per a lligar escaroles (2204). OBSERVACIONS. Elaboració d'estris d'ús hortícola/agrícola (2160, 2185, 2190, 2204).

***Lactarius deliciosus***(L.) Gray (russulàcies)
BCN-E-600

**NOMS POPULARS**

Rovelló (2192)

**USOS ALIMENTARIS**

**Part aèria**

Ingestió de la part de la planta cuita - Cuita en aigua

FONT 2192. DESCRIPCIÓ DE L'ÚS FETA PER L'INFORMANT. Per a fer conserva. DESTINACIÓ. Alimentació humana.

***Lactuca sativa***L. (compostes)
BCN 46842

**NOMS POPULARS**

Cabdell (2193)

Enciam (2160, 2168, 2169, 2178, 2182, 2192, 2193, 2198, 2204, 2210, 3700, 2221, 2212, 2216, 2220, 2217, 3946, 2218, 2226, 2228)

Enciam de fulla fina (raça) (2202, 2203)

Enciam del sucre (raça) (2226)

Enciam escaroler (raça) (2212)

Enciam francès (raça) (2205)

Enciam fulla de roure (raça) (2164, 2178, 2193, 2194, 2195, 2196, 2200, 2201, 2203, 2205)

Enciam fulla de roure verd (raça) (2195, 2201)

Enciam iceberg (raça) (2201, 2206, 3700)

Enciam Lollo rosso (raça) (2193)

Enciam Lollo verd (raça) (2193)

Enciam llarg (raça) (2178, 2193, 2199, 2200, 2201, 2202, 2226)

Enciam meravella (raça) (2164, 2196, 2198, 2200, 2202)

Enciam romà (raça) (2185, 2186, 3700, 2226)

Enciam Trocadero (raça) (2164, 2182, 2185, 2186, 2195, 2200, 2205, 3700)

*Lechuga* (castellà) (2173, 2174)

**USOS MEDICINALS**

**Fulla**

Hepatoprotector

FONT 2178. FORMA FARMACÈUTICA I ÚS. Sense forma farmacèutica (ús directe) (ús intern). DESTINACIÓ. Medicina humana.

Sedant

FONTS 2173, 2174, 3700. DESCRIPCIÓ DE L'ÚS FETA PELS INFORMANTS. A la nit ajuda a dormir (2173, 2174). FORMA FARMACÈUTICA I ÚS. Sense forma farmacèutica (ús directe) (ús intern). MODE D'UTILITZACIÓ/POSOLOGIA. Té propietats sedants. Menjada crua a la nit, ben amanida (3700). DESTINACIÓ. Medicina humana.

**USOS ALIMENTARIS**

**Fulla**

Ingestió de la part de la planta crua - Fresca (sense preparació)

FONTS 2160, 2164, 2168, 2169, 2173, 2174, 2178, 2182, 2185, 2186, 2193, 2194, 2195, 2198, 2200, 2202, 2203, 2206, 2210, 2212, 2216, 2217, 2220, 2221, 3700. CONSUMICIÓ. Amanida (2160, 2164, 2168, 2169, 2174, 2182, 2185, 2186, 2195, 2210, 3700). DESTINACIÓ. Alimentació humana (2160, 2164, 2168, 2169, 2173, 2174, 2178, 2182, 2185, 2186, 2193, 2194, 2195, 2198, 2200, 2202, 2203, 2206, 2210, 2212, 2216, 2217, 2220, 2221, 3700).

No consta el tipus d'ingestió - No consta el mode de preparació

FONTS 2196, 2199, 2201, 2205, 2218, 2226, 3946. DESCRIPCIÓ DE L'ÚS FETA PELS INFORMANTS. Se’n menja (2218). Comestible (2226). DESTINACIÓ. Alimentació humana (2196, 2199, 2201, 2205, 2218, 2226, 3946).

**Fulla vella**

Ingestió de la part de la planta crua - Fresca (sense preparació)

FONT 2186. DESCRIPCIÓ DE L'ÚS FETA PER L'INFORMANT. Per a les vaques. DESTINACIÓ. Alimentació animal.

**ALTRES OBSERVACIONS**

FONTS 2182, 2185, 2186, 2212, 3700. DESCRIPCIÓ FETA PELS INFORMANTS. Es plantava en lluna vella (3700). Els embarcaven (exportaven en camió o tren, sobretot, a França) (2182). En feien enciam Trocadero [raça] per a embarcar-lo (exportar-lo). Abans el Trocadero [raça] no se'n menjava aquí. Aquí el que es menjava era l'enciam romà [raça] (2185). El Trocadero [raça] no és tan gustós com l'enciam romà [raça] (2186). [Relatiu a l'enciam escaroler] És un cultiu ja desaparegut; sobretot, s'exportava a Navarra (2212).

**BARREGES AMB AQUEST TÀXON (vegeu catàleg de barreges)**

**USOS ALIMENTARIS**

**Fulla**

FONT 2228. Caldo depuratiu.

***Lagenaria siceraria* (Molina) Standley** (cucurbitàcies)

BCN 50212

**NOMS POPULARS**

Carbassa (fruit) (2189)

Carbassa del pelegrí (3946)

**Fruit**

Ajuda a la llar

FONTS 2189, 3946. DESCRIPCIÓ DE L'ÚS FETA PER L'INFORMANT. Un cop seques, es buiden per a fer recipients (3946). Es buidaven i es feien servir com a cantimplores (2189).

***Laurus nobilis***L. (lauràcies)
BCN 126566

**NOMS POPULARS**

Llaurer (2167, 2182, 2183, 2184, 2186, 2188, 2189, 2192, 2196, 2209, 2210, 2221, 2211, 2216, 2217, 2224, 2232)

Llorer (2197, 2206, 2212, 3952, 2218, 2227, 2226)

**USOS MEDICINALS**

**Fruit**

Antireumàtic

FONT 3952. DESCRIPCIÓ DE L'ÚS FETA PER L'INFORMANT. Es matxuquen en un morter baies de llorer madures. Es posen a bullir cobertes d'aigua durant 5-10 minuts. S'espremen després mitjançant un drap, es deixa refredar el líquid i es recull el greix que flota. S'aplica friccionant la zona afectada. FORMA FARMACÈUTICA I ÚS. Bàlsam (ús extern). DESTINACIÓ. Medicina humana.

Digestiu

FONT 3952. DESCRIPCIÓ DE L'ÚS FETA PER L'INFORMANT. En infusió, es prepara amb 20 o 30 grams de fulles per litre d'aigua, s'hi pot afegir uns quants fruits madurs, es pren una tassa després de dinar. FORMA FARMACÈUTICA I ÚS. Infusió (ús intern). DESTINACIÓ. Medicina humana.

Orexigen

FONT 3952. FORMA FARMACÈUTICA I ÚS. Infusió (ús intern). PREPARACIÓ. En infusió, es prepara amb 20 o 30 grams de fulles per litre d'aigua, s'hi pot afegir uns quants fruits madurs, se’n pren una tassa 10 minuts abans de cada menjar. DESTINACIÓ. Medicina humana.

**Fulla**

Antiinflamatori

FONT 3952. FORMA FARMACÈUTICA I ÚS. Macerat en oli (ús extern). PREPARACIÓ. Es prepara deixant macerar durant 10 dies al sol 30 grams de fulles en un litre d'oli d'oliva. S'aplica sobre la part dolorida. Serveix també per a espantar insectes paràsits. DESTINACIÓ. Medicina humana.

Antireumàtic

FONT 3952. DESCRIPCIÓ DE L'ÚS FETA PER L'INFORMANT. Antireumàtic i antiinflamatori. Molt efectiu aplicant oli de llorer externament. FORMA FARMACÈUTICA I ÚS. Macerat en oli (ús extern). DESTINACIÓ. Medicina humana.

**Fulla jove**

Antisèptic extern

FONT 2182. DESCRIPCIÓ DE L'ÚS FETA PER L'INFORMANT. Les embenaven a sobre de la pelada per a tirar el pus. FORMA FARMACÈUTICA I ÚS. Cataplasma (ús extern). DESTINACIÓ. Medicina humana.

Per a la distensió muscular

Per a la distensió muscular

FONT 2183. FORMA FARMACÈUTICA I ÚS. Infusió (ús intern). MODE D'UTILITZACIÓ/POSOLOGIA. Contra el flat. Es posen de tres a cinc cullerades a bullir i t'ho beus. DESTINACIÓ. Medicina humana.

**USOS ALIMENTARIS**

**Fulla**

Condiment

FONTS 2167, 2184, 2196, 2197, 2206, 2209, 2210, 2211, 2212, 2216, 2218, 2221, 2226. DESCRIPCIÓ DE L'ÚS FETA PELS INFORMANTS. Per a condimentar la pasta (2211). Per a fer rostits i estofats (2184). Per a guisats (2206). Sec, per a cuinar (2216). Com a herba per a cuinar (2218). S’utilitza per a cuinar (2221). Les fulles s’utilitzen per a molts plats de cuina, com estofats de carn (2226). DESTINACIÓ. Alimentació humana (2167, 2184, 2196, 2197, 2206, 2209, 2210, 2211, 2212, 2216, 2218, 2221, 2226).

**ALTRES USOS**

**Fulla**

Elaboració de rams

FONT 2186. DESCRIPCIÓ DE L'ÚS FETA PER L'INFORMANT. Feien corones commemoratives i les venien a la floristeria.

Repel·lent

FONT 3952. DESCRIPCIÓ DE L'ÚS FETA PER L'INFORMANT. Es prepara deixant macerar durant 10 dies al sol 30 grams de fulles en un litre d'oli d'oliva. Serveix també per a espantar insectes paràsits.

**Tija amb fulles/branques**

Creences i pràctiques magicoreligioses

FONTS 2189, 2232, 3700. DESCRIPCIÓ DE L'ÚS FETA PELS INFORMANTS. S’utilitzava durant el Diumenge de Rams per a aquells que no es podien permetre una palma o palmó. No acostumaven a guarnir-se (3700). La setmana d’abans del Diumenge de Rams sortien de Sant Climent camions plens de feixos de llaurer per vendre a l’engròs i a la menuda. El volien per a anar a beneir (2189). El Diumenge de Rams és tradició beneir a l'església rams de llorer, que després es guarden a casa com a protecció contra tot mal (2232).

Elaboració de rams

FONTS 2188, 2192, 2210. DESCRIPCIÓ DE L'ÚS FETA PELS INFORMANTS. En collien i en venien per Rams (2188). Ornamental. Es venia a la menuda per Rams (2192). Ornamental (2210).

**ACCIONS NOCIVES O TÒXIQUES**

**Fulla jove**

FONT 2217. DESCRIPCIÓ DE L'ACCIÓ NOCIVA O TÒXICA FETA PER L'INFORMANT. La fulla tendra és tòxica. VIA D'INTOXICACIÓ. Interna.

**BARREGES AMB AQUEST TÀXON (vegeu catàleg de barreges)**

**USOS ALIMENTARIS**

**Fulla**

FONT 2212. Peus de porc amb naps.

***Lavandula dentata***L. (labiades)
BCN 126557

**NOMS POPULARS**

Espígol (2189, 2204, 2209, 2210, 2211, 2232, 3952, 2225, 2226)

Lavanda (2167, 2187, 2189, 3933)

**USOS MEDICINALS**

**Part aèria**

Antiinflamatori / Antiàlgic muscular

FONT 3952. DESCRIPCIÓ DE L'ÚS FETA PER L'INFORMANT. Després d'un exercici físic intens, un bany amb aigua de lavanda activa la circulació i disminueix el dolor. El dolor també es redueix amb unes friccions amb un drap de llana untat amb oli d’espígol. FORMA FARMACÈUTICA I ÚS. Bany (ús extern). Liniment (ús extern). DESTINACIÓ. Medicina humana.

Antipiròtic

FONT 3952. DESCRIPCIÓ DE L'ÚS FETA PER L'INFORMANT. Calma el dolor de les cremades lleus. FORMA FARMACÈUTICA I ÚS. Macerat en oli (ús extern). DESTINACIÓ. Medicina humana.

Tranquil·litzant

FONT 3933. DESCRIPCIÓ DE L'ÚS FETA PER L'INFORMANT. Per a calmar els nervis. FORMA FARMACÈUTICA I ÚS. Sense forma farmacèutica (ús directe) (ús extern). MODE D'UTILITZACIÓ/POSOLOGIA. Es posa en saquets a sota el coixí. DESTINACIÓ. Medicina humana.

**Summitat florífera**

Antisèptic extern

FONT 3952. DESCRIPCIÓ DE L'ÚS FETA PER L'INFORMANT. La infusió de lavanda es fa servir per a rentar llagues i ferides infectades. FORMA FARMACÈUTICA I ÚS. Infusió (ús extern). DESTINACIÓ. Medicina humana.

Per a les picades

FONT 3952. FORMA FARMACÈUTICA I ÚS. Macerat en oli (ús extern). DESTINACIÓ. Medicina humana.

Sedant

FONTS 2226, 3952. DESCRIPCIÓ DE L'ÚS FETA PER L'INFORMANT. Només en olorar-la indueix el son i et tranquil·litza (2226). Només el fet d'aspirar l'aroma de la lavanda ja fa una acció sedant. És molt recomanable per a nens hiperactius o que dormen malament. Es posen unes gotes d'essència de lavanda en el coixí o un mocador al costat (3952). FORMA FARMACÈUTICA I ÚS. Aerosol (ús intern). DESTINACIÓ. Medicina humana.

**USOS ALIMENTARIS**

**Tija amb fulles/branques**

No consta el tipus d'ingestió - No consta el mode de preparació

FONT 3952. DESCRIPCIÓ DE L'ÚS FETA PER L'INFORMANT. Se'n posava un manat a les gàbies d'ocells quan mudaven la ploma. DESTINACIÓ. Alimentació animal.

**ALTRES USOS**

**Flor**

Ambientador

FONT 2232. DESCRIPCIÓ DE L'ÚS FETA PER L'INFORMANT. Les flors s'utilitzaven per a perfumar la roba i els armaris

Repel·lent

FONT 2232. DESCRIPCIÓ DE L'ÚS FETA PER L'INFORMANT. Les flors s'utilitzaven per a evitar que les arnes entressin als armaris i fessin malbé la roba.

Creences i pràctiques magicoreligioses

FONT 2232. DESCRIPCIÓ DE L'ÚS FETA PER L'INFORMANT. Cremant flors d'espígol s'aconsegueix purificar l'ambient.

**Part aèria florida**

Ambientador

FONT 2209, 2225, 2226. DESCRIPCIÓ DE L'ÚS FETA PER L'INFORMANT. Per a fer perfums (2209). Ornamental. Per a fer olor a la llar (2225). Se n’obté perfum (2226).

Elaboració de rams

FONT 2187. DESCRIPCIÓ DE L'ÚS FETA PER L'INFORMANT. Per a decoració. En el mes de les cireres, en venien al mercat en manats petits.

**BARREGES AMB AQUEST TÀXON (vegeu catàleg de barreges)**

**USOS MEDICINALS**

**Tija amb fulles/branques**

FONT 2204. Esperit de vi amb espígol i romaní.

FONT 2209, 2211. Esperit de vi d'herbes.

***Lavandula stoechas***L. (labiades)
BCN 127879

**NOMS POPULARS**

Cap d'ase (2162, 2190, 3952, 2229)

**USOS MEDICINALS**

**Summitat florífera**

Orexigen

FONT 3952. FORMA FARMACÈUTICA I ÚS. Desconegut per l'informant / No consta. DESTINACIÓ. Medicina humana.

Tranquil·litzant

FONT 3952. DESCRIPCIÓ DE L'ÚS FETA PER L'INFORMANT. En infusió, equilibra el sistema nerviós central. Va bé per al mareig, desmais o palpitacions. FORMA FARMACÈUTICA I ÚS. Infusió (ús intern). DESTINACIÓ. Medicina humana.

**ALTRES USOS**

**Part aèria florida**

Literatura oral popular: llegendes, gloses, contes, dites, refranys, poemes, cançons

FONT 2190. DESCRIPCIÓ DE L'ÚS FETA PER L'INFORMANT. El cap d’ase porta la sort a casa.

**Summitat florífera**

Cosmètic

FONT 2229. DESCRIPCIÓ DE L'ÚS FETA PER L'INFORMANT. Bona per al cabell. Banys amb la infusió.

**BARREGES AMB AQUEST TÀXON (vegeu catàleg de barreges)**

**Part aèria florida**

FONT 2162. Per al cos.

***Lepidium draba***L. (crucíferes)
BCN 150382

**NOMS POPULARS**

Capellans (2160, 2178)

**USOS ALIMENTARIS**

**Llavor**

Condiment

FONT 2214. DESCRIPCIÓ DE L'ÚS FETA PER L'INFORMANT. Les llavors s’utilitzen com la mostassa, per a aromatitzar menjars. En cru. DESTINACIÓ. Alimentació humana.

**ALTRES OBSERVACIONS**

FONT 2160. DESCRIPCIÓ FETA PER L'INFORMANT. Es diuen així perquè són difícils d’exterminar, com els capellans.

***Lilium candidum***L. (liliàcies)
BCN 46841

**NOMS POPULARS**

Lliri de Sant Antoni (2204)

**USOS MEDICINALS**

**Flor**

Cicatritzant

FONT 2204. FORMA FARMACÈUTICA I ÚS. Sense forma farmacèutica (ús directe) (ús extern). MODE D'UTILITZACIÓ/POSOLOGIA. S’aplicava un tall de la flor d’un lliri de damunt de la ferida. Calia anar canviant la flor cada dia. D’aquesta manera la ferida cicatritzava més ràpid. DESTINACIÓ. Medicina humana.

***Linum usitatissimum***L. (linàcies)
BCN 47281

**NOMS POPULARS**

Lli (2224, 2225)

Llinosa (2204)

**USOS MEDICINALS**

**Trituració del gra**

Estomacal

FONT 2204. FORMA FARMACÈUTICA I ÚS. Cataplasma (ús extern). PREPARACIÓ. S’havia de ficar uns grapadets de farina de llinosa dins d’un pot amb aigua, i posar-lo al foc. Quan hi havia bullit uns minuts, es posava la pasta de la farina en un drap. Aleshores, es col·locava damunt la panxa del malalt fins que el drap es refredava. MODE D'UTILITZACIÓ/POSOLOGIA. Es feia un cataplasma de farina de llinosa per a curar el mal de panxa. DESTINACIÓ. Medicina humana.

Laxant

FONT 2204. DESCRIPCIÓ DE L'ÚS FETA PER L'INFORMANT. Es ficava aigua i gra de llinosa a bullir en una olla i un raig d’oli. L’olla tenia una mànega de goma adossada a sota, d’un metre de llargada, amb una cànula amb aixeta. El pacient havia de col·locar-se de cul enlaire mentre una segona persona sostenia l’olla ben alta, perquè la força de la gravetat hi actués i una tercera persona introduïa la cànula per l’anus, per on rajava el líquid calent. FORMA FARMACÈUTICA I ÚS. Ènema (ús intern). DESTINACIÓ. Medicina humana.

**ALTRES USOS**

**Llavor**

Elaboració d'obres artístiques

FONTS 2224, 2225. DESCRIPCIÓ DE L'ÚS FETA PELS INFORMANTS. Per a fer paneres artístiques (2224, 2225).

***Lippia triphylla***(L'Hér.) O.Kuntze (verbenàcies)
BCN 126561

**NOMS POPULARS**

Marialluïsa (2167, 2191, 2204, 2210, 2211, 2219, 2217, 3935, 2229, 2218, 2228)

**USOS MEDICINALS**

**Fulla**

Analgèsic

FONT 3935. DESCRIPCIÓ DE L'ÚS FETA PER L'INFORMANT. És antineuràlgica. FORMA FARMACÈUTICA I ÚS. Infusió (ús intern). DESTINACIÓ. Medicina humana.

Carminatiu

FONT 2217. FORMA FARMACÈUTICA I ÚS. Infusió (ús intern). DESTINACIÓ. Medicina humana.

Digestiu

FONTS 2210, 2211, 2217. FORMA FARMACÈUTICA I ÚS. Infusió (ús intern). DESTINACIÓ. Medicina humana.

Estomacal

FONTS 2204, 3935. FORMA FARMACÈUTICA I ÚS. Infusió (ús intern). DESTINACIÓ. Medicina humana.

Per a trastorns del sistema digestiu

FONT 2217. FORMA FARMACÈUTICA I ÚS. Infusió (ús intern). DESTINACIÓ. Medicina humana.

Sedant

FONT 3935. FORMA FARMACÈUTICA I ÚS. Infusió (ús intern). DESTINACIÓ. Medicina humana.

**Tija amb fulles/branques**

Antisèptic extern

FONT 2229. DESCRIPCIÓ DE L'ÚS FETA PER L'INFORMANT. Desinfectant. FORMA FARMACÈUTICA I ÚS. Desconegut per l'informant / No consta. DESTINACIÓ. Medicina humana.

Per a trastorns del sistema digestiu

FONT 2229. DESCRIPCIÓ DE L'ÚS FETA PER L'INFORMANT. Per a l’estómac, en infusió. FORMA FARMACÈUTICA I ÚS. Infusió (ús intern). DESTINACIÓ. Medicina humana.

**USOS ALIMENTARIS**

**Fulla**

Preparació de begudes - Beguda preparada amb aigua

FONT 3935. DESCRIPCIÓ DE L'ÚS FETA PER L'INFORMANT. El preparat a base de les seves fulles és una beguda refrescant. DESTINACIÓ. Alimentació humana.

**ALTRES USOS**

**Fulla**

Ambientador

FONT 3935. DESCRIPCIÓ DE L'ÚS FETA PER L'INFORMANT. La utilització de les fulles seques en saquets, dins de l'armari, provoca que la roba faci bona olor.

***Litchi sinensis***Sonner (sapindàcies)
BCN 118017

**NOMS POPULARS**

Litxi (fruit) (2200)

**USOS ALIMENTARIS**

**Fruit**

Ingestió de la part de la planta crua - Fresca (sense preparació)

FONT 2200. DESTINACIÓ. Alimentació humana.

***Lolium multiflorum***Lam. (gramínies)
BCN 130941

**NOMS POPULARS**

Raigràs (2216)

***Lolium perenne***L. (gramínies)
BCN 58204

**NOMS POPULARS**

Margall (2184)

***Malva sylvestris***L. (malvàcies)
BCN 127888

**NOMS POPULARS**

Malva (2160, 2162, 2170, 2171, 2174, 2178, 2179, 2182, 2192, 2204, 2209, 2210, 2214, 2216, 2219, 2217, 2232, 3937, 3939, 2229, 2226, 2228)

Panellet (fruit tendre) (2214)

**USOS MEDICINALS**

**Flor**

Per a trastorns del sistema digestiu

FONT 2217. DESCRIPCIÓ DE L'ÚS FETA PER L'INFORMANT. Per a desinflamar la panxa i l’aparell digestiu. FORMA FARMACÈUTICA I ÚS. Infusió (ús intern). DESTINACIÓ. Medicina humana.

**Fulla**

Antidiarreic

FONT 2182. FORMA FARMACÈUTICA I ÚS. Infusió (ús intern). MODE D'UTILITZACIÓ/POSOLOGIA. L'aigua de malves es feia bullint fulles de malva en aigua. DESTINACIÓ. Medicina humana.

Antihemorroidal

FONT 3937. DESCRIPCIÓ DE L'ÚS FETA PER L'INFORMANT. Per a les morenes. Les molèsties s’alleugen des del primer bany. FORMA FARMACÈUTICA I ÚS. Desconegut per l'informant / No consta. MODE D'UTILITZACIÓ/POSOLOGIA. Es va mullant l’anus amb l’aigua del bull freda, sense tocar ni vagina ni penis. DESTINACIÓ. Medicina humana.

Antiinflamatori

FONT 2229. DESCRIPCIÓ DE L'ÚS FETA PER L'INFORMANT. Per a treure inflamacions (aigua de malves). FORMA FARMACÈUTICA I ÚS. Infusió (ús extern).

Antipneumònic

FONTS 2209, 2210. DESCRIPCIÓ DE L'ÚS FETA PELS INFORMANTS. Es feia un cataplasma per a curar la pulmonia. Es posava una nit a sobre del pit (2209). FORMA FARMACÈUTICA I ÚS. Cataplasma (ús extern). MODE D'UTILITZACIÓ/POSOLOGIA. En cataplasma, sobre el pit. Per a baixar la inflamació dels pulmons (2210). DESTINACIÓ. Medicina humana.

Digestiu

FONT 2182. FORMA FARMACÈUTICA I ÚS. Infusió (ús intern). MODE D'UTILITZACIÓ/POSOLOGIA. L'aigua de malves es feia bullint fulles de malva en aigua. DESTINACIÓ. Medicina humana.

Diürètic

FONT 2229. DESCRIPCIÓ DE L'ÚS FETA PER L'INFORMANT. Per a fer pipí. FORMA FARMACÈUTICA I ÚS. Infusió (ús intern). DESTINACIÓ. Medicina humana.

Laxant

FONT 2216. DESCRIPCIÓ DE L'ÚS FETA PER L'INFORMANT. Bullint les malves es feia aigua de malves, que donàvem als porcs per a anar de cos (per a afavorir l’evacuació). FORMA FARMACÈUTICA I ÚS. Decocció (ús intern). DESTINACIÓ. Medicina veterinària.

Per a trastorns del sistema digestiu

FONT 2162. DESCRIPCIÓ DE L'ÚS FETA PER L'INFORMANT. Bon remei per beure, contra el mal de panxa. FORMA FARMACÈUTICA I ÚS. Infusió (ús intern). DESTINACIÓ. Medicina humana.

Per a trastorns del sistema respiratori

FONTS 2226, 2232. DESCRIPCIÓ DE L'ÚS FETA PELS INFORMANTS. Els bafs [dit "bafos"] de bullir les seves fulles van bé per a la respiració (2226). FORMA FARMACÈUTICA I ÚS. Aerosol (ús intern) (2226). DESCRIPCIÓ DE L'ÚS FETA PELS INFORMANTS. Per als pulmons (2232). FORMA FARMACÈUTICA I ÚS. Infusió (ús intern) (2232). DESTINACIÓ. Medicina humana (2226, 2232).

Per al malestar

FONT 2182. DESCRIPCIÓ DE L'ÚS FETA PER L'INFORMANT. Per al mareig. FORMA FARMACÈUTICA I ÚS. Infusió (ús intern). DESTINACIÓ. Medicina humana.

**Part aèria**

Analgèsic

FONT 2228. DESCRIPCIÓ DE L'ÚS FETA PER L'INFORMANT. Calma el dolor. FORMA FARMACÈUTICA I ÚS. Desconegut per l'informant / No consta. DESTINACIÓ. Medicina humana.

Estomacal

FONT 2204. DESCRIPCIÓ DE L'ÚS FETA PER L'INFORMANT. Per a guarir el mal de ventre. S’havia de fer bullir les flors, les fulles o les llavors de les malves i, després d’haver colat el beuratge, beure’ns-el. FORMA FARMACÈUTICA I ÚS. Infusió (ús intern). DESTINACIÓ. Medicina humana.

No consta

FONT 3939. DESCRIPCIÓ DE L'ÚS FETA PER L'INFORMANT. Se’n fa aigua de malva, que és bona com a medicament (desconeix per a què). FORMA FARMACÈUTICA I ÚS. Infusió (ús intern). DESTINACIÓ. Medicina humana.

**Pecíol**

Purgant

FONT 2174. DESCRIPCIÓ DE L'ÚS FETA PER L'INFORMANT. Útil en bebès. S'agafa la cua [dit "el *rabo*"] de la fulla, s'unta amb oli d'oliva temperat i es fica en l'anus. FORMA FARMACÈUTICA I ÚS. Macerat en oli (ús extern). DESTINACIÓ. Medicina humana.

**USOS ALIMENTARIS**

**Flor**

Ingestió de la part de la planta crua - Fresca (sense preparació)

FONT 2228. DESCRIPCIÓ DE L'ÚS FETA PER L'INFORMANT. Les flors en amanida. CONSUMICIÓ. Amanida.

**Fruit**

Ingestió de la part de la planta crua - Fresca (sense preparació)

FONT 2160. DESCRIPCIÓ DE L'ÚS FETA PER L'INFORMANT. El fruit [dit "la llavor"] sembla un pa de Viena i se’l menjaven de petits. CONSUMICIÓ. Llaminadura. DESTINACIÓ. Alimentació humana.

**Fruit immadur sencer**

Ingestió de la part de la planta crua - Fresca (sense preparació)

FONTS 2214, 2216. DESCRIPCIÓ DE L'ÚS FETA PELS INFORMANTS. De petits, se’n menjaven els fruits tendres [dit "panellets"] (2214). El fruit, encara immadur, era menjat pels infants en cru, com a llepolia [dit "*golosina*"] (2216). CONSUMICIÓ. Llaminadura (2214). DESTINACIÓ. Alimentació humana (2214, 2216).

**Fulla**

Ingestió de la part de la planta cuita - Cuita en aigua

FONT 2232. DESCRIPCIÓ DE L'ÚS FETA PER L'INFORMANT. Se’n mengen les fulles com si fos una verdura. DESTINACIÓ. Alimentació humana.

**Part aèria**

No consta el tipus d'ingestió - No consta el mode de preparació

FONTS 2214, 2226. DESCRIPCIÓ DE L'ÚS FETA PER L'INFORMANT. Per als animals (2226). DESTINACIÓ. Alimentació animal (2226). Alimentació humana (2214).

**ALTRES USOS**

**Planta sencera**

Literatura oral popular: llegendes, gloses, contes, dites, refranys, poemes, cançons

FONT 2192. DESCRIPCIÓ DE L'ÚS FETA PER L'INFORMANT. “Està al país de les malves”, frase popular que fa referència al fet que algú està mort.

**Planta viva *in situ***

Agrosilvopastoral

FONT 2178. DESCRIPCIÓ DE L'ÚS FETA PER L'INFORMANT. És font de fauna auxiliar, tot i que dins del camp fa molta nosa perquè creix massa. OBSERVACIONS. Associació de cultius i equilibri sistèmic.

**BARREGES AMB AQUEST TÀXON (vegeu catàleg de barreges)**

**Part aèria florida**

FONT 2162. Per al cos.

***Mangifera indica***L. (anacardiàcies)
BCN-E-281

**NOMS POPULARS**

Mango (fruit) (2200, 2201)

**USOS ALIMENTARIS**

**Fruit**

Ingestió de la part de la planta crua - Fresca (sense preparació)

FONTS 2200, 2201. DESTINACIÓ. Alimentació humana.

***Manihot esculenta***Krantz (euforbiàcies)
BCN-E-219

**NOMS POPULARS**

Iuca (2200)

**USOS ALIMENTARIS**

**Arrel**

No consta el tipus d'ingestió - No consta el mode de preparació

FONT 2200. DESTINACIÓ. Alimentació humana.

***Matricaria recutita***L. (compostes)
BCN 130942

**NOMS POPULARS**

*Manzanilla* (castellà) (2182)

Bolitx (2184)

Camamilla (2183, 2184, 2204, 2209, 2214, 2216, 2219, 2217, 2231, 2229, 2218, 2226)

Mançanilla (2217)

**USOS MEDICINALS**

**Inflorescència**

Antisèptic ocular

FONTS 2183, 2226, 2231. DESCRIPCIÓ DE L'ÚS FETA PELS INFORMANTS. Per a rentar-se els ulls (2183). Serveix per a fer banys oculars (2226). FORMA FARMACÈUTICA I ÚS. Bany (ús extern) (2183, 2226, 2231). DESTINACIÓ. Medicina humana (2183, 2226, 2231).

Digestiu

FONTS 2182, 2216, 2217. DESCRIPCIÓ DE L'ÚS FETA PER L'INFORMANT. Se’n fan infusions per a afavorir la digestió. Hi havia unes dones de Barcelona que en feien manats per a emportar-se a vendre (2216). FORMA FARMACÈUTICA I ÚS. Infusió (ús intern) (2182, 2216, 2217). DESTINACIÓ. Medicina humana (2182, 2216, 2217).

Estomacal

FONTS 2204, 2229. DESCRIPCIÓ DE L'ÚS FETA PER L'INFORMANT. Per a guarir el mal de ventre (2204). En infusió, per al mal d’estómac (2229). FORMA FARMACÈUTICA I ÚS. Infusió (ús intern). DESTINACIÓ. Medicina humana.

Per a trastorns de la pell o del teixit subcutani

FONT 2229. DESCRIPCIÓ DE L'ÚS FETA PER L'INFORMANT. Per al cutis. Es feia un cataplasma: es bullia, es mullava un drap i es posava allà on feia mal. FORMA FARMACÈUTICA I ÚS. Cataplasma (ús extern). DESTINACIÓ. Medicina humana.

Per a trastorns del sistema digestiu

FONT 2219. DESCRIPCIÓ DE L'ÚS FETA PELS INFORMANTS. En infusió és bona per als problemes digestius. FORMA FARMACÈUTICA I ÚS. Infusió (ús intern). DESTINACIÓ. Medicina humana.

Per al refredat

FONT 2184. FORMA FARMACÈUTICA I ÚS. Infusió (ús intern). DESTINACIÓ. Medicina humana.

Per als ulls

FONTS 2204, 2209, 2214. DESCRIPCIÓ DE L'ÚS FETA PELS INFORMANTS. La infusió de camamilla es ficava dins d’unes copes. Aleshores, amb el cap cot, s’havia de col·locar la copa i empènyer-la tot al voltant dels ulls, s’aixecava el cap i movent els ulls rebien un bany, i així s’havia d’aguantar una bona estona, amb la infusió tèbia (2204). FORMA FARMACÈUTICA I ÚS. Bany (ús extern) (2204, 2209, 2214). MODE D'UTILITZACIÓ/POSOLOGIA. Es fa servir l’aigua de la infusió un cop tèbia (2214). DESTINACIÓ. Medicina humana (2204, 2209, 2214).

Sedant

FONT 2226. DESCRIPCIÓ DE L'ÚS FETA PER L'INFORMANT. En infusió és sedant. FORMA FARMACÈUTICA I ÚS. Infusió (ús intern). DESTINACIÓ. Medicina humana.

**ALTRES OBSERVACIONS**

FONT 2219. DESCRIPCIÓ FETA PER L'INFORMANT. De vegades havien vingut a buscar-ne al camp per a vendre.

***Medicago sativa***L. (papilionàcies)
BCN 126568

**NOMS POPULARS**

Alfals (2164, 2204, 2212)

Ufals (2166, 2181, 2182, 2184, 2186, 2209, 2210, 2217, 2218, 2229, 3700, 3934)

**USOS MEDICINALS**

**Part aèria**

Per a trastorns de la pell o del teixit subcutani

FONT 2181. DESCRIPCIÓ DE L'ÚS FETA PER L'INFORMANT. Quan tenies la carn esqueixada, t'embenaven ufals picat a sobre del mal. FORMA

Per als traumatismes

FONT 2204. DESCRIPCIÓ DE L'ÚS FETA PER L'INFORMANT. A vegades, a causa dels cops que els pagesos ens donàvem accidentalment als ossos mentre treballàvem al camp, per tal d’alleugerir-nos el dolor i com a cura d’emergència, havíem d’agafar grapats d’alfals tendre i el picàvem durant una estoneta. Ràpidament, ens l’aplicàvem damunt del cop amb un mocador lligat amb força. Això alleugeria força. FORMA FARMACÈUTICA I ÚS. Cataplasma (ús extern). DESTINACIÓ. Medicina humana.

**USOS ALIMENTARIS**

**Part aèria**

Ingestió de la part de la planta crua - Conservada dessecada a l'aire

FONTS 2184, 2186. DESCRIPCIÓ DE L'ÚS FETA PELS INFORMANTS. Venien ufals per al bestiar, sobretot per al del Parc de la Ciutadella (2186). DESTINACIÓ. Alimentació animal (2184, 2186).

Ingestió de la part de la planta crua - Fresca (sense preparació)

FONTS 2164, 2182, 2184, 3946. DESCRIPCIÓ DE L'ÚS FETA PELS INFORMANTS. Conills i cavalls (2182). Per als animals (3946). DESTINACIÓ. Alimentació animal (2164, 2182, 2184, 3946).

No consta el tipus d'ingestió - No consta el mode de preparació

FONTS 2209, 2210, 2212, 2217, 2218, 2229, 3700, 3934. DESCRIPCIÓ DE L'ÚS FETA PELS INFORMANTS. Cavalls, conills (2210). Per als animals de tir (2212). Per als animals (2217). Se'n feien manats i es venien per a donar de menjar als conills (2218). Menjar per a cavalls i conills (2229). Per al bestiar. A les vaques els espessa la llet (3934). DESTINACIÓ. Alimentació animal (2209, 2210, 2212, 2217, 2218, 2229, 3700, 3934).

**ALTRES USOS**

**Part aèria**

Agrosilvopastoral

FONT 2164, 2182. DESCRIPCIÓ DE L'ÚS FETA PER L'INFORMANT. Agafaven un pilot d'ufals tendre i fregaven la fulla de les dalles per a netejar-les (2182). OBSERVACIONS. Encoixinament. Adobs i fertilitzants naturals (2164).

**BARREGES AMB AQUEST TÀXON (vegeu catàleg de barreges)**

**USOS MEDICINALS**

**Part aèria**

FONT 2217. Per a treure els blaus.

***Melissa officinalis***L. subsp. ***officinalis***(labiades)
BCN 156594

**NOMS POPULARS**

Melissa (2228)

Tarongina (2227)

***Mentha ×gentilis***L. (labiades)
BCN 126534

**NOMS POPULARS**

Menta (2173, 2176, 2177, 2178, 2182, 2188, 2191, 2196, 2197, 2198, 2204, 2216, 2217, 2224, 2229, 2218, 2227, 2228)

**USOS MEDICINALS**

**Fulla**

Digestiu

FONTS 2173, 2217. FORMA FARMACÈUTICA I ÚS. Infusió (ús intern). DESTINACIÓ. Medicina humana.

Estomacal

FONTS 2182, 2204. DESCRIPCIÓ DE L'ÚS FETA PELS INFORMANTS. Per a guarir el mal de ventre (2204). FORMA FARMACÈUTICA I ÚS. Infusió (ús intern). DESTINACIÓ. Medicina humana.

Hepatoprotector

FONT 2228. FORMA FARMACÈUTICA I ÚS. Infusió (ús intern). DESTINACIÓ. Medicina humana.

**USOS ALIMENTARIS**

**Fulla**

Condiment

FONTS 2216, 2217, 2218, 2224, 2229. DESCRIPCIÓ DE L'ÚS FETA PELS INFORMANTS. Per a cuinar. En tens en un raconet i et dura sempre (2216). Cuinada amb les faves, els dona bon gust (2217). Per a les faves. Els dona bon sabor (2224). Per a cuinar (2229). Per a les faves (2218). DESTINACIÓ. Alimentació humana (2216, 2217, 2218, 2224, 2229).

No consta el tipus d'ingestió - No consta el mode de preparació

FONTS 2178, 2196, 2197, 2198. DESCRIPCIÓ DE L'ÚS FETA PELS INFORMANTS. Per a preparar alguns plats (2178). DESTINACIÓ. Alimentació humana (2178, 2196, 2197, 2198).

Preparació de begudes - Beguda preparada amb aigua

FONTS 2176, 2177, 2178. DESCRIPCIÓ DE L'ÚS FETA PELS INFORMANTS. Per a donar sabor a l’aigua (2178). DESTINACIÓ. Alimentació humana (2176, 2177, 2178).

Preparació de begudes - Beguda preparada amb licor

FONTS 2176, 2177. DESCRIPCIÓ DE L'ÚS FETA PELS INFORMANTS. Per a fer *mojitos*. DESTINACIÓ. Alimentació humana.

**BARREGES AMB AQUEST TÀXON (vegeu catàleg de barreges)**

**Fulla**

FONT 2190. Per al refredat.

***Mentha pulegium***L. (labiades)
BCN 127884

**NOMS POPULARS**

Menta (2224, 2226)

Menta poliol (2222)

Poliol (2210, 2226)

Poniol (2191, 2204)

**USOS MEDICINALS**

**Fulla**

Digestiu

FONT 2226. DESCRIPCIÓ DE L'ÚS FETA PER L'INFORMANT. En infusions, elimina les digestions pesades. FORMA FARMACÈUTICA I ÚS. Infusió (ús intern). DESTINACIÓ. Medicina humana.

Diürètic

FONT 2224. FORMA FARMACÈUTICA I ÚS. Infusió (ús intern). DESTINACIÓ. Medicina humana.

Hepatoprotector

FONT 2226. DESCRIPCIÓ DE L'ÚS FETA PER L'INFORMANT. En infusió és bona per als problemes de fetge. FORMA FARMACÈUTICA I ÚS. Infusió (ús intern). DESTINACIÓ. Medicina humana.

**Part aèria florida**

Digestiu

FONTS 2204, 2210, 2222. DESCRIPCIÓ DE L'ÚS FETA PELS INFORMANTS. Per a facilitar les digestions (2204). FORMA FARMACÈUTICA I ÚS. Infusió (ús intern). DESTINACIÓ. Medicina humana.

Estomacal

FONT 2191. FORMA FARMACÈUTICA I ÚS. Desconegut per l'informant / No consta. DESTINACIÓ. Medicina humana.

***Mercurialis annua***L. subsp. ***annua***(euforbiàcies)
BCN 129700

**NOMS POPULARS**

Mercurial (2214)

***Mesembryanthemum crystallinum***L. (aizoàcies)
BCN 130932

**NOMS POPULARS**

Ficoide (2165)

Ficoide glacial (2163)

**USOS ALIMENTARIS**

**Part aèria**

Condiment

FONT 2165. DESCRIPCIÓ DE L'ÚS FETA PER L'INFORMANT. Quan la menges, el seu gust recorda el de les escopinyes [dit "els *berberechos*"] al natural. Es fan sopes i, per sobre, hi tiren trossets de ficoide. DESTINACIÓ. Alimentació humana.

Ingestió de la part de la planta crua - Fresca (sense preparació)

FONT 2163. DESCRIPCIÓ DE L'ÚS FETA PER L'INFORMANT. En amanides i decoració de plats. CONSUMICIÓ. Amanida. DESTINACIÓ. Alimentació humana.

No consta el tipus d'ingestió - No consta el mode de preparació

FONT 2163. DESCRIPCIÓ DE L'ÚS FETA PER L'INFORMANT. A Tailàndia la fan servir en rebosteria. CONSUMICIÓ. Postres. DESTINACIÓ. Alimentació humana.

***Mespilus germanica***L. (rosàcies)
BCN 50768

**NOMS POPULARS**

Nespra (fruit) (2192)

**USOS ALIMENTARIS**

**Fruit**

Ingestió de la part de la planta crua - Fresca (sense preparació)

FONT 2192. DESTINACIÓ. Alimentació humana.

***Montia fontana***L. (portulacàcies)
BCN 126578

**NOMS POPULARS**

*Boruja* (castellà) (2163)

*Corruja* (castellà) (2163)

Herba de la font (2163)

Pamplina d'aigua (2163, 2165)

**USOS ALIMENTARIS**

**Part aèria**

Ingestió de la part de la planta crua - Fresca (sense preparació)

FONTS 2163, 2165. CONSUMICIÓ. Amanida. DESTINACIÓ. Alimentació humana.

***Musa ×paradisiaca***L. (musàcies)
BCN-E-207

**NOMS POPULARS**

*Plátano* (castellà) (2208)

Plàtan (2204)

Plàtan (fruit) (2199, 2200)

**USOS MEDICINALS**

**Fruit**

Antidiarreic

FONT 2208. FORMA FARMACÈUTICA I ÚS. Sense forma farmacèutica (ús directe) (ús intern). PREPARACIÓ. Per a aturar la diarrea. Ben triturat. DESTINACIÓ. Medicina humana.

**USOS ALIMENTARIS**

**Fruit**

Ingestió de la part de la planta crua - Fresca (sense preparació)

FONTS 2199, 2200. DESTINACIÓ. Alimentació humana.

***Musa acuminata***Colla (musàcies)
BCN-E-253

**NOMS POPULARS**

Banana (2201)

Plàtan mascle (fruit) (2200)

**USOS ALIMENTARIS**

**Fruit**

Ingestió de la part de la planta crua - Fresca (sense preparació)

FONT 2201. DESTINACIÓ. Alimentació humana.

No consta el tipus d'ingestió - No consta el mode de preparació

FONT 2200. DESTINACIÓ. Alimentació humana.

***Muscari neglectum***Guss. ex Ten. (asparagàcies)
BCN 129694

**NOMS POPULARS**

All de bruixa (2214)

Calabruixa (2226)

**USOS ALIMENTARIS**

**Flor**

Ingestió de la part de la planta crua - Fresca (sense preparació)

FONT 2226. DESCRIPCIÓ DE L'ÚS FETA PER L'INFORMANT. Alguna vegada se n’aprofitava alguna flor per a menjar. DESTINACIÓ. Alimentació humana.

***Narcissus tazetta***L. subsp. ***tazetta***(amaril·lidàcies)
BCN 129016

**NOMS POPULARS**

Jonquillo (2188, 2189, 2192)

**ALTRES USOS**

**Part aèria florida**

Elaboració de rams

FONT 2189. 2192. DESCRIPCIÓ DE L'ÚS FETA PER L'INFORMANT. Es collien per a vendre en manats (2189). Ornamental. En collien per a vendre (2192).

**ALTRES OBSERVACIONS**

FONTS 2189, 2192. DESCRIPCIÓ FETA PELS INFORMANTS. Diuen que en l’enterrament de Macià, al 1933, es van vendre molts jonquillos de Sant Climent (2189). Esmenta que les úniques flors que va tenir el president Macià en el seu funeral van ser jonquillos de Sant Climent (2192).

***Nerium oleander***L. (apocinàcies)
BCN 156616

**NOMS POPULARS**

Baladre (2191)

**USOS MEDICINALS**

**Flor i fulla**

Antialopècic

FONT 2191. FORMA FARMACÈUTICA I ÚS. Infusió (ús extern). DESTINACIÓ. Medicina humana.

***Ocimum basilicum***L. (labiades)
BCN 126577

**NOMS POPULARS**

*Albahaca* (castellà) (2165)

Alfàbrega (2215, 2217, 2218, 2227, 2226, 2228)

Alfàbrega *mozzarella* (raça) (2165)

Alfàbrega genovesa (raça) (2165)

**USOS MEDICINALS**

**Fulla**

Digestiu

FONT 2217. FORMA FARMACÈUTICA I ÚS. Sense forma farmacèutica (ús directe) (ús intern). DESTINACIÓ. Medicina humana.

**USOS ALIMENTARIS**

**Flor i fulla**

Ingestió de la part de la planta crua - Fresca (sense preparació)

FONT 2165. DESCRIPCIÓ DE L'ÚS FETA PER L'INFORMANT. Alguns clients li demanen només les flors. Les fan servir en cru per decorar amanides. La més típica pel cultiu és l'alfàbrega genovesa [raça], però s'espiga molt ràpid i té problemes per a ser cultivada. L'alfàbrega *mozzarella* [raça] té una fulla més gran i aguanta més el fred i la humitat. CONSUMICIÓ. Amanida. DESTINACIÓ. Alimentació humana.

**Fulla**

Condiment

FONT 2217. DESCRIPCIÓ DE L'ÚS FETA PER L'INFORMANT. Per a cuinar va molt bé. Picada per sobre el peix li va molt bé. DESTINACIÓ. Alimentació humana.

Ingestió de la part de la planta cuita - Cuita en oli

FONT 2217. DESCRIPCIÓ DE L'ÚS FETA PER L'INFORMANT. Es pot usar per a fer *pesto*. DESTINACIÓ. Alimentació humana.

No consta el tipus d'ingestió - No consta el mode de preparació

FONTS 2215, 2218, 2226, 2227, 2228. DESCRIPCIÓ DE L'ÚS FETA PELS INFORMANTS. Comestible (2218, 2226, 2227). Per a fer salsa *pesto* (2228). DESTINACIÓ. Alimentació humana (2215, 2218, 2226, 2227, 2228).

***Olea europaea***L. subsp. ***europaea*** var. ***europaea***(oleàcies)
BCN 129008

**NOMS POPULARS**

Oli d'oliva (producte elaborat) (2182, 2189, 2192, 2204, 2221, 2213, 2222, 2224, 2232, 2228)

Oliva (fruit) (2187, 2189, 2208)

Olivera (2167, 2181, 2184, 2189, 2190, 2191, 2192, 2212, 2229, 2226)

**USOS MEDICINALS**

**Fulla**

Regulador de la tensió arterial

FONT 2191. FORMA FARMACÈUTICA I ÚS. Infusió (ús intern). DESTINACIÓ. Medicina humana.

Antiamigdalític

FONT 2181. FORMA FARMACÈUTICA I ÚS. Loció (ús extern). MODE D'UTILITZACIÓ/POSOLOGIA. El seu oli servia per a curar les angines [dit “trencar les angines”]. Es posava oli al canell i es feien fregues estrenyent fortament el dit polze sobre l’avantbraç. DESTINACIÓ. Medicina humana.

Per a les picades d'abelles

FONT 2204. DESCRIPCIÓ DE L'ÚS FETA PER L'INFORMANT. Per a les picades d'abelles. FORMA FARMACÈUTICA I ÚS. Sense forma farmacèutica (mescla medicamentosa) (ús extern). DESTINACIÓ. Medicina humana.

Per a treure espines

FONT 2182. DESCRIPCIÓ DE L'ÚS FETA PER L'INFORMANT. Les palles es feien servir per a "fer una palla". Quan el bestiar o els mossos es clavaven una punxa, agafaven una palla de blat -del paller-, ho posaven perpendicularment sobre la ferida i hi tiraven oli d'oliva per dintre; s'encenia amb una espelma per la part superior i l'oli calent arribava fins a la zona adolorida, curant-la. FORMA FARMACÈUTICA I ÚS. Sense forma farmacèutica (ús directe) (ús extern). DESTINACIÓ. Medicina humana i veterinària.

**Tija amb fulles/branques**

Vasotònic

FONT 2229. DESCRIPCIÓ DE L'ÚS FETA PER L'INFORMANT. Per a la circulació, en decocció. FORMA FARMACÈUTICA I ÚS. Decocció (ús intern). DESTINACIÓ. Medicina humana.

**USOS ALIMENTARIS**

**Fruit**

Ingestió de la part de la planta crua - Conservada en salmorra

FONTS 2187, 2189, 2208. CONSUMICIÓ. Aperitiu (2187). DESTINACIÓ. Alimentació humana (2187, 2189, 2208).

Ingestió de la part de la planta crua - Fresca (sense preparació)

FONT 2192. DESCRIPCIÓ DE L'ÚS FETA PER L'INFORMANT. Com a menjar per a les cabres. DESTINACIÓ. Alimentació animal.

No consta el tipus d'ingestió - No consta el mode de preparació

FONT 2189. DESCRIPCIÓ DE L'ÚS FETA PER L'INFORMANT. Per a fer oli. DESTINACIÓ. Alimentació humana.

**ALTRES USOS**

**Tija amb fulles/branques**

Creences i pràctiques magicoreligioses

FONT 2189. DESCRIPCIÓ DE L'ÚS FETA PER L'INFORMANT. La setmana d’abans del Diumenge de Rams sortien de Sant Climent camions plens de feixos d'olivera per a vendre a l’engròs i a la menuda. El volien per a anar a beneir.

**Tija defoliada**

Agrosilvopastoral

FONT 2167. DESCRIPCIÓ DE L'ÚS FETA PER L'INFORMANT. Per al pas d'una aixada. OBSERVACIONS. Elaboració d'estris d'ús hortícola/agrícola.

**BARREGES AMB AQUEST TÀXON (vegeu catàleg de barreges)**

**USOS ALIMENTARIS**

**Fruit**

FONT 2190, 2192. Olives arreglades.

FONT 2192. Conserva de tomàquet.

FONT 2192. Platillo de Sant Climent.

**USOS MEDICINALS**

**Suc del fruit**

FONT 2182. Palla.

FONT 2192. Cataplasma per al mal d'esquena.

FONT 2204. Palla.

FONT 2213. Cataplasma per a les hemorroides.

FONT 2228. Oli de boixac i rosa.

***Opuntia maxima***Mill. (cactàcies)
BCN-E-293

**NOMS POPULARS**

Figa de moro (fruit) (2189, 2190, 2211, 2213, 2224, 2218)

Figuera de moro (2210)

**USOS MEDICINALS**

**Cladodi**

Per al refredat

FONT 2190. FORMA FARMACÈUTICA I ÚS. Infusió (ús intern). PREPARACIÓ. El cladodi [dit "la fulla"], bullida com a infusió, és bona per al constipat. DESTINACIÓ. Medicina humana.

**USOS ALIMENTARIS**

**Fruit**

Ingestió de la part de la planta crua - Fresca (sense preparació)

FONTS 2189, 2190, 2211, 2213, 2218, 2224. DESCRIPCIÓ DE L'ÚS FETA PELS INFORMANTS. Els venien per a l’alimentació (2218). DESTINACIÓ. Alimentació humana (2189, 2190, 2211, 2213, 2218, 2224).

**ALTRES USOS**

**Planta viva *in situ***

Agrosilvopastoral

FONTS 2190, 2210. DESCRIPCIÓ DE L'ÚS FETA PELS INFORMANTS. Es planta als marges dels camps per a evitar que la gent hi entri (2210). Per a fer tancaments en els camps i que no hi entri el porc senglar (2190). OBSERVACIONS. Elements paisatgístics (2210).

***Origanum majorana***L. (labiades)
BCN 128107

**NOMS POPULARS**

Marduix (2191, 2226)

**USOS ALIMENTARIS**

**Tija amb fulles/branques**

Condiment

FONTS 2191, 2226. DESCRIPCIÓ DE L'ÚS FETA PELS INFORMANTS. Per a adobar els cargols (2191). Per a aromatitzar estofats (2226). DESTINACIÓ. Alimentació humana (2191, 2226).

**ALTRES USOS**

**Fulla**

Altres informacions

FONT 2226. DESCRIPCIÓ DE L'ÚS FETA PER L'INFORMANT. Les fulles seques serveixen per a les indústries farmacèutica i licorera.

***Origanum vulgare***L. (labiades)
BCN 130951

**NOMS POPULARS**

Orenga (2189, 2190, 2207, 3933, 3952, 2229, 2226, 2228)

**USOS MEDICINALS**

**Part aèria**

Antiinflamatori / Antiàlgic muscular

FONT 3952. DESCRIPCIÓ DE L'ÚS FETA PER L'INFORMANT. Per al torticoli i per al lumbago, aplicat externament tant en cataplasmes com en friccions sobre la pell (amb l'essència). FORMA FARMACÈUTICA I ÚS. Cataplasma (ús extern). DESTINACIÓ. Medicina humana.

Antitussigen

FONT 3952. DESCRIPCIÓ DE L'ÚS FETA PER L'INFORMANT. Té una acció antitussígena. MODE D'UTILITZACIÓ/POSOLOGIA. Se'n fan bafs. FORMA FARMACÈUTICA I ÚS. Aerosol (ús intern). DESTINACIÓ. Medicina humana.

Digestiu

FONT 3952. FORMA FARMACÈUTICA I ÚS. Desconegut per l'informant / No consta. DESTINACIÓ. Medicina humana.

Expectorant

FONT 3952. DESCRIPCIÓ DE L'ÚS FETA PER L'INFORMANT. Té una acció expectorant. MODE D'UTILITZACIÓ/POSOLOGIA. Se'n fan bafs. FORMA FARMACÈUTICA I ÚS. Aerosol (ús intern). DESTINACIÓ. Medicina humana.

Salutífer

FONT 2228. DESCRIPCIÓ DE L'ÚS FETA PER L'INFORMANT. Estimula les defenses. FORMA FARMACÈUTICA I ÚS. Sense forma farmacèutica (ús directe) (ús intern). DESTINACIÓ. Medicina humana.

**Summitat florífera**

Per a trastorns del sistema digestiu

FONTS 2226, 3933. FORMA FARMACÈUTICA I ÚS. Infusió (ús intern). DESTINACIÓ. Medicina humana.

Per a trastorns del sistema respiratori

FONT 2226. FORMA FARMACÈUTICA I ÚS. Infusió (ús intern). DESTINACIÓ. Medicina humana.

**USOS ALIMENTARIS**

**Fulla**

Condiment

FONTS 2190, 2207, 2228, 3952. DESCRIPCIÓ DE L'ÚS FETA PELS INFORMANTS. Per a condimentar la salsa de tomàquet (2207). Com a condiment en amanides i pizzes (3952). Per a la pizza (2228). DESTINACIÓ. Alimentació humana (2190, 2207, 2228, 3952).

**Tija amb fulles/branques**

Condiment

FONTS 2189, 2229. DESCRIPCIÓ DE L'ÚS FETA PELS INFORMANTS. Per a fer sardines en escabetx (2189). Per a cuinar (2229). DESTINACIÓ. Alimentació humana (2189, 2229).

***Oryza sativa***L. (gramínies)
BCN 30000

**NOMS POPULARS**

Arròs (2185, 3700, 2216, 2224, 2225, 2223)

**USOS ALIMENTARIS**

**Llavor**

Ingestió de la part de la planta cuita - Cuita en aigua

FONT 2216. DESTINACIÓ. Alimentació humana.

**ALTRES USOS**

**Llavor**

Elaboració d'obres artístiques

FONTS 2223, 2224, 2225. DESCRIPCIÓ DE L'ÚS FETA PELS INFORMANTS. Per a l’elaboració de les paneres artístiques. Es posa cola blanca per a enganxar plastilina; quan està sec, s’enganxen els grans a sobre de la plastilina amb més cola blanca (2223). Per a fer les paneres artístiques, els grans es poden tenyir amb tint per a la roba. Pintar-los no està ben vist. Per a fer les paneres, els grans, els van col·locant d’un en un, amb cola blanca, ajudant-se d’un escuradents (2224). Per a fer paneres artístiques (2224, 2225).

**Planta viva *in situ***

Agrosilvopastoral

FONT 2185. DESCRIPCIÓ DE L'ÚS FETA PER L'INFORMANT. Fa molts anys se n'havia fet en terrenys salins. La terra estava lliure de sal l'any següent i s'hi podien plantar altres coses. OBSERVACIONS. Associació de cultius i equilibri sistèmic.

**ALTRES OBSERVACIONS**

FONTS 2216, 2224, 3700. DESCRIPCIÓ FETA PELS INFORMANTS. Durant els primers anys del segle XX, l’arròs es va estendre al Prat de Llobregat. Als anys 1920 va ser prohibit per la seva incidència en l’extensió del paludisme. Després de la Guerra Civil, es va conrear en les finques properes a la costa (3700). Abans se’n feia molt en les maresmes, sobretot a cal Lluc i l’Estorac, a la Ricarda i a l’Aviació (2216).

***Oryzopsis miliacea***(L.) Asch. et Graebn. subsp. ***miliacea***(gramínies)
BCN 130940

**NOMS POPULARS**

Margall (2219)

***Oxalis debilis***Humb., Bonpl. et Kunth. (oxalidàcies)
BCN 133002

**NOMS POPULARS**

Trèvol (2219)

***Papaver rhoeas***L. (papaveràcies)
BCN 130964

**NOMS POPULARS**

*Amapola* (castellà) (3932)

Piripipí (2226)

Roella (2226)

Rosella (2210, 3952, 2226)

**USOS MEDICINALS**

**Flor**

Antiodontàlgic

FONT 3952. FORMA FARMACÈUTICA I ÚS. Infusió (ús extern). MODE D'UTILITZACIÓ/POSOLOGIA. Glopejar la infusió dels seus pètals produeix un notable efecte analgèsic, útil per al mal de queixal. DESTINACIÓ. Medicina humana.

**Llavor**

Tranquil·litzant

FONT 3932. DESCRIPCIÓ DE L'ÚS FETA PER L'INFORMANT. Per a l’insomni i el nerviosisme. En cru va bé. FORMA FARMACÈUTICA I ÚS. Sense forma farmacèutica (ús directe) (ús intern). DESTINACIÓ. Medicina humana.

**ALTRES USOS**

**Flor**

Jocs i joguines

FONT 2210. DESCRIPCIÓ DE L'ÚS FETA PER L'INFORMANT. Recorda el joc del “poll, gallina o pollastre”; es diu què sortirà del capoll: si surt blanc, és poll; rosa és gallina i vermell és pollastre.

***Papaver somniferum***L. (papaveràcies)
BCN 24941

**NOMS POPULARS**

Cascall (2231, 3952)

**USOS MEDICINALS**

**No consta**

Sedant

FONT 2231. DESCRIPCIÓ DE L'ÚS FETA PER L'INFORMANT. A cada casa en tenien. FORMA FARMACÈUTICA I ÚS. Desconegut per l'informant / No consta. DESTINACIÓ. Medicina humana.

**ALTRES OBSERVACIONS**

FONT 3952. DESCRIPCIÓ FETA PER L'INFORMANT. Sempre n'hi havia a les cases de pagès.

***Parietaria officinalis***L. subsp. ***judaica***(L.) Béguinot (urticàcies)
BCN 126542

**NOMS POPULARS**

Herba morella roquera (2204)

Herba roquera (2226)

Paretària (2160, 2219)

Parietària (3952)

Trenca-roques (2178)

**USOS MEDICINALS**

**Fulla**

Diürètic

FONT 3952. DESCRIPCIÓ DE L'ÚS FETA PER L'INFORMANT. Es pot prendre en infusió -40 a 60 grams de planta fresca per litre d'aigua-, de la qual es prenen quatre o cinc tasses diàries- o en suc fresc, prenent-ne mig got tres cops al dia.

FORMA FARMACÈUTICA I ÚS. Infusió (ús intern).

Litotríptic renal

FONT 2204. FORMA FARMACÈUTICA I ÚS. Infusió (ús intern). MODE D'UTILITZACIÓ/POSOLOGIA. Contra el mal de ronyons a causa de pedretes. Calia fer-ne una “novena”, és a dir, prendre durant nou dies seguits, cada matí i en dejú, una infusió d’herba morella roquera. DESTINACIÓ. Medicina humana.

**Part aèria**

Antihemorroidal

FONT 2204. FORMA FARMACÈUTICA I ÚS. Bany (ús extern). MODE D'UTILITZACIÓ/POSOLOGIA. Es bullia la planta i es posava en un gibrell i, col·locat estratègicament damunt d’una cadira, els afectats s’hi asseien una bona estona, almenys dues vegades al dia. DESTINACIÓ. Medicina humana.

Per a trastorns de la pell o del teixit subcutani

FONT 3952. DESCRIPCIÓ DE L'ÚS FETA PER L'INFORMANT. Externament s'usa amb molt bons resultats per a curar ferides, cremades, llavis i pell tallats o fissures anals. Es fa un cataplasma de la planta fresca matxucada, que s'aplica sobre la zona afectada. FORMA FARMACÈUTICA I ÚS. Cataplasma (ús extern). DESTINACIÓ. Medicina humana.

Per a trastorns del sistema genitourinari

FONT 3952. DESCRIPCIÓ DE L'ÚS FETA PER L'INFORMANT. Relaxa i desinflama els òrgans urinaris. FORMA FARMACÈUTICA I ÚS. Desconegut per l'informant / No consta. DESTINACIÓ. Medicina humana.

**ALTRES USOS**

**Fulla**

Ajuda a la llar

FONT 2226. DESCRIPCIÓ DE L'ÚS FETA PER L'INFORMANT. Per a netejar l’interior dels porrons de vi, amb aigua, ben esbaldit.

***Paspalum dilatatum***Poiret in Lam. (gramínies)
BCN 133005

**NOMS POPULARS**

Agram (2184)

Herba del gra d'arròs (2219)

Margall (2210)

***Paspalum distichum***L. (gramínies)
BCN 45022

**NOMS POPULARS**

Agram (2184)

Gram del Biscarri (2210)

***Passiflora edulis***Sims (passifloràcies)
BCN E-370

**NOMS POPULARS**

Fruita de la passió (fruit) (2200)

**USOS ALIMENTARIS**

**Fruit**

Ingestió de la part de la planta crua - Fresca (sense preparació)

FONT 2200. DESTINACIÓ. Alimentació humana.

***Pastinaca sativa***L. var. ***hortensis***Ehrh. (umbel·líferes)
BCN-E-255

**NOMS POPULARS**

Xirivia (2178, 2193, 2200, 2205, 2227)

**USOS ALIMENTARIS**

**Arrel**

No consta el tipus d'ingestió - No consta el mode de preparació

FONTS 2178, 2193, 2200, 2205, 2227. DESCRIPCIÓ DE L'ÚS FETA PELS INFORMANTS. Comestible (2227). DESTINACIÓ. Alimentació humana (2178, 2193, 2200, 2205, 2227).

***Pelargonium citrosum***Voigt ex Breiter (geraniàcies)
BCN 127881

**NOMS POPULARS**

Malva-rosa (2190)

**BARREGES AMB AQUEST TÀXON (vegeu catàleg de barreges)**

**USOS ALIMENTARIS**

**Fulla**

FONT 2190. Anís de cireres.

***Persea gratissima***Gaertn.f. (lauràcies)
BCN-E-230

**NOMS POPULARS**

*Aguacate* (castellà) (2199, 2200)

Alvocat (2201, 2205, 2207)

**USOS ALIMENTARIS**

**Fruit**

Ingestió de la part de la planta crua - Fresca (sense preparació)

FONTS 2199, 2200, 2201, 2205, 2207. DESCRIPCIÓ DE L'ÚS FETA PELS INFORMANTS. El fa servir per a les amanides. També per a untar sobre el pa (2207). DESTINACIÓ. Alimentació humana (2199, 2200, 2201, 2205, 2207).

***Petroselinum crispum***(Mill.) Hill (umbel·líferes)
BCN 127873

**NOMS POPULARS**

*Perejil* (castellà) (2180)

Julivert (2167, 2178, 2193, 2196, 2197, 2198, 2200, 2201, 2202, 3700, 2221, 3936, 2218)

**USOS MEDICINALS**

**Fulla**

Diürètic

FONT 3700. FORMA FARMACÈUTICA I ÚS. Sense forma farmacèutica (ús directe) (ús intern). PREPARACIÓ. Barrejada en el menjar. DESTINACIÓ. Medicina humana.

**USOS ALIMENTARIS**

**Arrel**

Condiment

FONT 3700. DESCRIPCIÓ DE L'ÚS FETA PER L'INFORMANT. S’utilitzen les arrels assecades al sol com a espècia. DESTINACIÓ. Alimentació humana.

**Fulla**

Condiment

FONTS 2167, 2178, 2221, 3700. DESCRIPCIÓ DE L'ÚS FETA PELS INFORMANTS. Es pot menjar fresc, acompanyant plats (3700). Per a guarnir plats (2178). DESTINACIÓ. Alimentació humana (2167, 2178, 2221, 3700).

No consta el tipus d'ingestió - No consta el mode de preparació

FONTS 2180, 2196, 2197, 2200, 2201, 2202, 2218, 3936. DESCRIPCIÓ DE L'ÚS FETA PELS INFORMANTS. Per a cuinar amb el peix (2180). Picat. En molts plats (2202). Comestible (2218). DESTINACIÓ. Alimentació humana (2180, 2196, 2197, 2200, 2201, 2202, 2218, 3936).

**Llavor**

Condiment

FONT 3700. DESCRIPCIÓ DE L'ÚS FETA PER L'INFORMANT. També s’utilitzen les llavors -prèviament assecades al sol- com a espècia. DESTINACIÓ. Alimentació humana.

**Part aèria**

No consta el tipus d'ingestió - No consta el mode de preparació

FONTS 2193, 2198. DESTINACIÓ. Alimentació humana.

**ALTRES OBSERVACIONS**

FONT 3700. DESCRIPCIÓ FETA PER L'INFORMANT. Si es plantava el matí de Divendres Sant, la planta no s’espigava en tot l’any.

***Phaseolus vulgaris***L. (papilionàcies)
BCN 126564

**NOMS POPULARS**

Bajoca (2204)

Mongeta (2190, 2193, 2199, 2202)

Mongeta (fruit) (2186)

Mongeta afartapobres (raça) (2204)

Mongeta blanca (2174, 2223)

Mongeta Bobi (raça) (2203)

Mongeta Carolina (raça) (2204)

Mongeta d'Alcanar (raça) (2204)

Mongeta de Buenos Aires (raça) (2193, 2204)

Mongeta de l'abundància (raça) (2204)

Mongeta de l'avellaneta (raça) (2204)

Mongeta de la mantega (raça) (2204)

Mongeta de la neu (raça) (2226)

Mongeta de mig dol (raça) (2192, 2221, 2212, 2218)

Mongeta de Petrushka (raça) (2204)

Mongeta del carall (raça) (2204)

Mongeta del cuc (raça) (2204, 2212, 2226)

Mongeta del cuc francès (raça) (2204)

Mongeta del ganxet (raça) (2191, 2194, 2202, 2204, 2216, 2217, 2218, 2226)

Mongeta del genoll de Crist (raça) (2204)

Mongeta del mig dol negre (raça) (2204)

Mongeta del mig dol ros (raça) (2204)

Mongeta del pic (raça) (2204)

Mongeta del rector (raça) (2204)

Mongeta escurçatites (raça) (2204)

Mongeta facciosa (raça) (2193, 2204, 2218, 2226)

Mongeta fina (raça) (2206)

Mongeta llaminera (raça) (2204)

Mongeta Perona (raça) (2164, 2203, 2204, 2218, 2226)

Mongeta seca (raça) (2218)

Mongeta tendra (2200, 2204, 2206, 3700, 2212, 2218)

Mongeta terrera del carall (raça) (2218)

Mongetera (2179, 2186, 2188, 2226)

**USOS ALIMENTARIS**

**Fruit**

Ingestió de la part de la planta cuita - Cuita en aigua

FONTS 2164, 2179, 2212. CONSUMICIÓ. Bullit (2164, 2179). DESTINACIÓ. Alimentació humana (2164, 2179, 2212).

No consta el tipus d'ingestió - No consta el mode de preparació

FONTS 2186, 2190, 2193, 2199, 2200, 2203, 2206, 3700. DESTINACIÓ. Alimentació humana.

**Llavor**

Ingestió de la part de la planta cuita - Cuita en aigua

FONTS 2194, 2218, 2221, 2226. DESCRIPCIÓ DE L'ÚS FETA PELS INFORMANTS. Es posa en aigua una nit i després es bull (2221). Se'n menja bullida (2218). Se’n mengen les llavors seques, un cop bullides (2226). DESTINACIÓ. Alimentació humana (2194, 2218, 2221, 2226).

Ingestió de la part de la planta cuita - Cuita en aigua i greix

FONT 2174. DESCRIPCIÓ DE L'ÚS FETA PER L'INFORMANT. En menja en potatges. DESTINACIÓ. Alimentació humana.

No consta el tipus d'ingestió - No consta el mode de preparació

FONTS 2191, 2202, 2216, 2217. DESTINACIÓ. Alimentació humana.

**ALTRES USOS**

**Llavor**

Elaboració d'obres artístiques

FONT 2223. DESCRIPCIÓ DE L'ÚS FETA PER L'INFORMANT. Per a l’elaboració de les paneres artístiques.

**ALTRES OBSERVACIONS**

FONTS 2192, 2212, 2216. DESCRIPCIÓ FETA PELS INFORMANTS. [Relatiu a la mongeta de mig dol (raça)] Raça desapareguda (2192). [Relatiu a la mongeta del cuc (raça)] Creixia molt i molt ràpidament. Se sembrava pel maig i durant estiu i tardor se’n collien mongetes dia sí, dia no. Ja desapareguda (2212). [Relatiu a la mongeta de mig dol (raça)] Era la més fina del mercat. Ja ha desaparegut. Es feien dues anyades: la primera se sembrava a finals d’hivern i durant tota la primavera es collia. Es destinava, sobretot, a l’exportació. La segona, a la tardor i de curta durada, es venia als mercats de Barcelona (2212). Abans se’n feia molt. Era la mongeta del Prat i se’n batien 15-20 mujades [unitat de mesura agrària equivalent a 4.896,5 m^2^] (2216).

***Phoenix dactylifera***L. (arecàcies)
BCN 52783

**NOMS POPULARS**

Dàtil (fruit) (2200)

Palma (producte elaborat) (3700)

Palmó (producte elaborat) (3700)

**USOS ALIMENTARIS**

**Fruit**

Ingestió de la part de la planta crua - Fresca (sense preparació)

FONT 2200. DESTINACIÓ. Alimentació humana.

**ALTRES USOS**

**Fulla**

Creences i pràctiques magicoreligioses

FONT 3700. DESCRIPCIÓ DE L'ÚS FETA PER L'INFORMANT. Les seves fulles servien per a fer les palmes i els palmons de Diumenge de Rams.

***Phormium tenax***Forst. (asparagàcies)
BCN 129015

**NOMS POPULARS**

Fòrnio (2211)

Fòrnius (2209, 2210)

Forpa (2190)

**ALTRES USOS**

**Fulla jove**

Agrosilvopastoral

FONTS 2190, 2209, 2210, 3946. DESCRIPCIÓ DE L'ÚS FETA PELS INFORMANTS. Per a fer lligalls i per a lligar canyes. S’agafa la fulla tendra i se’n treuen les fibres (2190). Les fibres serveixen per a lligar els enciams (2209). Les fibres de les fulles llargues serveixen per a lligar els enciams i també les tomaqueres que vagin pujant amunt per les canyes (2210). Per a lligar enciams (3946). OBSERVACIONS. Elaboració d'estris d'ús hortícola/agrícola (2190, 2209, 2210, 3946).

**Inflorescència**

Elaboració de rams

FONTS 2210, 2211. DESCRIPCIÓ DE L'ÚS FETA PER L'INFORMANT. Ornamental.

***Phragmites australis***(Cav.) Steudel (gramínies)
BCN 126573

**NOMS POPULARS**

Canyís (2167)

Canyota (2163)

**ALTRES USOS**

**Planta sencera**

Agrosilvopastoral

FONT 2163. DESCRIPCIÓ DE L'ÚS FETA PER L'INFORMANT. Per a reblir el terreny. OBSERVACIONS. Encoixinament.

**ALTRES OBSERVACIONS**

FONT 2167. DESCRIPCIÓ FETA PER L'INFORMANT. Es fica pertot arreu.

***Physalis alkekengi***L. (solanàcies)
BCN 100516

**NOMS POPULARS**

Fisalis (2200)

***Pimpinella anisum***L. (umbel·líferes)
BCN 47278

**NOMS POPULARS**

Anís (2182, 2228)

Anís verd (3932)

Matafaluga (2210, 3947)

**USOS MEDICINALS**

**Fruit**

Digestiu

FONTS 2182, 2210. FORMA FARMACÈUTICA I ÚS. Infusió (ús intern) (2182, 2210). MODE D'UTILITZACIÓ/POSOLOGIA. Després de dinar o sopar (2182). DESTINACIÓ. Medicina humana (2182, 2210).

Galactogen

FONT 3932. DESCRIPCIÓ DE L'ÚS FETA PER L'INFORMANT. Per a promoure la secreció de llet. FORMA FARMACÈUTICA I ÚS. Desconegut per l'informant / No consta. DESTINACIÓ. Medicina humana.

Per a les flatulències

FONT 3932. DESCRIPCIÓ DE L'ÚS FETA PER L'INFORMANT. Per a les flatulències. FORMA FARMACÈUTICA I ÚS. Infusió (ús intern). DESTINACIÓ.

Medicina humana.

Per a trastorns del sistema respiratori

FONT 2228. DESCRIPCIÓ DE L'ÚS FETA PER L'INFORMANT. Bafs per a malalties dels pulmons. FORMA FARMACÈUTICA I ÚS. Aerosol (ús intern). DESTINACIÓ. Medicina humana.

**BARREGES AMB AQUEST TÀXON (vegeu catàleg de barreges)**

**USOS ALIMENTARIS**

**Fruit**

FONT 3947. Pa de Sant Nicasi.

***Pinus halepensis***Mill. (pinàcies)
BCN 129014

**NOMS POPULARS**

Pi (2162, 2166, 2179, 2184, 2189, 2192, 2209, 2211, 2212, 2229)

**USOS MEDICINALS**

**Fulla**

Expectorant

FONT 2166. FORMA FARMACÈUTICA I ÚS. Xarop (ús intern). PREPARACIÓ. Xarop amb la decocció de les fulles i mel. DESTINACIÓ. Medicina humana.

**Part aèria jove**

Per al refredat

FONT 2229. DESCRIPCIÓ DE L'ÚS FETA PER L'INFORMANT. Els bafs de brots de pi són bons per als constipats. S’han de posar en nombre senar. FORMA FARMACÈUTICA I ÚS. Aerosol (ús intern). DESTINACIÓ. Medicina humana.

**ALTRES USOS**

**Planta viva *in situ***

Agrosilvopastoral

FONT 2179. DESCRIPCIÓ DE L'ÚS FETA PER L'INFORMANT. Per a aguantar les dunes de la costa. OBSERVACIONS. Elements paisatgístics.

**Tija amb fulles/branques**

Altres informacions

FONT 2212. DESCRIPCIÓ DE L'ÚS FETA PER L'INFORMANT. Una branca de pi penjada sobre el portal anunciava que allà hi havia un celler.

Construcció de vehicles amb rodes

FONT 2162. DESCRIPCIÓ DE L'ÚS FETA PER L'INFORMANT. Per a fer galgues per al carro.

Creences i pràctiques magicoreligioses

FONT 2184. DESCRIPCIÓ DE L'ÚS FETA PER L'INFORMANT. S'esqueixaven unes branques de pi de la Pineda del Delta en la nit de Sant Joan i es posava una a cada punta dels camps de melons, perquè així s'evitaven les pedregades.

Obtenció de combustible: llenya

FONT 2192. DESCRIPCIÓ DE L'ÚS FETA PER L'INFORMANT. Feien feixines o gavelles, que servien per a cremar als forns.

**Tronc**

Obtenció de materials per a la construcció

FONTS 2162, 2189. DESCRIPCIÓ DE L'ÚS FETA PELS INFORMANTS. Per a fer bigues i puntals (2162). Se’n feien bigues per a la construcció (2189).

**BARREGES AMB AQUEST TÀXON (vegeu catàleg de barreges)**

**USOS MEDICINALS**

**Gemma de la fulla**

FONT 2209, 2211. Esperit de vi d'herbes.

***Pinus pinea***L. (pinàcies)
BCN 130950

**NOMS POPULARS**

Pi (2166, 2192, 2204, 3700, 2221)

Pinyó (llavors) (2192)

**USOS MEDICINALS**

**Fulla**

Expectorant

FONT 2166. FORMA FARMACÈUTICA I ÚS. Xarop (ús intern). PREPARACIÓ. Xarop amb la decocció de les fulles i mel. DESTINACIÓ. Medicina humana.

**ALTRES USOS**

**Tija amb fulles/branques**

Creences i pràctiques magicoreligioses

FONT 3700. DESCRIPCIÓ DE L'ÚS FETA PER L'INFORMANT. La vigília de Sant Joan, durant la nit, alguns pagesos anaven a la platja a buscar una branca ben grossa de pi. D'aquesta, es tallaven branques curtes d’un o dos pams, sempre en nombre senar, i es portaven a un camp plantat de melons. Les branques es clavaven al voltant del camp per tal de protegir la collita. S’havia de fer abans que sortís el sol. Les branques només es treien quan es collien els melons.

**Tija defoliada**

Obtenció de combustible: llenya

FONT 2221.

**BARREGES AMB AQUEST TÀXON (vegeu catàleg de barreges)**

**USOS ALIMENTARIS**

**Llavor**

FONT 2192. Platillo de Sant Climent.

**USOS MEDICINALS**

**Escorça**

FONT 2204. Per al mal de queixal.

***Piper nigrum***L. (piperàcies)
BCN 47277

**NOMS POPULARS**Pebre (2212) **USOS ALIMENTARIS
Fruit**Condiment
FONT 2212. DESTINACIÓ. Alimentació humana.

**BARREGES AMB AQUEST TÀXON (vegeu catàleg de barreges)**

**USOS ALIMENTARIS**

**Fruit**

FONT 2212. Peus de porc amb naps.

***Pistacia lentiscus***L. (anacardiàcies)
BCN 129706

**NOMS POPULARS**

Llentiscle (2214)

**USOS ALIMENTARIS**

**Fulla**

Condiment

FONT 2214. DESCRIPCIÓ DE L'ÚS FETA PER L'INFORMANT. Per a aromatitzar el menjar. DESTINACIÓ. Alimentació humana.

No consta el tipus d'ingestió - No consta el mode de preparació

FONT 2214. DESCRIPCIÓ DE L'ÚS FETA PER L'INFORMANT. Antigament, per a fer xiclets. DESTINACIÓ. Alimentació humana.

Preparació de begudes - Beguda preparada amb licor

FONT 2214. DESCRIPCIÓ DE L'ÚS FETA PER L'INFORMANT. Per a fer licors. DESTINACIÓ. Alimentació humana.

**ALTRES USOS**

**Tija amb fulles/branques**

Elaboració de rams

FONT 2214. DESCRIPCIÓ DE L'ÚS FETA PER L'INFORMANT. Els marroquins l’agafen per a ús ornamental.

***Pisum sativum***L. (papilionàcies)
BCN-E-211

**NOMS POPULARS**

Pèsol (2178, 2190, 2193, 2194, 2200, 2202, 2205, 2206, 2208, 3700, 2225, 2218, 2227, 2226)

Pèsol caputxí (raça) (2226)

Pèsol negret (raça) (3700)

Tirabec (2205, 2226)

**USOS ALIMENTARIS**

**Fruit**

Ingestió de la part de la planta cuita - Cuita en aigua

FONT 2205. DESTINACIÓ. Alimentació humana.

**Llavor**

Ingestió de la part de la planta crua - Conservada dessecada a l'aire

FONT 3700. DESCRIPCIÓ DE L'ÚS FETA PELS INFORMANTS. Abans se'n menjava el gra sec. DESTINACIÓ. Alimentació humana.

Ingestió de la part de la planta cuita - Cuita en aigua

FONT 2208. DESCRIPCIÓ DE L'ÚS FETA PELS INFORMANTS. Es desgranen i es mengen bullits. DESTINACIÓ. Alimentació humana.

No consta el tipus d'ingestió - No consta el mode de preparació

FONTS 2178, 2190, 2193, 2194, 2200, 2206, 2218. DESCRIPCIÓ DE L'ÚS FETA PELS INFORMANTS. Se’n menja (2218). DESTINACIÓ. Alimentació humana (2178, 2190, 2193, 2194, 2200, 2206, 2218).

**ALTRES USOS**

**Fruit**

Elaboració d'obres artístiques

FONT 2225. DESCRIPCIÓ DE L'ÚS FETA PER L'INFORMANT. La beina del fruit serveix per a elaborar les paneres artístiques.

***Plantago afra***L. (plantaginàcies)
BCN 150357

**NOMS POPULARS**

Llavor de puça (2218)

**ALTRES USOS**

**Llavor**

Cosmètic

FONT 2218. DESCRIPCIÓ DE L'ÚS FETA PER L'INFORMANT. Ho compraven a una senyora. Eren unes llavors molt petites, que la seva mare bullia, colava i, quan es refredaven, quedaven convertides en una pasta tan espessa, que, estesa amb l’ajuda d’un raspall de dents gastat, servia de fixador per al cabell.

***Plantago lagopus***L. (plantaginàcies)
BCN 129697

**NOMS POPULARS**

Plantatge (2184, 2213)

**USOS MEDICINALS**

**Fulla**

Cicatritzant

FONT 2184. FORMA FARMACÈUTICA I ÚS. Sense forma farmacèutica (ús directe) (ús extern). DESTINACIÓ. Medicina humana.

***Plantago lanceolata***L. (plantaginàcies)
BCN 133004

**NOMS POPULARS**

Cap d'ase (2219)

Plantatge (2229)

Plantatge menor (3952)

**USOS MEDICINALS**

**Part aèria**

Per a la conjuntivitis

FONT 3952. DESCRIPCIÓ DE L'ÚS FETA PER L'INFORMANT. En rentats, la decocció alleugereix la conjuntivitis. FORMA FARMACÈUTICA I ÚS. Decocció (ús extern). DESTINACIÓ. Medicina humana.

Per al refredat

FONT 2229. DESCRIPCIÓ DE L'ÚS FETA PER L'INFORMANT. Per als constipats. FORMA FARMACÈUTICA I ÚS. Desconegut per l'informant / No consta. DESTINACIÓ. Medicina humana.

***Plantago major***L. (plantaginàcies)
BCN 133000

**NOMS POPULARS**

Llengua de vaca (2219)

***Pleurochaete squarrosa***(Brid.) Lindb. (pottiàcies)
BCN-Bryo-3959

**NOMS POPULARS**

Molsa (2188, 2189)

**ALTRES USOS**

**Part aèria**

Elaboració d'obres artístiques

FONTS 2188, 2189. DESCRIPCIÓ DE L'ÚS FETA PELS INFORMANTS. Per a fer els pessebres. OBSERVACIONS. Ornamental.

Recol·lecció per a la venda

FONTS 2188, 2189. DESCRIPCIÓ DE L'ÚS FETA PELS INFORMANTS. Es recollia abans de Nadal i es venia a la menuda (2189). Ornamental. En venien per a fer els pessebres (2188).

***Pleurotus ostreatus***(Jacq.) P.Kumm. (pleurotàcies)
BCN-E-273

**NOMS POPULARS**

Gírgola (2203)

**USOS ALIMENTARIS**

**Part aèria**

Ingestió de la part de la planta cuita - Cuita en oli

FONT 2203. DESTINACIÓ. Alimentació humana.

***Poa annua***L. (gramínies)
BCN 126555

**NOMS POPULARS**

Herba de jardí (2160, 2178)

***Polygonum aviculare***L. (poligonàcies)
BCN 126554

**NOMS POPULARS**

Estiravelles (2162)

Passacamins (2219)

Tiravell (2216)

Tiravella (2160)

Travacavalls (2219)

***Polypodium vulgare***L. subsp. ***serrulatum***Arcang. (polipodiàcies)
BCN 129709

**NOMS POPULARS**

Polipodi (2213)

**USOS MEDICINALS**

**Fulla**

Antibronquític

FONT 2213. FORMA FARMACÈUTICA I ÚS. Desconegut per l'informant / No consta. DESTINACIÓ. Medicina humana.

***Portulaca oleracea***L. (portulacàcies)
BCN 130962

**NOMS POPULARS**

Verdolaga (2160, 2161, 2163, 2162, 2172, 2175, 2179, 2184, 2204, 2208, 2211, 2215, 2216, 2217, 2224, 2231, 2218, 2226, 2228, 3933, 3934, 3936, 3939, 3946)

Vordolaga (2178, 2210, 2221, 2219)

**USOS MEDICINALS**

**Fulla**

Per a les picades d'abelles

FONT 3934. DESCRIPCIÓ DE L'ÚS FETA PER L'INFORMANT. Per a les picades de les abelles. FORMA FARMACÈUTICA I ÚS. Sense forma farmacèutica (ús directe) (ús extern). MODE D'UTILITZACIÓ/POSOLOGIA. Es frega la picada i s’alleuja el dolor. DESTINACIÓ. Medicina humana.

**Part aèria**

Hematocatàrtic

FONT 2228. DESCRIPCIÓ DE L'ÚS FETA PER L'INFORMANT. Depurativa. FORMA FARMACÈUTICA I ÚS. Desconegut per l'informant / No consta. DESTINACIÓ. Medicina humana.

**USOS ALIMENTARIS**

**Fulla**

Ingestió de la part de la planta crua - Fresca (sense preparació)

FONTS 2184, 2210, 2215, 3934. DESCRIPCIÓ DE L'ÚS FETA PELS INFORMANTS. Se'n menja (2215). En amanida (3934). CONSUMICIÓ. Amanida (2184, 2210, 3934). DESTINACIÓ. Alimentació humana (2184, 2210, 2215, 3934).

Ingestió de la part de la planta cuita - Cuita en oli

FONT 2218. DESCRIPCIÓ DE L'ÚS FETA PER L'INFORMANT. Se’n feien truites. CONSUMICIÓ. Truita. DESTINACIÓ. Alimentació humana.

No consta el tipus d'ingestió - No consta el mode de preparació

FONTS 2216, 2221, 2224, 2226. DESCRIPCIÓ DE L'ÚS FETA PELS INFORMANTS. Quan són tendres, les fulles es mengen (2221). Comestible (2224, 2226). DESTINACIÓ. Alimentació humana (2216, 2221, 2224, 2226).

**Llavor**

No consta el tipus d'ingestió - No consta el mode de preparació

FONT 2231. DESCRIPCIÓ DE L'ÚS FETA PER L'INFORMANT. Aliment per als canaris. DESTINACIÓ. Alimentació animal.

**Part aèria**

Ingestió de la part de la planta crua - Fresca (sense preparació)

FONTS 2160, 2161, 2162, 2163, 2172, 2204, 2208, 2211, 2219, 3936, 3946. DESCRIPCIÓ DE L'ÚS FETA PELS INFORMANTS. Per a amanides. No la conrea, però de vegades li’n demanen els seus clients (2163). Per a les vaques (2162). S’havia menjat en èpoques de guerra. Els pagesos no en menjaven, perquè sempre tenien coses millors per a menjar (2219). Per a les gallines (3936, 3946). CONSUMICIÓ. Amanida (2160, 2161, 2163, 2211). DESTINACIÓ. Alimentació humana (2160, 2161, 2163, 2172, 2204, 2208, 2211, 2219). Alimentació animal (2162, 3936, 3946).

Ingestió de la part de la planta cuita - Cuita en aigua

FONT 2175. DESCRIPCIÓ DE L'ÚS FETA PER L'INFORMANT. Per a fer sopa. DESTINACIÓ. Alimentació humana.

No consta el tipus d'ingestió - No consta el mode de preparació

FONTS 2217, 3933, 3939, 3946. DESCRIPCIÓ DE L'ÚS FETA PELS INFORMANTS. En donaven als porcs i els anava molt bé (2217). Per als porcs (3939). Se'n menja (3946). DESTINACIÓ. Alimentació animal (2217, 3933, 3939). Alimentació humana (3946).

Preparació de begudes - Beguda preparada amb aigua

FONT 2217. DESCRIPCIÓ DE L'ÚS FETA PER L'INFORMANT. Per a fer sucs. DESTINACIÓ. Alimentació humana.

***Prunus armeniaca***L. (rosàcies)
BCN-E-296

**NOMS POPULARS**

Abrecoc (fruit) (2218)

Albercoc (fruit) (2186, 2217)

Albercoquer (2210)

Bercoc (fruit) (2179, 2225)

**USOS ALIMENTARIS**

**Fruit**

Ingestió de la part de la planta crua - Fresca (sense preparació)

FONTS 2179, 2186, 2218, 2225. DESCRIPCIÓ DE L'ÚS FETA PELS INFORMANTS. Comestible (2225). DESTINACIÓ. Alimentació humana (2179, 2186, 2218, 2225).

Ingestió de la part de la planta cuita - Cuita en sucre

FONT 2217. DESCRIPCIÓ DE L'ÚS FETA PER L'INFORMANT. Per a fer melmelades. DESTINACIÓ. Alimentació humana.

***Prunus avium***(L.) L. (rosàcies)
BCN 126541

**NOMS POPULARS**

Cirera (fruit) (2179, 2187, 2188, 2189, 2191, 2192, 2200, 2206, 2208, 3700, 2215, 3939, 2231, 2225, 2228)

Cirera forta (raça) (2189)

Cirerer (2179, 2188, 2218, 2226, 2228)

**USOS MEDICINALS**

**Fruit**

Diürètic

FONT 2228. FORMA FARMACÈUTICA I ÚS. Desconegut per l'informant / No consta. DESTINACIÓ. Medicina humana.

Salutífer

FONT 2228. DESCRIPCIÓ DE L'ÚS FETA PER L'INFORMANT. Energètic i remineralitzant. FORMA FARMACÈUTICA I ÚS. Desconegut per l'informant / No consta. DESTINACIÓ. Medicina humana.

**Peduncle**

Diürètic

FONT 2231. DESCRIPCIÓ DE L'ÚS FETA PER L'INFORMANT. Els pecíols [dit “les cues”] dels fruits, en decocció, són útils per a fer pipí. FORMA FARMACÈUTICA I ÚS. Decocció (ús intern).

Protector renal

FONTS 2215, 2228. DESCRIPCIÓ DE L'ÚS FETA PER L'INFORMANT. El peduncle [dit “la cua seca de les cireres”], en infusió, és bo per al ronyó. El peduncle [dit “mànec”] és bo per als ronyons, en decocció. FORMA FARMACÈUTICA I ÚS. Infusió (ús intern) (2215). Decocció (ús intern) (2228). DESTINACIÓ. Medicina humana (2215, 2228).

**USOS ALIMENTARIS**

**Flor**

Ingestió de la part de la planta crua - Fresca (sense preparació)

FONT 2228. DESCRIPCIÓ DE L'ÚS FETA PER L'INFORMANT. Les flors en amanida. CONSUMICIÓ. Amanida. DESTINACIÓ. Alimentació humana.

**Fruit**

Ingestió de la part de la planta crua - Conservada dessecada a l'aire

FONT 2192. DESCRIPCIÓ DE L'ÚS FETA PER L'INFORMANT. Les seques són per fer el platillo (vegeu barreges alimentàries) i rostits. Un cop collides les cireres es posen 24 h a sol i serena amb un drap estès a terra. Passat aquest temps, s’aboca aigua bullint a sobre, quedant-se a sol i serena quaranta dies més. Es posen en caixes amb una malla per sobre per tal que no se les mengin les aus. Es recullen quan són ben seques. No s’han de guardar calentes perquè, si no, es podreixen. DESTINACIÓ. Alimentació humana.

Ingestió de la part de la planta crua - Conservada en licor

FONT 2189. DESTINACIÓ. Alimentació humana.

Ingestió de la part de la planta crua - Fresca (sense preparació)

FONTS 2179, 2188, 2189, 2192, 2200, 2206, 2208, 2218, 2225, 3939. DESCRIPCIÓ DE L'ÚS FETA PELS INFORMANTS. Comestible (2225). Se’n menja el fruit (2218). DESTINACIÓ. Alimentació humana (2179, 2188, 2189, 2192, 2200, 2206, 2208, 2218, 2225, 3939).

Ingestió de la part de la planta cuita - Cuita en sucre

FONT 2187. DESCRIPCIÓ DE L'ÚS FETA PER L'INFORMANT. Confitura. DESTINACIÓ. Alimentació humana.

Preparació de begudes - Beguda preparada amb vi

FONT 3700. DESCRIPCIÓ DE L'ÚS FETA PER L'INFORMANT. Per a fer arrop. DESTINACIÓ. Alimentació humana.

Preparació de begudes - Beguda preparada amb aiguardent

FONTS 2187, 2189, 2191. DESCRIPCIÓ DE L'ÚS FETA PELS INFORMANTS. Se'n fa licor de cirera (2187). Se’n feia anís de cirera (o aiguardent de cirera): te’n menjaves les cireres i te’n bevies el licor. Es guardava per a celebracions importants, tot i que després ningú no es recordava d’obrir-lo (2189). DESTINACIÓ. Alimentació humana (2187, 2189, 2191).

**BARREGES AMB AQUEST TÀXON (vegeu catàleg de barreges)**

**USOS ALIMENTARIS**

**Fruit**

FONT 2192. Anís de cireres.

FONT 3700. Arrop.

FONT 2192. Platillo de Sant Climent.

***Prunus cerasifera***Ehrh. (rosàcies)
BCN 127883

**NOMS POPULARS**

Mariana (2188)

Mirabolà (2160, 2162, 2192)

Mirabolà (fruit) (2216)

Pisardi (2204)

**USOS ALIMENTARIS**

**Fruit**

Ingestió de la part de la planta crua - Fresca (sense preparació)

FONT 2204, 2216. DESCRIPCIÓ DE L'ÚS FETA PER L'INFORMANT. Són molt dolços. N’anaven a buscar de silvestres a la Ricarda (2216). DESTINACIÓ. Alimentació humana (2204, 2216).

**ALTRES USOS**

**Planta viva *in situ***

Agrosilvopastoral

FONTS 2188, 2192. DESCRIPCIÓ DE L'ÚS FETA PER L'INFORMANT. Es fa servir com a peu per empeltar pruneres (2188). S’utilitza per a empeltar pruneres (2192).

***Prunus domestica***L. (rosàcies)
BCN-E-279

**NOMS POPULARS**

Pruna (fruit) (2188, 2192, 2204, 2211, 2212, 3939, 2225, 2218)

Pruna Clàudia (raça) (fruit) (2200, 2204, 2212, 2218)

Pruna colló de mico (raça) (fruit) (2204)

Pruna geniva (raça) (fruit) (2204)

Pruna groga (raça) (fruit) (2203, 2205)

Pruna japonesa (raça) (fruit) (2204, 2212, 2218)

Pruna Santa Rosa (raça) (fruit) (2218)

Pruna vermella (raça) (fruit) (2205)

Prunell (raça) (fruit) (2204)

Pruner (2179, 2218)

Prunera (2188, 2189, 2212)

**USOS ALIMENTARIS**

**Fruit**

Ingestió de la part de la planta crua - Fresca (sense preparació)

FONTS 2188, 2200, 2203, 2205, 2212, 2218, 2225, 3939. DESCRIPCIÓ DE L'ÚS FETA PELS INFORMANTS. Comestible (2225). Es mengen (2218). DESTINACIÓ. Alimentació humana (2188, 2200, 2203, 2205, 2212, 2218, 2225, 3939).

Ingestió de la part de la planta cuita - Cuita en sucre

FONT 2211, 2212. DESCRIPCIÓ DE L'ÚS FETA PER L'INFORMANT. Per a fer conserves (2211). Per a fer confitura (2212). DESTINACIÓ. Alimentació humana (2211, 2212).

**ALTRES USOS**

**Tija amb fulles/branques**

Obtenció de combustible: llenya

FONT 2179.

**BARREGES AMB AQUEST TÀXON (vegeu catàleg de barreges)**

**Fruit**

FONT 2192. Platillo de Sant Climent.

***Prunus dulcis***(Mill.) Weeb. (rosàcies)
BCN 126539

**NOMS POPULARS**

Ametlla (bessó/fruit) (2188, 2190, 2192)

Ametller (2160, 2188, 2190, 3936, 2226)

**USOS ALIMENTARIS**

**Bessó**

Ingestió de la part de la planta crua - Fresca (sense preparació)

FONTS 2188, 3936. DESTINACIÓ. Alimentació humana.

**Fruit**

Ingestió de la part de la planta cuita - Cuita en aigua

FONT 2190. DESCRIPCIÓ DE L'ÚS FETA PER L'INFORMANT. Es trenquen, es bullen una mica i s’hi posa sal. Es mengen sobretot en les postres. CONSUMICIÓ. Postres. DESTINACIÓ. Alimentació humana.

**BARREGES AMB AQUEST TÀXON (vegeu catàleg de barreges)**

**USOS ALIMENTARIS**

**Bessó**

FONT 2192. Platillo de Sant Climent.

***Prunus mahaleb***L. (rosàcies)
BCN 129711

**NOMS POPULARS**

Llúcia (2213)

Santa Llúcia (2188, 2189)

**ALTRES USOS**

**Planta viva *in situ***

Agrosilvopastoral

FONT 2188. DESCRIPCIÓ DE L'ÚS FETA PER L'INFORMANT. Es fa servir com a peu per a empeltar els cirerers. Es planta una Santa Llúcia, i quan és gruixuda, que té uns tres anys, es talla i s’hi posen dos eixarts amb pega calenta (2188). És el peu on es fa el cirerer. Tradicionalment, mai es planta directament un cirerer, sinó que es planta una Santa Llúcia i als tres anys es talla i s’hi posa un eixart de cirerer amb pega calenta (2189). És l’arbre on s’empelten els cirerers (2213).

***Prunus persica***(L.) Batsch (rosàcies)
BCN 46832

**NOMS POPULARS**

Nectarina (fruit) (2200)

Paraguaià (fruit) (2200)

Préssec (fruit) (2170, 2171, 2179, 2187, 2188, 2189, 2192, 2204, 2208, 2210, 3700, 2211, 2212, 2217, 3939, 2218)

Préssec belga (raça) (fruit) (2204)

Préssec benvingut (raça) (fruit) (2204)

Préssec d'aigua (fruit) (2202, 2205)

Préssec de llei (raça) (fruit) (2212)

Préssec de Sant Miquel (raça) (fruit) (2204)

Préssec escarolita (raça) (fruit) (2204)

Préssec gavatx de Sant Jaume (raça) (fruit) (2204)

Préssec groc (raça) (fruit) (2194)

Préssec hamzam (raça) (fruit) (2204)

Préssec roquet gelat (raça) (fruit) (2204)

Préssec teclo (raça) (fruit) (2204)

Préssec vermell (fruit) (2200, 2212)

Préssec vinya (fruit) (2205)

Presseguer (2170, 2171, 2178, 2179, 2184, 2186, 2188, 2189, 2212, 2219, 3939, 2225, 2226)

**USOS ALIMENTARIS**

**Fruit**

Ingestió de la part de la planta crua - Fresca (sense preparació)

FONTS 2170, 2171, 2179, 2188, 2192, 2194, 2200, 2202, 2212, 2225, 3939. DESCRIPCIÓ DE L'ÚS FETA PELS INFORMANTS. Se’n menja el fruit (2225). DESTINACIÓ. Alimentació humana (2170, 2171, 2179, 2188, 2192, 2194, 2200, 2202, 2212, 2225, 3939).

Ingestió de la part de la planta cuita - Cuita en sucre

FONTS 2187, 2189, 2192, 2208, 2212, 2217. DESCRIPCIÓ DE L'ÚS FETA PELS INFORMANTS. Se'n fa en almívar (2189). Melmelada (2208). Per a fer confitura (2187, 2189, 2212). Per a fer melmelades (2217). DESTINACIÓ. Alimentació humana (2187, 2189, 2192, 2208, 2212, 2217).

No consta el tipus d'ingestió - No consta el mode de preparació

FONTS 2205, 2210, 2211, 2218, 2219. DESCRIPCIÓ DE L'ÚS FETA PELS INFORMANTS. Per a fer conserves (2210, 2211). Se’n menja (2218). DESTINACIÓ. Alimentació humana (2205, 2210, 2211, 2218, 2219).

**ALTRES USOS**

**Tija**

Obtenció de combustible: llenya

FONT 2179.

***Prunus spinosa***L. (rosàcies)
BCN 156618

**NOMS POPULARS**

Prunell (fruit) (2212)

Pruneller (2212)

**USOS ALIMENTARIS**

**Fruit**

Preparació de begudes - Beguda preparada amb licor

FONT 2212. DESCRIPCIÓ DE L'ÚS FETA PER L'INFORMANT. Per a fer licors. DESTINACIÓ. Alimentació humana.

***Psidium guajava***L. (mirtàcies)
BCN-E-217

**NOMS POPULARS**
Guaiaba (fruit) (2200)

**USOS ALIMENTARIS**

**Fruit**

Ingestió de la part de la planta crua - Fresca (sense preparació)

FONT 2200. DESTINACIÓ. Alimentació humana.

***Punica granatum***L. (litràcies)
BCN 126538

**NOMS POPULARS**

*Granada* (fruit) (castellà) (2172, 2175, 2176, 2177)

Magrana (fruit) (2179, 2182, 2188, 2190, 2199, 2200, 2201, 2206, 2210, 2211)

Magraner (2179, 2182, 2188, 2190, 3700, 2218)

Mangraner (2184, 3936)

**USOS MEDICINALS**

**Arrel**

Antihelmíntic

FONT 2172. DESCRIPCIÓ DE L'ÚS FETA PER L'INFORMANT. L'escorça de l'arrel serveix per a combatre els cucs intestinals. FORMA FARMACÈUTICA I ÚS. Macerat en aigua (ús intern). PREPARACIÓ. Es bull aigua, es fica l'arrel i es deixa macerant 24 hores. El líquid es pren en dejú, cada 15 minuts. DESTINACIÓ. Medicina humana.

**Suc del fruit**

Antioxidant

FONTS 2176, 2177. FORMA FARMACÈUTICA I ÚS. Sense forma farmacèutica (ús directe) (ús intern). DESTINACIÓ. Medicina humana.

Per a prevenir el càncer

FONTS 2176, 2177. FORMA FARMACÈUTICA I ÚS. Sense forma farmacèutica (ús directe) (ús intern). DESTINACIÓ. Medicina humana.

**USOS ALIMENTARIS**

**Fruit**

Ingestió de la part de la planta crua - Fresca (sense preparació)

FONTS 2175, 2176, 2177, 2179, 2182, 2188, 2190, 2199, 2200, 2201, 2206, 2210, 2218, 3936. DESCRIPCIÓ DE L'ÚS FETA PELS INFORMANTS. Se’n menja el fruit (2218). DESTINACIÓ. Alimentació humana.

**ALTRES USOS**

**Tija**

Obtenció de combustible: llenya

FONT 2179.

**Tija amb fulles/branques**

Ajuda a la llar

FONTS 2184, 3700. DESCRIPCIÓ DE L'ÚS FETA PELS INFORMANTS. La llar de foc, calia netejar-la. Es feia amb branques de magraner perquè tenien moltes punxes i rascaven molt (3700). Per a fer caure el sutge de la xemeneia, perquè rascava amb les punxes (2184).

***Pyrus communis***L. subsp. ***communis***(rosàcies)
BCN 156582

**NOMS POPULARS**

Pera (fruit) (2179, 2192, 2200, 2204, 2212, 3933, 3939, 2218, 2227)

Pera blanquilla (raça) (fruit) (2202, 2206)

Pera camosina (raça) (fruit) (2204)

Pera camosina del gra (raça) (fruit) (2204)

Pera Campmanya (raça) (fruit) (2192, 2204)

Pera Castells (raça) (fruit) (2204, 2218)

Pera Conference (raça) (fruit) (2193, 2199, 2202, 2205)

Pera d'aigua (raça) (fruit) (2204)

Pera d'hivern (raça) (fruit) (2212)

Pera de la senyera catalana (raça) (fruit) (2204)

Pera de Sant Jaume (raça) (fruit) (2204, 2212)

Pera Forelle (raça) (fruit) (2205)

Pera llimonera (raça) (2193)

Pera moscatella (raça) (fruit) (2204)

Pera picona (raça) (fruit) (2204)

Pera rica (raça) (fruit) (2204)

Pera Rocha (raça) (fruit) (2205)

Pera santjoanes (raça) (fruit) (2204)

Pera tendral de Reus (raça) (fruit) (2204)

Perer (3933, 3939, 2225, 2218)

Perera (2179, 2184, 2212)

**USOS ALIMENTARIS**

**Fruit**

Ingestió de la part de la planta crua - Fresca (sense preparació)

FONTS 2179, 2193, 2200, 2206, 2212, 2225, 3939. DESCRIPCIÓ DE L'ÚS FETA PELS INFORMANTS. Es conservaven fins a Nadal si es penjaven al lloc més sec de la casa (2212). Se'n menja (3939). Se’n menja el fruit (2225). DESTINACIÓ. Alimentació humana (2179, 2193, 2200, 2206, 2212, 2225, 3939).

Ingestió de la part de la planta cuita - Cuita en sucre

FONTS 2192, 2212, 2227. DESCRIPCIÓ DE L'ÚS FETA PELS INFORMANTS. Melmelada (2192). Per a fer confitura (2212). DESTINACIÓ. Alimentació humana (2192, 2212). Per a fer confitura (2227). DESTINACIÓ. Alimentació humana (2192, 2212, 2227).

No consta el tipus d'ingestió - No consta el mode de preparació

FONTS 2199, 2202, 2205, 2218, 3933. DESCRIPCIÓ DE L'ÚS FETA PELS INFORMANTS. Se’n menja el fruit (2218). DESTINACIÓ. Alimentació humana (2199, 2202, 2205, 2218, 3933).

**ALTRES USOS**

**Tija**

Obtenció de combustible: llenya

FONT 2179.

***Pyrus malus***L. subsp. ***mitis***(Wallr.) O.Bolòs et J.Vigo (rosàcies)
BCN-E-205

**NOMS POPULARS**

Poma (fruit) (2179, 2187, 2188, 2189, 2192, 2200, 2204, 2208, 2211, 2215, 3933, 3939, 2227)

Poma àcida (fruit) (2206)

Poma camosa (raça) (fruit) (2192, 2204)

Poma de cara bruta (raça) (fruit) (2204)

Poma de gel (raça) (fruit) (2178)

Poma del ciri (raça) (fruit) (2192, 2204)

Poma Fuji (raça) (fruit) (2178, 2193, 2196, 2199, 2202, 2205)

Poma Golden (raça) (fruit) (2199, 2202, 2205)

Poma manyaga (raça) (fruit) (2192, 2204)

Poma mingueta (raça) (fruit) (2204)

Poma reineta (raça) (fruit) (2203, 2204)

Poma ronyosa (raça) (fruit) (2192)

Poma Royal Gala (raça) (fruit) (2193, 2202)

Poma Starking (raça) (fruit) (2193)

Poma ties (raça) (fruit) (2204)

Poma verd donzella (raça) (fruit) (2203, 2204)

Pomer (3700, 3933, 3939, 2225)

Pomera (2179, 2188, 2226)

**USOS MEDICINALS**

**Fruit**

Antidiarreic

FONT 2208. FORMA FARMACÈUTICA I ÚS. Sense forma farmacèutica (ús directe) (ús intern). PREPARACIÓ. Es pot fer crua ben triturada o cuita al microones o al forn. DESTINACIÓ. Medicina humana.

Estomacal

FONTS 2204, 2208. FORMA FARMACÈUTICA I ÚS. Sense forma farmacèutica (ús directe) (ús intern). PREPARACIÓ. Es pot fer crua ben triturada o cuita al microones o al forn (2208). DESTINACIÓ. Medicina humana.

**USOS ALIMENTARIS**

**Fruit**

Ingestió de la part de la planta crua - Fresca (sense preparació)

FONTS 2178, 2179, 2193, 2199, 2202, 2203, 2204, 2206, 2225, 3939. DESCRIPCIÓ DE L'ÚS FETA PELS INFORMANTS. [La poma de gel] té un aspecte de gelat a l'interior. Per madura que sigui, no queda seca (2178). Se'n menja (3939). Se’n menja el fruit (2225). DESTINACIÓ. Alimentació humana (2178, 2179, 2193, 2199, 2202, 2203, 2204, 2206, 2225, 3939).

Ingestió de la part de la planta cuita - Cuita sense vehicle

FONTS 2204, 2215. DESCRIPCIÓ DE L'ÚS FETA PELS INFORMANTS. Al forn (2204). Al vapor (2215). DESTINACIÓ. Alimentació humana (2204, 2215).

Ingestió de la part de la planta cuita - Cuita en sucre

FONTS 2187, 2189, 2192, 2225, 2227. DESCRIPCIÓ DE L'ÚS FETA PELS INFORMANTS. Confitura (2187, 2189). Melmelada (2192). En fa compota (2225). Per a fer confitura (2227). DESTINACIÓ. Alimentació humana (2187, 2189, 2192, 2225, 2227).

No consta el tipus d'ingestió - No consta el mode de preparació

FONTS 2188, 2196, 2200, 2205, 2211, 3933. DESCRIPCIÓ DE L'ÚS FETA PELS INFORMANTS. Per a fer conserves (2211). DESTINACIÓ. Alimentació humana (2188, 2196, 2200, 2205, 2211, 3933).

**ALTRES USOS**

**Tija**

Obtenció de combustible: llenya

FONT 2179.

***Quercus humilis***Mill. (fagàcies)
BCN 129002

**NOMS POPULARS**

Roure (2189)

**ALTRES USOS**

**Tronc**

Obtenció de combustible: carbó

FONT 2189. DESCRIPCIÓ DE L'ÚS FETA PER L'INFORMANT. Fa 100 anys, se'n feia aquí.

***Quercus ilex***L. (fagàcies)
BCN 129010

**NOMS POPULARS**

Alzina (2189, 2192)

**USOS ALIMENTARIS**

**Fruit**

Ingestió de la part de la planta crua - Fresca (sense preparació)

FONT 2192. DESCRIPCIÓ DE L'ÚS FETA PER L'INFORMANT. Per als porcs. DESTINACIÓ. Alimentació animal.

**ALTRES USOS**

**Tronc**

Obtenció de combustible: carbó

FONT 2189. DESCRIPCIÓ DE L'ÚS FETA PER L'INFORMANT. Fa 100 anys, se'n feia aquí.

***Quercus suber***L. (fagàcies)
BCN 46829

**NOMS POPULARS**

Suro (escorça) (2212)

Suro (producte elaborat) (2218)

**ALTRES USOS**

**Escorça**

Ajuda a la llar

FONT 2218. DESCRIPCIÓ DE L'ÚS FETA PER L'INFORMANT. Per a tapar les ampolles en el procés d’elaboració de conserva de tomàquet.

Elaboració d'estris de cuina

FONT 2212. DESCRIPCIÓ DE L'ÚS FETA PER L'INFORMANT. Per a tapar el canut de la sal.

***Ranunculus sardous***Crantz (ranunculàcies)
BCN 130956

**NOMS POPULARS**

Botó d'or (2213)

**ACCIONS NOCIVES O TÒXIQUES**

**No consta**

FONTS 2213, 2214. DESCRIPCIÓ DE L'ACCIÓ NOCIVA O TÒXICA FETA PELS INFORMANTS. És tòxica.

***Raphanus raphanistrum***L. subsp. ***sativus***(L.) Domin (crucíferes)
BCN 129005

**NOMS POPULARS**

Rave (2178, 2182, 2193, 2194, 2196, 2198, 2205, 2221, 2226)

Ravenet (3936)

**USOS ALIMENTARIS**

**Arrel**

Ingestió de la part de la planta crua - Fresca (sense preparació)

FONT 2178. DESTINACIÓ. Alimentació humana.

No consta el tipus d'ingestió - No consta el mode de preparació

FONTS 2182, 2193, 2194, 2196, 2198, 2205, 2221, 3936. DESTINACIÓ. Alimentació humana.

***Reichardia picroides***(L.) Roth (compostes)
BCN 113704

**NOMS POPULARS**

Coscúnia (2228)

**USOS ALIMENTARIS**

**Fulla**

Ingestió de la part de la planta crua - Fresca (sense preparació)

FONT 2228. DESCRIPCIÓ DE L'ÚS FETA PER L'INFORMANT. En amanida. CONSUMICIÓ. Amanida. DESTINACIÓ. Alimentació humana.

***Ricinus communis***L. (euforbiàcies)
BCN 46089

**NOMS POPULARS**

Ricí (2204)

**USOS MEDICINALS**

**Oli de la llavor**

Purgant

FONT 2204. FORMA FARMACÈUTICA I ÚS. Sense forma farmacèutica (ús directe) (ús intern). DESTINACIÓ. Medicina humana.

***Rorippa nasturtium-aquaticum***(L.) Hayek (crucíferes)
BCN 126579

**NOMS POPULARS**

*Berro de agua* (castellà) (2163)

Créixem (2187, 2191, 2192, 2211)

Creixen (2163, 2165)

**USOS MEDICINALS**

**Suc de la fulla**

Per a la intoxicació alcohòlica

FONT 2163. FORMA FARMACÈUTICA I ÚS. Sense forma farmacèutica (ús directe) (ús intern). MODE D'UTILITZACIÓ/POSOLOGIA. En batut, contra la ressaca. DESTINACIÓ. Medicina humana.

**USOS ALIMENTARIS**

**Part aèria**

Ingestió de la part de la planta crua - Fresca (sense preparació)

FONTS 2165, 2187, 2192. DESCRIPCIÓ DE L'ÚS FETA PELS INFORMANTS. Els recollien de les rieres per a vendre’ls a la menuda [al mercat] (2187). CONSUMICIÓ. Amanida (2165, 2187, 2192). DESTINACIÓ. Alimentació humana (2165, 2187, 2192).

Preparació de begudes - Beguda preparada amb aigua

FONT 2163. DESCRIPCIÓ DE L'ÚS FETA PER L'INFORMANT. Per a fer batuts contra la ressaca. DESTINACIÓ. Alimentació humana.

**Planta sencera**

Ingestió de la part de la planta crua - Fresca (sense preparació)

FONTS 2191, 2211. CONSUMICIÓ. Amanida (2211). DESTINACIÓ. Alimentació humana (2191, 2211).

***Rosa canina***L. (rosàcies)
BCN 156597

**NOMS POPULARS**

Gratacul (fruit) (3952)

Pica-pica (part del fruit) (3952)

Rosa (2228)

Roser (2229)

Roser silvestre (3952)

*Tapaculo* (fruit) (castellà) (2229)

**USOS MEDICINALS**

**Fruit**

Per al refredat

FONT 2229. DESCRIPCIÓ DE L'ÚS FETA PER L'INFORMANT. Els “*tapaculos*” serveixen per als constipats, tot i que, segons a qui, produeix picor i irritació. Cal fer una decocció del fruit. FORMA FARMACÈUTICA I ÚS. Decocció (ús intern). DESTINACIÓ. Medicina humana.

Tònic

FONT 3952. DESCRIPCIÓ DE L'ÚS FETA PER L'INFORMANT. Els fruits frescos són tonificants i combaten l’esgotament físic i l’astènia, i ajuden en les convalescències. FORMA FARMACÈUTICA I ÚS. Sense forma farmacèutica (ús directe) (ús intern). Decocció (ús intern). DESTINACIÓ. Medicina humana.

**No consta**

Per a la conjuntivitis

FONT 3952. DESCRIPCIÓ DE L'ÚS FETA PER L'INFORMANT. L’aigua de roses és bona per a tractar la conjuntivitis i la inflamació de les parpelles. FORMA FARMACÈUTICA I ÚS. Bany (ús extern). DESTINACIÓ. Medicina humana.

**Pètal**

Vulnerari

FONT 3952. DESCRIPCIÓ DE L'ÚS FETA PER L'INFORMANT. Es posa a macerar un grapat de pètals den un got d'aigua. Després d'un dia, cal esprémer-los i llençar-los. L'aigua serveix per a guarir les ferides. FORMA FARMACÈUTICA I ÚS. Bàlsam (ús extern). DESTINACIÓ. Medicina humana.

**USOS ALIMENTARIS**

**Pètal**

Ingestió de la part de la planta crua - Fresca (sense preparació)

FONT 2228. DESCRIPCIÓ DE L'ÚS FETA PER L'INFORMANT. Els pètals en amanida. CONSUMICIÓ. Amanida. DESTINACIÓ. Alimentació humana.

**ACCIONS NOCIVES O TÒXIQUES**

**Llavor**

FONT 3952. DESCRIPCIÓ DE L'ACCIÓ NOCIVA O TÒXICA FETA PER L'INFORMANT. No s’han de menjar les llavors de dintre del fruit. Els pèls de dintre dels fruits són coneguts com “pica-pica”, ja que provoquen una gran coïssor quan es posen entre el coll de la camisa i la pell. VIA D'INTOXICACIÓ. Externa.

**BARREGES AMB AQUEST TÀXON (vegeu catàleg de barreges)**

**USOS MEDICINALS**

**Flor**

FONT 2228. Oli de boixac i rosa.

***Rosmarinus officinalis***L. (labiades)
BCN 126559

**NOMS POPULARS**

Romaní (2162, 2167, 2183, 2184, 2187, 2188, 2189, 2190, 2191, 2192, 2194, 2195, 2204, 2209, 2210, 2221, 2211, 2215, 2216, 2222, 2217, 3932, 2224, 2232, 2229, 2218, 2227, 2226, 2228)

**USOS MEDICINALS**

**Fulla**

Antiinflamatori / Antiàlgic / Antiequimòtic

FONT 2204. FORMA FARMACÈUTICA I ÚS. Liniment (ús extern). MODE D'UTILITZACIÓ/POSOLOGIA. Per al dolor d’ossos i músculs. DESTINACIÓ.

Medicina humana.

**Part aèria**

Antiinflamatori / Antiàlgic / Antiequimòtic

FONT 2191. DESCRIPCIÓ DE L'ÚS FETA PER L'INFORMANT. Per als cops, es fan fregues d’alcohol amb romaní. FORMA FARMACÈUTICA I ÚS. Loció (ús extern). DESTINACIÓ. Medicina humana.

Per a trastorns del sistema sanguini

FONT 2190. DESCRIPCIÓ DE L'ÚS FETA PER L'INFORMANT. Rebaixa la sang. FORMA FARMACÈUTICA I ÚS. Infusió (ús intern). DESTINACIÓ. Medicina humana.

**Summitat florífera**

Hepatoprotector

FONT 3932. FORMA FARMACÈUTICA I ÚS. Desconegut per l'informant / No consta. DESTINACIÓ. Medicina humana.

**Tija amb fulles/branques**

Antiàlgic lumbar

FONT 2232. DESCRIPCIÓ DE L'ÚS FETA PER L'INFORMANT. Per a fer olis i fer fregues a l’esquena. És bo per a relaxar els músculs. FORMA FARMACÈUTICA I ÚS. Loció (ús extern). DESTINACIÓ. Medicina humana.

Antiinflamatori / Antiàlgic / Antiequimòtic

FONTS 2217, 2224. DESCRIPCIÓ DE L'ÚS FETA PELS INFORMANTS.

Macerat en oli, per a fer fregues que alleugen el dolor muscular (2224). FORMA FARMACÈUTICA I ÚS. Loció (ús extern). MODE D'UTILITZACIÓ/POSOLOGIA. Per a fer fregues (2217). DESTINACIÓ. Medicina humana.

Diürètic

FONT 2184. FORMA FARMACÈUTICA I ÚS. Infusió (ús intern). DESTINACIÓ. Medicina humana.

Per al refredat

FONT 2209. FORMA FARMACÈUTICA I ÚS. Infusió (ús intern). Aerosol (ús intern). DESTINACIÓ. Medicina humana.

Vasotònic

FONT 2229. DESCRIPCIÓ DE L'ÚS FETA PER L'INFORMANT. Bona per a la circulació de la sang. FORMA FARMACÈUTICA I ÚS. Desconegut per l'informant / No consta. DESTINACIÓ. Medicina humana.

**USOS ALIMENTARIS**

**Fulla**

Condiment

FONTS 2183, 2189, 2194, 2211, 2215, 2221. DESCRIPCIÓ DE L'ÚS FETA PELS INFORMANTS. Per posar gust a les salses. Com a condiment (2211). S’utilitza per a cuinar com a condiment (2221). En cuina, com a condiment de plats (2215). DESTINACIÓ. Alimentació humana (2183, 2189, 2194, 2211, 2215, 2221).

**Tija amb fulles/branques**

Condiment

FONTS 2187, 2191, 2195, 2210, 2216, 2217, 2226. DESCRIPCIÓ DE L'ÚS FETA PELS INFORMANTS. Com a condiment de carns rostides (2187). Confereix gust al menjar (2216). Per a donar sabor a la carn (2217). Té usos culinaris com a condiment (2226). DESTINACIÓ. Alimentació humana (2187, 2191, 2195, 2210, 2216, 2217, 2226).

**ALTRES USOS**

**Summitat florífera**

Elaboració de rams

FONT 2227. DESCRIPCIÓ DE L'ÚS FETA PER L'INFORMANT. Decoratiu. OBSERVACIONS. Decoratiu.

**BARREGES AMB AQUEST TÀXON (vegeu catàleg de barreges)**

**USOS ALIMENTARIS**

**Tija amb fulles/branques**

FONT 2192. Anís de cireres.

**USOS MEDICINALS**

**Tija amb fulles/branques**

FONT 2209, 2211. Esperit de vi d'herbes.

FONT 2204. Esperit de vi amb espígol i romaní.

**Fulla**

FONT 2204. Infusió de comí, romaní i sàlvia.

**Summitat florífera**

FONT 2228. Oli de romaní, ortiga i pericó.

FONT 2184. Infusió diürètica.

**Part aèria florida**

FONT 2162. Per al cos.

***Rubus ulmifolius***Schott (rosàcies)
BCN 126565

**NOMS POPULARS**

Esbarzer (2167, 2184, 2192)

Mora (fruit) (2208, 2222, 2225, 2227)

Sargués (2229)

Sargués (fruit) (2188, 2189, 2224, 3934)

**USOS MEDICINALS**

**Part aèria jove**

Per a l'afonia

FONT 2229. FORMA FARMACÈUTICA I ÚS. Infusió (ús intern). DESTINACIÓ. Medicina humana.

Per al refredat

FONT 2229. DESCRIPCIÓ DE L'ÚS FETA PER L'INFORMANT. El brot del sargués, en infusió, per a constipats i catarros. FORMA FARMACÈUTICA I ÚS. Infusió (ús intern). DESTINACIÓ. Medicina humana.

**USOS ALIMENTARIS**

**Fruit**

Ingestió de la part de la planta crua - Fresca (sense preparació)

FONTS 2188, 2224, 2225, 2227, 3934. DESCRIPCIÓ DE L'ÚS FETA PELS INFORMANTS. Comestible (2225, 2227). DESTINACIÓ. Alimentació humana (2188, 2224, 2225, 2227, 3934).

Ingestió de la part de la planta cuita - Cuita en sucre

FONTS 2198, 2208, 2222. DESCRIPCIÓ DE L'ÚS FETA PELS INFORMANTS. Melmelada (2208). Per a fer melmelades (2222). Per a fer confitura (3934). DESTINACIÓ. Alimentació humana (2208, 2222, 3934).

**ALTRES USOS**

**Part aèria**

Obtenció de combustible: llenya

FONT 2192. DESCRIPCIÓ DE L'ÚS FETA PER L'INFORMANT. Feien feixines o gavelles, que servien per a cremar als forns.

**Tija amb fulles/branques**

Ajuda a la llar

FONTS 2184, 3934. DESCRIPCIÓ DE L'ÚS FETA PELS INFORMANTS. Per a fer caure el sutge de la xemeneia. Es feia una feixa de branques d'esbarzer, es lligava amb un cordill i un estirava per amunt i un altre per avall per tal de netejar la xemeneia (2184). Per a netejar les xemeneies (3934).

***Rumex cristatus***DC. (poligonàcies)
BCN 130966

**NOMS POPULARS**

Anangadera (2216)

***Ruscus aculeatus***L. (asparagàcies)
BCN 128108

**NOMS POPULARS**

Galerà (2162, 2189, 2210)

**ALTRES USOS**

**Part aèria**

Elaboració de rams

FONTS 2189, 2210. DESCRIPCIÓ DE L'ÚS FETA PELS INFORMANTS. Ornamental (2210). Es collia per a vendre per Nadal (2189).

***Ruta graveolens***L. (rutàcies)
BCN 127876

**NOMS POPULARS**

Ruda (2184, 2190, 2204, 2209, 2210, 2211, 2213, 2214, 2217, 3937, 2231, 3952, 2229)

**USOS MEDICINALS**

**No consta**

Abortiu

FONTS 2214, 3952. FORMA FARMACÈUTICA I ÚS. Desconegut per l'informant / No consta. DESTINACIÓ. Medicina humana.

**Part aèria**

Antisèptic ocular

FONTS 2210, 2211. FORMA FARMACÈUTICA I ÚS. Bany (ús extern) (2211). Infusió (ús intern) (2210). MODE D'UTILITZACIÓ/POSOLOGIA. Per als ulls. Se’n fa un bany amb l’aigua de la infusió (2211). Per a netejar els ulls. Se’n fa una infusió i es renten els ulls amb l’aigua obtinguda (2210). DESTINACIÓ. Medicina humana.

Per al refredat

FONT 2184. FORMA FARMACÈUTICA I ÚS. Desconegut per l'informant / No consta. DESTINACIÓ. Medicina humana.

Tranquil·litzant

FONT 2190. FORMA FARMACÈUTICA I ÚS. Infusió (ús intern). DESTINACIÓ. Medicina humana.

**Tija amb fulles/branques**

Per a la conjuntivitis

FONT 2217. FORMA FARMACÈUTICA I ÚS. Bany (ús extern). MODE D'UTILITZACIÓ/POSOLOGIA. Es posa amb un cotó fluix mullat amb aigua del bullit de ruda. DESTINACIÓ. Medicina humana.

Per als ulls

FONTS 2204, 2209. FORMA FARMACÈUTICA I ÚS. Bany (ús extern). MODE D'UTILITZACIÓ/POSOLOGIA. Es bullia un branquilló de ruda i, amb un cotó fluix amarat d’aquella aigua tèbia, calia fer-ne fregues als ulls. D’aquesta manera, es guarien les infeccions superficials dels ulls (2204). DESTINACIÓ. Medicina humana.

**ALTRES USOS**

**No consta**

Creences i pràctiques magicoreligioses

FONT 2229. DESCRIPCIÓ DE L'ÚS FETA PER L'INFORMANT. Protectora de les famílies, expulsava el mal d’ull.

Literatura oral popular: llegendes, gloses, contes, dites, refranys, poemes, cançons

FONT 3937. DESCRIPCIÓ DE L'ÚS FETA PER L'INFORMANT. “Qui té ruda, Déu l’ajuda”.

**Planta sencera**

Repel·lent

FONT 2231. DESCRIPCIÓ DE L'ÚS FETA PER L'INFORMANT. Expulsa els insectes.

**ACCIONS NOCIVES O TÒXIQUES**

**No consta**

FONT 3952. DESCRIPCIÓ DE L'ACCIÓ NOCIVA O TÒXICA FETA PER L'INFORMANT. El contacte de la planta amb la pell, sobretot si aquesta ha sofert l'acció del sol, pot provocar reaccions al·lèrgiques. VIA D'INTOXICACIÓ. Externa.

**BARREGES AMB AQUEST TÀXON (vegeu catàleg de barreges)**

**USOS MEDICINALS**

**Part aèria**

FONT 2213. Cataplasma per a les hemorroides.

***Salicornia patula***Duval-Jouve (amarantàcies)
BCN 126576

**NOMS POPULARS**

Herba salada (2165)

Salicòrnia (2163)

**USOS ALIMENTARIS**

**Part aèria**

Ingestió de la part de la planta crua - Fresca (sense preparació)

FONTS 2163, 2165. DESCRIPCIÓ DE L'ÚS FETA PELS INFORMANTS. Se'n menja crua i serveix per a decorar plats (2163). DESTINACIÓ. Alimentació humana (2163, 2165).

***Salix fragilis***L. (salicàcies)
BCN 31305

**NOMS POPULARS**

Vima (2218)

Vímet (2218)

**ALTRES USOS**

**Tija**

Agrosilvopastoral

FONT 2218. DESCRIPCIÓ DE L'ÚS FETA PER L'INFORMANT. Per a lligar les canyes. Per a lligar els enciams. OBSERVACIONS. Elaboració d'estris d'ús hortícola/agrícola.

Ajuda a la llar

FONT 2218. DESCRIPCIÓ DE L'ÚS FETA PER L'INFORMANT. Per a estrènyer els tomàquets dins les ampolles en el procés d’elaboració de conserva.

***Salvia officinalis***L. subsp. ***officinalis***(labiades)
BCN 126558

**NOMS POPULARS**

Sàlvia (2167, 2187, 2189, 2191, 2204, 2210, 2231, 2229)

**USOS MEDICINALS**

**Fulla**

Antihipertensor

FONT 2229. FORMA FARMACÈUTICA I ÚS. Desconegut per l'informant / No consta. DESTINACIÓ. Medicina humana.

Carminatiu

FONT 2189. FORMA FARMACÈUTICA I ÚS. Desconegut per l'informant / No consta. DESTINACIÓ. Medicina humana.

Desconegut per a l'informant

FONT 2187. FORMA FARMACÈUTICA I ÚS. Infusió (ús intern). DESTINACIÓ. Medicina humana.

**USOS ALIMENTARIS**

**Fulla**

Condiment

FONT 2210, 2229. DESCRIPCIÓ DE L'ÚS FETA PER L'INFORMANT. Per al pollastre (2229). DESTINACIÓ. Alimentació humana (2210, 2229).

**ALTRES USOS**

**Fulla**

Cosmètic

FONTS 2191, 2231. DESCRIPCIÓ DE L'ÚS FETA PELS INFORMANTS. Amb les fulles es netejaven les dents, refregant-les [dit "restregant-les"] (2191). Per a fregar-se les dents (2231).

**Planta viva *in situ***

Agrosilvopastoral

FONT 2167. DESCRIPCIÓ DE L'ÚS FETA PER L'INFORMANT. Va bé per al camp. OBSERVACIONS. Associació de cultius i equilibri sistèmic.

**BARREGES AMB AQUEST TÀXON (vegeu catàleg de barreges)**

**Fulla**

FONT 2204. Infusió de comí, romaní i sàlvia.

***Sambucus nigra***L. (adoxàcies)
BCN 130934

**NOMS POPULARS**

Saüc (2182, 2184, 2204, 2217, 3932, 3952, 2229, 2218, 2226)

**USOS MEDICINALS**

**Fulla**

Per a la conjuntivitis

FONT 3952. DESCRIPCIÓ DE L'ÚS FETA PER L'INFORMANT. Conjuntivitis. S'apliquen compreses sobre els ulls i rentats oculars. Les fulles tenen propietats similars a les de les flors, encara que la infusió no és tan agradable per la seva olor més forta. FORMA FARMACÈUTICA I ÚS. Bany (ús extern). DESTINACIÓ. Medicina humana.

**Inflorescència**

Antitussigen

FONT 2184. FORMA FARMACÈUTICA I ÚS. Decocció (ús intern). DESTINACIÓ. Medicina humana.

Per als ulls

FONTS 2204, 2217, 2229. DESCRIPCIÓ DE L'ÚS FETA PELS INFORMANTS. Per rentar els ulls. Se’n fan bafs (2229). FORMA FARMACÈUTICA I ÚS. Boira (ús extern) (2204). Bany (ús extern) (2217). Aerosol (ús intern) (2229). MODE D'UTILITZACIÓ/POSOLOGIA. S’havia de col·locar unes brases mig apagades sobre una tapadora d’alumini girada. La tapadora es posava com si fos un plat a taula, i el malalt acostava els ulls a la tapadora i es tapava el cap amb un drap gran. Aleshores, ell mateix, col·locava les flors de saüc damunt les brases, amb els ulls ben oberts, per a aprofitar-ne els vapors (2204). DESTINACIÓ. Medicina humana.

Restauratiu visual

FONT 2182. DESCRIPCIÓ DE L'ÚS FETA PER L'INFORMANT. Feien bullir les flors i, la infusió resultant, la sucaven en draps, que es posaven sobre els ulls quan tenien cops d'aire (ulls vermells o aclucats com a conseqüència d'anar en bici o d'estar al camp). FORMA FARMACÈUTICA I ÚS. Bany (ús extern). DESTINACIÓ. Medicina humana.

**No consta**

Antipirètic (per a febre periòdica)

FONT 3952. DESCRIPCIÓ DE L'ÚS FETA PER L'INFORMANT. En el xarampió, la rubèola i l’escarlatina fa que baixi la febre en provocar la sudoració i eliminar toxines. FORMA FARMACÈUTICA I ÚS. Desconegut per l'informant / No consta. DESTINACIÓ. Medicina humana.

Per al refredat

FONT 3932. DESCRIPCIÓ DE L'ÚS FETA PER L'INFORMANT. Va bé per als refredats. FORMA FARMACÈUTICA I ÚS. Desconegut per l'informant / No consta. DESTINACIÓ. Medicina humana.

Resolutiu

FONT 3952. DESCRIPCIÓ DE L'ÚS FETA PER L'INFORMANT. En cas de furóncols donen molts bons resultats els rentats amb la infusió. FORMA FARMACÈUTICA I ÚS. Infusió (ús extern). Bany (ús extern). DESTINACIÓ. Medicina humana.

**USOS ALIMENTARIS**

**Fruit**

Ingestió de la part de la planta crua - Fresca (sense preparació)

FONT 3952. DESCRIPCIÓ DE L'ÚS FETA PER L'INFORMANT. Les baies ben madures són bones. DESTINACIÓ. Alimentació humana.

Ingestió de la part de la planta cuita - Cuita en sucre

FONT 3952. DESCRIPCIÓ DE L'ÚS FETA PER L'INFORMANT. Per a fer melmelada. DESTINACIÓ. Alimentació humana.

**Inflorescència**

Ingestió de la part de la planta cuita - Cuita en oli

FONTS 2229, 3952. DESCRIPCIÓ DE L'ÚS FETA PER L'INFORMANT. Per a fer bunyols (2229, 3952). DESTINACIÓ. Alimentació humana (2229, 3952).

**Suc del fruit**

Ingestió de la part de la planta cuita - Cuita en sucre

FONT 3952. DESCRIPCIÓ DE L'ÚS FETA PER L'INFORMANT. Es prepara un arrop de saüc amb els seus fruits. Es posa una part del suc dels fruits i dues de mel en una olla. Es bull fins que té la consistència de xarop. DESTINACIÓ. Alimentació humana.

**ALTRES USOS**

**Flor**

Elaboració de rams

FONT 2226. DESCRIPCIÓ DE L'ÚS FETA PER L'INFORMANT. Ornamental.

**Planta viva *in situ***

Agrosilvopastoral

FONT 3952. DESCRIPCIÓ DE L'ÚS FETA PER L'INFORMANT. Ara en queden ben pocs, però es podien trobar a les vores dels camins i a les ribes dels rius. El saüc millora la terra, accelera la descomposició de la matèria orgànica. OBSERVACIONS. Associació de cultius i equilibri sistèmic.

Creences i pràctiques magicoreligioses

FONT 3952. DESCRIPCIÓ DE L'ÚS FETA PER L'INFORMANT. Antigament se'l considerava un arbre màgic. El plantaven a prop de les cases, perquè protegia de les males influències tots els que hi vivien.

**Tija**

Elaboració d'instruments musicals

FONT 3952. DESCRIPCIÓ DE L'ÚS FETA PER L'INFORMANT. Abans la mainada en feia xiulets amb la fusta.

**ACCIONS NOCIVES O TÒXIQUES**

**Fruit**

FONT 3952. DESCRIPCIÓ DE L'ACCIÓ NOCIVA O TÒXICA FETA PER L'INFORMANT. Quan se’n mengen en massa quantitat poden arribar a ser tòxics. VIA D'INTOXICACIÓ. Interna.

**ALTRES OBSERVACIONS**

FONT 2182. DESCRIPCIÓ FETA PER L'INFORMANT. Actualment, ja no hi ha saüc a la zona.

**BARREGES AMB AQUEST TÀXON (vegeu catàleg de barreges)**

**USOS MEDICINALS**

**Inflorescència**

FONT 2204. Per al refredat.

***Santolina chamaecyparissus***L. (compostes)
BCN 113709

**NOMS POPULARS**

Espernallac (2209, 2210, 2211, 3937)

Espernallat femella (3935)

**USOS MEDICINALS**

**Inflorescència**

Antihelmíntic

FONT 3935. DESCRIPCIÓ DE L'ÚS FETA PER L'INFORMANT. Vermífug, es prepara en decocció, es té per costum posar-hi un parell de capítols [dit “caparrons”]. FORMA FARMACÈUTICA I ÚS. Decocció (ús intern). DESTINACIÓ. Medicina humana.

Digestiu

FONTS 2210, 2211. FORMA FARMACÈUTICA I ÚS. Infusió (ús intern). DESTINACIÓ. Medicina humana.

**Tija amb fulles/branques**

Antisèptic bucal

FONT 3937. DESCRIPCIÓ DE L'ÚS FETA PER L'INFORMANT. També serveix per a fer gàrgares quan es té algun problema a la boca. FORMA FARMACÈUTICA I ÚS. Col·lutori (ús extern). DESTINACIÓ. Medicina humana.

Vulnerari

FONT 3937. DESCRIPCIÓ DE L'ÚS FETA PER L'INFORMANT. Per a les ferides a la pell. FORMA FARMACÈUTICA I ÚS. Bany (ús extern). MODE D'UTILITZACIÓ/POSOLOGIA. Es fan banys amb un drap humitejat amb l’aigua del bull. DESTINACIÓ. Medicina humana.

**ALTRES USOS**

**Summitat florífera**

Ambientador

FONT 3935. DESCRIPCIÓ DE L'ÚS FETA PER L'INFORMANT. Es pot utilitzar per a fer saquets olorosos.

***Satureja calamintha***(L.) Scheele (labiades)
BCN 132991

**NOMS POPULARS**

*Almorahú* (castellà) (2174)

Rementerola (2192)

**USOS ALIMENTARIS**

**Tija amb fulles/branques**

Ingestió de la part de la planta crua - Conservada en salmorra

FONT 2174. DESCRIPCIÓ DE L'ÚS FETA PER L'INFORMANT. Adobat d'olives. DESTINACIÓ. Alimentació humana.

**ALTRES USOS**

**Part aèria**

Ajuda a la llar

FONT 2192. DESCRIPCIÓ DE L'ÚS FETA PER L'INFORMANT. La decocció es fa servir per netejar les botes de vi.

***Satureja fruticosa***(L.) Briq. (labiades)
BCN 125387

**NOMS POPULARS**

Poliol blanc (2222)

Poniol (2192)

**USOS MEDICINALS**

**Summitat florífera**

Digestiu

FONT 2222. FORMA FARMACÈUTICA I ÚS. Infusió (ús intern). DESTINACIÓ. Medicina humana.

**BARREGES AMB AQUEST TÀXON (vegeu catàleg de barreges)**

**USOS ALIMENTARIS**

**Part aèria**

FONT 2192. Anís de cireres.

***Satureja montana***L. (labiades)
BCN 130952

**NOMS POPULARS**

Sajolida (2184, 2187, 2188, 2189, 2190, 2192, 2206, 2209, 2210, 2222, 2229, 2225, 2227, 2228)

**USOS MEDICINALS**

**Part aèria**

Digestiu

FONT 2228. DESCRIPCIÓ DE L'ÚS FETA PER L'INFORMANT. És digestiu i estimula l’apetit. FORMA FARMACÈUTICA I ÚS. Infusió (ús intern). DESTINACIÓ. Medicina humana.

Estomacal

FONT 2209. FORMA FARMACÈUTICA I ÚS. Infusió (ús intern). DESTINACIÓ. Medicina humana.

Per a trastorns del sistema digestiu

FONT 2229. DESCRIPCIÓ DE L'ÚS FETA PER L'INFORMANT. Per al mal d’estómac. FORMA FARMACÈUTICA I ÚS. Desconegut per l'informant / No consta. DESTINACIÓ. Medicina humana.

Per al refredat

FONT 2209. FORMA FARMACÈUTICA I ÚS. Infusió (ús intern). DESTINACIÓ. Medicina humana.

**USOS ALIMENTARIS**

**Fulla**

Condiment

FONTS 2228, 2229. DESCRIPCIÓ DE L'ÚS FETA PELS INFORMANTS. Per a amanir la pasta (2228). OBSERVACIONS. Adobat d'olives (2229). DESTINACIÓ. Alimentació humana (2228, 2229).

No consta el tipus d'ingestió - No consta el mode de preparació

FONT 2228. DESCRIPCIÓ DE L'ÚS FETA PER L'INFORMANT. Per a l’adobat de les olives. OBSERVACIONS. Adobat d'olives. DESTINACIÓ. Alimentació humana.

**Tija amb fulles/branques**

Condiment

FONTS 2184, 2187, 2189, 2209, 2210, 2225, 2227. DESCRIPCIÓ DE L'ÚS FETA PELS INFORMANTS. Per a l'adobat d'olives (2184, 2187, 2189, 2209, 2210, 2225, 2227) DESTINACIÓ. Alimentació humana.

**BARREGES AMB AQUEST TÀXON (vegeu catàleg de barreges)**

**USOS ALIMENTARIS**

**Tija amb fulles/branques**

FONTS 2190, 2192. Olives arreglades.

***Scirpus holoschoenus***L. (ciperàcies)
BCN 132988

**NOMS POPULARS**

Jonc boval (2184)

**BARREGES AMB AQUEST TÀXON (vegeu catàleg de barreges)**

**USOS MEDICINALS**

**No consta**

FONT 2184. Per al refredat.

***Secale cereale***L. (gramínies)
BCN 46828

**NOMS POPULARS**

Sègol (2212)

**USOS ALIMENTARIS**

**Trituració del gra**

No consta el tipus d'ingestió - No consta el mode de preparació

FONT 2212. DESCRIPCIÓ DE L'ÚS FETA PER L'INFORMANT. Se’n cultivava pel gra per a fer farina. Conreu ja desaparegut. DESTINACIÓ. Alimentació humana.

**ALTRES USOS**

**Tija**

Agrosilvopastoral

FONT 2212. DESCRIPCIÓ DE L'ÚS FETA PER L'INFORMANT. Les seves tiges servien per a enforcar alls, cebes i tomàquets. OBSERVACIONS. Elaboració d'estris d'ús hortícola/agrícola.

***Sedum sediforme***(Jacq.) Pau (crassulàcies)
BCN 129708

**NOMS POPULARS**

Crespinell (2214)

***Setaria verticillata***(L.) Beauv. (gramínies)
BCN 133001

**NOMS POPULARS**

Panissola (2161, 3700, 2219)

***Silene vulgaris***(Moench) Garcke (cariofil·làcies)
BCN 126580

**NOMS POPULARS**

*Colleja* (castellà) (2165)

***Silybum marianum***(L.) Gaertn. (compostes)
BCN 129007

**NOMS POPULARS**

*Cardo mariano* (castellà) (2172)

Card (2161, 2178)

Card marià (3952)

Carxofa (inflorescència) (3952)

**USOS MEDICINALS**

**Flor i fulla**

Hepatoprotector

FONT 2172. DESCRIPCIÓ DE L'ÚS FETA PER L'INFORMANT. Neteja el fetge. FORMA FARMACÈUTICA I ÚS. Desconegut per l'informant / No consta. DESTINACIÓ. Medicina humana.

**USOS ALIMENTARIS**

**Arrel**

Ingestió de la part de la planta cuita - Cuita en aigua

FONT 3952. DESTINACIÓ. Alimentació humana.

**Fulla**

Ingestió de la part de la planta cuita - Cuita en aigua

FONT 2172. DESCRIPCIÓ DE L'ÚS FETA PER L'INFORMANT. Se'n pelen les fulles i se'n menja el nervi central. DESTINACIÓ. Alimentació humana.

**Fulla jove**

Ingestió de la part de la planta crua - Fresca (sense preparació)

FONT 3952. DESCRIPCIÓ DE L'ÚS FETA PER L'INFORMANT. Les fulles joves es preparen en amanides. CONSUMICIÓ. Amanida. DESTINACIÓ. Alimentació humana.

**Inflorescència**

Ingestió de la part de la planta cuita - Cuita en aigua

FONT 3952. DESTINACIÓ. Alimentació humana.

**Llavor**

Ingestió de la part de la planta crua - Fresca (sense preparació)

FONT 2178. DESCRIPCIÓ DE L'ÚS FETA PER L'INFORMANT. Per als ocells. DESTINACIÓ. Alimentació animal.

No consta el tipus d'ingestió - No consta el mode de preparació

FONT 3952. DESCRIPCIÓ DE L'ÚS FETA PER L'INFORMANT. Per als ocells. DESTINACIÓ. Alimentació animal.

**Planta sencera**

No consta el tipus d'ingestió - No consta el mode de preparació

FONT 3952. DESCRIPCIÓ DE L'ÚS FETA PER L'INFORMANT. Tota la planta triturada serveix de menjar per al bestiar. DESTINACIÓ. Alimentació animal.

**Tija**

Ingestió de la part de la planta cuita - Cuita en aigua

FONT 2161. DESCRIPCIÓ DE L'ÚS FETA PER L'INFORMANT. Es pelen i es mengen les tiges. DESTINACIÓ. Alimentació humana.

***Sisymbrium irio***L. (crucíferes)
BCN 129702

**USOS ALIMENTARIS**

**Llavor**

Ingestió de la part de la planta crua - Fresca (sense preparació)

FONT 2214. DESCRIPCIÓ DE L'ÚS FETA PER L'INFORMANT. Se’n mengen les llavors crues. DESTINACIÓ. Alimentació humana.

***Smilax aspera***L. (esmilacàcies)
BCN 130954

**NOMS POPULARS**

*Zarzaparrilla* (castellà) (2214)

**USOS MEDICINALS**

**Arrel**

Tònic

FONT 2214. FORMA FARMACÈUTICA I ÚS. Desconegut per l'informant / No consta. DESTINACIÓ. Medicina humana.

***Solanum lycopersicum***L. (solanàcies)
BCN-E-202

**NOMS POPULARS**

Cirerol (raça) (fruit) (3700)

Tomacó (raça) (fruit) (2205, 3700)

Tomaquera (2179, 2186, 2188, 2210, 3934)

Tomàquet (fruit) (2160, 2168, 2169, 2178, 2179, 2182, 2192, 2196, 2197, 2204, 2207, 2208, 2210, 3700, 2211, 2212, 2217, , 2218, 2227, 2226, 3934, 3936)

Tomàquet branca (fruit) (2202, 2203, 2205, 2206)

Tomàquet capollet (raça) (fruit) (2204)

Tomàquet caramba (raça) (fruit) (2204)

Tomàquet cor de bou (raça) (fruit) (2200, 2204)

Tomàquet Cherry (fruit) (raça) (2200, 2202, 2205)

Tomàquet d'amanida (fruit) (2205, 2221)

Tomàquet d'ampolla (fruit) (raça) (2198)

Tomàquet d'untar (fruit) (2194)

Tomàquet dàtil (raça) (fruit) (2204, 3700)

Tomàquet de Barbastro (raça) (fruit) (2202)

Tomàquet de conserva (raça) (fruit) (2192, 2204)

Tomàquet de pa (fruit) (2206)

Tomàquet de penjar (raça) (fruit) (2192, 2202, 2204, 2221)

Tomàquet francès (raça) (fruit) (2204)

Tomàquet geperut (raça) (fruit) (2192)

Tomàquet holandès (raça) (fruit) (2192, 2204)

Tomàquet Kumato (raça) (fruit) (2194)

Tomàquet madur (fruit) (2202)

Tomàquet Montserrat (raça) (fruit) (2192, 2196, 2198, 2204)

Tomàquet Mutxamel (raça) (fruit) (2198)

Tomàquet Palosanto (raça) (fruit) (2204, 3700)

Tomàquet pera (raça) (fruit) (2199, 2203, 2204, 3700, 2221)

Tomàquet poma (raça) (fruit) (2204)

Tomàquet poma gros (raça) (fruit) (2192)

Tomàquet poma verd (raça) (fruit) (2192)

Tomàquet pometa (raça) (fruit) (2204)

Tomàquet Raf (raça) (fruit) (2200, 2203, 2205)

Tomàquet rosa (raça) (fruit) (2200, 2204)

Tomàquet rosa de Barbastro (raça) (fruit) (2204)

Tomàquet tísic (raça) (fruit) (2204)

Tomàquet verd (fruit) (2202)

Tomàquet verd d'amanir (fruit) (2198)

Tomàquet vermell (fruit) (2220)

Tomata (fruit) (2193)

Tomata Cebrino (raça) (fruit) (2193)

Tomata de Barbastre (raça) (fruit) (2193)

Tomata de penjar (raça) (fruit) (2193)

Tomata Lobello (raça) (fruit) (2193)

Tomata Raf (raça) (fruit) (2193)

Tomata verda (fruit) (2193)

Tomata vermella (fruit) (2193)

**USOS ALIMENTARIS**

**Fruit**

Ingestió de la part de la planta crua - Conservada en oli

FONT 3936. DESTINACIÓ. Alimentació humana.

Ingestió de la part de la planta crua - Fresca (sense preparació)

FONTS 2160, 2168, 2169, 2179, 2194, 2204, 2212, 2217, 2220, 3700, 3934, 3936. DESCRIPCIÓ DE L'ÚS FETA PELS INFORMANTS. Se'n menja en amanides (2179). Molt bo per a menjar (2204). S’enforcaven amb trenes de sègol i es penjaven del sostre (2212). CONSUMICIÓ. Amanida (2168). DESTINACIÓ. Alimentació humana (2160, 2168, 2169, 2179, 2194, 2212, 2217, 2220, 3700, 3934, 3936).

Ingestió de la part de la planta cuita - Cuita sense vehicle

FONTS 2182, 2208, 2210, 2211, 2212, 2218, 2226, 2227. DESCRIPCIÓ DE L'ÚS FETA PELS INFORMANTS. Per a fer conserva (2182). Es feien en conserva. Se’ls hi treia la pell, es trinxaven i s’afegien uns pols que es compraven a la farmàcia (2208). Per a fer conserves. S’escalda el tomàquet, es pela, es fica dintre de pots, es tapa i al bany maria (2210). Per a fer conserves (2211). Per a fer conserves. Es trinxaven i amb el suc resultant s’omplien flascons juntament amb unes pólvores que es venien a la farmàcia. A la part de sobre es posaven uns dits d’oli. Més endavant es feia tapant hermèticament el flascó i al bany maria (2212). En feien conserva casolana (2218). Per a fer conserva (2226). Per a fer conserva (2227). DESTINACIÓ. Alimentació humana (2182, 2208, 2210, 2211, 2212, 2218, 2226, 2227).

Ingestió de la part de la planta cuita - Cuita en aigua

FONT 2218. DESCRIPCIÓ DE L'ÚS FETA PELS INFORMANTS. Els menjats pels cargols es bullien i es donaven de menjar al bestiar (2218). DESTINACIÓ. Alimentació animal.

Ingestió de la part de la planta cuita - Cuita en aigua i oli

FONTS 2179, 2207. DESCRIPCIÓ DE L'ÚS FETA PELS INFORMANTS. Per a fer salses (2179). Se'n fan salses (2207). DESTINACIÓ. Alimentació humana (2179, 2207).

Ingestió de la part de la planta cuita - Cuita en sucre

FONTS 2212, 2217, 3934. DESCRIPCIÓ DE L'ÚS FETA PELS INFORMANTS. Per a fer confitura (2212, 3934). Per a fer melmelades (2217). DESTINACIÓ. Alimentació humana (2212, 2217, 3934).

No consta el tipus d'ingestió - No consta el mode de preparació

FONTS 2178, 2193, 2196, 2197, 2198, 2199, 2200, 2202, 2203, 2205, 2206, ¡ 2221, 2227. DESTINACIÓ. Alimentació humana (2178, 2193, 2196, 2197, 2198, 2199, 2200, 2202, 2203, 2205, 2206, 2221, 2227).

Preparació de begudes - Beguda preparada per liquació

FONT 2179. DESCRIPCIÓ DE L'ÚS FETA PER L'INFORMANT. Per a fer gaspatxo. DESTINACIÓ. Alimentació humana.

**ALTRES USOS**

**Llavor**

Recol·lecció per a la venda

FONT 2218. DESCRIPCIÓ DE L'ÚS FETA PER L'INFORMANT. Extreien les llavors del fruit i les venien a distribuïdors de llavors de Barcelona. Com que els tomàquets, quan es collien, ja eren madurs, tenien molt de suc. Amb paciència, l’anàvem colant, per a separar-ne les llavors. Aquestes, posades en una borrassa al sol, les deixàvem assecar els dies que fes falta. Una vegada seques, les anàvem a vendre a Barcelona. El Cultivador Modern, Llavors Fitó o Llavors Nonell eren els compradors habituals.

**ALTRES OBSERVACIONS**

FONTS 2218, 3700. DESCRIPCIÓ FETA PELS INFORMANTS. S’ha d’esbrotar per tal que surtin bons tomàquets. S’arrencaven els brots que sortien a les vores de les fulles. Només en podien quedar dos, el principal i un altre (3700). Manera de fer tomàquet en conserva. Els tomàquets es pelaven i es tallaven en bocins petits i allargats per a facilitar el treball d’omplir les ampolles de gasosa que arreplegàvem durant tot l’any. Fèiem servir un bastó de vímet per a estrènyer el tomàquet ja dins de l’ampolla, perquè, si no s’estrenyia, després de la bullida quedava l’ampolla mig buida, i no era acceptable per a la venda. Tapàvem les ampolles amb taps de suro que compràvem a la ferreteria de la Rambla, es bullien i, encara calents, els fèiem servir per a tapar les ampolles. Després, posàvem un tros de sac al fons de la caldera, i hi posàvem les ampolles plenes i tapades per a bullir-les durant quinze minuts. Quedava així feta la conserva (2218). S’han d’enramar perquè si es deixa la planta rastrejar, els tomàquets es podreixen amb la humitat (2218).

**BARREGES AMB AQUEST TÀXON (vegeu catàleg de barreges)**

**USOS ALIMENTARIS**

**Fruit**

FONT 2192. Conserva de tomàquet.

***Solanum melongena***L. (solanàcies)
BCN-E-272

**NOMS POPULARS**

Albergínia (2178, 2194, 2199, 2200, 2201, 2205, 2210)

Albergínia (fruit) (2218)

Albergínia ratllada (raça) (fruit) (2203, 2205, 2220)

Esbergínia (2168, 2169, 2192, 2202, 3700, 2221, 3936, 2225, 2223)

**USOS ALIMENTARIS**

**Fruit**

Ingestió de la part de la planta cuita - Cuita sense vehicle

FONTS 2194, 2220, 2221. DESCRIPCIÓ DE L'ÚS FETA PELS INFORMANTS. Escalivada (2221). Al forn (2194). CONSUMICIÓ. Escalivada (2220). DESTINACIÓ. Alimentació humana (2194, 2220, 2221).

Ingestió de la part de la planta cuita - Cuita en oli

FONTS 2168, 2169, 2178, 2218, 2221. DESCRIPCIÓ DE L'ÚS FETA PELS INFORMANTS. Fregida (2221). Per a fer samfaina (2218). DESTINACIÓ. Alimentació humana (2168, 2169, 2178, 2218, 2221).

No consta el tipus d'ingestió - No consta el mode de preparació

FONTS 2199, 2200, 2201, 2202, 2203, 2205, 2210, 3700, 3936. DESTINACIÓ. Alimentació humana.

**ALTRES USOS**

**Epicarpi**

Elaboració d'obres artístiques

FONTS 2223, 2225. DESCRIPCIÓ DE L'ÚS FETA PELS INFORMANTS. La pell del fruit serveix per a elaborar les paneres artístiques (2225). La pell serveix per a l’elaboració de les paneres artístiques (2223).

**BARREGES AMB AQUEST TÀXON (vegeu catàleg de barreges)**

**USOS ALIMENTARIS**

**Fruit**

FONT 2192. Arrop.

***Solanum nigrum***L. (solanàcies)
BCN 127890

**NOMS POPULARS**

Tomaquet (2165, 2219)

**ACCIONS NOCIVES O TÒXIQUES**

**Fruit**

FONT 2165. DESCRIPCIÓ DE L'ACCIÓ NOCIVA O TÒXICA FETA PER L'INFORMANT. És una herbota tòxica. GRAU DE TOXICITAT. Indeterminat. VIA D'INTOXICACIÓ. Interna.

***Solanum tuberosum***L. (solanàcies)
BCN 29797

**NOMS POPULARS**

Patata (2170, 2171, 2178, 2182, 2194, 2195, 2197, 2204, 2210, 2221, 2212, 2217, 3936, 2225, 2218)

Patata Alfa (raça) (2204)

Patata anglesa (raça) (2212)

Patata Bintje (raça) (2204)

Patata blanca (2164, 2193, 2196, 2198, 2199, 2200, 2216)

Patata Kondor (raça) (2204)

Patata d'Àlaba (raça) (2204)

Patata de guardar (2167)

Patata del bufet (raça) (2204)

Patata Desirée (raça) (2204)

Patata Étoile de Lyon (raça) (2204)

Patata Eureka (raça) (2204)

Patata Furore (raça) (2204)

Patata Kennebec (raça) (2202, 2204, 2206)

Patata Monalisa (raça) (2206)

Patata morada (raça) (2200)

Patata Olalla (raça) (2204)

Patata Palogan (raça) (2204)

Patata platillo (raça) (2200)

Patata Red Pontiac (raça) (2202, 2204)

Patata Royal Kidney (raça) (2204)

Patata Spunta (raça) (2204)

Patata Túria (raça) (2204)

Patata vermella (2164, 2198, 2206, 2216)

Patata Víctor (raça) (2204)

Patatera (2204)

**USOS MEDICINALS**

**Tubercle**

Antipiròtic

FONT 2217. DESCRIPCIÓ DE L'ÚS FETA PER L'INFORMANT. Per a les cremades. FORMA FARMACÈUTICA I ÚS. Sense forma farmacèutica (ús directe) (ús intern). PREPARACIÓ. Es tallava per la meitat i s’aplicava directament. DESTINACIÓ. Medicina humana.

**USOS ALIMENTARIS**

**Tubercle**

Ingestió de la part de la planta cuita - Cuita sense vehicle

FONT 2218. DESCRIPCIÓ DE L'ÚS FETA PER L'INFORMANT. Es ficaven sota el foc durant una nit per a coure-les. DESTINACIÓ. Alimentació humana.

Ingestió de la part de la planta cuita - Cuita en aigua

FONTS 2164, 2167, 2182, 2212, 2216, 2218, 2221. DESCRIPCIÓ DE L'ÚS FETA PELS INFORMANTS. Bullida (2167). Per a guardar-les, es posaven a un racó de la porxada, fresc i fosc, tapades amb llençols fets amb roba de sac. La patata anglesa era la més primerenca i s’exportava a Anglaterra (2212). Es mengen bullides (2218). Les patates petites es bullien i es donaven de menjar al bestiar (2218). CONSUMICIÓ. Bullit (2182, 2221). DESTINACIÓ. Alimentació humana (2164, 2167, 2182, 2216, 2218, 2221). Alimentació humana i animal (2212). Alimentació animal (2218).

Ingestió de la part de la planta cuita - Cuita en oli

FONTS 2164, 2167, 2202, 2216. DESCRIPCIÓ DE L'ÚS FETA PELS INFORMANTS. Per a fer truita (2164). Per a fer truita o fregides (2167). CONSUMICIÓ. Truita (2164, 2167). DESTINACIÓ. Alimentació humana (2164, 2167, 2202, 2216).

No consta el tipus d'ingestió - No consta el mode de preparació

FONTS 2170, 2171, 2178, 2193, 2194, 2195, 2196, 2197, 2198, 2199, 2200, 2206, 2210, 2217, 3936. DESTINACIÓ. Alimentació humana (2170, 2171, 2178, 2193, 2194, 2195, 2196, 2197, 2198, 2199, 2200, 2206, 2217, 3936). Alimentació animal (2210).

**ALTRES USOS**

**Epicarpi**

Elaboració d'obres artístiques

FONT 2225. DESCRIPCIÓ DE L'ÚS FETA PER L'INFORMANT. La pell de la patata serveix per a elaborar les paneres artístiques.

**Fulla**

Fumable

FONT 2204. DESCRIPCIÓ DE L'ÚS FETA PER L'INFORMANT. Alguns homes a qui se’ls havia acabat el tabac trituraven fulles de patatera i ben seques se les fumaven.

***Sonchus oleraceus***L. (compostes)
BCN 126553

**NOMS POPULARS**

*Cerraja* (castellà) (2172)

Llecsó (2182, 2210, 2211, 2219, 2229)

Lletera (2210)

Lletsó (2160, 2162, 2178, 2192, 2204, 2216, 2228)

Llisó (2161, 2175, 2218)

**USOS MEDICINALS**

**No consta**

Per a trastorns del sistema digestiu

FONT 2172. DESCRIPCIÓ DE L'ÚS FETA PER L'INFORMANT. És depuratiu. FORMA FARMACÈUTICA I ÚS. Desconegut per l'informant / No consta. DESTINACIÓ. Medicina humana.

**USOS ALIMENTARIS**

**Fruit**

No consta el tipus d'ingestió - No consta el mode de preparació

FONT 2218. DESCRIPCIÓ DE L'ÚS FETA PER L'INFORMANT. Per als animals. DESTINACIÓ. Alimentació animal.

**Fulla**

Ingestió de la part de la planta crua - Conservada dessecada a l'aire

FONT 2216. DESCRIPCIÓ DE L'ÚS FETA PER L'INFORMANT. Es deixa pansir i l’endemà en donen als conills. DESTINACIÓ. Alimentació animal.

Ingestió de la part de la planta crua - Fresca (sense preparació)

FONTS 2172, 2182, 2204, 2210, 2211, 2228. DESCRIPCIÓ DE L'ÚS FETA PELS INFORMANTS. Per als conills (2182). CONSUMICIÓ. Amanida (2172, 2210, 2211, 2228). DESTINACIÓ. Alimentació humana (2172, 2204, 2210, 2211, 2228). Alimentació animal (2182).

**Fulla jove**

Ingestió de la part de la planta crua - Fresca (sense preparació)

FONT 2161. CONSUMICIÓ. Amanida. DESTINACIÓ. Alimentació humana.

**Part aèria**

Ingestió de la part de la planta crua - Conservada dessecada a l'aire

FONT 2160. DESCRIPCIÓ DE L'ÚS FETA PELS INFORMANTS. La gent que té conills els en dona, però s’ha de deixar pansir per tal que no tinguin mal de panxa. DESTINACIÓ. Alimentació animal.

Ingestió de la part de la planta crua - Fresca (sense preparació)

FONTS 2162, 2175, 2192, 2211. DESCRIPCIÓ DE L'ÚS FETA PELS INFORMANTS. Per als conills (2162, 2192, 2211). Per a conills i tortugues (2175). DESTINACIÓ. Alimentació animal (2162, 2175, 2192, 2211).

No consta el tipus d'ingestió - No consta el mode de preparació

FONTS 2210, 2229. DESCRIPCIÓ DE L'ÚS FETA PELS INFORMANTS. Per a menjar dels conills (2229). DESTINACIÓ. Alimentació animal (2210, 2229).

**ALTRES USOS**

**Planta viva *in situ***

Agrosilvopastoral

FONT 2178. DESCRIPCIÓ DE L'ÚS FETA PER L'INFORMANT. És font de fauna auxiliar. OBSERVACIONS. Associació de cultius i equilibri sistèmic.

***Sorbus domestica***L. (rosàcies)
BCN 130955

**NOMS POPULARS**

*Serbal* (castellà) (2213)

Serva (fruit) (2224)

Servera (2192)

**USOS ALIMENTARIS**

**Fruit**

Ingestió de la part de la planta crua - Fresca (sense preparació)

FONTS 2192, 2224. DESCRIPCIÓ DE L'ÚS FETA PELS INFORMANTS. Es mengen els fruits (2192). Comestible (2224). DESTINACIÓ. Alimentació

humana (2192, 2224).

**ALTRES USOS**

**Planta viva *in situ***

Agrosilvopastoral

FONT 2213. DESCRIPCIÓ DE L'ÚS FETA PER L'INFORMANT. S'està reintroduint per a augmentar la biodiversitat. OBSERVACIONS. Elements paisatgístics.

***Sorghum bicolor***(L.) Moench (gramínies)
BCN 31310

**NOMS POPULARS**

*Sordo* (castellà) (3700)

Sorgo (3700)

**USOS ALIMENTARIS**

**No consta**

No consta el tipus d'ingestió - No consta el mode de preparació

FONT 3700. DESTINACIÓ. Alimentació animal.

***Sorghum halepense***(L.) Pers. (gramínies)
BCN 133003

**NOMS POPULARS**

Canyota (2210, 2219)

***Spartium junceum***L. (papilionàcies)
BCN 127878

**NOMS POPULARS**

Ginesta (2187, 2189, 2214, 3937, 2227)

Ginestera (2190, 2192, 2204)

**USOS MEDICINALS**

**Flor**

Antiedematós

FONT 3937. DESCRIPCIÓ DE L'ÚS FETA PER L'INFORMANT. Serveix per a treure la inflamació de les cames. FORMA FARMACÈUTICA I ÚS. Bany (ús extern). PREPARACIÓ. Es bull, es mullen uns draps amb l’aigua del bull i s’hi emboliquen les extremitats inferiors. DESTINACIÓ. Medicina humana.

**ALTRES USOS**

**Flor**

Elaboració de rams

FONT 2227. DESCRIPCIÓ DE L'ÚS FETA PER L'INFORMANT. Per a les catifes florals. OBSERVACIONS. Ornamental.

**Fulla jove**

Agrosilvopastoral

FONT 2189, 2190, 2192. DESCRIPCIÓ DE L'ÚS FETA PER L'INFORMANT. Es feia servir per a fer lligalls per als enciams (2189). Per a fer lligalls (2190, 2192). OBSERVACIONS. Elaboració d'estris d'ús hortícola/agrícola (2189, 2190, 2192).

**Part aèria**

Elaboració d'escombres

FONT 2214.

**Part aèria florida**

Creences i pràctiques magicoreligioses

FONT 2204. DESCRIPCIÓ DE L'ÚS FETA PER L'INFORMANT. Els joves anaven a la muntanya el dia abans del Corpus a buscar-ne. El dia de Corpus, de matinada, ben proveïts de flors grogues de ginestera, preparaven l’altar.

Elaboració de rams

FONT 2192. DESCRIPCIÓ DE L'ÚS FETA PER L'INFORMANT. Ornamental. Es collien les branques florides per vendre.

***Spinacia oleracea***L. (amarantàcies)

BCN 129688

**NOMS POPULARS**

Espinac (2178, 2190, 2191, 2193, 2194, 2195, 2198, 2200, 2202, 2203, 2210, 3700, 2218, 2227, 2228)

**USOS ALIMENTARIS**

**Fulla**

Ingestió de la part de la planta crua - Fresca (sense preparació)

FONT 3700. CONSUMICIÓ. Amanida. DESTINACIÓ. Alimentació humana.

Ingestió de la part de la planta cuita - Cuita en aigua

FONTS 2210, 2228. DESCRIPCIÓ DE L'ÚS FETA PELS INFORMANTS. Per a fer caldo (2228). DESTINACIÓ. Alimentació humana (2210, 2228).

No consta el tipus d'ingestió - No consta el mode de preparació

FONTS 2190, 2191, 2193, 2194, 2195, 2200, 2202, 2203, 2218, 2227. DESCRIPCIÓ DE L'ÚS FETA PELS INFORMANTS. Se’n mengen (2218). Comestible (2227). DESTINACIÓ. Alimentació humana (2190, 2191, 2193, 2194, 2195, 2200, 2202, 2203, 2218, 2227).

**Part aèria**

No consta el tipus d'ingestió - No consta el mode de preparació

FONTS 2178, 2198. DESTINACIÓ. Alimentació humana.

**BARREGES AMB AQUEST TÀXON (vegeu catàleg de barreges)**

**USOS ALIMENTARIS**

**Fulla**

FONT 2228. Caldo depuratiu.

***Stachys officinalis***(L.) Trevisan (labiades)
BCN 25011

**NOMS POPULARS**

Herba brotònica (2204)

**USOS MEDICINALS**

**No consta**

Antihipertensor

FONT 2204. FORMA FARMACÈUTICA I ÚS. Infusió (ús intern). DESTINACIÓ. Medicina humana.

**ALTRES OBSERVACIONS**

FONT 2204. DESCRIPCIÓ FETA PER L'INFORMANT. L’herba brotònica només neix i creix a prop de les soques dels garrofers vius.

***Stellaria media***(L.) Vill. (cariofil·làcies)
BCN 126556

**NOMS POPULARS**

Gallinassa (2160, 2178, 2216)

Gallinassa d'hivern (2210)

Herba gallinera (2178)

**USOS ALIMENTARIS**

**Part aèria**

Ingestió de la part de la planta crua - Fresca (sense preparació)

FONT 2214. CONSUMICIÓ. Amanida. DESTINACIÓ. Alimentació humana.

**Planta sencera**

No consta el tipus d'ingestió - No consta el mode de preparació

FONT 2160. DESCRIPCIÓ DE L'ÚS FETA PER L'INFORMANT. A les gallines els encanta i la gent els en donava. DESTINACIÓ. Alimentació animal.

***Stevia rebaudiana***(Bertoni) Bertoni (compostes)
BCN 30644

**NOMS POPULARS**

Estèvia (2222)

**USOS ALIMENTARIS**

**Fulla**

Condiment

FONT 2222. DESCRIPCIÓ DE L'ÚS FETA PER L'INFORMANT. Edulcorant. DESTINACIÓ. Alimentació humana.

***Symphytum officinale***L. (boraginàcies)
BCN 126537

**NOMS POPULARS**

Consolda (2164, 2179)

**ALTRES USOS**

**Fulla**

Agrosilvopastoral

FONT 2179. DESCRIPCIÓ DE L'ÚS FETA PER L'INFORMANT. Les fulles tallades es posen sobre la terra com a adob verd. Posada sobre la terra, no deixa créixer massa el gra ni les males herbes. OBSERVACIONS. Adobs i fertilitzants naturals. Encoixinament.

**Part aèria**

Agrosilvopastoral

FONT 2164. OBSERVACIONS. Adobs i fertilitzants naturals. Encoixinament.

**Planta viva *in situ***

Agrosilvopastoral

FONT 2179. DESCRIPCIÓ DE L'ÚS FETA PER L'INFORMANT. Agafa els minerals de set o vuit metres avall i els tira cap amunt. No fa cap perjudici a les plantacions. OBSERVACIONS. Associació de cultius i equilibri sistèmic.

***Syringa vulgaris***L. (oleàcies)
BCN 132995

**NOMS POPULARS**

Lilà (2189, 2192, 2218)

**ALTRES USOS**

**Part aèria florida**

Recol·lecció per a la venda

FONTS 2189, 2192. DESCRIPCIÓ DE L'ÚS FETA PELS INFORMANTS. Se'n collien les branques florides per a vendre en manats (2189). Ornamental. Se'n collien les branques florides per a vendre (2192).

***Syzygium aromaticum***(L.) Merr. et Perry (mirtàcies)
BCN 47279

**NOMS POPULARS**

Clau (2228)

**USOS MEDICINALS**

**Poncella**

Antiodontàlgic

FONT 2228. DESCRIPCIÓ DE L'ÚS FETA PER L'INFORMANT. Anestèsic. És bo per al mal de queixal. Es tritura i es posa a la boca. FORMA FARMACÈUTICA I ÚS. Pólvores simples (ús extern). DESTINACIÓ. Medicina humana.

***Tamarindus indica***L. (papilionàcies)
BCN-E-603

**NOMS POPULARS**

Tamarinde (2200)

**USOS ALIMENTARIS**

**Mesocarpi**

No consta el tipus d'ingestió - No consta el mode de preparació

FONT 2200. DESTINACIÓ. Alimentació humana.

***Taraxacum officinale***Weber in Wiggers (compostes)
BCN 150371

**NOMS POPULARS**

Dent de lleó (3932)

Pixallits (2219, 2229, 2228)

**USOS MEDICINALS**

**Fulla**

Hepatoprotector

FONT 2228. DESCRIPCIÓ DE L'ÚS FETA PER L'INFORMANT. Bo per al fetge. FORMA FARMACÈUTICA I ÚS. Infusió (ús intern). DESTINACIÓ. Medicina humana.

**Fulla jove**

Diürètic

FONT 2229. DESCRIPCIÓ DE L'ÚS FETA PER L'INFORMANT. Diürètic. Se'n menja cru. FORMA FARMACÈUTICA I ÚS. Sense forma farmacèutica (ús directe) (ús intern). DESTINACIÓ. Medicina humana.

**Inflorescència**

Diürètic

FONT 3932. FORMA FARMACÈUTICA I ÚS. Infusió (ús intern). DESTINACIÓ. Medicina humana.

Hepatoprotector

FONT 2228. DESCRIPCIÓ DE L'ÚS FETA PER L'INFORMANT. Bo per al fetge. FORMA FARMACÈUTICA I ÚS. Infusió (ús intern). DESTINACIÓ. Medicina humana.

***Thymelaea hirsuta***(L.) Endl. (timeleàcies)
BCN 96988

**NOMS POPULARS**

Bufalaga (2166)

**USOS MEDICINALS**

**Part aèria**

Antisèptic gingival

FONT 2166. DESCRIPCIÓ DE L'ÚS FETA PER L'INFORMANT. Bullida serveix per a glopejar i per a desinflamar les genives. FORMA FARMACÈUTICA I ÚS. Gargarisme (ús extern). PREPARACIÓ. Bullida. Fer gàrgares amb el líquid d'ebullició. DESTINACIÓ. Medicina humana.

***Thymus vulgaris***L. (labiades)
BCN 126560

**NOMS POPULARS**

Farigola (2162, 2167, 2181, 2182, 2183, 2184, 2187, 2188, 2189, 2190, 2191, 2192, 2195, 2204, 2206, 2207, 2209, 2210, 2221, 2211, 2214, 2215, 2216, 2222, 2217, 3932, 2224, 2198, 2229, 2225, 2218, 2226, 2228, 3934, 3946)

Timó (2184)

**USOS MEDICINALS**

**No consta**

Antiinflamatori faringi

FONT 2221. FORMA FARMACÈUTICA I ÚS. Gargarisme (ús extern). DESTINACIÓ. Medicina humana.

Antiinflamatori intestinal

FONT 2218. DESCRIPCIÓ DE L'ÚS FETA PER L'INFORMANT. Per al mal de panxa. FORMA FARMACÈUTICA I ÚS. Sense forma farmacèutica (ús directe) (ús intern). PREPARACIÓ. En sopa. DESTINACIÓ. Medicina humana.

Antipirètic

FONT 2209. FORMA FARMACÈUTICA I ÚS. Infusió (ús extern). MODE D'UTILITZACIÓ/POSOLOGIA. L’aigua timolada [infusió] servia per a rebaixar la febre. Es mullava un drap en l'aigua timolada i es posava al front. DESTINACIÓ. Medicina humana.

Antisèptic bucal

FONT 2183. FORMA FARMACÈUTICA I ÚS. Gargarisme (ús extern). DESTINACIÓ. Medicina humana.

Antisèptic ocular

FONT 2183. FORMA FARMACÈUTICA I ÚS. Bany (ús extern). DESTINACIÓ. Medicina humana.

**Part aèria**

Anticefalàlgic

FONT 2184. DESCRIPCIÓ DE L'ÚS FETA PER L'INFORMANT. Se'n feia aigua timolada, se’n mullava un drap i es posava al front quan tenies mal de cap. FORMA FARMACÈUTICA I ÚS. Infusió (ús extern). DESTINACIÓ. Medicina humana.

Antisèptic extern

FONTS 2188, 2204. DESCRIPCIÓ DE L'ÚS FETA PELS INFORMANTS. Per a curar ferides obertes o talls, calia fer-ne rentades amb aigua de farigola. Es bullia aigua amb farigola i amb cotó fluix es desinfectava la ferida (2204). FORMA FARMACÈUTICA I ÚS. Decocció (ús extern) (2188). Infusió (ús extern) (2204). MODE D'UTILITZACIÓ/POSOLOGIA. Com a desinfectant. S’assecava, es bullia i l’aigua servia per a netejar ferides (2188). DESTINACIÓ. Medicina humana.

Antitussigen

FONT 2190. FORMA FARMACÈUTICA I ÚS. Infusió (ús intern). DESTINACIÓ. Medicina humana.

Diürètic

FONT 2184. FORMA FARMACÈUTICA I ÚS. Infusió (ús intern). DESTINACIÓ. Medicina humana.

Estomacal

FONT 2184. FORMA FARMACÈUTICA I ÚS. Infusió (ús intern). DESTINACIÓ. Medicina humana.

Per a la conjuntivitis

FONT 2210. FORMA FARMACÈUTICA I ÚS. Infusió (ús extern). MODE D'UTILITZACIÓ/POSOLOGIA. Per a fer aigua timolada. Per a la conjuntivitis, gases amb aigua timolada sobre els ulls. DESTINACIÓ. Medicina humana.

Per a trastorns del sistema digestiu

FONT 2188. FORMA FARMACÈUTICA I ÚS. Suspensió (ús intern). MODE D'UTILITZACIÓ/POSOLOGIA. En sopes era depuratiu. DESTINACIÓ. Medicina humana.

Per a trastorns del sistema sensorial

FONTS 2192, 2204. FORMA FARMACÈUTICA I ÚS. Infusió (ús extern). MODE D'UTILITZACIÓ/POSOLOGIA. Per a desfer els taps de les orelles (2192). L’aigua de farigola també servia per a guarir el mal d’orella. Calia agafar una xeringa, amb una pera de goma i una cànula, i introduir el líquid a l’orella (2204). DESTINACIÓ. Medicina humana.

Per al refredat

FONTS 2190, 2192. FORMA FARMACÈUTICA I ÚS. Infusió (ús intern). DESTINACIÓ. Medicina humana.

Restauratiu visual

FONT 2192. FORMA FARMACÈUTICA I ÚS. Infusió (ús extern). DESTINACIÓ. Medicina humana.

Tranquil·litzant

FONT 2190. FORMA FARMACÈUTICA I ÚS. Infusió (ús intern). DESTINACIÓ. Medicina humana.

**Part aèria florida**

Antiodontàlgic

FONT 2216. FORMA FARMACÈUTICA I ÚS. Gargarisme (ús extern). DESTINACIÓ. Medicina humana.

Antisèptic extern

FONT 2216. FORMA FARMACÈUTICA I ÚS. Decocció (ús extern).

Per a la conjuntivitis

FONT 2216. DESCRIPCIÓ DE L'ÚS FETA PER L'INFORMANT. Bullida i en banys als ulls, per a la conjuntivitis. FORMA FARMACÈUTICA I ÚS. Bany (ús extern). DESTINACIÓ. Medicina humana.

Per a prevenir el càncer

FONT 2216. DESCRIPCIÓ DE L'ÚS FETA PER L'INFORMANT. L’aigua de farigola ben freda és bona, en fregues, per a prevenir el càncer de mama. FORMA FARMACÈUTICA I ÚS. Loció (ús extern). DESTINACIÓ. Medicina humana.

Per a trastorns del sistema respiratori

FONT 3932. FORMA FARMACÈUTICA I ÚS. Desconegut per l'informant / No consta. DESTINACIÓ. Medicina humana.

**Tija amb fulles/branques**

Antisèptic extern

FONTS 2214, 2217, 2229. DESCRIPCIÓ DE L'ÚS FETA PELS INFORMANTS. És un gran desinfectant (2217). És desinfectant de les ferides (2229). FORMA FARMACÈUTICA I ÚS. Bany (ús extern) (2214, 2217). Desconegut per l'informant / No consta (2229). PREPARACIÓ. L’aigua timolada es feia bullint la farigola durant una estona i deixant refredar (2214). L’aigua timolada es prepara bullint branques de farigola en aigua (2217). MODE D'UTILITZACIÓ/POSOLOGIA. Es feia aigua timolada i es posava sobre les ferides (2214). Es posa a sobre de les ferides (2217). DESTINACIÓ. Medicina humana.

Antitussigen

FONT 2229. DESCRIPCIÓ DE L'ÚS FETA PER L'INFORMANT. Se’n fa un remei per a la tos. FORMA FARMACÈUTICA I ÚS. Desconegut per l'informant / No consta. DESTINACIÓ. Medicina humana.

Estomacal

FONT 2204. DESCRIPCIÓ DE L'ÚS FETA PER L'INFORMANT. Per al mal de ventre. FORMA FARMACÈUTICA I ÚS. Infusió (ús intern).

Per al refredat

FONT 2224. FORMA FARMACÈUTICA I ÚS. Decocció (ús intern). DESTINACIÓ. Medicina humana.

Antisèptic ocular

FONT 2226. DESCRIPCIÓ DE L'ÚS FETA PER L'INFORMANT. Serveix per a fer banys oculars. FORMA FARMACÈUTICA I ÚS. Bany (ús extern). DESTINACIÓ. Medicina humana.

Digestiu

FONT 2226. DESCRIPCIÓ DE L'ÚS FETA PER L'INFORMANT. És digestiva. En infusió. FORMA FARMACÈUTICA I ÚS. Infusió (ús intern). DESTINACIÓ. Medicina humana.

Tranquil·litzant

FONT 2226. DESCRIPCIÓ DE L'ÚS FETA PER L'INFORMANT. Va bé per a calmar els nervis. En infusió. FORMA FARMACÈUTICA I ÚS. Infusió (ús intern). DESTINACIÓ. Medicina humana.

**USOS ALIMENTARIS**

**Flor**

Condiment

FONT 2221. DESTINACIÓ. Alimentació humana.

**Fulla**

Condiment

FONTS 2183, 2206, 2215. DESTINACIÓ. Alimentació humana.

Ingestió de la part de la planta cuita - Cuita en aigua

FONT 2225. DESCRIPCIÓ DE L'ÚS FETA PER L'INFORMANT. Se'n fan sopes. OBSERVACIONS. Sopa. DESTINACIÓ. Alimentació humana.

**Part aèria**

Ingestió de la part de la planta cuita - Cuita en aigua

FONTS 2181, 2183, 2184, 2218, 2221, 3934. DESCRIPCIÓ DE L'ÚS FETA PELS INFORMANTS. En sopa (2218). Sopa (3934). Se'n fan sopes (2221). DESTINACIÓ. Alimentació humana.

**Tija amb fulles/branques**

Condiment

FONTS 2184, 2187, 2195, 2210, 2221, 3946. DESCRIPCIÓ DE L'ÚS FETA PELS INFORMANTS. Per a l'adobat d'olives (2184). Com a condiment de carns rostides (2187). Per als llagostins bullits (2221). DESTINACIÓ. Alimentació humana (2187, 2195, 2210, 2221, 3946).

Ingestió de la part de la planta cuita - Cuita en aigua

FONTS 2187, 2188, 2189, 2191, 2192, 2226, 2228. DESCRIPCIÓ DE L'ÚS FETA PELS INFORMANTS. Sopa. En feien sopes escaldades amb farigola, pa sec i un ou (2187). Sopa (2188, 2191, 2192). Sopa . Amb pa sec i ou (2189). En sopes (2226). Per al caldo (2228). OBSERVACIONS. Es fa bullir l’aigua, s'hi posa la farigola cinc minuts, es cola i després s’hi posa una mica de pa. A vegades s’hi posa també un ou cru (2192). Sopa (2226). DESTINACIÓ. Alimentació humana (2187, 2188, 2189, 2191, 2192, 2226, 2228).

**BARREGES AMB AQUEST TÀXON (vegeu catàleg de barreges)**

**USOS ALIMENTARIS**

**Tija amb fulles/branques**

FONT 2192. Anís de cireres.

FONT 2228. Caldo depuratiu.

**USOS MEDICINALS**

**Tija amb fulles/branques**

FONT 2209, 2211. Esperit de vi d'herbes.

FONT 2229. Remei per a la tos.

**Summitat florífera**

FONT 2184. Infusió diürètica.

**Part aèria florida**

FONT 2190. Per al refredat.

FONT 2162. Per al cos.

***Tilia platyphyllos***Scop. (malvàcies)
BCN 156595

**NOMS POPULARS**

Til·la (2217, 2231, 2229, 2228)

**USOS MEDICINALS**

**Bràctea i inflorescència**

Tranquil·litzant

FONTS 2217, 2228, 2229, 2231. DESCRIPCIÓ DE L'ÚS FETA PELS INFORMANTS. Per als nervis, en infusió (2229). Calmant (2228). Per a calmar els nervis (2231). FORMA FARMACÈUTICA I ÚS. Infusió (ús intern) (2217, 2229, 2231). Decocció (ús intern) (2228). DESTINACIÓ. Medicina humana.

***Tribulus terrestris***L. (zigofil·làcies)
BCN 130938

**NOMS POPULARS**

Queixal de vella (2162, 2204, 2208, 2210, 3946)

Rebentabicicletes (2216)

**USOS MEDICINALS**

**No consta**

Hepatoprotector

FONTS 2204, 2208. FORMA FARMACÈUTICA I ÚS. Desconegut per l'informant / No consta (2204). Infusió (ús intern) (2208). DESTINACIÓ. Medicina humana.

Per a infeccions a la boca

FONT 2162. FORMA FARMACÈUTICA I ÚS. Desconegut per l'informant / No consta. DESTINACIÓ. Medicina humana.

Protector renal

FONTS 2204, 2208. FORMA FARMACÈUTICA I ÚS. Desconegut per l'informant / No consta (2204). Infusió (ús intern) (2208). DESTINACIÓ. Medicina humana.

**ALTRES OBSERVACIONS**

FONT 3946. DESCRIPCIÓ FETA PER L'INFORMANT. Punxa les rodes de les bicicletes.

***Trifolium alexandrinum***L. (papilionàcies)
BCN 14003

**NOMS POPULARS**

Alfals d'hivern (3700)

Alfals francès (3700)

Bensim (2216)

Bersim (3700)

**USOS ALIMENTARIS**

**Part aèria**

No consta el tipus d'ingestió - No consta el mode de preparació

FONTS 2216, 3700. DESCRIPCIÓ DE L'ÚS FETA PER L'INFORMANT. Herba de farratge per als animals. Es feia a l’estiu (2216). Sobretot es donava de menjar a cavalls i vaques (3700). DESTINACIÓ. Alimentació animal (2216, 3700).

**ALTRES USOS**

**Part aèria**

Agrosilvopastoral

FONT 2216. DESCRIPCIÓ DE L'ÚS FETA PER L'INFORMANT. La part aèria seca [dit "palla"] del bensim se l’emportaven al Born per a facilitar la distribució dels melons. Feien com uns canals per on rodaven des del camió fins als coixins de bensim. Així, els organitzaven per mides. OBSERVACIONS. Elaboració d'estris d'ús hortícola/agrícola.

***Triticum aestivum***L. (gramínies)
BCN 156578

**NOMS POPULARS**

Blat (2182, 2185, 2186, 2192, 2204, 2210, 2211, 2212, 2217, 3939, 3947, 2229)

Blat Aragó 3 (raça) (2204)

Blat Clàdor (raça) (2204)

Blat espelta (raça) (2204)

Blat foraster (raça) (2204)

Blat Fortunato (raça) (2204)

Blat Impeto (raça) (2204)

Blat Impetuoso (raça) (2204)

Blat Littorio (raça) (2204)

Blat Mentana (raça) (2204)

Blat Mistral (raça) (2204)

Blat Montjuïc (raça) (2204)

Blat paner (raça) (2204)

Blat Sasserres (raça) (2204)

Blat Velino (raça) (2204)

Farina (producte elaborat) (2212, 2215, 3947)

Pa (producte elaborat) (2187, 2189, 2207, 3947)

Segó (producte elaborat) (2210, 2211)

Segonet (producte elaborat) (2210)

**USOS MEDICINALS**

**Segó**

Antiinflamatori faringi

FONTS 2204, 2210, 2211. FORMA FARMACÈUTICA I ÚS. Sinapisme (ús extern) (2204). Cataplasma (ús extern) (2210, 2211). MODE D'UTILITZACIÓ/POSOLOGIA. Per a guarir el mal de coll, s’havia de fer un cataplasma ben calent de segó de blat, ben pastat i aplicar-lo al coll, a l’hora d’anar a dormir (2204). Coixí calent de segó a dins i posat al coll, per al mal de gola (2210). Amb el segó del blat s’omplien uns coixins que, calents i posats al coll, servien per a guarir el mal de gola (2211). DESTINACIÓ. Medicina humana.

**USOS ALIMENTARIS**

**Segó**

No consta el tipus d'ingestió - No consta el mode de preparació

FONT 2210. DESCRIPCIÓ DE L'ÚS FETA PER L'INFORMANT. La clofolla de blat se’n diu segó i és un bon aliment per als animals. El segonet és el segó més fi i també se’n donava als animals. DESTINACIÓ. Alimentació animal.

**Tija**

Ingestió de la part de la planta crua - Conservada dessecada a l'aire

FONT 2185, 2229. DESCRIPCIÓ DE L'ÚS FETA PER L'INFORMANT. Per als cavalls (2185). La palla es donava de menjar a cavalls (2229). DESTINACIÓ. Alimentació animal (2185, 2229).

**Trituració del gra**

No consta el tipus d'ingestió - No consta el mode de preparació

FONTS 2187, 2189, 2207, 2212, 2215, 2217, 3939, 3947. DESCRIPCIÓ DE L'ÚS FETA PELS INFORMANTS. Per a fer sopes (2187, 2189). Per a arrebossar (2215). En donaven als porcs (2217). Per a fer pa (3939). Se'n menja (3947). DESTINACIÓ. Alimentació humana (2187, 2189, 2207, 2212, 2215, 3939, 3947). Alimentació animal (2217).

**ALTRES OBSERVACIONS**

FONT 2212. DESCRIPCIÓ FETA PER L'INFORMANT. Se sembrava a la tardor a les Sorres i als camps d’argila de sobre el camí Ral, en terra convenientment llaurada i adobada. La sembra es feia escampant la llavor a eixam o llençant la llavor dins els solcs. Abans de la sembra, la llavor es sotmetia a un bany de sulfat de coure o s’encalcinava per a evitar el carbó. A la primavera, s’escampava sobre el camp nitrat de Xile i, al juny, es començava la sega. La sega es feia amb el volant, una eina semblant a la falç, típica del Baix Llobregat, que aconseguia fer més palla. Conreu ja desaparegut.

**BARREGES AMB AQUEST TÀXON (vegeu catàleg de barreges)**

**USOS ALIMENTARIS**

**Trituració del gra**

FONT 2212. Peus de porc amb naps.

FONT 3947. Pa de Sant Nicasi.

**USOS MEDICINALS**

**Tija**

FONT 2182. Palla.

**Trituració del gra**

FONT 2192. Cataplasma per al mal d'esquena.

***Tropaeolum majus***L. (tropeolàcies)
BCN 81721

**NOMS POPULARS**

Caputxina (2165)

**USOS ALIMENTARIS**

**Flor i fulla**

Ingestió de la part de la planta crua - Fresca (sense preparació)

FONT 2165. CONSUMICIÓ. Amanida. DESTINACIÓ. Alimentació humana.

**Fulla**

Ingestió de la part de la planta cuita - Cuita en aigua

FONT 2165. DESCRIPCIÓ DE L'ÚS FETA PER L'INFORMANT. Per a fer farcellets, que després es farceixen de carn o verdures. DESTINACIÓ. Alimentació humana.

***Typha latifolia***L. (tifàcies)
BCN 31314

**NOMS POPULARS**

Boga (2209, 2210, 2211)

Coet (inflorescència) (2209, 2211)

**ALTRES USOS**

**Fulla**

Elaboració de mobles

FONT 2210. DESCRIPCIÓ DE L'ÚS FETA PER L'INFORMANT. Per a fer els culs de les cadires.

**Tija florífera**

Elaboració de rams

FONTS 2209, 2210, 2211. DESCRIPCIÓ DE L'ÚS FETA PER L'INFORMANT. Ornamental. Es pintava o s'assecava (2211).

***Ulex parviflorus***Pourr. (papilionàcies)
BCN 129011

**NOMS POPULARS**

Gatosa (2187, 2188, 2189, 2192, 2213)

**ALTRES USOS**

**Tija amb fulles/branques**

Ajuda a la llar

FONTS 2187, 2188, 2189. DESCRIPCIÓ DE L'ÚS FETA PELS INFORMANTS. Per a netejar la xemeneia (2187). Per a netejar el sutge de la xemeneia. Se’n feia un lligall i, amb una corda, un des de dalt i un altre des de sota, anaven rascant el tub de la xemeneia (2188). Per a netejar el sutge de les xemeneies. Es feia un lligall i un des de dalt i l’altre des d’avall estiraven i treien la brutícia (2189).

Ajuda en la matança del porc

FONT 2213. DESCRIPCIÓ DE L'ÚS FETA PER L'INFORMANT. S’encenia i s’utilitzava per a cremar i treure el pèl dels porcs.

Obtenció de combustible: llenya

FONTS 2188, 2189, 2192, 2213. DESCRIPCIÓ DE L'ÚS FETA PELS INFORMANTS. Per Sant Joan es collien per a encendre els focs (2189). En feien feixines o gavelles que servien per a cremar als forns (2192). Per a fer foc (2213).

***Umbilicus rupestris***(Salisb.) Dandy (crassulàcies)
BCN 129707

**NOMS POPULARS**

Barralets (2214)

***Urtica dioica***L. (urticàcies)
BCN 29814

**NOMS POPULARS**

Ortiga (2179, 3933, 3939, 3952, 2229, 2228)

**USOS MEDICINALS**

**Fulla**

Diürètic

FONTS 3933, 3952. FORMA FARMACÈUTICA I ÚS. Infusió (ús intern). DESTINACIÓ. Medicina humana.

Hematocatàrtic

FONT 2228. DESCRIPCIÓ DE L'ÚS FETA PER L'INFORMANT. Remineralitzant de la sang. FORMA FARMACÈUTICA I ÚS. Desconegut per l'informant / No consta. DESTINACIÓ. Medicina humana.

Hemostàtic extern

FONT 3952. DESCRIPCIÓ DE L'ÚS FETA PER L'INFORMANT. Per a detenir les hemorràgies, especialment les nasals, taponant amb una gasa xopa del suc. FORMA FARMACÈUTICA I ÚS. Bany (ús extern). DESTINACIÓ. Medicina humana.

No consta

FONT 3939. DESCRIPCIÓ DE L'ÚS FETA PER L'INFORMANT. Se’n fa aigua d’ortigues, que és bona com a medicament (desconeix per a què). FORMA FARMACÈUTICA I ÚS. Decocció (ús intern). DESTINACIÓ. Medicina humana.

**Part aèria**

Antihipertensor

FONT 2229. DESCRIPCIÓ DE L'ÚS FETA PER L'INFORMANT. Per a la sang. FORMA FARMACÈUTICA I ÚS. Desconegut per l'informant / No consta. DESTINACIÓ. Medicina humana.

Antireumàtic

FONT 3952. DESCRIPCIÓ DE L'ÚS FETA PER L'INFORMANT. Es fan urticacions: s’agafa un ram d'ortigues i es pica suaument sobre l'articulació inflamada o en què es pateix reuma. Es produeix un efecte revulsiu que atreu la sang cap a la pell, descongestionant a la vegada els teixits interns. FORMA FARMACÈUTICA I ÚS. Sense forma farmacèutica (ús directe) (ús extern). DESTINACIÓ. Medicina humana.

Salutífer

FONT 2229. DESCRIPCIÓ DE L'ÚS FETA PER L'INFORMANT. En infusió, dona energia. FORMA FARMACÈUTICA I ÚS. Infusió (ús intern).

**Suc de la fulla**

Diürètic

FONT 3952. FORMA FARMACÈUTICA I ÚS. Sense forma farmacèutica (ús directe) (ús intern). DESTINACIÓ. Medicina humana.

**USOS ALIMENTARIS**

**Fulla**

Ingestió de la part de la planta crua - Fresca (sense preparació)

FONT 3952. DESCRIPCIÓ DE L'ÚS FETA PER L'INFORMANT. És un bon aliment si es consumeix crua en amanida (a les 12 hores d'haver-la arrancat ja no pica). CONSUMICIÓ. Amanida. DESTINACIÓ. Alimentació humana.

Ingestió de la part de la planta cuita - Cuita en aigua

FONTS 2228, 3952. DESCRIPCIÓ DE L'ÚS FETA PELS INFORMANTS. Bullida (3952). Escaldada, en amanida (2228). OBSERVACIONS. Sopa (3952). CONSUMICIÓ. Amanida (2228). DESTINACIÓ. Alimentació humana (2228, 3952).

Ingestió de la part de la planta cuita - Cuita en oli

FONT 3952. CONSUMICIÓ. Plat principal - Truita. DESTINACIÓ. Alimentació humana.

**BARREGES AMB AQUEST TÀXON (vegeu catàleg de barreges)**

**USOS MEDICINALS**

**Fulla**

FONT 2228. Oli de romaní, ortiga i pericó.

***Urtica urens***L. (urticàcies)
BCN 126571

**NOMS POPULARS**

Ortiga (2161, 2162, 2178, 2180, 2182, 2191, 2209, 2210, 2219, 2224, 3934)

**USOS MEDICINALS**

**Fulla**

Antialopècic

FONT 2162. FORMA FARMACÈUTICA I ÚS. Infusió (ús extern). DESTINACIÓ. Medicina humana.

Desconegut per l'informant

FONT 2180. FORMA FARMACÈUTICA I ÚS. Infusió (ús intern). DESTINACIÓ. Medicina humana.

**No consta**

No consta

FONT 3934. DESCRIPCIÓ DE L'ÚS FETA PER L'INFORMANT. Té propietats medicinals, però en desconeix l’ús. FORMA FARMACÈUTICA I ÚS. Desconegut per l'informant / No consta. DESTINACIÓ. Medicina humana.

**Part aèria**

Antihipertensor

FONT 2191. FORMA FARMACÈUTICA I ÚS. Infusió (ús intern). DESTINACIÓ. Medicina humana.

Diürètic

FONT 2178. FORMA FARMACÈUTICA I ÚS. Sense forma farmacèutica (ús directe) (ús intern). MODE D'UTILITZACIÓ/POSOLOGIA. En sopa. DESTINACIÓ. Medicina humana.

Protector renal

FONT 2178. FORMA FARMACÈUTICA I ÚS. Sense forma farmacèutica (ús directe) (ús intern). MODE D'UTILITZACIÓ/POSOLOGIA. En sopa. DESTINACIÓ. Medicina humana.

**USOS ALIMENTARIS**

**Fulla**

Ingestió de la part de la planta cuita - Cuita en aigua

FONT 2210. DESCRIPCIÓ DE L'ÚS FETA PER L'INFORMANT. Sopa. DESTINACIÓ. Alimentació humana.

Ingestió de la part de la planta cuita - Cuita en oli

FONT 2224. DESCRIPCIÓ DE L'ÚS FETA PER L'INFORMANT. Comestible. Se’n poden fer truites. CONSUMICIÓ. Plat principal - Truita. DESTINACIÓ. Alimentació humana.

**ALTRES USOS**

**Part aèria**

Agrosilvopastoral

FONT 2182. DESCRIPCIÓ DE L'ÚS FETA PER L'INFORMANT. Les picaven i en feien preparats contra totes les malures del camp. OBSERVACIONS. Plaguicides naturals.

***Vaccinium myrtillus***L. (ericàcies)
BCN-E-231

**NOMS POPULARS**

Nabiu (fruit) (2205)

**USOS ALIMENTARIS**

**Fruit**

No consta el tipus d'ingestió - No consta el mode de preparació

FONT 2205. DESTINACIÓ. Alimentació humana.

***Valeriana officinalis***L. (caprifoliàcies)
BCN 29816

**NOMS POPULARS**

Valeriana (2182)

**USOS MEDICINALS**

**Arrel**

Sedant

FONT 2182. FORMA FARMACÈUTICA I ÚS. Infusió (ús intern). DESTINACIÓ. Medicina humana.

***Valerianella locusta***(L.) Laterrade (caprifoliàcies)
BCN 129693

**NOMS POPULARS**

Canonge (2200, 2203)

**USOS ALIMENTARIS**

**Part aèria**

Ingestió de la part de la planta crua - Fresca (sense preparació)

FONTS 2200, 2203. DESTINACIÓ. Alimentació humana.

***Verbascum sinuatum***L. (escrofulariàcies)
BCN 130948

**ALTRES USOS**

**Fulla**

Ajuda a la llar

FONT 2214. DESCRIPCIÓ DE L'ÚS FETA PER L'INFORMANT. Per a netejar-se el cul.

Cosmètic

FONT 2214. DESCRIPCIÓ DE L'ÚS FETA PER L'INFORMANT. Elaboració de sabons.

***Verbena officinalis***L. (verbenàcies)
BCN 132992

**USOS MEDICINALS**

**No consta**

Mucolític

FONT 2174. DESCRIPCIÓ DE L'ÚS FETA PER L'INFORMANT. Per a alleujar la mucositat al pit. FORMA FARMACÈUTICA I ÚS. Desconegut per l'informant / No consta. DESTINACIÓ. Medicina humana.

***Veronica persica***Poiret in Lam. (plantaginàcies)
BCN 25038

**NOMS POPULARS**

Verònica (2214)

***Vicia faba***L. (papilionàcies)
BCN 129001

**NOMS POPULARS**

Fava (2178, 2184, 2186, 2191, 2193, 2194, 2195, 2198, 2199, 2205, 2206, 3700, 2221, 2212, 2217, 2224, 2225, 2218, 2227)

Fava tendra (2203)

**USOS ALIMENTARIS**

**Llavor**

Ingestió de la part de la planta crua - Conservada dessecada a l'aire

FONT 3700. DESCRIPCIÓ DE L'ÚS FETA PELS INFORMANTS. De vegades es deixaven assecar per a donar-les a les vaques. Alimentació animal.

Ingestió de la part de la planta cuita - Cuita en aigua

FONTS 2217, 2224, 3700. DESCRIPCIÓ DE L'ÚS FETA PELS INFORMANTS. Cuites (2224). DESTINACIÓ. Alimentació humana (2217, 2224, 3700).

Ingestió de la part de la planta cuita - Cuita en aigua i oli

FONT 2218. DESCRIPCIÓ DE L'ÚS FETA PER L'INFORMANT. Se’n mengen estofades. DESTINACIÓ. Alimentació humana.

No consta el tipus d'ingestió - No consta el mode de preparació

FONTS 2178, 2193, 2194, 2195, 2198, 2199, 2203, 2205, 2206, 2221, 2227. DESCRIPCIÓ DE L'ÚS FETA PELS INFORMANTS. Se'n menja (2227). DESTINACIÓ. Alimentació humana (2178, 2193, 2194, 2195, 2198, 2199, 2203, 2205, 2206, 2221, 2227).

**Part aèria jove**

No consta el tipus d'ingestió - No consta el mode de preparació

FONT 2218. DESCRIPCIÓ DE L'ÚS FETA PER L'INFORMANT. Les puntes de les faveres, que eren tendres quan les arrencàvem, les donàvem als conills i a les cabres. DESTINACIÓ. Alimentació animal.

**Trituració del gra**

No consta el tipus d'ingestió - No consta el mode de preparació

FONTS 2184, 3700. DESCRIPCIÓ DE L'ÚS FETA PELS INFORMANTS. Se'n feia farina (3700). En feien farinassa per a alimentar els porcs durant els últims tres mesos abans de la matança del porc (2184). DESTINACIÓ. Alimentació humana (3700). Alimentació animal (2184).

**ALTRES USOS**

**Llavor**

Elaboració d'obres artístiques

FONT 2225. DESCRIPCIÓ DE L'ÚS FETA PER L'INFORMANT. Per a elaborar les paneres artístiques.

**Planta viva *in situ***

Agrosilvopastoral

FONT 2191. DESCRIPCIÓ DE L'ÚS FETA PER L'INFORMANT. L'avi se’n posava un grapat a la butxaca i les anava sembrant als marges, perquè atreien el pugó. OBSERVACIONS. Associació de cultius i equilibri sistèmic.

***Vicia sativa***L. (papilionàcies)
BCN 130961

**NOMS POPULARS**

Trepadella (2210)

Veça (2160, 2216)

**USOS ALIMENTARIS**

**Llavor**

No consta el tipus d'ingestió - No consta el mode de preparació

FONT 2216. DESCRIPCIÓ DE L'ÚS FETA PER L'INFORMANT. Els coloms se’n mengen les llavors. DESTINACIÓ. Alimentació animal.

**ALTRES USOS**

**Llavor**

Jocs i joguines

FONTS 2160, 2216. DESCRIPCIÓ DE L'ÚS FETA PELS INFORMANTS. Les llavors es posaven en un canó [dit "*canuto*"] i es llençaven amb la boca, per a jugar (2160, 2216).

**Planta sencera**

Agrosilvopastoral

FONT 2216. DESCRIPCIÓ DE L'ÚS FETA PER L'INFORMANT. Com a adob verd, per als camps. Es sega, s’asseca i es tira sobre els camps. OBSERVACIONS. Adobs i fertilitzants naturals.

***Vinca major***L. (apocinàcies)
BCN 129695

**NOMS POPULARS**

Herba donzella (2214)

***Viola sylvestris***Lam. (violàcies)
BCN 129013

**NOMS POPULARS**

Violeta (2229)

***Viola tricolor***L. var.***hortensis***DC.
BCN 132997

**NOMS POPULARS**

Pensament (2165, 2193, 2225)

**USOS MEDICINALS**

**No consta**

Desconegut per l'informant

FONT 2165. FORMA FARMACÈUTICA I ÚS. Desconegut per l'informant / No consta. DESTINACIÓ. Medicina humana.

**USOS ALIMENTARIS**

**Flor**

Condiment

FONT 2225. DESTINACIÓ. Alimentació humana.

Ingestió de la part de la planta crua - Fresca (sense preparació)

FONTS 2165, 2193. CONSUMICIÓ. Amanida (2165). DESTINACIÓ. Alimentació humana (2165, 2193).

***Vitis vinifera***L. (vitàcies)
BCN 150353

**NOMS POPULARS**

Carràs de raïm (infructescència) (2212)

Cep (2188)

Gra de raïm (fruit) (2192)

Gotim (part de la infructescència) (2192)

Parra (2166, 2173, 2190)

Raïm (fruit) (2190, 2192)

Raïm (infructescència) (2229)

Raïm negre (fruit) (2200, 2203)

Raïm rosat (fruit) (2203)

Rastella de raïm (infructescència) (2192)

Vi (producte elaborat) (2184)

Vi dolç (producte elaborat) (3700)

Vinagre (producte elaborat) (2204)

Vinya (2168, 2169, 2185, 2204, 2212)

**USOS ALIMENTARIS**

**Fruit**

Ingestió de la part de la planta crua - Conservada dessecada a l'aire

FONT 2192. DESCRIPCIÓ DE L'ÚS FETA PER L'INFORMANT. Per a consumir-ne sec. Es posava una rastella de raïm de gra gros penjada d’un cordill del sostre o d’una canya en un lloc on passés l’aire. Aguantava durant tres o quatre mesos. Quedava pansit, però seguia sent raïm. DESTINACIÓ. Alimentació humana.

Ingestió de la part de la planta crua - Fresca (sense preparació)

FONTS 2168, 2169, 2190, 2200, 2203. DESCRIPCIÓ DE L'ÚS FETA PELS INFORMANTS. Se'n mengen els seus grans crus (2168). DESTINACIÓ. Alimentació humana (2168, 2169, 2190, 2200, 2203).

Preparació de begudes - Beguda preparada per fermentació

FONT 2192. DESCRIPCIÓ DE L'ÚS FETA PER L'INFORMANT. S'elaborava vi per al consum propi. DESTINACIÓ. Alimentació humana.

**Fulla**

Ingestió de la part de la planta cuita - Cuita en aigua

FONT 2166. DESCRIPCIÓ DE L'ÚS FETA PER L'INFORMANT. Per a fer farcellets. DESTINACIÓ. Alimentació humana.

No consta el tipus d'ingestió - No consta el mode de preparació

FONT 2173. DESCRIPCIÓ DE L'ÚS FETA PER L'INFORMANT. La fulla es pot cuinar. DESTINACIÓ. Alimentació humana.

**Infructescència**

Ingestió de la part de la planta crua - Fresca (sense preparació)

FONT 2212. DESCRIPCIÓ DE L'ÚS FETA PER L'INFORMANT. Es conservaven fins Nadal si es penjaven al lloc més sec de la casa. DESTINACIÓ. Alimentació humana.

Preparació de begudes - Beguda preparada per fermentació

FONT 2229. DESCRIPCIÓ DE L'ÚS FETA PER L'INFORMANT. Es feia mistela amb el primer raïm que es trepitjava. DESTINACIÓ. Alimentació humana.

**ALTRES USOS**

**Oli de la llavor**

Cosmètic

FONT 2166.

**ALTRES OBSERVACIONS**

FONTS 2185, 2192. DESCRIPCIÓ FETA PELS INFORMANTS. Fa cent anys se n'havia fet al Delta del Llobregat (2185). El gotim és el raïm que queda a la planta després de la verema (2192).

**BARREGES AMB AQUEST TÀXON (vegeu catàleg de barreges)**

**USOS ALIMENTARIS**

**Fruit**

FONT 2192. Arrop.

**USOS MEDICINALS**

**Suc del fruit**

FONT 2184. Per al refredat.

FONT 2204. Per al mal de queixal.

FONT 2217. Per a treure els blaus.

***Xanthosoma sagittifolium***(L.) Schott (aràcies)
BCN-E-215

**NOMS POPULARS**

Malanga (2200)

**USOS ALIMENTARIS**

**Tubercle**

No consta el tipus d'ingestió - No consta el mode de preparació

FONT 2200. DESTINACIÓ. Alimentació humana.

***Zea mays***L. (gramínies)
BCN-E-196

**NOMS POPULARS**

Blat de moro (2160, 2179, 2182, 2184, 2185, 2186, 2187, 2191, 2192, 2209, 2210, 3700, 2211, 2212, 2223, 2218)

Cabellera (estil i estigmes) (2209, 2211)

Cor de la panotxa (eix de la infructescència) (2229)

Espigot (eix de la infructescència) (2179)

Moresc (2212)

Notari (eix de la infructescència) (2160, 2185)

Panotxa (infructescència) (2183, 2187, 2192, 2204, 2209, 2210, 2217, 2229, 2223)

Pèl de la panotxa (estils i estigmes) (2217)

**USOS MEDICINALS**

**Estil i/o estigma**

Antiprostatític

FONT 2217. DESCRIPCIÓ DE L'ÚS FETA PER L'INFORMANT. Els estils i estigmes [dit “pèl de la panotxa”], en infusió, eren bons per als que tenien problemes de pròstata. FORMA FARMACÈUTICA I ÚS. Infusió (ús intern). DESTINACIÓ. Medicina humana.

Diürètic

FONTS 2187, 2191, 2192, 2204, 2210, 2211, 2217. DESCRIPCIÓ DE L'ÚS FETA PELS INFORMANTS. La cabellera, en infusió, per a orinar (2211). La pelussa de les panotxes, en infusió, serveix per a fer orinar (2191). El pèl de la panotxa, en infusió, eren bons per a fer pipí (2217). FORMA FARMACÈUTICA I ÚS. Infusió (ús intern). MODE D'UTILITZACIÓ/POSOLOGIA. La pelussa de les panotxes va bé per orinar (2192). La cabellera de panotxa era molt bona per a fer orinar. Calia bullir la cabellera de panotxa i beure’n un got al dia, en dejú, durant nou dies seguits (2204). La cabellera de la panotxa, per a orinar (2210). DESTINACIÓ. Medicina humana.

Litotríptic renal

FONT 2183. DESCRIPCIÓ DE L'ÚS FETA PER L'INFORMANT. El pèl de panotxa va bé per al ronyó. FORMA FARMACÈUTICA I ÚS. Infusió (ús intern). DESTINACIÓ. Medicina humana.

Protector renal

FONT 2187. FORMA FARMACÈUTICA I ÚS. Infusió (ús intern). DESTINACIÓ. Medicina humana.

**Infructescència**

Antitussigen

FONT 2229. DESCRIPCIÓ DE L'ÚS FETA PER L'INFORMANT. Se’n fa un remei per a la tos. FORMA FARMACÈUTICA I ÚS. Desconegut per l'informant / No consta. DESTINACIÓ. Medicina humana.

Diürètic

FONT 2209. FORMA FARMACÈUTICA I ÚS. Infusió (ús intern). MODE D'UTILITZACIÓ/POSOLOGIA. La cabellera de la panotxa va bé per a orinar. DESTINACIÓ. Medicina humana.

**USOS ALIMENTARIS**

**Bràctea**

No consta el tipus d'ingestió - No consta el mode de preparació

FONTS 2185, 2186. DESCRIPCIÓ DE L'ÚS FETA PELS INFORMANTS. Per a les vaques (2185). Les bràctees [dit "la pellofa"] es donaven de menjar a les vaques (2186). DESTINACIÓ. Alimentació animal (2185, 2186).

**Llavor**

No consta el tipus d'ingestió - No consta el mode de preparació

FONTS 2182, 2210. DESCRIPCIÓ DE L'ÚS FETA PELS INFORMANTS. Per a l'aviram (2182). DESTINACIÓ. Alimentació animal (2182, 2210).

**No consta**

No consta el tipus d'ingestió - No consta el mode de preparació

FONT 2184. DESCRIPCIÓ DE L'ÚS FETA PER L'INFORMANT. Per als porcs. DESTINACIÓ. Alimentació animal.

**Trituració del gra**

No consta el tipus d'ingestió - No consta el mode de preparació

FONT 2218. DESCRIPCIÓ DE L'ÚS FETA PER L'INFORMANT. Feien una espècie de pa, molent el gra, fent una pasta i ficant-lo a la paella, com si fossin unes coquetes. DESTINACIÓ. Alimentació humana. DESCRIPCIÓ DE L'ÚS FETA PER L'INFORMANT. Per als animals. DESTINACIÓ. Alimentació animal.

**ALTRES USOS**

**Bràctea**

Elaboració de fibres

FONT 3700. DESCRIPCIÓ DE L'ÚS FETA PER L'INFORMANT. Les bràctees [dit “pellofa”] trenades servien per a decorar les botelles de rom. OBSERVACIONS. Ornamental.

Elaboració de joguines

FONT 2186. DESCRIPCIÓ DE L'ÚS FETA PER L'INFORMANT. Les bràctees [dit “pell de la panotxa”] les comprava una senyora de Barcelona per a fer nines artesanes.

Elaboració de matalassos

FONT 3700. DESCRIPCIÓ DE L'ÚS FETA PER L'INFORMANT. Les bràctees [dit “pellofa”] servien per a omplir matalassos i coixins.

Elaboració d'obres artístiques

FONT 2223. DESCRIPCIÓ DE L'ÚS FETA PER L'INFORMANT. Les bràctees [dit “pell de la panotxa”] serveixen per a l’elaboració de les paneres artístiques.

**Infructescència**

Obtenció de combustible: llenya

FONTS 2179, 2185. DESCRIPCIÓ DE L'ÚS FETA PELS INFORMANTS. Els espigots del blat de moro també es guardaven i es cremaven a l'hivern (2179). Els notaris es cremaven a la llar de foc perquè feien una bona brasa (2185).

**Raquis**

Obtenció de combustible: llenya

FONTS 2160, 2218. DESCRIPCIÓ DE L'ÚS FETA PELS INFORMANTS. Els fan servir per a cremar i fer brasa (2160). Les panotxes, les penjaven al sol de tres en tres. El raquis [dit "pinya seca"], el feien servir per a encendre el foc (2218).

**Tija**

Agrosilvopastoral

FONT 2210. DESCRIPCIÓ DE L'ÚS FETA PER L'INFORMANT. Les cames [tiges] servien com a adob orgànic per a les síndries. OBSERVACIONS. Adobs i fertilitzants naturals.

**ALTRES OBSERVACIONS**

FONT 3700. DESCRIPCIÓ FETA PER L'INFORMANT. Es retirava la pellofa de la panotxa [acció denominada "espanotxar"] i s’anaven lligant fent manats de sis o vuit panotxes. Es posaven al voltant d’un pal perquè s’assequessin.

**BARREGES AMB AQUEST TÀXON (vegeu catàleg de barreges)**

**USOS MEDICINALS**

**Infructescència**

FONT 2229. Remei per a la tos.

***Zingiber officinale***Roscoe (zingiberàcies)
BCN-E-213

**NOMS POPULARS**

Gingebre (2206)

*Jengibre* (castellà) (2200)

**USOS ALIMENTARIS**

**Rizoma**

Condiment

FONTS 2200, 2206. DESCRIPCIÓ DE L'ÚS FETA PELS INFORMANTS. Per a plats exòtics (2206). DESTINACIÓ. Alimentació humana (2200, 2206).

**BARREGES MEDICINALS**

Analgèsic

**Esperit de vi d'herbes**

FONTS 2209, 2211. Gavà (Baix Llobregat)

INGREDIENTS:

***Lavandula dentata***L. (labiades) - Tija amb fulles/branques

***Pinus halepensis***Mill. (pinàcies) - Gemma de la fulla

***Rosmarinus officinalis***L. (labiades) - Tija amb fulles/branques

***Thymus vulgaris***L. (labiades) - Tija amb fulles/branques

FORMA FARMACÈUTICA I ÚS. Liniment (ús extern)
EFECTES SECUNDARIS. No consta.
DESTINACIÓ. Medicina humana.
MODE D'UTILITZACIÓ/POSOLOGIA. Se'n feien fregues (2211).

PREPARACIÓ. Es fica tot en alcohol de 70º. Serveix per a fer fregues, per a mitigar el dolor (2209). Es fica romaní, farigola, espígol i brots de pi en alcohol de 70º. Es deixa macerar i es cola. És útil per a fer fregues i alleugerir el dolor (2211).

Antiàlgic lumbar

**Cataplasma per al mal d'esquena**

FONT 2192. Sant Climent de Llobregat (Baix Llobregat)

INGREDIENTS:

***Olea europaea***L. subsp. ***europaea*** var. ***europaea***(oleàcies) - Suc del fruit

***Triticum aestivum***L. (gramínies) - Trituració del gra

FORMA FARMACÈUTICA I ÚS. Cataplasma (ús extern)

EFECTES SECUNDARIS. No consta.

DESTINACIÓ. Medicina humana.

MODE D'UTILITZACIÓ/POSOLOGIA. Cataplasma de farina de blat amb un raig d’oli, es posava sobre els ronyons tota la nit per a apaivagar el mal.

Antidismenorreic

**Infusió de comí, romaní i sàlvia**

FONT 2204. Gavà (Baix Llobregat)

INGREDIENTS:

***Cuminum cyminum***L. (umbel·líferes) - Fruit

***Rosmarinus officinalis***L. (labiades) - Fulla

***Salvia officinalis***L. subsp. ***officinalis***(labiades) - Fulla

FORMA FARMACÈUTICA I ÚS. Infusió (ús intern)
EFECTES SECUNDARIS. No consta.
DESTINACIÓ. Medicina humana.

Antiinflamatori / Antiàlgic / Antiequimòtic

**Esperit de vi amb espígol i romaní**

FONT 2204. Gavà (Baix Llobregat)

INGREDIENTS:

***Lavandula dentata***L. (labiades) - Tija amb fulles/branques

***Rosmarinus officinalis***L. (labiades) - Tija amb fulles/branques

FORMA FARMACÈUTICA I ÚS. Liniment (ús extern)
EFECTES SECUNDARIS. No consta.
DESTINACIÓ. Medicina humana.
MODE D'UTILITZACIÓ/POSOLOGIA. Per al dolor d’ossos i músculs. Prèviament, i durant algun temps, calia haver ficat dins d’una ampolla esperit de vi, amb espígol i romaní -això era una pràctica habitual en moltes cases-. Aleshores, es feien fregues a les articulacions, a les zones dels ossos i dels ronyons per a calmar el dolor.

**Oli de romaní, ortiga i pericó**

FONT 2228. El Prat de Llobregat (Baix Llobregat)

INGREDIENTS:

***Hypericum perforatum***L. (gutíferes) - Flor

***Rosmarinus officinalis***L. (labiades) - Summitat florífera

***Urtica dioica***L. (urticàcies) - Fulla

FORMA FARMACÈUTICA I ÚS. Liniment (ús extern).
EFECTES SECUNDARIS. No consta.
DESTINACIÓ. Medicina humana.
DESCRIPCIÓ DE L'ÚS FETA PER L'INFORMANT. És bo per al dolor. Se’n fan fregues.
PREPARACIÓ. Es fiquen les plantes en un pot amb oli d'oliva i es deixa 40 dies a la foscor tapat amb una gasa. Es cola i es guarda en una ampolla de color ambre.

**Per a treure els blaus**

FONT 2217. El Prat de Llobregat (Baix Llobregat)

INGREDIENTS:

***Medicago sativa***L. (papilionàcies) - Part aèria
***Vitis vinifera***L. (vitàcies) - Suc del fruit

FORMA FARMACÈUTICA I ÚS. Cataplasma (ús extern).
EFECTES SECUNDARIS. No consta.
DESTINACIÓ. Medicina humana.
DESCRIPCIÓ DE L'ÚS FETA PER L'INFORMANT. És útil per a treure els blaus.

PREPARACIÓ. Es pica l'ufals amb vinagre.

Antihemorroidal

**Cataplasma per a les hemorroides**

FONT 2213. Gavà (Baix Llobregat)

INGREDIENTS:

***Olea europaea***L. subsp. ***europaea*** var. ***europaea***(oleàcies) - Suc del fruit

***Ruta graveolens***L. (rutàcies) - Part aèria
FORMA FARMACÈUTICA I ÚS. Cataplasma (ús extern).

EFECTES SECUNDARIS. No consta.

DESTINACIÓ. Medicina humana.

PREPARACIÓ. Es fa un cataplasma amb l’oli d’oliva en el qual prèviament s’ha fregit lleugerament la ruda.

POSOLOGIA. Es posa a sobre de la part afectada durant una bona estona.

Antiodontàlgic

**Per al mal de queixal**

FONT 2204. Gavà (Baix Llobregat).

INGREDIENTS:

***Pinus pinea***L. (pinàcies) - Escorça
***Vitis vinifera***L. (vitàcies) - Suc del fruit

FORMA FARMACÈUTICA I ÚS. Gargarisme (ús extern).

EFECTES SECUNDARIS. No consta.

DESTINACIÓ. Medicina humana.

PREPARACIÓ. Per a alleugerir-ne el mal de queixal, calia bullir una mica de vinagre i una teia de pi dins d’una olleta.

POSOLOGIA. Un cop fred, s'havia de glopejar diverses vegades al dia.

Antitetànic

**Palla**

FONTS 2182, 2204. Gavà (Baix Llobregat).

INGREDIENTS:

***Olea europaea***L. subsp. ***europaea*** var. ***europaea***(oleàcies) - Suc del fruit

***Triticum aestivum***L. (gramínies) - Tija

FORMA FARMACÈUTICA I ÚS. Apòsit medicamentós (ús extern).
EFECTES SECUNDARIS. No consta.
DESTINACIÓ. Medicina humana i veterinària (2182). Medicina humana (2204).
DESCRIPCIÓ DE L'ÚS FETA PER L'INFORMANT. Es feia servir quan et punxaves, contra el tètanus (2204).

PREPARACIÓ. Les palles es feien servir per a "fer una palla". Quan el bestiar o els mossos es clavaven una punxa, agafaven una palla de blat -del paller-, la posaven perpendicularment sobre la ferida i hi tiraven oli d'oliva per dintre, s'encenia amb una espelma per la part superior i l'oli calent arribava fins a la zona adolorida, curant-la (2182). S’agafava una palla llarga, s’amarava d’oli d’oliva i es posava vertical, se li calava foc per la punta de baix i l’oli anava baixant per la palla. Quan arribava al foc, s’escalfava i, aleshores, regalimava a l’interior de la ferida, com una boleta de foc (2204).

Antitussigen

**Remei per a la tos**

FONT 2229. Viladecans (Baix Llobregat)

INGREDIENTS:

***Ceratonia siliqua***L. (papilionàcies) - Fruit

***Eucalyptus globulus***Labill. (mirtàcies) - Fulla

***Thymus vulgaris***L. (labiades) - Tija amb fulles/branques

***Zea mays***L. (gramínies) - Infructescència

FORMA FARMACÈUTICA I ÚS. Infusió (ús intern)
EFECTES SECUNDARIS. No consta.
DESTINACIÓ. Medicina humana.
PREPARACIÓ. Es bull tot en aigua i, opcionalment, s’hi afegeix un rajolí de conyac.

Diürètic

**Infusió diürètica**

FONT 2184. El Prat de Llobregat (Baix Llobregat)

INGREDIENTS:

***Rosmarinus officinalis***L. (labiades) - Summitat florífera

***Thymus vulgaris***L. (labiades) - Summitat florífera

FORMA FARMACÈUTICA I ÚS. Infusió (ús intern)
EFECTES SECUNDARIS. No consta.
DESTINACIÓ. Medicina humana.

Hematocatàrtic

**Caldo depuratiu**

FONT 2228. El Prat de Llobregat (Baix Llobregat)

INGREDIENTS:

***Allium cepa***L. (amaril·lidàcies) - Planta sencera

***Apium graveolens***L. var. ***dulce*** (Mill.) Pers. (umbel·líferes) - Fulla

***Cichorium endivia***L. subsp. ***endivia***(compostes) - Fulla

***Citrus limon***(L.) Burm. (rutàcies) - Fruit

***Cucurbita pepo***L. (cucurbitàcies) - Fruit

***Cynara scolymus***L. (compostes) - Bràctea i inflorescència

***Daucus carota***L. subsp. ***sativus***(Hoffm.) Arcang. (umbel·líferes) - Arrel

***Lactuca sativa***L. (compostes) - Fulla

***Spinacia oleracea***L. (amarantàcies) - Fulla

***Thymus vulgaris***L. (labiades) - Tija amb fulles/branques

FORMA FARMACÈUTICA I ÚS. El caldo es pren mitja hora abans dels àpats, amb mitja llimona espremuda a dins.

EFECTES SECUNDARIS. No consta.
DESTINACIÓ. Medicina humana.
DESCRIPCIÓ DE L'ÚS FETA PER L'INFORMANT. És depuratiu.
PREPARACIÓ. Tres cebes, tres pastanagues, carbassó (a l’estiu), carxofa (a l’hivern), àpit i farigola. Es posen en una cassola dos litres d’aigua i es porta a ebullició durant 30 minuts. Al cap de 15 minuts s’hi afegeixen fulles verdes: escarola, enciam, fulles de les cebes, espinacs. Es deixa reposar cinc minuts, es cola i ja tenim el caldo.

Per a trastorns de la pell o del teixit subcutani

**Oli de boixac i rosa**

FONT 2228. El Prat de Llobregat (Baix Llobregat)

INGREDIENTS:

***Calendula officinalis***L. (compostes) - Inflorescència

***Rosa canina***L. (rosàcies) - Flor

FORMA FARMACÈUTICA I ÚS. Liniment (ús extern)
EFECTES SECUNDARIS. No consta.
DESTINACIÓ. Medicina humana.
DESCRIPCIÓ DE L'ÚS FETA PER L'INFORMANT. És bo per a la pell. Se’n fan fregues.
PREPARACIÓ. En un pot es fiquen les plantes en oli d'oliva i es deixa 40 dies en la foscor tapat amb una gasa. Es cola i es guarda en una ampolla de color ambre.

Per al refredat

**Per al refredat**

FONT 2184. El Prat de Llobregat (Baix Llobregat)

INGREDIENTS:

***Eryngium maritimum***L. (umbel·líferes) - Arrel

***Glycyrrhiza glabra***L. (papilionàcies) - Arrel

***Scirpus holoschoenus***L. (ciperàcies) - No consta

FORMA FARMACÈUTICA I ÚS. Vi medicinal (ús intern)
EFECTES SECUNDARIS. No consta.
DESTINACIÓ. Medicina humana.
PREPARACIÓ. Es feien bullir tots els ingredients en vi negre.
MODE D'UTILITZACIÓ/POSOLOGIA. Una tassa abans d'anar a dormir.

**Per al refredat**

FONT 2190. Sant Climent de Llobregat (Baix Llobregat)

INGREDIENTS:

***Bidens aurea***(Ait.) Sherff (compostes) - Part aèria florida

***Mentha ×gentilis***L. (labiades) - Fulla

***Thymus vulgaris***L. (labiades) - Part aèria florida

FORMA FARMACÈUTICA I ÚS. Infusió (ús intern)
EFECTES SECUNDARIS. No consta.
DESTINACIÓ. Medicina humana.

**Per al refredat**

FONT 2204. Gavà (Baix Llobregat)

INGREDIENTS:

***Sambucus nigra***L. (adoxàcies) - Inflorescència

***Vitis vinifera***L. (vitàcies) - Suc del fruit

FORMA FARMACÈUTICA I ÚS. Cataplasma (ús extern).

EFECTES SECUNDARIS. No consta.
DESTINACIÓ. Medicina humana.

PREPARACIÓ. S’havia d’agafar un mocador o drap, esquitxar-lo amb un poquet de vinagre, i, damunt d’unes brases mig apagades, s’hi tiraven flors de saüc, mentre s’aguantava el drap, aproximadament uns quatre dits per sobre, perquè s’impregnés de fum.

MODE D'UTILITZACIÓ/POSOLOGIA. A l’hora d’anar a dormir, el drap es col·locava damunt del pit.

Salutífer

**Per al cos**

FONT 2162. Viladecans (Baix Llobregat)

INGREDIENTS:

***Lavandula stoechas***L. (labiades) - Part aèria florida

***Malva sylvestris***L. (malvàcies) - Part aèria florida

***Rosmarinus officinalis***L. (labiades) - Part aèria florida

***Thymus vulgaris***L. (labiades) - Part aèria florida

FORMA FARMACÈUTICA I ÚS. Decocció (ús extern)
EFECTES SECUNDARIS. No consta
DESTINACIÓ. Medicina humana
DESCRIPCIÓ DE L'ÚS FETA PER L'INFORMANT. La seva dona, cada nit, feia un bullit amb moltes herbes, que venia bé per al cos.

**BARREGES ALIMENTÀRIES**

Beguda preparada amb aiguardent

**Licor de llet**

FONT 2211. Gavà (Baix Llobregat)

INGREDIENTS:

***Beta vulgaris***L. subsp. ***vulgaris*** var. ***crassa***(Alef.) Helm (amarantàcies) - Arrel

***Cinnamomum verum***J.Presl (lauràcies) - Escorça

***Citrus sinensis***(L.) Osbeck (rutàcies) - Fruit

CONSUMICIÓ. Es beu quan és freda.
DESTINACIÓ. Alimentació humana.
PREPARACIÓ. Els ingredients són: un litre d’alcohol de 98º; un litre de llet fresca; una branca de canyella; 600 grams de sucre; sis taronges (només la pell de taronja raspada). Es té uns dos o tres mesos macerant en flascons grans tapats amb un drap. Passat aquest temps es filtra amb una gasa.

Beguda preparada amb licor

**Anís de cireres**

FONT 2190. Sant Climent de Llobregat (Baix Llobregat)

INGREDIENTS:

***Pelargonium citrosum*** Voigt ex Breiter (geraniàcies) - Fulla

***Prunus avium***(L.) L. (rosàcies) - Fruit

DESTINACIÓ. Alimentació humana.

**Anís de cireres**

FONT 2192. Sant Climent de Llobregat (Baix Llobregat)

INGREDIENTS:

***Prunus avium***(L.) L. (rosàcies) - Fruit

***Rosmarinus officinalis***L. (labiades) - Tija amb fulles/branques ***Satureja fruticosa***(L.) Briq. (labiades) - Tija amb fulles/branques

***Thymus vulgaris***L. (labiades) - Tija amb fulles/branques

CONSUMICIÓ. Es beu aquest licor en festes i grans dinars familiars.
DESTINACIÓ. Alimentació humana.
PREPARACIÓ. Es posen cireres fortes en un pot amb anís, farigola, romaní i poniol. Es poden posar amb peduncle [dit "cua"] o sense. Es pot substituir l’anís per conyac o, fins i tot, whisky, tot i que queda millor amb anís sec.

Conserva en salmorra

**Olives arreglades**

FONT 2190. Sant Climent de Llobregat (Baix Llobregat)

INGREDIENTS:

***Ceratonia siliqua***L. (papilionàcies) - Part aèria jove

***Olea europaea***L. subsp. ***europaea*** var. ***europaea***(oleàcies) - Fruit ***Satureja montana***L. (labiades) - Tija amb fulles/branques

CONSUMICIÓ. Se'n mengen com a aperitiu.
DESTINACIÓ. Alimentació humana.
DESCRIPCIÓ DE L'ÚS FETA PER L'INFORMANT. Adobat d'olives.
PREPARACIÓ. Es posen les olives uns 15 dies en aigua salada amb algun brot de garrofer. Passats aquests 15 dies, es treuen i s'arreglen amb sajolida i sal.

**Olives arreglades**

FONT 2192. Sant Climent de Llobregat (Baix Llobregat)

INGREDIENTS:

***Allium sativum***L. (amaril·lidàcies) - Bulb

***Citrus limon***(L.) Burm. (rutàcies) - Fruit

***Foeniculum vulgare***Mill. subsp. ***piperitum***(Ucria) Cout. (umbel·líferes) - Tija amb fulles/branques

***Olea europaea***L. subsp. ***europaea*** var. ***europaea***(oleàcies) - Fruit ***Satureja montana***L. (labiades) - Tija amb fulles/branques

CONSUMICIÓ. Abans o durant els àpats.
DESTINACIÓ. Alimentació humana.
PREPARACIÓ. Durant nou setmanes l’aigua es canvia cada dia; en acabat, s’hi posa sal i un tros de llimona a sobre. Es fan bullir sajolida i fonoll i l’aigua es posa en pots amb les olives quan es refreda. També s’hi pot posar all.

Cuita en aigua

**Peus de porc amb naps**

FONT 2212. Gavà (Baix Llobregat)

INGREDIENTS:

***Brassica napus***L. (crucíferes) - Arrel

***Laurus nobilis***L. (lauràcies) - Fulla

***Piper nigrum***L. (piperàcies) - Fruit

***Triticum aestivum***L. (gramínies) - Trituració del gra

CONSUMICIÓ. Recepta que es feia el dia de Sant Antoni Abat, el 17 de gener. DESTINACIÓ. Alimentació humana.
PREPARACIÓ. Es deixen reposar els peus de porc amb sal a la nevera durant tres dies. S’esbandeixen en el moment de cuinar-los. Es posa a coure en olla exprés amb els naps, altres verdures, llorer i pebre. Un cop freds els peus, s’enfarinen i es fregeixen.

Cuita en aigua i oli

**Conserva de tomàquet**

FONT 2192. Sant Climent de Llobregat (Baix Llobregat)

INGREDIENTS:

***Allium sativum***L. (amaril·lidàcies) - Bulb

***Olea europaea***L. subsp. ***europaea*** var. ***europaea***(oleàcies) - Oli de la llavor ***Solanum lycopersicum***L. (solanàcies) - Fruit

CONSUMICIÓ. No consta.
DESTINACIÓ. Alimentació humana.
PREPARACIÓ. Es renten i es posen a l’olla, amb sal, oli i grans d’all. S’hi posa mig got d’aigua i es deixa bullir. Es deixa refredar i s’envasa. El [tomàquet] de pera és el millor per a fer conserva.

**Platillo de Sant Climent**

FONT 2192. Sant Climent de Llobregat (Baix Llobregat)

INGREDIENTS:

***Castanea sativa***Mill. (fagàcies) - Llavor

***Corylus avellana*** L. (betulàcies) - Bessó

***Olea europaea***L. subsp. ***europaea*** var. ***europaea***(oleàcies) - Oli de la llavor ***Pinus pinea*** L. (pinàcies) - Llavor

***Prunus avium***(L.) L. (rosàcies) - Fruit

***Prunus domestica***L. (rosàcies) - Fruit

***Prunus dulcis***(Mill.) Weeb. (rosàcies) - Bessó

CONSUMICIÓ. Plat principal
DESTINACIÓ. Alimentació humana
PREPARACIÓ. Recepta tradicional que es fa per la festa major (23 de novembre) i Nadal (25 de desembre). Porta cireres seques, costella de porc, salsitxes, pollastre en trossos petits, rovellons, prunes seques, pinyons, picada d’ametlles, avellanes i fetge de conill, castanyes i botifarra negra.

Cuita en vi

**Arrop**

FONT 2192. Sant Climent de Llobregat (Baix Llobregat)

INGREDIENTS:

***Beta vulgaris***L. subsp. ***vulgaris*** var. ***crassa***(Alef.) Helm (amarantàcies) - Arrel

***Solanum melongena***L. (solanàcies) - Fruit

***Vitis vinifera***L. (vitàcies) - Fruit

CONSUMICIÓ. No consta.
DESTINACIÓ. Alimentació humana.
PREPARACIÓ. Albergínies en trossos i grans de raïm, tot plegat bullit en vi amb sucre. Es tracta d’una melmelada compacta de color negre.

**Arrop**

FONT 2184. El Prat de Llobregat (Baix Llobregat)

INGREDIENTS:

***Beta vulgaris***L. subsp. ***vulgaris*** var. ***crassa***(Alef.) Helm (amarantàcies) - Arrel

***Citrullus lanatus***(Thunb.) Matsumara et Nakai (cucurbitàcies) - Fruit ***Citrus limon***(L.) Burm. (rutàcies) - Fruit

***Cucumis melo***L. subsp. ***melo***(cucurbitàcies) - Fruit

CONSUMICIÓ. No consta
DESTINACIÓ. Alimentació humana
DESCRIPCIÓ DE L'ÚS FETA PER L'INFORMANT. És una confitura de fruita feta bàsicament amb vi, sucre, pell de meló, pell de llimona i pell de síndria.

**Arrop**

FONT 3700. El Prat de Llobregat (Baix Llobregat)

INGREDIENTS:

***Beta vulgaris***L. subsp. ***vulgaris*** var. ***crassa***(Alef.) Helm (amarantàcies) - Arrel

***Prunus avium***(L.) L. (rosàcies) - Fruit

CONSUMICIÓ. No consta
DESTINACIÓ. Alimentació humana
PREPARACIÓ. Molt típic de Sant Climent. Es feien amb diverses races de fruita, sobretot de cirera, que es feien bullir amb vi dolç i sucre, i quedava com una mena de confitura.

Cuita sense vehicle

**Pa de Sant Nicasi**

FONT 3947. Gavà (Baix Llobregat)

INGREDIENTS:

***Pimpinella anisum***L. (umbel·líferes) - Fruit

***Triticum aestivum***L. (gramínies) - Trituració del gra

CONSUMICIÓ. És una tradició local molt arrelada el fet de fabricar uns panets especials per la diada de Sant Nicasi, el 14 de desembre.
DESTINACIÓ. Alimentació humana
OBSERVACIONS. Es tenia la creença que protegien contra les malalties contagioses. Per a fer aquests pans, cada veí donava una part de la farina, excepte els pobres, que rebien el pa de franc. Els panets, un cop cuits, els portaven a l’església, on eren beneïts pel senyor rector i, tot seguit, eren repartits.
